# Blood metabolic biomarkers and colorectal cancer risk: results from large prospective cohort and Mendelian randomisation analyses

**DOI:** 10.1038/s41416-025-02997-4

**Published:** 2025-04-30

**Authors:** Fangcheng Yuan, Guochong Jia, Wanqing Wen, Shuai Xu, Valerie Gunchick, Kui Deng, Jirong Long, Danxia Yu, Xiao-Ou Shu, Wei Zheng

**Affiliations:** Division of Epidemiology, Department of Medicine, Vanderbilt Epidemiology Center, Vanderbilt-Ingram Cancer Center, Vanderbilt University Medical Center, Nashville, TN USA

**Keywords:** Cancer epidemiology, Epidemiology, Risk factors

## Abstract

**Background:**

Emerging evidence suggests metabolic dysregulation may contribute to colorectal cancer (CRC) aetiology. We aimed to identify pre-diagnostic metabolic biomarkers for CRC risk in 230,420 UK Biobank participants.

**Methods:**

Nuclear magnetic resonance spectroscopy was used to quantify 249 metabolic biomarkers in plasma samples collected at baseline. Cox proportional hazards models were used to estimate hazard ratios and 95% confidence intervals (CIs) for associations of metabolic biomarkers with CRC risk after adjusting for potential confounders. To infer the potential causality of biomarkers that were associated with CRC independent of the others, we performed genome-wide association analyses among 199,732 UK Biobank participants of European ancestry to identify biomarker-associated genetic variants, followed by two-sample Mendelian randomization (MR) analyses using summary statistics of 78,473 CRC cases and 107,143 controls of European ancestry.

**Results:**

During a median follow-up time of 9.7 years, 2,410 incident primary CRC cases were identified. Among 43 CRC-associated (*P*-value < 0.001) metabolic biomarkers, ten biomarkers including fatty acids (FAs), inflammation, ketone bodies, and lipoprotein lipids were associated with CRC risk after mutual adjustment. MR analyses provided strong evidence for potential causal associations of CRC risk with percentages of linolic acid [odds ratio (OR) = 0.89, 95% CI = 0.83-0.96, *P*-value = 3 × 10^-3^] and saturated FAs (OR = 1.14, 95% CI = 1.03–1.25, *P*-value = 9  ×  10^-3^) to total FAs.

**Conclusions:**

We identified multiple CRC-associated metabolic biomarkers. Perturbed lipid and lipoprotein metabolism may promote colorectal carcinogenesis.

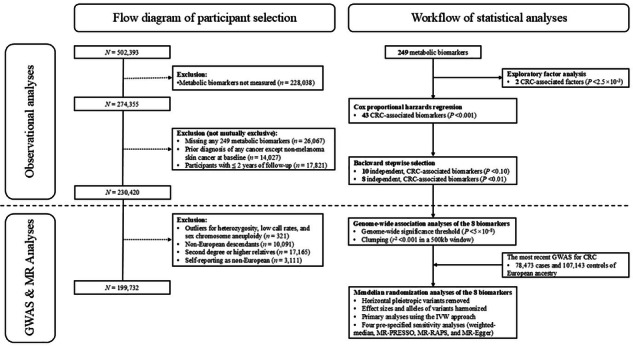

## Introduction

Globally, colorectal cancer (CRC) is the third most common cancer and the second-leading cause of cancer mortality [1]. Altered energy metabolism and metabolic reprogramming have been recognised as key contributors to tumorigenesis, including that of the colorectum [2]. These experimental evidence are further supported by epidemiologic studies, in which metabolic syndrome and its determinants (e.g., obesity, physical inactivity, unhealthy dietary patterns) are found to be associated with an elevated CRC risk [3]. One major component of metabolic syndrome is dyslipidemia, which is characterised by several lipid and lipoprotein abnormalities including increased levels of total triglycerides and low-density lipoprotein (LDL) cholesterol and decreased levels of high-density lipoprotein (HDL) cholesterol [4].

Metabolomics has been increasingly incorporated into epidemiologic studies to improve understanding of disease aetiology and uncover novel biomarkers for targeted cancer prevention [5]. However, previous epidemiologic investigations on the associations of pre-diagnostic metabolite levels with CRC risk typically included small numbers of participants and reported inconsistent findings [6–9]. To address this research need, we analysed data from 230,420 participants in the UK Biobank and investigated associations between 249 metabolic biomarkers and incident CRC risk.

Mendelian randomization uses genetic variants as proxies for exposures and evaluated their potential causal effects on outcomes [10]. This approach minimises biases such as residual confounding and reverse causation to which observational studies are known to subject, provided key assumptions (i.e., relevance, independence, exclusion restriction) are satisfied [11]. To further infer potential causality of CRC-associated biomarkers, we conducted two-sample Mendelian randomization (MR) analyses using summary statistics of 78,473 CRC cases and 107,143 controls of European ancestry from a recent genome-wide association study (GWAS) meta-analysis for CRC (2023) [12]. Genetic instruments for this MR analysis were selected from a GWAS of metabolite biomarkers among 199,732 UK Biobank participants of European ancestry.

## Materials and methods

### Study design and population

Between 2006 and 2010, over 502,000 adults aged 40–70 years from 22 assessment centres across England, Wales, and Scotland were recruited into UK Biobank, a population-based prospective cohort study aiming to identify determinants of complex diseases of middle and old age. Details about the UK Biobank study design have been described elsewhere [13]. Briefly, at baseline, participants provided information on sociodemographic characteristics, health and medical history, and lifestyle factors, underwent physical examinations, and provided blood samples. Among 274,355 participants with metabolic biomarker measurements (Supplementary Fig. S1), we excluded individuals with missing measurements for any of the 249 metabolic biomarkers (*n* = 26,067), diagnosed with any cancer except non-melanoma skin cancer at baseline (*n* = 14,027), and with less than two years of follow-up (*n* = 17,821). In total, 230,420 participants remained after these exclusions.

### Metabolic biomarker profiling

Non-fasting plasma samples were measured using a targeted high-throughput nuclear magnetic resonance (NMR) metabolomics [5, 14] developed by Nightingale Health Plc [15]. The platform simultaneously quantified 249 metabolic biomarkers (Supplementary Table S1), including 168 absolute levels (mostly in mmol/L) and 81 ratios/percentages that captured cholesterol metabolism, lipid concentrations and composition within 14 lipoprotein subclasses, fatty acid (FA) compositions, and various low-molecular-weight metabolites (e.g., glycolysis-related metabolites, ketone bodies, and amino acids). The coefficients of variation for biomarkers are generally <5% [15]. Quality control was performed for the metabolomics data to remove unwanted technical variation (e.g., batch effects) resulting from difference in sample handling and measurement [16].

### Ascertainment of incident colorectal cancer cases

Cancer cases in the UK Biobank were identified via linkage to national cancer registries. Information on incident cancer diagnoses was obtained from the National Health Service (NHS) Information Center for participants from England and Wales (follow-up through February 29th, 2020 and December 31st, 2016, respectively) and from the NHS Central Register Scotland for participants from Scotland (follow-up through January 31st, 2021). The primary outcome was the first diagnosis of incident primary CRC based on the International Classification of Disease, 10th Revision (ICD-10) codes C18-C20. Three CRC subsites (proximal colon, distal colon, rectum) were also defined according to anatomical locations (Supplementary Methods).

### Measurement of baseline covariates

Data on sociodemographics (e.g., age, sex, race, educational attainment), medical history (e.g., family and screening histories of CRC, cholesterol-lowering medication use), and lifestyles (e.g., dietary intake, alcohol consumption, tobacco smoking) were collected via self-administered touchscreen questionnaires and nurse-led interviews at baseline. Trained staff used calibrated instruments to measure height (cm), weight (kg), and waist and hip circumferences (cm), from which body mass index (BMI, kg/m^2^) and waist-to-hip ratio were derived. Fasting time was recorded at the time of blood collection.

### Statistical analyses

The overall workflow of the current analysis is presented in Supplementary Fig. S1. Baseline characteristics were described using medians for continuous variables and proportions for categorical variables. We used the Wilcoxon rank-sum test and Pearson *χ*^2^ test to assess differences between cases and non-cases for continuous and categorical variables, respectively. Values of 249 metabolic biomarkers were standardised to the same scale by *z* scores [mean = 0, standard deviation (SD) = 1]. Hazard ratios (HRs) and 95% confidence intervals (CIs) describing the associations of standardised metabolic biomarker levels with CRC risk were estimated using Cox proportional hazards models with age as the time scale. To minimise possible bias due to reverse causation (i.e., change in metabolic biomarker levels due to CRC development), we excluded the first two years of follow-up time including CRC cases diagnosed in this period. Participants were censored at the date of first CRC diagnosis, diagnosis of primary cancers of other sites, death, loss to follow-up, or the censoring date [February 29th, 2020 (England), December 31st, 2016 (Wales), and January 31st, 2021(Scotland)], whichever occurred first. The proportional hazards assumption was checked using statistical tests based on the scaled Schoenfeld residuals and no violation was detected. The base model was adjusted for sex (female, male), race (White, Other), educational attainment (college/university degree, some professional qualifications, secondary education, none of the above), fasting time (<3 h, 3–5 h, >5 h), cholesterol-lowering medication use (yes, no), CRC family history (yes, no) and CRC screening history (ever, never). Since ~95% participants were self-identified as White, we combined Black, South Asian, Chinese, and mixed/others into a single category. The full model was additionally adjusted for alcohol consumption (never/seldom, 1–4 times per week, >4 times per week), tobacco smoking [never, former/current (light), current (heavy)], fruits and vegetables intake (≤1 serving/day, 2–4 servings/day, ≥5 servings/day), and processed and red meat intake (≤4 points, 5–7 points, ≥8 points). The processed and red meat intake were derived by summing the frequency of intake across four variables (i.e., processed meat, beef, lamb/mutton, and pork), each of which was coded as never (0 point), less than once a week (1 point), once a week (2 points), 2–4 times a week (3 points), 5-6 times a week (4 points), once or more daily (5 points) [17]. Adiposity-related traits (i.e., BMI, waist-to-hip ratio) and physical activity were not adjusted for as they may contribute to CRC development via altering blood lipids and lipoproteins levels. We imputed covariates with missing values (<5%) using the median for continuous variables and the mode for categorical variables. To determine metabolic biomarkers that were associated with CRC risk independent of the others, among all CRC-associated biomarkers in the base model, we first performed backward stepwise Cox proportional hazards regression to select biomarkers within the same class [e.g., triglycerides, fatty acids (FAs)] using a *P*-value < 0.10 as the inclusion criterion. All retained biomarkers were then included in the final, cross-class backward stepwise Cox model for further selection. Covariates in the base model were forced into the backward stepwise Cox models.

Several sensitivity analyses were conducted to evaluate finding robustness. First, we excluded participants who reported using cholesterol-lowering medications at baseline because these medications may alter biomarker levels and bias their associations with CRC risk [18]. Second, to assess the extent to which biomarker outliers influenced results, we removed biomarker levels outside of four interquartile ranges from the median [19] and reanalysed associations in the remaining dataset. Third, rather than excluding the 26,067 individuals with missing measurements for any biomarkers, we kept them in a larger study sample and re-evaluated associations. Fourth, we imputed covariates with missing values using the sex-specific median or mode to consider possible differences in their distributions by sex. We also performed stratified analyses by sex and anatomic subsite of CRC (Supplementary Methods).

We conducted an exploratory factor analysis using varimax rotation to reduce multicollinear data on the 249 biomarkers into a smaller number of uncorrelated and interpretable factors (i.e., metabolic patterns) [20]. The number of factors to extract (*N* = 20) was determined by the parallel analysis in which eigenvalues of observed data were compared against those of a randomly generated correlation matrix of the same size [21]. Cox proportional hazards models were used to estimate associations of the 20 factors with CRC risk, adjusting for the same sets of covariates. All statistical tests were two-sided. To account for the high correlation among the NMR biomarkers, we performed a principal component (PC) analysis and calculated the number of PCs (i.e., independent tests, *N* = 50) that explained >99.5% of total variance in the 249 biomarkers (Supplementary Table S2) [22, 23]. Hence, we considered a *P*-value < 1 × 10^−3^ (0.05/50) statistically significant which was in line with previous publications [5, 19]. A Bonferroni-corrected *P*-value < 2.5 × 10^−3^ (0.05/20) was considered statistically significant for factor-CRC associations. All analyses were performed in Stata version 14 (StataCorp) and R version 4.2.2.

### Genome-wide association and Mendelian randomization analyses

After conducting sample-level filtering and excluding 30,688 participants from the analytic dataset (Supplementary Figure S1), we performed GWASs among 199,732 UK Biobank participants of European ancestry using the PLINK 2.0 (https://www.cog-genomics.org/plink/2.0/) [24] to identify genetic variants associated with the following eight metabolic biomarkers that showed the most significant (*P*-value < 0.01 in the final backward stepwise Cox model) associations with CRC risk after mutual adjustment: triglycerides to phosphoglycerides ratio, percentages of linoleic acid, saturated FA (SFA), and omega-6 FA to total FAs, concentrations of glycoprotein acetyls and 3-hydroxybutyrate, and percentages of cholesterol to total lipids in small LDL and triglycerides to total lipids in intermediate-density lipoprotein (IDL). The values of triglycerides to phosphoglycerides ratio, 3-hydroxybutyrate, triglycerides to total lipids in IDL percentage were log-transformed to normalise their distributions. Details about genotyping, genetic imputation, and quality control in the UK Biobank were provided in the Supplementary Methods. Among >90 million genetic markers in the final imputed dataset, 8,639,989 were analysed after quality control. The linear regression model was adjusted for age, sex, genotyping array, and the first ten PCs for population structure. Compared with previous GWAS of NMR metabolic biomarkers [25, 26], our analysis included a larger sample size and hence improved the statistical power to identify associated genetic variants.

We conducted two-sample MR analyses [10, 27] to infer potential causality of the eight CRC-associated biomarkers. Principles and underlying assumptions of MR were described in the Supplementary Methods. Independent (*r*^2^ < 0.001 in a 500 kb window) and genome-wide significant (*P* value < 5 × 10^-8^) variants from aforementioned GWASs for biomarkers were used as genetic instruments in the MR analyses using summary statistics from a recent GWAS meta-analysis for CRC (2023) [12], which included 78,473 cases and 107,143 controls from 80 studies (17 analytical units) in European descent populations and 8,782,440 variants with *I*^2^ ≤ 65%. The GWAS meta-analysis included 25,089 (4,800 CRC cases and 20,289 controls) UK Biobank participants. The primary MR analysis was conducted using the inverse-variance weighted (IVW) approach under a multiplicative random-effects model [28], supplemented by weighted-median [29], MR-pleiotropy residual sum and outlier (MR-PRESSO) [30], MR-robust adjusted profiles score (MR-RAPS) [31], and MR-Egger [32] as sensitivity analyses. Complete descriptions of GWAS and MR analyses are in the Supplementary Methods.

## Results

During a median follow-up of 9.7 years, 2410 incident CRC cases were identified among 230,420 UK Biobank participants included in the current analysis. Compared with non-cases at baseline, CRC cases were older, had a higher BMI and waist-to-hip ratio, consumed more processed and red meat, drank alcohol more frequently, more likely to be male, White, have a family history of CRC, have received CRC screening, and use cholesterol-lowering medications. They were less likely to be never-smokers and attain college/university degrees (Table 1).

**Table 1:** Selected baseline characteristics of study participants by study groups in the UK Biobank

Characteristics		Colorectal cancer		
	All subjects (*N* = 230,420)	Cases (*N* = 2410)	Non-cases (*N* = 228,010)	*P*-value
Age (year), median (25th–75th percentile)	58 (50–63)	62 (56–66)	57 (50–63)	<0.001
Male sex, %	47.4	58.7	47.3	<0.001
White race, %	94.8	96.5	94.8	<0.001
Education, %				<0.001
College/university degree	32.8	30.7	32.9	
Some professional qualifications	27.7	27.3	27.7	
Secondary education	22.1	21.4	22.1	
None of the above	17.4	20.6	17.3	
BMI (kg/m^2^), median (25th – 75th percentile)	26.9 (24.3–30.0)	27.6 (25.1–30.5)	26.9 (24.3–30.0)	<0.001
Waist-to-hip ratio, median (25th–75th percentile)	0.88 (0.81–0.94)	0.91 (0.83–0.96)	0.88 (0.81–0.94)	<0.001
Physical activity^a^, %				0.16
None	18.1	19.7	18.1	
Intermediate	68.0	66.8	68.0	
Intense	13.9	13.5	13.9	
Processed/red meat intake^b^, %				<0.001
≤4 points	29.0	23.9	29.0	
5–7 points	51.7	52.7	51.7	
≥8 points	19.3	23.4	19.3	
Fruit/vegetables intake, %				0.87
≤1 serving per day	4.2	4.2	4.2	
2–4 servings per day	54.5	55.0	54.4	
≥5 servings per day	41.3	40.8	41.4	
Alcohol consumption, %				<0.001
Never or seldom^c^	30.8	28.1	30.8	
1–4 times per week	49.7	48.3	49.7	
Almost daily	19.5	23.6	19.5	
Tobacco smoking, %				<0.001
Never	55.2	47.1	55.3	
Former or current, light^d^	37.5	45.4	37.4	
Current, heavy	7.3	7.5	7.3	
Family history of colorectal cancer, %	10.8	14.8	10.8	<0.001
Ever received colorectal cancer screening, %	30.0	35.0	29.9	<0.001
Cholesterol-lowering medication use, %	17.2	24.2	17.1	<0.001
Fasting time (h), %				0.23
<3 h	25.5	25.5	25.5	
3–5 h	63.5	62.4	63.5	
>5 h	11.0	12.1	11.0	

Abbreviations: BMI, body mass index.

^a^Intense physical activity is defined as having 6–7 days/week of moderate activity and 3–5 days/week of vigorous activity, or having 6–7 days/week of vigorous activity; intermediate physical activity is defined as having 1-5 days/week of moderate activity, or 1–2 days/week of vigorous activity.

^b^This variable was summed across four variables measuring frequency of intakes of processed meat, beef, lamb/mutton, and pork. The original four variables were coded as: never (0), less than once a week (1), once a week (2), 2–4 times a week (3), 5–6 times a week (4), once or more daily (5). The derived variable had a range from 0 to 20 points and was categorised into three levels.

^c^Seldom alcohol consumption is defined as special occasions only, or 1–3 times a month.

^d^Light current smoking is defined as smoking <20 pack-years; heavy current smoking is defined as smoking ≥20 pack-year.

**Fig. 1:**
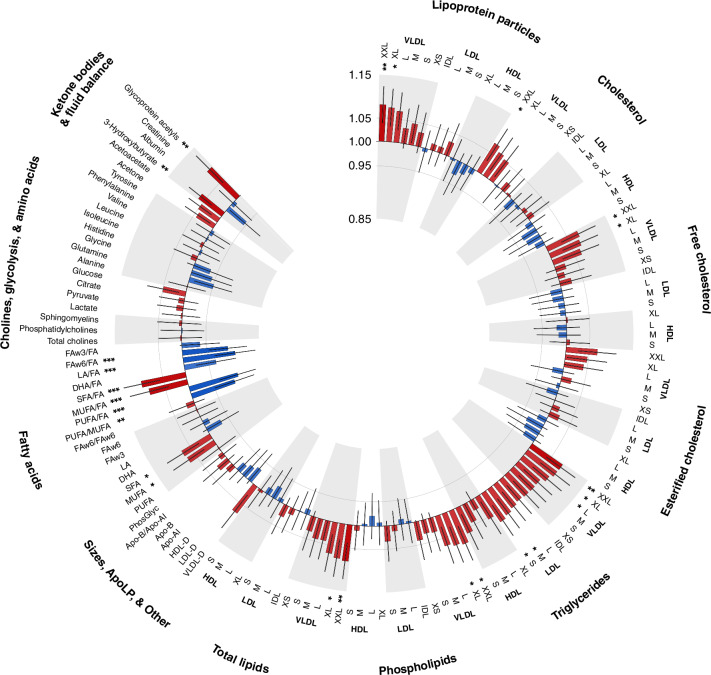
Associations between selected metabolic biomarkers and incident colorectal cancer risk in the UK biobank cohort. Hazard ratios (HR) and 95% confidence intervals (CIs) for 1-standard deviation (SD) increase in biomarker levels were estimated after adjusting for sex, race, educational attainment, fasting time, self-reported use of cholesterol-lowering medications, colorectal cancer family history and colorectal cancer screening history. *P-value < 1×10^−3^. **P-value < 1 × 10^-4^. ***P-value < 1 × 10^−5^. Apo-A1 apolipoprotein A1, Apo-B apolipoprotein B, DHA docosahexaenoic acid, FA fatty acids, FAw3 omega-3 fatty acids, FAw6 omega-6 fatty acids, HDL high-density lipoproteins, HDL-D high-density lipoprotein particle diameter, IDL intermediate-density lipoproteins, L large, LA linoleic acid, LDL low-density lipoproteins, LDL-D low-density lipoprotein particle diameter, LP lipoprotein, M medium, MUFA monounsaturated fatty acids, PUFA polyunsaturated fatty acids, S small, SFA saturated fatty acids, VLDL very low-density lipoproteins, VLDL-D very low-density lipoprotein particle diameter, XL very large, XS very small, XXL extremely large.

Figure 1 depicts the association patterns for 143 metabolic biomarkers selected to represent various biomarker classes. The selected biomarkers were directly measured in absolute concentrations and could not be derived otherwise, except for measures of fatty acids and apolipoproteins. Positive associations with CRC risk were seen for levels of SFA and MUFA, glycoprotein acetyls, 3-hydroxybutyrate, and very and extremely large very low-density lipoprotein (VLDL) particles and the lipid contents within them. In most lipoprotein subclasses, concentrations of triglyceride were also positively associated with CRC risk. In addition, inverse associations were seen for percentages of linoleic acid, polyunsaturated fatty acids (PUFA), and omega-6 FA to total FAs, PUFA to monounsaturated fatty acids (MUFA) ratio, and degree of unsaturation. We also observed non-significant inverse associations with CRC risk for concentrations of HDL particles, particularly medium- and large-sized, and cholesterol contents within them.

Of the 249 metabolic biomarkers, 43 were associated with incident CRC risk at a *P*-value < 1 × 10^−3^ in the base model (Supplementary Table S3). The correlation matrix for significant biomarkers is shown in Supplementary Fig. S2. After additionally adjusting for lifestyle covariates, 19 biomarkers remained significantly associated with CRC risk (*P*-value < 1 × 10^−3^, Supplementary Table S3). Ten metabolic biomarkers were identified to be associated with CRC risk (*P*-value < 0.10) after mutually adjusting for other significant biomarkers using the backward stepwise Cox model (Supplementary Table S4). Their individual associations with CRC risk after adjusting for potential confounders in base and full models are shown in Table 2.

**Table 2:** Associations of ten selected metabolic biomarkers with colorectal cancer risk, the UK Biobank cohort^a^

Metabolic biomarker	HR^b^ (95%CI)	*P*-value	HR^c^ (95%CI)	*P*-value
Triglycerides to Phosphoglycerides (ratio)	1.09 (1.04–1.13)	7.19 × 10^−5^	1.08 (1.04–1.13)	2.43 × 10^−4^
Linoleic Acid to Total Fatty Acids (%)	0.90 (0.86–0.94)	5.17 × 10^−7^	0.91 (0.87–0.95)	2.55 × 10^−5^
Saturated Fatty Acids to Total Fatty Acids (%)	1.10 (1.06–1.14)	1.51 × 10^−6^	1.08 (1.04–1.13)	7.49 × 10^−5^
Omega−6 Fatty Acids to Total Fatty Acids (%)	0.91 (0.87–0.95)	2.87 × 10^−6^	0.92 (0.89–0.96)	1.00 × 10^−4^
Glycoprotein Acetyls (mmol/L)	1.09 (1.05–1.13)	3.18 × 10^−5^	1.08 (1.03–1.12)	2.95 × 10^−4^
3-Hydroxybutyrate (mmol/L)	1.06 (1.03–1.10)	7.82 × 10^−5^	1.06 (1.03–1.09)	2.16 × 10^−4^
Concentration of Chylomicrons and Extremely Large VLDL Particles (mmol/L)	1.08 (1.04–1.12)	8.15 × 10^−5^	1.07 (1.03–1.11)	5.82 × 10^−4^
Triglycerides in Chylomicrons and Extremely Large VLDL (mmol/L)	1.08 (1.04–1.12)	4.13 × 10^−5^	1.07 (1.03–1.11)	3.44 × 10^−4^
Cholesterol to Total Lipids in Small LDL (%)	0.93 (0.90–0.96)	7.46 × 10^−6^	0.93 (0.90–0.96)	1.19 × 10^−5^
Triglycerides to Total Lipids in IDL (%)	1.07 (1.03–1.12)	5.01 × 10^−4^	1.07 (1.02–1.11)	2.00 × 10^−3^

*CI* confidence interval; *CRC* colorectal cancer; *HR* hazard ratio; *IDL* intermediate-density lipoprotein; *LDL* low-density lipoprotein; *SD* standard deviation; *VLDL* very low-density lipoprotein.

^a^Biomarkers presented in the table were selected by the final backward stepwise Cox model (*P*-value < 0.10).

^b^HRs and 95% CIs for 1-SD increase in biomarker levels were estimated using the base Cox model with the age as the time scale, adjusting for sex, race, educational attainment, fasting time, self-reported cholesterol-lowering medication use, and CRC family history and CRC screening history.

^c^The full Cox model was additionally adjusted for alcohol drinking, tobacco smoking, fruit/vegetables intake, and processed/red meat intake.

In sensitivity analyses, effect estimates for these ten metabolic biomarkers remained essentially unchanged among 190,857 (including 1827 CRC cases) non-users of cholesterol-lowering medications, albeit their statistical significance level attenuated possibly due to a reduced sample size (Supplementary Table S5). Except for 3-hydroxybutyrate, associations for the other nine biomarkers were mostly preserved after excluding biomarker outliers (≤3.3%) from (Supplementary Table S6) or including participants with incomplete biomarker measurements in the analysis (Supplementary Table S7). Using sex-specific information to impute missing covariates did not alter the findings (Supplementary Table S8). In stratified analyses (Supplementary Tables S9-S10), the ten biomarkers were more strongly associated with CRC risk in males than females, especially for the three fatty acid measures (*P*_heterogeneity_ ≤ 0.02 for all). Their associations did not differ significantly across the three CRC subsites.

**Fig. 2:**
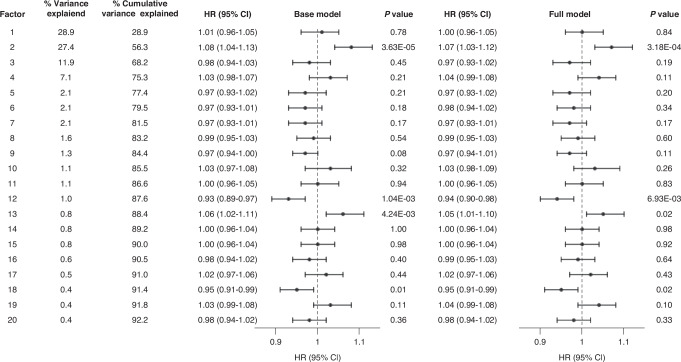
Associations of the first 20 factors with incident colorectal cancer risk in the UK Biobank. Hazard ratios (HRs) and 95% confidence intervals (CIs) were estimated for 1-unit increase in factor value. The base model was adjusted for sex, race, educational attainment, fasting time, self-reported use of cholesterol-lowering medications, colorectal cancer family history and colorectal cancer screening history. The full model was additionally adjusted for alcohol drinking, tobacco smoking, fruits/vegetables intake, and processed/red meat intake.

The first 20 factors collectively explained >92% of the total variance in 249 metabolic biomarkers (Fig. 2). Factor 2 (HR = 1.08, 95% CI = 1.04-1.13, *P*-value = 3.63 × 10^−5^) was positively associated, while factor 12 (HR = 0.93, 95% CI = 0.89-0.97, *P*-value = 1.04 × 10^−3^) was inversely associated with CRC risk in the base model. Factor 13 was marginally associated with an increased CRC risk (HR = 1.06, 95% CI = 1.02–1.11, *P*-value = 4.24 × 10^−3^). Only factor 2 (HR = 1.07, 95% CI = 1.03-1.12, *P*-value = 3.18 × 10^−4^) remained significantly associated with CRC risk in the full model. The loadings of the three factors were depicted for top-contributing biomarkers in Supplementary Figs. S3–S5. Factor 2 was positively correlated with triglyceride contents in most lipoprotein subclasses, and concentrations of very and extremely large VLDL particles and their lipid constituents. Conversely, it was inversely correlated with percentages of cholesterol contents to total lipids in most lipoprotein subclasses, and percentages of omega-6 FA and PUFA to total FAs. Factor 12 contributed positively to concentrations of linoleic acid, and inversely to that of SFA. Factor 13 was positively related to levels of 3-hydroxybutyrate, acetoacetate, and acetone, ketone bodies that are products of fatty acid catabolism. Factor analysis results were generally consistent with individual biomarker results.

**Fig. 3:**
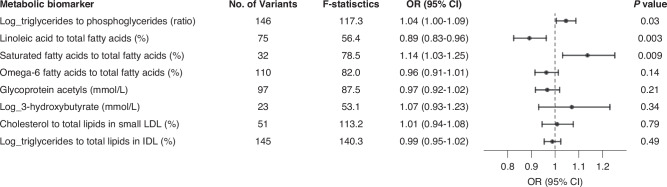
Mendelian randomization analyses of eight metabolic biomarkers selected from cohort analyses of the UK biobank data. Odds ratios (ORs) and 95% confidence intervals (CIs) were estimated per 1 standard deviation (SD) increase in biomarker levels. Levels of triglycerides to phosphoglycerides (ratio), 3-hydroxybutyrate (mmol/L), triglycerides to total lipids in IDL (%) were log-transformed before *z*-score standardisation.

We conducted GWASs in the UK Biobank to identify genetic determinants of standardised levels of eight selected metabolic biomarkers. In total, among 694 independent variants associated with any of these biomarkers at a *P*-value < 5 × 10^−8^, 323 variants had not been previously reported [25]. The proportion of novel variants for each biomarker ranged from 28 to 57% (Supplementary Table S11), and identified genetic variants were summarised in Supplementary Tables S12–S19. For each biomarker, the number of variants used to construct genetic instruments ranged from 23 to 146, and the *F*-statistic ranged from 53.1 to 140.3 (Supplementary Table S20). Genetic variants used in the MR analyses were provided in Supplementary Tables S21–S28. One-SD increase in genetically predicted triglycerides to phosphoglycerides ratio (log-transformed) was associated with a 4% [odds ratio (OR) = 1.04, 95% CI = 1.00–1.09, *P*-value = 0.03] higher CRC risk (Fig. 3). One-SD increase in genetically predicted percentages of SFA and linoleic acid to total FAs was associated with a respective 14% (OR = 1.14, 95% CI = 1.03–1.25, *P*-value = 9 × 10^−3^) increased and 11% (OR = 0.89, 95% CI = 0.83–0.96, *P*-value = 3 × 10^−3^) decreased CRC risk. MR-PRESSO did not detect any horizontal pleiotropic outliers, and the MR-Egger intercept test indicated no evidence of directional horizontal pleiotropy (all three *P*-values > 0.05). Results from four pre-specified sensitivity analyses were largely consistent with IVW estimates, suggesting our findings were robust to potential violations of different MR assumptions (Supplementary Table S20). Additionally, due to the concern that findings may be biased due to “winner’s curse” as instruments were selected based on their genetic associations in the dataset where they were initially discovered [33], we used a more stringent threshold (*P*-value < 5 × 10^−11^) to select instruments and re-estimated their associations. This sensitivity analysis did not considerably alter effect estimates. MR estimates for the other five biomarkers were not statistically significant.

## Discussion

To our knowledge, this is the first prospective cohort study to comprehensively evaluate associations of NMR-quantified metabolic biomarkers with CRC risk. Using data from 230,420 UK Biobank participants, we identified ten metabolic biomarkers representing a broad array of classes (e.g., lipoprotein lipids, FAs, ketone bodies, and inflammation) that were associated with CRC risk independent of one another. These findings were supported by factor analyses which uncovered two CRC-associated metabolic patterns. The MR analyses provided strong evidence for potential causal associations of percentages of saturated fatty acids and linolic acids to total FAs with CRC risk. Our results suggest aberrant lipids and lipoproteins metabolism may contribute to the aetiology of CRC.

To date, only a limited number of prospective studies have systematically interrogated the associations between blood metabolites and risks of incident CRC or its precursors [6–9, 34]. These metabolomics studies exclusively utilised a nested case-control design and mass spectrometry (MS)-based profiling technologies for metabolite quantification. In a Chinese study using pre-diagnostic non-fasting blood samples collected from 250 case–control pairs, 35 metabolites (including lipids, aromatic compounds, and organic acids) were associated with CRC risk [7]. The study identified 3 CRC-associated phosphatidylcholine species with opposite directions of association. In our study, the overall concentrations of phosphatidylcholines were not associated with CRC risk. It is possible that that this overall measure of phosphatidylcholines could have masked the associations of certain phosphatidylcholine species that are differentially associated with CRC risk [35]. Since specific phosphatidylcholine species were not measured on the NMR-based platform, we could not evaluate their associations with CRC risk. One recent study based on 517 non-fasting case-control pairs in the Cancer Prevention Study II Nutrition cohort identified six CRC-associated metabolites of diverse classes, including cofactors and vitamins, nucleotides, xenobiotics, lipids, and amino acids [8]. Another Swedish study including 902 case-control pairs (83% fasting) showed that 1-SD change in 3-hydroxybutyrate and valine levels were associated with 1.14- and 1.20-fold increased CRC risks [9]. In the current analysis, 3-hydroxybutyrate levels were positively associated, while valine levels were not associated with CRC risk. In a case-control study among US women using non-fasting samples to identify pre-diagnostic blood metabolites associated with CRC precursors, C36:3 phosphatidylcholine plasmalogen levels were inversely associated with risk of conventional adenomas, and C54:8 triglyceride levels were positively associated with risk of serrated polyps [34]. Evidence from prospective cohorts has shown a consistent positive association between triglyceride concentrations with CRC risk [36], including in the UK Biobank cohort [18]. Several other studies focused on specific blood metabolite classes, including cholines, FAs, and amino acids [37–42]. One study reported that 1-SD increase in levels of histidine and glutamine was associated with 20% and 15% reduced CRC risk among 654 fasting case-control pairs in the European Prospective Investigation into Cancer and Nutrition study, and 7% and 5% decreased risk in 111,323 non-fasting UK Biobank participants [42]. The findings were consistent with ours.

Our observational analyses showed that percentages of omega-6 FA, PUFA, and linoleic acid to total FAs were inversely associated, whereas the percentage of SFA to total FAs was positively associated with CRC risk. MR analyses provided strong evidence for a potential causal association of these SFA and linoleic acid biomarkers with CRC risk. These biomarkers were evaluated in previous studies using pre-diagnostic blood samples with inconsistent findings. For example, a study conducted among Singapore Chinese found that non-fasting participants in the highest quartile of linoleic acid had a 57% reduced colon cancer risk [39], which was consistent to our findings. Contrary to our results, that study found that palmitic acid (a type of SFA) level was inversely related to colon cancer risk. One Australian case-cohort study found that percentages of SFA and linoleic acid to total FAs were associated with an elevated CRC risk [38]. No associations were observed for PUFA and omega-6 FA. In a nested case-control study from 9 European countries, percentages of SFA and MUFA to total FAs were not associated with colon cancer risk [41]. Different study designs, FA measurement techniques, sample sizes, and covariate adjustments may contribute to discrepant findings across studies. In addition, some of the significant findings could be due to inflated type 1 error rate as not all studies adjusted for multiple comparisons. In contrast, three MR studies reported a consistent 4-5% reduced CRC risk per 1-SD increase in genetically predicted percentage of linoleic acid to total FAs [43–45]. Regarding SFA, genetically predicted percentage of stearic acid to total FAs was positively associated with CRC risk, whereas those of arachidic acid and palmitic acid were not [43]. Our findings for SFA and linolic acid were generally supported by prior studies.

FAs contribute to the initiation and progression of cancer via modulating immunity, inflammation, cell proliferation, apoptosis, and insulin sensitivity [46–48]. Although mechanistic underpinnings of the colonic neoplastic transformation in relation to SFA remain elusive, growing evidence from in vitro and murine studies show that SFA may promote CRC initiation via upregulating inflammatory gene expression [48], altering the tumorigenic capacity of intestinal stem and progenitor cells [49], and inducing gut microbial dysbiosis that perturbs immune homoeostasis [50] and elevates tumour-promoting metabolite levels [51]. In respect of PUFAs, omega-3 and omega-6 FAs are believed to have opposing effects on cancer [46]. Arachidonic acid, an omega-6 FA derived from its precursor linoleic acid after a series of desaturation reactions, is metabolised by cyclooxygenase-2 to produce pro-inflammatory eicosanoids such as prostaglandin E2, which has been shown to promote proliferation, suppress apoptosis, and silence tumour-suppressor and DNA-repair genes via epigenetic regulation [52, 53]. In contrast, omega-3 FA counteracts the pro-carcinogenic effects of omega-6 FA metabolism by suppressing the biosynthesis of arachidonic acid-derived eicosanoids [46]. We speculate that the protective effect of elevated percentage of linoleic acid to total FAs may reflect slow desaturase activity in the conversion of linoleic acid to arachidonic acid. Indeed, an association between 1-SD increase in genetically predicted percentage of arachidonic acid to total FAs and a 5-6% higher CRC risk was consistently reported across four MR studies [43–45, 54]. Furthermore, observational and MR studies reported that a high PUFA desaturase activity, estimated by the plasma arachidonic to linoleic acid ratio and proxied by a genetic variant at the *FADS* locus, was associated with an elevated CRC risk [39, 55]. Despite the lack of measurements on arachidonic acid and other specific PUFAs precludes further investigation in the UK Biobank, evidence from these studies has corroborated our hypothesis.

To our knowledge, this is the first prospective cohort study that reports a positive association of CRC risk with concentrations of larger VLDL particles and their lipid constituents, as well as triglyceride levels in lipoprotein subclasses. Epidemiologic studies on the association of lipid and lipoprotein subclasses with future cancer risk are still sparse. One Norwegian nested case-control study shows that concentrations of lipids in VLDL subfractions and total serum triglyceride were inversely associated with breast cancer risk among premenopausal but not postmenopausal women [56]. Emerging evidence from metabolomics studies links systemic metabolic disturbances to type 2 diabetes (T2D) [57]. Two recent large prospective studies leveraging NMR metabolite data from four Finnish cohorts and UK Biobank similarly identified an increased T2D risk among participants with elevated pre-diagnostic blood concentrations of larger VLDL subclass particles and their lipid components, along with higher triglyceride levels in all lipoprotein subclasses [58, 59]. Of note, these pathological alternations in lipoprotein metabolism were also characteristics of prevalent and incident hyperglycaemia and insulin resistance [4, 58, 60]. Insulin may promote tumour development via its mitogenic and anti-apoptotic effects and inducing low-grade chronic inflammation [61]. Deregulated glucose metabolism prompts metabolic transformation and alters signalling pathways and epigenetic modifications via increased reactive oxygen species and oncometabolites, facilitating tumorigenesis of the colon [62]. The carcinogenic effect of triglycerides has been shown using murine models, in which intestinal polyp formation was suppressed after experimentally lowering serum triglyceride levels [63, 64].

Of interest, we observed a potential causal relationship between the triglycerides to phosphoglycerides ratio and CRC risk. In the current analysis, plasma levels of total triglycerides were positively associated, whereas those of phosphoglycerides were not associated with CRC risk. It is likely that the identified association for this ratio measure was primarily driven by triglycerides. Previous nested case-control studies among Chinese and Europeans have reported inverse associations between blood levels of specific phosphoglycerides and incident CRC risk [7, 65]. Phosphoglycerides are the most abundant phospholipids and the major constituents of mammalian cell membranes [66]. Dysregulation of phosphoglycerides metabolism can perturb energy balance and contribute to metabolic disorders such as insulin resistance and obesity [66]. Furthermore, experimental evidence has suggested a potential anti-inflammatory effect of phosphoglycerides on the colorectum [67]. Our finding for the triglycerides to phosphoglycerides ratio warrants investigations in future epidemiologic and experimental studies.

This study has several unique strengths. Our systematic investigation of associations between pre-diagnostic plasma NMR metabolic biomarkers and CRC risk was complemented by MR analyses, which provide further evidence on plausible causality of FA metabolism in colorectal carcinogenesis. Other strengths include the prospective design and a long follow-up period. However, our study also has several limitations. First, the NMR-based platform only measured a small panel of the circulating metabolites in comparison to MS-based platforms. Second, although the use of non-fasting samples may introduce unwanted variation in measurements of most NMR metabolic biomarkers, their concentrations at fasting and postprandial timepoints have been found to be highly correlated [68]. Moreover, we controlled for time since last meal in regression models to account for the potential impact of food intake on biomarker levels. Third, given a relatively small number of CRC cases accrued over the study period, our subgroup analyses may be underpowered to detect associations. Fourth, since our selection of genetic instruments was solely based on a statistical threshold rather than a priori knowledge of their biological functions, the MR analyses may be subject to horizontal pleiotropy that results in violation of the exclusion restriction assumption. However, we took several remedial measures such as excluding potential pleotropic outliers and performing multiple sensitivity analyses to assess the robustness of findings. Fifth, although the “winner’s curse” could impinge on MR results because the same dataset was used to discover and select genetic instruments [33], its impact should be minimal given the large GWAS sample size. We also used a more stringent statistical threshold to select instruments in sensitivity analyses and found it did not alter effect estimates appreciably. Sixth, our MR findings may be biased due to a potential sample overlap between the exposure and outcome GWAS [69]. However, the magnitude of such a bias is likely negligible due to a small-scale overlap (<6.9% assuming random sampling for metabolic profiling) and strong instrumental strengths (all *F*-statistic ≥53.1). Finally, since the study populations of our observational and MR analyses were predominantly of White/European ancestry, findings may not be readily generalisable to other racial/ancestral groups.

In conclusion, we identified multiple CRC-associated metabolic biomarkers related to FAs and lipoprotein lipids in this prospective analysis of the UK Biobank cohort data. Our MR analysis provides additional evidence for a potential causal association of SFA and linolic acid measures with CRC risk. Elevated concentrations of triglyceride in lipoprotein subclasses as well as larger VLDL particles and their lipid compositions, which are characteristics of impaired insulin sensitivity and T2D, could also contribute to CRC aetiology. Our results for some metabolic biomarkers (e.g., triglycerides, linoleic acid to total FAs) are corroborated by previous studies. However, findings for other biomarkers are not consistent, resulting in part from different techniques used for metabolite measurements across studies. For instance, HDL cholesterol concentrations quantified by enzymatic assays were inversely associated with CRC risk in a prior analysis of the UK Biobank cohort [18], whereas in the current study NMR-quantified HDL cholesterol concentrations were not related to CRC risk. Furthermore, given that a relatively small number of metabolic biomarkers are measured on both NMR- and MS-based platforms, we were unable to evaluate certain MS-quantified biomarkers (e.g., specific phosphatidylcholine species) that had been differentially associated with CRC risk in prior studies. Future prospective metabolomics studies of CRC that utilise more comprehensive metabolomics platforms are warranted. Additional experimental evidence is also needed to elucidate biological mechanisms underlying colorectal carcinogenesis in relation to metabolic dysregulation. Interventions aimed at altering circulating levels of key biomarkers may be beneficial for susceptible populations if their causal roles in CRC aetiology are proven.

## Supplementary information

BJC_Supplementary Files_Final Submission

## Data Availability

This research was conducted using the UK Biobank Resource under Application Number 55411. Data used in this project can be obtained directly from the UK Biobank by submitting a data request proposal. Full GWAS summary statistics for the eight metabolic biomarkers generated in this work are available in the GWAS catalogue (https://www.ebi.ac.uk/gwas/) under accession IDs GCST90454483 to GCST90454490.

## Code availability

The code is available from the corresponding author upon reasonable request.
