# Supplementary material for: Blood metabolic biomarkers and colorectal cancer risk: results from large prospective cohort and Mendelian randomisation analyses
Source: Br J Cancer. 2025 Apr 30;133(1):94–103. doi: 10.1038/s41416-025-02997-4 (PMC12238234; doi:10.1038/s41416-025-02997-4)
Supplement: Supplementary file 1 — BJC_Supplementary Files_Final Submission [file 41416_2025_2997_MOESM1_ESM.docx]

**Supplementary methods**

**Definitions of anatomical subsites of colorectal cancer**

Three CRC subsites were defined using anatomical locations (ICD-10 codes) as follows: proximal colon [cecum (C18.0), appendix (C18.1), ascending colon (C18.2), hepatic flexure (C18.3), transverse colon (C18.4), splenic flexure (C18.5)], distal colon [descending colon (C18.6) and sigmoid colon (C18.7)], rectum [rectosigmoid junction (C19) and rectum (C20)]. CRC cases with overlapping (C18.8) or unspecified (C18.9) lesions of colon were not included in the subsite analysis (*n*=66).

**Stratified analyses**

We also performed stratified analyses by sex and anatomic subsite of CRC (proximal colon, distal colon, rectum). Heterogeneity by sex was examined using likelihood ratio tests; heterogeneity across tumor subsites was evaluated by including an interaction term between each biomarker and the indicator of tumor site in the joint Cox model and testing its statistical significance using a global Wald test.^1^

**Genotyping, genetic imputation, and quality control**

Details about genotyping, quality control (QC), and genetic imputation procedures in the UK Biobank have been described previously.^2^ In brief, the genotypes for 488,377 UK Biobank participants were assayed using two genotyping arrays sharing a 95% marker content; 49,950 participants were genotyped using the UK BiLEVE Axiom Array by Affymetrix (UKBL; 807,411 markers) and 438,247 participants were genotyped using the UK Biobank Axiom Array (UKBB; 825,927 markers). Genotyping data were then imputed using either the Haplotype Reference Consortium data or the merged UK10K and 1000 Genomes Project phase 3 reference panels and were combined thereafter. The final imputed dataset contains >90 million genetic markers.

We followed the sample-level exclusion criteria outlined in a previous publication.^3^ Of the 230,420 individuals included in the observational analyses, we excluded those marked as outliers for heterozygosity, low call rates, and sex chromosome aneuploidy (*n*=321). European individuals were identified from the genotype data by projecting all of the UK Biobank samples on the first two major principal components of the four 1000 Genome populations (CEU, YRI, CHB, and JPT). We excluded individuals who did not fall into the CEU cluster (*n*=10,091). A kinship coefficient was estimated for each pair of samples using KING’s robust estimator in the UK Biobank dataset.^4^ Second-degree (or higher) related individuals (kinship coefficient ≥0.0442) were excluded (*n*=17,165). Lastly, we excluded participants self-reporting as non-European at study baseline (*n*=3,111). The final GWAS analytic dataset consisted of 199,732 participants (Supplementary Figure S1). Variants were removed from the analysis if they had a call rate <95%, a minor allele frequency (MAF) <1%, or a *P*-value <1×10^-6^ in the Hardy-Weinberg equilibrium test.

**Genome-wide association analysis**

We conducted genome-wide association studies (GWASs) to identify genetic variants associated with standardized levels of the following eight metabolic biomarkers that showed the most significant (*P*-value <0.01) and independent associations with CRC risk in the final backward stepwise Cox model: triglycerides to phosphoglycerides ratio, percentages of linoleic acid, saturated FA (SFA), and omega-6 FA to total FAs, concentrations of glycoprotein acetyls and 3-hydroxybutyrate, and percentages of cholesterol to total lipids in small LDL and triglycerides to total lipids in intermediate-density lipoprotein (IDL). Linear regression was used to estimate an additive (i.e., per-allele) effect on biomarker levels using the PLINK 2.0 (<https://www.cog-genomics.org/plink/2.0/>),^5^ adjusting for age, sex, genotyping arrays (UKBL, UKBB), and the first ten principal components for population structure. A *P*-value < 5×10^-8^ was considered genome-wide significant. Linkage disequilibrium (LD) clumping was performed to identify independently associated variants (*r*^2^ <1×10^-3^ in a 500kb window) using the PLINK 1.9 (<https://www.cog-genomics.org/plink/1.9/>).^5^

**Mendelian randomization analysis**

*Principles and underlying assumptions*

Mendelian randomization (MR), via employing genetic variants as instrument variables for the exposure, offers protections against biases such as residual confounding and reverse causation and herein strengthens the causal inference.^6^ Briefly, since genetic variants are randomly assorted from parents to offspring during gamete formation, they are not associated with the vast majority of variables that exert influences on the exposure and outcome after conception. Hence, by using genetic variants to proxy the exposure, MR mimics the design of randomized controlled trials and minimizes the influence of residual confounding. In addition, since the germline genotype is fixed at conception and cannot be modified by the natural history of disease, MR is also largely protected from reverse causation. Valid causal inference from MR analyses is contingent upon meeting the three underlying assumptions:^7^ (1) selected genetic instruments are strongly associated with the exposure (i.e., relevance), (2) there are no causes of genetic instruments that also influence the outcome (i.e., independence), and (3) genetic instruments do not affect the outcome independent of the exposure (i.e., exclusion restriction).

*Data sources and selection of the genetic instruments*

Independent and genome-wide significant (*P*-value <5×10^-8^) genetic variants from GWASs of select biomarkers were used as genetic instruments in MR analyses. For the outcome, we obtained summary statistics of the most recent consortium GWAS for CRC^8^ (78,473 cases and 107,143 controls of European ancestry) from the NHGRI-EBI GWAS Catalog^9^ (accession ID: GCST90255675) to maximize statistical power of the analysis. If an instrument was not available in the outcome GWAS dataset, we replaced it with a proxy variant in high LD (*r*^2^ >0.8) using LD Proxy (<https://ldlink.nih.gov/>).^10^ Palindromic variants with intermediate allele frequencies (MAF >0.42) were excluded. To minimize the potential horizontal pleotropic effect (i.e., variants influencing the outcome via other traits independent of the exposure), we searched each instrumental variables in the PhenoScanner GWAS database^11,12^ and removed variants significantly associated (*P*-value <5×10^-8^) with plausible confounders (e.g., educational attainment, cigarette smoking, alcohol consumption). In addition, we performed Radial MR analysis to identify and remove outlier pleotropic variants with the largest contribution to Cochran’s *Q* statistic using a stringent *P*-value threshold of 0.05.^13^ The remaining variants were then harmonized to ensure their exposure and outcome effect sizes were aligned to the same allele and included in the MR analysis.

We assessed the strength of genetic instruments using the *F*-statistic, which is a function of the proportion of variance in the phenotype explained by the genetic variants (*R*^2^), sample size (*N*), and number of instruments (*k*) according to the following formula:^14^

$$F=\frac{R^{2}(N-k-1)}{k(1-R^{2})}$$

*Statistical analyses*

In the two-sample MR analyses, odds ratios (ORs) and 95% confidence intervals (CIs) for the association of each metabolic biomarker with CRC risk were estimated primarily using the inverse-variance weighted (IVW) approach under a multiplicative random-effects model.^15^ We conducted four additional sensitivity analyses to evaluate the robust of primary findings based on different assumptions.^16^ First, we used the MR-Egger regression method to assess the presence of horizontal pleiotropy.^17^ An intercept term from the Egger regression that differs from zero (*P*-value <0.05) is indicative of directional horizontal pleiotropy, and the slope estimate provides a bias-corrected causal effect under the InSIDE assumption. Second, we employed a weighted-median approach which assumes that less than half of the variants used to construct the genetic instruments are invalid.^18^ Third, we used the MR-pleiotropy residual sum and outlier (MR-PRESSO) method to detect and correct for horizontal pleiotropic outliers.^19^ Fourth, we applied the MR-robust adjusted profiles score (MR-RAPS) method which models the systematic pleiotropy by a random effects model and provide robust estimates even in the presence of many weak instruments.^20^ All analyses were performed using the following R packages: ‘TwoSampleMR’, ‘MendelianRandomization’, ‘RadialMR’, ‘MRPRESSO’, and ‘mr.raps’.

**References**

1 Xue, X., Kim, M. Y., Gaudet, M. M., Park, Y., Heo, M., Hollenbeck, A. R. *et al.* A comparison of the polytomous logistic regression and joint cox proportional hazards models for evaluating multiple disease subtypes in prospective cohort studies. *Cancer Epidemiol Biomarkers Prev* **22**, 275-285 (2013).

2 Bycroft, C., Freeman, C., Petkova, D., Band, G., Elliott, L. T., Sharp, K. *et al.* The UK Biobank resource with deep phenotyping and genomic data. *Nature* **562**, 203-209 (2018).

3 Jia, G., Lu, Y., Wen, W., Long, J., Liu, Y., Tao, R. *et al.* Evaluating the Utility of Polygenic Risk Scores in Identifying High-Risk Individuals for Eight Common Cancers. *JNCI Cancer Spectr* **4**, pkaa021 (2020).

4 Manichaikul, A., Mychaleckyj, J. C., Rich, S. S., Daly, K., Sale, M. & Chen, W. M. Robust relationship inference in genome-wide association studies. *Bioinformatics* **26**, 2867-2873 (2010).

5 Chang, C. C., Chow, C. C., Tellier, L. C., Vattikuti, S., Purcell, S. M. & Lee, J. J. Second-generation PLINK: rising to the challenge of larger and richer datasets. *Gigascience* **4**, 7 (2015).

6 Sanderson, E., Glymour, M. M., Holmes, M. V., Kang, H., Morrison, J., Munafo, M. R. *et al.* Mendelian randomization. *Nat Rev Methods Primers* **2** (2022).

7 Davies, N. M., Holmes, M. V. & Davey Smith, G. Reading Mendelian randomisation studies: a guide, glossary, and checklist for clinicians. *BMJ* **362**, k601 (2018).

8 Fernandez-Rozadilla, C., Timofeeva, M., Chen, Z., Law, P., Thomas, M., Schmit, S. *et al.* Deciphering colorectal cancer genetics through multi-omic analysis of 100,204 cases and 154,587 controls of European and east Asian ancestries. *Nat Genet* **55**, 89-99 (2023).

9 Sollis, E., Mosaku, A., Abid, A., Buniello, A., Cerezo, M., Gil, L. *et al.* The NHGRI-EBI GWAS Catalog: knowledgebase and deposition resource. *Nucleic Acids Res* **51**, D977-D985 (2023).

10 Machiela, M. J. & Chanock, S. J. LDlink: a web-based application for exploring population-specific haplotype structure and linking correlated alleles of possible functional variants. *Bioinformatics* **31**, 3555-3557 (2015).

11 Staley, J. R., Blackshaw, J., Kamat, M. A., Ellis, S., Surendran, P., Sun, B. B. *et al.* PhenoScanner: a database of human genotype-phenotype associations. *Bioinformatics* **32**, 3207-3209 (2016).

12 Kamat, M. A., Blackshaw, J. A., Young, R., Surendran, P., Burgess, S., Danesh, J. *et al.* PhenoScanner V2: an expanded tool for searching human genotype-phenotype associations. *Bioinformatics* **35**, 4851-4853 (2019).

13 Bowden, J., Spiller, W., Del Greco, M. F., Sheehan, N., Thompson, J., Minelli, C. *et al.* Improving the visualization, interpretation and analysis of two-sample summary data Mendelian randomization via the Radial plot and Radial regression. *Int J Epidemiol* **47**, 1264-1278 (2018).

14 Burgess, S., Thompson, S. G. & Collaboration, C. C. G. Avoiding bias from weak instruments in Mendelian randomization studies. *Int J Epidemiol* **40**, 755-764 (2011).

15 Burgess, S., Butterworth, A. & Thompson, S. G. Mendelian randomization analysis with multiple genetic variants using summarized data. *Genet Epidemiol* **37**, 658-665 (2013).

16 Burgess, S., Davey Smith, G., Davies, N. M., Dudbridge, F., Gill, D., Glymour, M. M. *et al.* Guidelines for performing Mendelian randomization investigations: update for summer 2023. *Wellcome Open Res* **4**, 186 (2019).

17 Bowden, J., Davey Smith, G. & Burgess, S. Mendelian randomization with invalid instruments: effect estimation and bias detection through Egger regression. *Int J Epidemiol* **44**, 512-525 (2015).

18 Bowden, J., Davey Smith, G., Haycock, P. C. & Burgess, S. Consistent Estimation in Mendelian Randomization with Some Invalid Instruments Using a Weighted Median Estimator. *Genet Epidemiol* **40**, 304-314 (2016).

19 Verbanck, M., Chen, C. Y., Neale, B. & Do, R. Detection of widespread horizontal pleiotropy in causal relationships inferred from Mendelian randomization between complex traits and diseases. *Nat Genet* **50**, 693-698 (2018).

20 Zhao, Q., Wang, J., Hemani, G., Bowden, J. & Small, D. S. Statistical inference in two-sample summary-data Mendelian randomization using robust adjusted profile score. *The Annals of Statistics* **48**, 1742-1769, 1728 (2020).

**Supplementary Table S1.** 249 metabolic biomarkers included in the current analysis, the UK Biobank cohort

| **Metabolic biomarker** | **Abbreviation** | **Unit of measurement** |
| --- | --- | --- |
| **Cholesterol** |  |  |
| Total Cholesterol | Total-C | mmol/L |
| Total Cholesterol Minus HDL-C | Total-C_HDL-C | mmol/L |
| Remnant Cholesterol (Non-HDL, Non-LDL -Cholesterol) | Remnant-C | mmol/L |
| VLDL Cholesterol | VLDL-C | mmol/L |
| Clinical LDL Cholesterol | LDL-C-clinical | mmol/L |
| LDL Cholesterol | LDL-C | mmol/L |
| HDL Cholesterol | HDL-C | mmol/L |
| **Triglycerides** |  |  |
| Total Triglycerides | Total-TG | mmol/L |
| Triglycerides in VLDL | VLDL-TG | mmol/L |
| Triglycerides in LDL | LDL-TG | mmol/L |
| Triglycerides in HDL | HDL-TG | mmol/L |
| Triglycerides to Phosphoglycerides ratio | TG_PG_ratio | (ratio) |
| **Phospholipids** |  |  |
| Total Phospholipids in Lipoprotein Particles | Total-PL | mmol/L |
| Phospholipids in VLDL | VLDL-PL | mmol/L |
| Phospholipids in LDL | LDL-PL | mmol/L |
| Phospholipids in HDL | HDL-PL | mmol/L |
| **Cholesterol esters** |  |  |
| Total Esterified Cholesterol | EstC | mmol/L |
| Cholesteryl Esters in VLDL | VLDL-CE | mmol/L |
| Cholesteryl Esters in LDL | LDL-CE | mmol/L |
| Cholesteryl Esters in HDL | HDL-CE | mmol/L |
| **Free cholesterol** |  |  |
| Total Free Cholesterol | FC | mmol/L |
| Free Cholesterol in VLDL | VLDL-FC | mmol/L |
| Free Cholesterol in LDL | LDL-FC | mmol/L |
| Free Cholesterol in HDL | HDL-FC | mmol/L |
| **Total lipids** |  |  |
| Total Lipids in Lipoprotein Particles | Total-L | mmol/L |
| Total Lipids in VLDL | VLDL-L | mmol/L |
| Total Lipids in LDL | LDL-L | mmol/L |
| Total Lipids in HDL | HDL-L | mmol/L |
| **Lipoprotein particle concentrations** |  |  |
| Total Concentration of Lipoprotein Particles | Total-P | mmol/L |
| Concentration of VLDL Particles | VLDL-P | mmol/L |
| Concentration of LDL Particles | LDL-P | mmol/L |
| Concentration of HDL Particles | HDL-P | mmol/L |
| **Lipoprotein particle sizes** |  |  |
| Average Diameter for VLDL Particles | VLDL-D | nm |
| Average Diameter for LDL Particles | LDL-D | nm |
| Average Diameter for HDL Particles | HDL-D | nm |
| **Other lipids** |  |  |
| Phosphoglycerides | PG | mmol/L |
| Total Cholines | Cholines | mmol/L |
| Phosphatidylcholines | PC | mmol/L |
| Sphingomyelins | SM | mmol/L |
| **Apolipoproteins** |  |  |
| Apolipoprotein B | ApoB | g/L |
| Apolipoprotein A1 | ApoA1 | g/L |
| Apolipoprotein B to Apolipoprotein A1 ratio | ApoB_ApoA1_ratio | (ratio) |
| **Fatty acids** |  |  |
| Total Fatty Acids | Total-FA | mmol/L |
| Degree of Unsaturation | UnSat | degree |
| Omega-3 Fatty Acids | FAw3 | mmol/L |
| Omega-6 Fatty Acids | FAw6 | mmol/L |
| Polyunsaturated Fatty Acids | PUFA | mmol/L |
| Monounsaturated Fatty Acids | MUFA | mmol/L |
| Saturated Fatty Acids | SFA | mmol/L |
| Linoleic Acid | LA | mmol/L |
| Docosahexaenoic Acid | DHA | mmol/L |
| Omega-3 Fatty Acids to Total Fatty Acids percentage | FAw3_FA_pct | (%) |
| Omega-6 Fatty Acids to Total Fatty Acids percentage | FAw6_FA_pct | (%) |
| Polyunsaturated Fatty Acids to Total Fatty Acids percentage | PUFA_FA_pct | (%) |
| Monounsaturated Fatty Acids to Total Fatty Acids percentage | MUFA_FA_pct | (%) |
| Saturated Fatty Acids to Total Fatty Acids percentage | SFA_FA_pct | (%) |
| Linoleic Acid to Total Fatty Acids percentage | LA_FA_pct | (%) |
| Docosahexaenoic Acid to Total Fatty Acids percentage | DHA_FA_pct | (%) |
| Polyunsaturated Fatty Acids to Monounsaturated Fatty Acids ratio | PUFA_MUFA_ratio | (ratio) |
| Omega-6 Fatty Acids to Omega-3 Fatty Acids ratio | FAw3_FAw6_ratio | (ratio) |
| **Amino acids** |  |  |
| Alanine | Ala | mmol/L |
| Glutamine | Gln | mmol/L |
| Glycine | Gly | mmol/L |
| Histidine | His | mmol/L |
| Total Concentration of Branched-Chain Amino Acids (Leucine + Isoleucine + Valine) | BCAA | mmol/L |
| Isoleucine | Ile | mmol/L |
| Leucine | Leu | mmol/L |
| Valine | Val | mmol/L |
| Phenylalanine | Phe | mmol/L |
| Tyrosine | Tyr | mmol/L |
| **Glycolysis related biomarkers** |  |  |
| Glucose | Glc | mmol/L |
| Lactate | Lac | mmol/L |
| Pyruvate | Pyr | mmol/L |
| Citrate | Cit | mmol/L |
| **Ketone bodies** |  |  |
| 3-Hydroxybutyrate | bOHBut | mmol/L |
| Acetate | AceOH | mmol/L |
| Acetoacetate | AcAce | mmol/L |
| Acetone | AceCH3 | mmol/L |
| **Fluid balance** |  |  |
| Creatinine | Crea | mmol/L |
| Albumin | Alb | g/L |
| **Inflammation** |  |  |
| Glycoprotein Acetyls | Gp | mmol/L |
| **Lipoprotein lipid concentrations in 14 subclasses** |  |  |
| ***Chylomicrons and Extremely Large VLDL*** |  |  |
| Concentration of Chylomicrons and Extremely Large VLDL Particles | XXL-VLDL-P | mmol/L |
| Total Lipids in Chylomicrons and Extremely Large VLDL | XXL-VLDL-L | mmol/L |
| Phospholipids in Chylomicrons and Extremely Large VLDL | XXL-VLDL-PL | mmol/L |
| Cholesterol in Chylomicrons and Extremely Large VLDL | XXL-VLDL-C | mmol/L |
| Cholesteryl Esters in Chylomicrons and Extremely Large VLDL | XXL-VLDL-CE | mmol/L |
| Free Cholesterol in Chylomicrons and Extremely Large VLDL | XXL-VLDL-FC | mmol/L |
| Triglycerides in Chylomicrons and Extremely Large VLDL | XXL-VLDL-TG | mmol/L |
| ***Very Large VLDL*** |  |  |
| Concentration of Very Large VLDL Particles | XL-VLDL-P | mmol/L |
| Total Lipids in Very Large VLDL | XL-VLDL-L | mmol/L |
| Phospholipids in Very Large VLDL | XL-VLDL-PL | mmol/L |
| Cholesterol in Very Large VLDL | XL-VLDL-C | mmol/L |
| Cholesteryl Esters in Very Large VLDL | XL-VLDL-CE | mmol/L |
| Free Cholesterol in Very Large VLDL | XL-VLDL-FC | mmol/L |
| Triglycerides in Very Large VLDL | XL-VLDL-TG | mmol/L |
| ***Large VLDL*** |  |  |
| Concentration of Large VLDL Particles | L-VLDL-P | mmol/L |
| Total Lipids in Large VLDL | L-VLDL-L | mmol/L |
| Phospholipids in Large VLDL | L-VLDL-PL | mmol/L |
| Cholesterol in Large VLDL | L-VLDL-C | mmol/L |
| Cholesteryl Esters in Large VLDL | L-VLDL-CE | mmol/L |
| Free Cholesterol in Large VLDL | L-VLDL-FC | mmol/L |
| Triglycerides in Large VLDL | L-VLDL-TG | mmol/L |
| ***Medium VLDL*** |  |  |
| Concentration of Medium VLDL Particles | M-VLDL-P | mmol/L |
| Total Lipids in Medium VLDL | M-VLDL-L | mmol/L |
| Phospholipids in Medium VLDL | M-VLDL-PL | mmol/L |
| Cholesterol in Medium VLDL | MVLDL-C | mmol/L |
| Cholesteryl Esters in Medium VLDL | M-VLDL-CE | mmol/L |
| Free Cholesterol in Medium VLDL | M-VLDL-FC | mmol/L |
| Triglycerides in Medium VLDL | M-VLDL-TG | mmol/L |
| ***Small VLDL*** |  |  |
| Concentration of Small VLDL Particles | S-VLDL-P | mmol/L |
| Total Lipids in Small VLDL | S-VLDL-L | mmol/L |
| Phospholipids in Small VLDL | S-VLDL-PL | mmol/L |
| Cholesterol in Small VLDL | S-VLDL-C | mmol/L |
| Cholesteryl Esters in Small VLDL | S-VLDL-CE | mmol/L |
| Free Cholesterol in Small VLDL | S-VLDL-FC | mmol/L |
| Triglycerides in Small VLDL | S-VLDL-TG | mmol/L |
| ***Very Small VLDL*** |  |  |
| Concentration of Very Small VLDL Particles | XS-VLDL-P | mmol/L |
| Total Lipids in Very Small VLDL | XS-VLDL-L | mmol/L |
| Phospholipids in Very Small VLDL | XS-VLDL-PL | mmol/L |
| Cholesterol in Very Small VLDL | XS-VLDL-C | mmol/L |
| Cholesteryl Esters in Very Small VLDL | XS-VLDL-CE | mmol/L |
| Free Cholesterol in Very Small VLDL | XS-VLDL-FC | mmol/L |
| Triglycerides in Very Small VLDL | XS-VLDL-TG | mmol/L |
| ***IDL*** |  |  |
| Concentration of IDL Particles | IDL-P | mmol/L |
| Total Lipids in IDL | IDL-L | mmol/L |
| Phospholipids in IDL | IDL-PL | mmol/L |
| Cholesterol in IDL | IDL-C | mmol/L |
| Cholesteryl Esters in IDL | IDL-CE | mmol/L |
| Free Cholesterol in IDL | IDL-FC | mmol/L |
| Triglycerides in IDL | IDL-TG | mmol/L |
| ***Large LDL*** |  |  |
| Concentration of Large LDL Particles | L-LDL-P | mmol/L |
| Total Lipids in Large LDL | L-LDL-L | mmol/L |
| Phospholipids in Large LDL | L-LDL-PL | mmol/L |
| Cholesterol in Large LDL | L-LDL-C | mmol/L |
| Cholesteryl Esters in Large LDL | L-LDL-CE | mmol/L |
| Free Cholesterol in Large LDL | L-LDL-FC | mmol/L |
| Triglycerides in Large LDL | L-LDL-TG | mmol/L |
| ***Medium LDL*** |  |  |
| Concentration of Medium LDL Particles | M-LDL-P | mmol/L |
| Total Lipids in Medium LDL | M-LDL-L | mmol/L |
| Phospholipids in Medium LDL | M-LDL-PL | mmol/L |
| Cholesterol in Medium LDL | M-LDL-C | mmol/L |
| Cholesteryl Esters in Medium LDL | M-LDL-CE | mmol/L |
| Free Cholesterol in Medium LDL | M-LDL-FC | mmol/L |
| Triglycerides in Medium LDL | M-LDL-TG | mmol/L |
| ***Small LDL*** |  |  |
| Concentration of Small LDL Particles | S-LDL-P | mmol/L |
| Total Lipids in Small LDL | S-LDL-L | mmol/L |
| Phospholipids in Small LDL | S-LDL-PL | mmol/L |
| Cholesterol in Small LDL | S-LDL-C | mmol/L |
| Cholesteryl Esters in Small LDL | S-LDL-CE | mmol/L |
| Free Cholesterol in Small LDL | S-LDL-FC | mmol/L |
| Triglycerides in Small LDL | S-LDL-TG | mmol/L |
| ***Very Large HDL*** |  |  |
| Concentration of Very Large HDL Particles | XL-HDL-P | mmol/L |
| Total Lipids in Very Large HDL | XL-HDL-L | mmol/L |
| Phospholipids in Very Large HDL | XL-HDL-PL | mmol/L |
| Cholesterol in Very Large HDL | XL-HDL-C | mmol/L |
| Cholesteryl Esters in Very Large HDL | XL-HDL-CE | mmol/L |
| Free Cholesterol in Very Large HDL | XL-HDL-FC | mmol/L |
| Triglycerides in Very Large HDL | XL-HDL-TG | mmol/L |
| ***Large HDL*** |  |  |
| Concentration of Large HDL Particles | L-HDL-P | mmol/L |
| Total Lipids in Large HDL | L-HDL-L | mmol/L |
| Phospholipids in Large HDL | L-HDL-PL | mmol/L |
| Cholesterol in Large HDL | L-HDL-C | mmol/L |
| Cholesteryl Esters in Large HDL | L-HDL-CE | mmol/L |
| Free Cholesterol in Large HDL | L-HDL-FC | mmol/L |
| Triglycerides in Large HDL | L-HDL-TG | mmol/L |
| ***Medium HDL*** |  |  |
| Concentration of Medium HDL Particles | M-HDL-P | mmol/L |
| Total Lipids in Medium HDL | M-HDL-L | mmol/L |
| Phospholipids in Medium HDL | M-HDL-PL | mmol/L |
| Cholesterol in Medium HDL | M-HDL-C | mmol/L |
| Cholesteryl Esters in Medium HDL | M-HDL-CE | mmol/L |
| Free Cholesterol in Medium HDL | M-HDL-FC | mmol/L |
| Triglycerides in Medium HDL | M-HDL-TG | mmol/L |
| ***Small HDL*** |  |  |
| Concentration of Small HDL Particles | S-HDL-P | mmol/L |
| Total Lipids in Small HDL | S-HDL-L | mmol/L |
| Phospholipids in Small HDL | S-HDL-PL | mmol/L |
| Cholesterol in Small HDL | S-HDL-C | mmol/L |
| Cholesteryl Esters in Small HDL | S-HDL-CE | mmol/L |
| Free Cholesterol in Small HDL | S-HDL-FC | mmol/L |
| Triglycerides in Small HDL | S-HDL-TG | mmol/L |
| **Relative lipoprotein lipid concentrations in 14 subclasses** |  |  |
| ***Chylomicrons and Extremely Large VLDL*** |  |  |
| Phospholipids to Total Lipids in Chylomicrons and Extremely Large VLDL percentage | XXL-VLDL-PL_pct | (%) |
| Cholesterol to Total Lipids in Chylomicrons and Extremely Large VLDL percentage | XXL-VLDL-C_pct | (%) |
| Cholesteryl Esters to Total Lipids in Chylomicrons and Extremely Large VLDL percentage | XXL-VLDL-CE_pct | (%) |
| Free Cholesterol to Total Lipids in Chylomicrons and Extremely Large VLDL percentage | XXL-VLDL-FC_pct | (%) |
| Triglycerides to Total Lipids in Chylomicrons and Extremely Large VLDL percentage | XXL-VLDL-TG_pct | (%) |
| ***Very Large VLDL*** |  |  |
| Phospholipids to Total Lipids in Very Large VLDL percentage | XL-VLDL-PL_pct | (%) |
| Cholesterol to Total Lipids in Very Large VLDL percentage | XL-VLDL-C_pct | (%) |
| Cholesteryl Esters to Total Lipids in Very Large VLDL percentage | XL-VLDL-CE_pct | (%) |
| Free Cholesterol to Total Lipids in Very Large VLDL percentage | XL-VLDL-FC_pct | (%) |
| Triglycerides to Total Lipids in Very Large VLDL percentage | XL-VLDL-TG_pct | (%) |
| ***Large VLDL*** |  |  |
| Phospholipids to Total Lipids in Large VLDL percentage | L-VLDL-PL_pct | (%) |
| Cholesterol to Total Lipids in Large VLDL percentage | L-VLDL-C_pct | (%) |
| Cholesteryl Esters to Total Lipids in Large VLDL percentage | L-VLDL-CE_pct | (%) |
| Free Cholesterol to Total Lipids in Large VLDL percentage | L-VLDL-FC_pct | (%) |
| Triglycerides to Total Lipids in Large VLDL percentage | L-VLDL-TG_pct | (%) |
| ***Medium VLDL*** |  |  |
| Phospholipids to Total Lipids in Medium VLDL percentage | M-VLDL-PL_pct | (%) |
| Cholesterol to Total Lipids in Medium VLDL percentage | M-VLDL-C_pct | (%) |
| Cholesteryl Esters to Total Lipids in Medium VLDL percentage | MVLDL-CE_pct | (%) |
| Free Cholesterol to Total Lipids in Medium VLDL percentage | M-VLDL-FC_pct | (%) |
| Triglycerides to Total Lipids in Medium VLDL percentage | M-VLDL-TG_pct | (%) |
| ***Small VLDL*** |  |  |
| Phospholipids to Total Lipids in Small VLDL percentage | S-VLDL-PL_pct | (%) |
| Cholesterol to Total Lipids in Small VLDL percentage | S-VLDL-C_pct | (%) |
| Cholesteryl Esters to Total Lipids in Small VLDL percentage | S-VLDL-CE_pct | (%) |
| Free Cholesterol to Total Lipids in Small VLDL percentage | S-VLDL-FC_pct | (%) |
| Triglycerides to Total Lipids in Small VLDL percentage | S-VLDL-TG_pct | (%) |
| ***Very Small VLDL*** |  |  |
| Phospholipids to Total Lipids in Very Small VLDL percentage | XS-VLDL-PL_pct | (%) |
| Cholesterol to Total Lipids in Very Small VLDL percentage | XS-VLDL-C_pct | (%) |
| Cholesteryl Esters to Total Lipids in Very Small VLDL percentage | XS-VLDL-CE_pct | (%) |
| Free Cholesterol to Total Lipids in Very Small VLDL percentage | XS-VLDL-FC_pct | (%) |
| Triglycerides to Total Lipids in Very Small VLDL percentage | XS-VLDL-TG_pct | (%) |
| ***IDL*** |  |  |
| Phospholipids to Total Lipids in IDL percentage | IDL-PL_pct | (%) |
| Cholesterol to Total Lipids in IDL percentage | IDL-C_pct | (%) |
| Cholesteryl Esters to Total Lipids in IDL percentage | IDL-CE_pct | (%) |
| Free Cholesterol to Total Lipids in IDL percentage | IDL-FC_pct | (%) |
| Triglycerides to Total Lipids in IDL percentage | IDL-TG_pct | (%) |
| ***Large LDL*** |  |  |
| Phospholipids to Total Lipids in Large LDL percentage | L-LDL-PL_pct | (%) |
| Cholesterol to Total Lipids in Large LDL percentage | L-LDL-C_pct | (%) |
| Cholesteryl Esters to Total Lipids in Large LDL percentage | L-LDL-CE_pct | (%) |
| Free Cholesterol to Total Lipids in Large LDL percentage | L-LDL-FC_pct | (%) |
| Triglycerides to Total Lipids in Large LDL percentage | L-LDL-TG_pct | (%) |
| ***Medium LDL*** |  |  |
| Phospholipids to Total Lipids in Medium LDL percentage | M-LDL-PL_pct | (%) |
| Cholesterol to Total Lipids in Medium LDL percentage | M-LDL-C_pct | (%) |
| Cholesteryl Esters to Total Lipids in Medium LDL percentage | M-LDL-CE_pct | (%) |
| Free Cholesterol to Total Lipids in Medium LDL percentage | M-LDL-FC_pct | (%) |
| Triglycerides to Total Lipids in Medium LDL percentage | M-LDL-TG_pct | (%) |
| ***Small LDL*** |  |  |
| Phospholipids to Total Lipids in Small LDL percentage | S-LDL-PL_pct | (%) |
| Cholesterol to Total Lipids in Small LDL percentage | S-LDL-C_pct | (%) |
| Cholesteryl Esters to Total Lipids in Small LDL percentage | S-LDL-CE_pct | (%) |
| Free Cholesterol to Total Lipids in Small LDL percentage | S-LDL-FC_pct | (%) |
| Triglycerides to Total Lipids in Small LDL percentage | S-LDL-TG_pct | (%) |
| ***Very Large HDL*** |  |  |
| Phospholipids to Total Lipids in Very Large HDL percentage | XL-HDL-PL_pct | (%) |
| Cholesterol to Total Lipids in Very Large HDL percentage | XL-HDL-C_pct | (%) |
| Cholesteryl Esters to Total Lipids in Very Large HDL percentage | XL-HDL-CE_pct | (%) |
| Free Cholesterol to Total Lipids in Very Large HDL percentage | XL-HDL-FC_pct | (%) |
| Triglycerides to Total Lipids in Very Large HDL percentage | XL-HDL-TG_pct | (%) |
| ***Large HDL*** |  |  |
| Phospholipids to Total Lipids in Large HDL percentage | L-HDL-PL_pct | (%) |
| Cholesterol to Total Lipids in Large HDL percentage | L-HDL-C_pct | (%) |
| Cholesteryl Esters to Total Lipids in Large HDL percentage | L-HDL-CE_pct | (%) |
| Free Cholesterol to Total Lipids in Large HDL percentage | L-HDL-FC_pct | (%) |
| Triglycerides to Total Lipids in Large HDL percentage | L-HDL-TG_pct | (%) |
| ***Medium HDL*** |  |  |
| Phospholipids to Total Lipids in Medium HDL percentage | M-HDL-PL_pct | (%) |
| Cholesterol to Total Lipids in Medium HDL percentage | M-HDL-C_pct | (%) |
| Cholesteryl Esters to Total Lipids in Medium HDL percentage | M-HDL-CE_pct | (%) |
| Free Cholesterol to Total Lipids in Medium HDL percentage | M-HDL-FC_pct | (%) |
| Triglycerides to Total Lipids in Medium HDL percentage | M-HDL-TG_pct | (%) |
| ***Small HDL*** |  |  |
| Phospholipids to Total Lipids in Small HDL percentage | S-HDL-PL_pct | (%) |
| Cholesterol to Total Lipids in Small HDL percentage | S-HDL-C_pct | (%) |
| Cholesteryl Esters to Total Lipids in Small HDL percentage | S-HDL-CE_pct | (%) |
| Free Cholesterol to Total Lipids in Small HDL percentage | S-HDL-FC_pct | (%) |
| Triglycerides to Total Lipids in Small HDL percentage | S-HDL-TG_pct | (%) |

**Supplementary Table S2:** Total variance of 249 metabolic biomarkers explained by the first 50 principal components in the current analysis, the UK Biobank cohort

| **Principal component** | **% Variance explained** | **% Cumulative variance explained** |
| --- | --- | --- |
| 1 | 33.83 | 33.8 |
| 2 | 26.71 | 60.5 |
| 3 | 11.40 | 71.9 |
| 4 | 5.55 | 77.5 |
| 5 | 2.97 | 80.5 |
| 6 | 2.30 | 82.8 |
| 7 | 1.80 | 84.6 |
| 8 | 1.61 | 86.2 |
| 9 | 1.29 | 87.5 |
| 10 | 1.23 | 88.7 |
| 11 | 0.88 | 89.6 |
| 12 | 0.80 | 90.4 |
| 13 | 0.70 | 91.1 |
| 14 | 0.67 | 91.7 |
| 15 | 0.58 | 92.3 |
| 16 | 0.53 | 92.9 |
| 17 | 0.50 | 93.4 |
| 18 | 0.43 | 93.8 |
| 19 | 0.40 | 94.2 |
| 20 | 0.37 | 94.5 |
| 21 | 0.35 | 94.9 |
| 22 | 0.32 | 95.2 |
| 23 | 0.30 | 95.5 |
| 24 | 0.29 | 95.8 |
| 25 | 0.29 | 96.1 |
| 26 | 0.27 | 96.4 |
| 27 | 0.27 | 96.6 |
| 28 | 0.25 | 96.9 |
| 29 | 0.23 | 97.1 |
| 30 | 0.22 | 97.3 |
| 31 | 0.20 | 97.5 |
| 32 | 0.19 | 97.7 |
| 33 | 0.17 | 97.9 |
| 34 | 0.16 | 98.0 |
| 35 | 0.16 | 98.2 |
| 36 | 0.15 | 98.4 |
| 37 | 0.14 | 98.5 |
| 38 | 0.12 | 98.6 |
| 39 | 0.11 | 98.7 |
| 40 | 0.10 | 98.8 |
| 41 | 0.09 | 98.9 |
| 42 | 0.08 | 99.0 |
| 43 | 0.08 | 99.1 |
| 44 | 0.07 | 99.2 |
| 45 | 0.06 | 99.2 |
| 46 | 0.06 | 99.3 |
| 47 | 0.05 | 99.3 |
| 48 | 0.05 | 99.4 |
| 49 | 0.05 | 99.4 |
| 50 | 0.05 | 99.5 |

**Supplementary Table S3.** Associations of 249 metabolic biomarkers with CRC risk, the UK Biobank cohort^a^

| **Metabolic biomarker** | **Base Model^b^** | | | |  | **Full Model^c^** | | | |
| --- | --- | --- | --- | --- | --- | --- | --- | --- | --- |
|  | **HR** | **Lower 95% CI** | **Upper 95% CI** | ***P*-value** |  | **HR** | **Lower 95% CI** | **Upper 95% CI** | ***P*-value** |
| **Cholesterol** |  |  |  |  |  |  |  |  |  |
| Total Cholesterol | 0.99 | 0.95 | 1.04 | 0.80 |  | 0.99 | 0.95 | 1.04 | 0.65 |
| Total Cholesterol Minus HDL-C | 1.01 | 0.96 | 1.05 | 0.80 |  | 1.00 | 0.96 | 1.05 | 0.93 |
| Remnant Cholesterol (Non-HDL, Non-LDL -Cholesterol) | 1.01 | 0.97 | 1.06 | 0.61 |  | 1.01 | 0.96 | 1.05 | 0.74 |
| VLDL Cholesterol | 1.04 | 0.99 | 1.08 | 0.10 |  | 1.03 | 0.99 | 1.08 | 0.16 |
| Clinical LDL Cholesterol | 0.99 | 0.95 | 1.03 | 0.66 |  | 0.99 | 0.95 | 1.03 | 0.59 |
| LDL Cholesterol | 1.00 | 0.96 | 1.04 | 1.00 |  | 1.00 | 0.95 | 1.04 | 0.89 |
| HDL Cholesterol | 0.97 | 0.92 | 1.01 | 0.15 |  | 0.96 | 0.91 | 1.01 | 0.10 |
| **Triglycerides** |  |  |  |  |  |  |  |  |  |
| Total Triglycerides | **1.07** | **1.03** | **1.12** | **2.95E-04** |  | 1.06 | 1.02 | 1.11 | 1.81E-03 |
| Triglycerides in VLDL | **1.08** | **1.03** | **1.12** | **2.43E-04** |  | 1.07 | 1.03 | 1.11 | 1.33E-03 |
| Triglycerides in LDL | **1.07** | **1.03** | **1.11** | **9.00E-04** |  | 1.06 | 1.02 | 1.10 | 5.61E-03 |
| Triglycerides in HDL | 1.05 | 1.01 | 1.09 | 0.03 |  | 1.04 | 0.99 | 1.08 | 0.09 |
| Triglycerides to Phosphoglycerides (ratio) | **1.09** | **1.04** | **1.13** | **7.19E-05** |  | **1.08** | **1.04** | **1.13** | **2.43E-04** |
| **Phospholipids** |  |  |  |  |  |  |  |  |  |
| Total Phospholipids in Lipoprotein Particles | 1.02 | 0.98 | 1.07 | 0.39 |  | 1.01 | 0.96 | 1.06 | 0.67 |
| Phospholipids in VLDL | 1.06 | 1.02 | 1.10 | 6.14E-03 |  | 1.05 | 1.01 | 1.10 | 0.02 |
| Phospholipids in LDL | 1.00 | 0.96 | 1.05 | 0.92 |  | 1.00 | 0.96 | 1.04 | 0.95 |
| Phospholipids in HDL | 0.99 | 0.95 | 1.04 | 0.74 |  | 0.98 | 0.94 | 1.03 | 0.45 |
| **Cholesterol esters** |  |  |  |  |  |  |  |  |  |
| Total Esterified Cholesterol | 0.99 | 0.95 | 1.04 | 0.66 |  | 0.99 | 0.94 | 1.03 | 0.52 |
| Cholesteryl Esters in VLDL | 1.02 | 0.98 | 1.07 | 0.31 |  | 1.02 | 0.98 | 1.06 | 0.39 |
| Cholesteryl Esters in LDL | 1.01 | 0.96 | 1.05 | 0.76 |  | 1.00 | 0.96 | 1.05 | 0.90 |
| Cholesteryl Esters in HDL | 0.96 | 0.92 | 1.01 | 0.09 |  | 0.96 | 0.91 | 1.00 | 0.06 |
| **Free cholesterol** |  |  |  |  |  |  |  |  |  |
| Total Free Cholesterol | 1.00 | 0.96 | 1.05 | 0.84 |  | 1.00 | 0.96 | 1.05 | 0.99 |
| Free Cholesterol in VLDL | 1.05 | 1.01 | 1.10 | 0.02 |  | 1.04 | 1.00 | 1.09 | 0.04 |
| Free Cholesterol in LDL | 0.98 | 0.94 | 1.03 | 0.41 |  | 0.98 | 0.94 | 1.03 | 0.39 |
| Free Cholesterol in HDL | 0.99 | 0.94 | 1.03 | 0.57 |  | 0.98 | 0.93 | 1.03 | 0.43 |
| **Total lipids** |  |  |  |  |  |  |  |  |  |
| Total Lipids in Lipoprotein Particles | 1.03 | 0.99 | 1.08 | 0.16 |  | 1.02 | 0.98 | 1.07 | 0.31 |
| Total Lipids in VLDL | 1.07 | 1.02 | 1.11 | 1.87E-03 |  | 1.06 | 1.02 | 1.10 | 6.73E-03 |
| Total Lipids in LDL | 1.01 | 0.96 | 1.05 | 0.79 |  | 1.00 | 0.96 | 1.05 | 0.93 |
| Total Lipids in HDL | 0.98 | 0.94 | 1.03 | 0.48 |  | 0.97 | 0.93 | 1.02 | 0.29 |
| **Lipoprotein particle concentrations** |  |  |  |  |  |  |  |  |  |
| Total Concentration of Lipoprotein Particles | 0.98 | 0.94 | 1.03 | 0.40 |  | 0.97 | 0.93 | 1.01 | 0.18 |
| Concentration of VLDL Particles | 1.05 | 1.00 | 1.09 | 0.03 |  | 1.04 | 1.00 | 1.08 | 0.06 |
| Concentration of LDL Particles | 1.02 | 0.97 | 1.06 | 0.48 |  | 1.01 | 0.97 | 1.06 | 0.53 |
| Concentration of HDL Particles | 0.98 | 0.94 | 1.02 | 0.32 |  | 0.97 | 0.92 | 1.01 | 0.13 |
| **Lipoprotein particle sizes** |  |  |  |  |  |  |  |  |  |
| Average Diameter for VLDL Particles | 1.07 | 1.03 | 1.12 | 1.69E-03 |  | 1.06 | 1.02 | 1.11 | 5.27E-03 |
| Average Diameter for LDL Particles | 0.97 | 0.93 | 1.01 | 0.12 |  | 0.97 | 0.93 | 1.01 | 0.18 |
| Average Diameter for HDL Particles | 0.97 | 0.93 | 1.02 | 0.28 |  | 0.98 | 0.93 | 1.03 | 0.38 |
| **Other lipids** |  |  |  |  |  |  |  |  |  |
| Phosphoglycerides | 1.01 | 0.97 | 1.06 | 0.63 |  | 1.00 | 0.96 | 1.05 | 0.99 |
| Total Cholines | 1.00 | 0.96 | 0.96 | 0.91 |  | 0.99 | 0.95 | 1.04 | 0.75 |
| Phosphatidylcholines | 1.00 | 0.95 | 1.04 | 0.94 |  | 0.99 | 0.94 | 1.03 | 0.59 |
| Sphingomyelins | 1.01 | 0.96 | 1.05 | 0.83 |  | 1.00 | 0.95 | 1.05 | 0.95 |
| **Apolipoproteins** |  |  |  |  |  |  |  |  |  |
| Apolipoprotein B | 1.02 | 0.97 | 1.06 | 0.47 |  | 1.01 | 0.97 | 1.06 | 0.55 |
| Apolipoprotein A1 | 0.98 | 0.94 | 1.03 | 0.43 |  | 0.97 | 0.93 | 1.02 | 0.21 |
| Apolipoprotein B to Apolipoprotein A1 (ratio) | 1.03 | 0.98 | 1.07 | 0.22 |  | 1.03 | 0.99 | 1.08 | 0.18 |
| **Fatty acids** |  |  |  |  |  |  |  |  |  |
| Total Fatty Acids | 1.05 | 1.01 | 1.10 | 0.01 |  | 1.04 | 1.00 | 1.09 | 0.05 |
| Degree of Unsaturation | **0.92** | **0.88** | **0.96** | **4.13E-05** |  | **0.93** | **0.89** | **0.97** | **6.74E-04** |
| Omega-3 Fatty Acids | 1.00 | 0.96 | 1.04 | 1.00 |  | 1.00 | 0.96 | 1.05 | 0.92 |
| Omega-6 Fatty Acids | 1.00 | 0.96 | 1.05 | 0.94 |  | 1.00 | 0.95 | 1.04 | 0.92 |
| Polyunsaturated Fatty Acids | 1.00 | 0.96 | 1.05 | 0.95 |  | 1.00 | 0.96 | 1.04 | 0.96 |
| Monounsaturated Fatty Acids | **1.07** | **1.03** | **1.11** | **8.08E-04** |  | 1.06 | 1.01 | 1.10 | 7.36E-03 |
| Saturated Fatty Acids | **1.07** | **1.03** | **1.11** | **5.61E-04** |  | 1.06 | 1.02 | 1.10 | 5.78E-03 |
| Linoleic Acid | 0.99 | 0.95 | 1.03 | 0.57 |  | 0.99 | 0.94 | 1.03 | 0.57 |
| Docosahexaenoic Acid | 0.97 | 0.93 | 1.01 | 0.12 |  | 0.97 | 0.93 | 1.02 | 0.20 |
| Omega-3 Fatty Acids to Total Fatty Acids (%) | 0.96 | 0.92 | 1.00 | 0.07 |  | 0.97 | 0.93 | 1.01 | 0.17 |
| Omega-6 Fatty Acids to Total Fatty Acids (%) | **0.91** | **0.87** | **0.95** | **2.87E-06** |  | **0.92** | **0.89** | **0.96** | **1.00E-04** |
| Polyunsaturated Fatty Acids to Total Fatty Acids (%) | **0.90** | **0.86** | **0.94** | **1.61E-07** |  | **0.91** | **0.88** | **0.95** | **1.71E-05** |
| Monounsaturated Fatty Acids to Total Fatty Acids (%) | **1.09** | **1.04** | **1.13** | **6.19E-06** |  | 1.07 | 1.03 | 1.12 | 1.24E-03 |
| Saturated Fatty Acids to Total Fatty Acids (%) | **1.10** | **1.06** | **1.14** | **1.51E-06** |  | **1.08** | **1.04** | **1.13** | **7.49E-05** |
| Linoleic Acid to Total Fatty Acids (%) | **0.90** | **0.86** | **0.94** | **5.17E-07** |  | **0.91** | **0.87** | **0.95** | **2.55E-05** |
| Docosahexaenoic Acid to Total Fatty Acids (%) | 0.94 | 0.90 | 0.98 | 1.87E-03 |  | 0.94 | 0.90 | 0.99 | 0.01 |
| Polyunsaturated Fatty Acids to Monounsaturated Fatty Acids (ratio) | **0.91** | **0.87** | **0.95** | **1.42E-05** |  | **0.92** | **0.88** | **0.96** | **3.85E-04** |
| Omega-6 Fatty Acids to Omega-3 Fatty Acids (ratio) | 1.02 | 0.98 | 1.06 | 0.40 |  | 1.02 | 0.98 | 1.06 | 0.44 |
| **Amino acids** |  |  |  |  |  |  |  |  |  |
| Alanine | 1.00 | 0.96 | 1.04 | 0.97 |  | 1.00 | 0.96 | 1.04 | 0.89 |
| Glutamine | 0.95 | 0.91 | 0.99 | 0.01 |  | 0.96 | 0.92 | 1.00 | 0.03 |
| Glycine | 0.96 | 0.91 | 1.00 | 0.05 |  | 0.97 | 0.92 | 1.01 | 0.12 |
| Histidine | 0.96 | 0.92 | 1.02 | 0.17 |  | 0.97 | 0.92 | 1.02 | 0.18 |
| Total Concentration of Branched-Chain Amino Acids (Leucine + Isoleucine + Valine) | 1.00 | 0.96 | 1.05 | 0.85 |  | 1.00 | 0.96 | 1.04 | 0.98 |
| Isoleucine | 1.01 | 0.97 | 1.05 | 0.57 |  | 1.01 | 0.97 | 1.05 | 0.68 |
| Leucine | 1.00 | 0.95 | 1.04 | 0.86 |  | 0.99 | 0.95 | 1.03 | 0.68 |
| Valine | 1.01 | 0.96 | 1.05 | 0.80 |  | 1.00 | 0.96 | 1.04 | 0.97 |
| Phenylalanine | 1.00 | 0.96 | 1.04 | 0.91 |  | 1.00 | 0.96 | 1.04 | 0.84 |
| Tyrosine | 0.99 | 0.95 | 1.04 | 0.76 |  | 0.99 | 0.95 | 1.03 | 0.61 |
| **Glycolysis related biomarkers** |  |  |  |  |  |  |  |  |  |
| Glucose | 1.05 | 1.01 | 1.09 | 4.65E-03 |  | 1.05 | 1.01 | 1.08 | 6.53E-03 |
| Lactate | 1.00 | 0.96 | 1.04 | 0.89 |  | 1.00 | 0.96 | 1.04 | 0.96 |
| Pyruvate | 1.01 | 0.97 | 1.06 | 0.51 |  | 1.01 | 0.97 | 1.05 | 0.62 |
| Citrate | 1.01 | 0.97 | 1.06 | 0.58 |  | 1.02 | 0.98 | 1.07 | 0.31 |
| **Ketone bodies** |  |  |  |  |  |  |  |  |  |
| 3-Hydroxybutyrate | **1.06** | **1.03** | **1.10** | **7.82E-05** |  | **1.06** | **1.03** | **1.09** | **2.16E-04** |
| Acetate | 0.99 | 0.95 | 1.04 | 0.77 |  | 0.99 | 0.95 | 1.04 | 0.82 |
| Acetoacetate | 1.05 | 1.02 | 1.08 | 1.11E-03 |  | 1.04 | 1.01 | 1.08 | 4.24E-03 |
| Acetone | 1.04 | 1.01 | 1.08 | 8.79E-03 |  | 1.04 | 1.01 | 1.07 | 0.02 |
| **Fluid balance** |  |  |  |  |  |  |  |  |  |
| Creatinine | 0.99 | 0.95 | 1.04 | 0.75 |  | 0.99 | 0.94 | 1.04 | 0.64 |
| Albumin | 0.95 | 0.91 | 0.99 | 0.03 |  | 0.96 | 0.92 | 1.00 | 0.04 |
| **Inflammation** |  |  |  |  |  |  |  |  |  |
| Glycoprotein Acetyls | **1.09** | **1.05** | **1.13** | **3.18E-05** |  | **1.08** | **1.03** | **1.12** | **2.95E-04** |
| **Lipoprotein lipid concentrations in 14 subclasses** |  |  |  |  |  |  |  |  |  |
| ***Chylomicrons and Extremely Large VLDL*** |  |  |  |  |  |  |  |  |  |
| Concentration of Chylomicrons and Extremely Large VLDL Particles | **1.08** | **1.04** | **1.12** | **8.15E-05** |  | **1.07** | **1.03** | **1.11** | **5.82E-04** |
| Total Lipids in Chylomicrons and Extremely Large VLDL | **1.08** | **1.04** | **1.12** | **7.82E-05** |  | **1.07** | **1.03** | **1.11** | **5.61E-04** |
| Phospholipids in Chylomicrons and Extremely Large VLDL | **1.08** | **1.04** | **1.12** | **1.39E-04** |  | **1.07** | **1.03** | **1.11** | **8.68E-04** |
| Cholesterol in Chylomicrons and Extremely Large VLDL | **1.07** | **1.03** | **1.12** | **6.04E-04** |  | 1.06 | 1.02 | 1.11 | 2.88E-03 |
| Cholesteryl Esters in Chylomicrons and Extremely Large VLDL | 1.07 | 1.03 | 1.11 | 1.37E-03 |  | 1.06 | 1.02 | 1.10 | 5.61E-03 |
| Free Cholesterol in Chylomicrons and Extremely Large VLDL | **1.08** | **1.03** | **1.12** | **2.52E-04** |  | 1.07 | 1.03 | 1.11 | 1.42E-03 |
| Triglycerides in Chylomicrons and Extremely Large VLDL | **1.08** | **1.04** | **1.12** | **4.13E-05** |  | **1.07** | **1.03** | **1.11** | **3.44E-04** |
| ***Very Large VLDL*** |  |  |  |  |  |  |  |  |  |
| Concentration of Very Large VLDL Particles | **1.07** | **1.03** | **1.12** | **3.85E-04** |  | 1.07 | 1.02 | 1.11 | 1.94E-03 |
| Total Lipids in Very Large VLDL | **1.08** | **1.03** | **1.12** | **2.73E-04** |  | 1.07 | 1.03 | 1.11 | 1.47E-03 |
| Phospholipids in Very Large VLDL | **1.07** | **1.03** | **1.12** | **4.48E-04** |  | 1.07 | 1.02 | 1.11 | 2.00E-03 |
| Cholesterol in Very Large VLDL | 1.06 | 1.02 | 1.10 | 4.95E-03 |  | 1.05 | 1.01 | 1.10 | 0.01 |
| Cholesteryl Esters in Very Large VLDL | 1.05 | 1.00 | 1.09 | 0.03 |  | 1.04 | 1.00 | 1.09 | 0.06 |
| Free Cholesterol in Very Large VLDL | **1.07** | **1.03** | **1.11** | **7.52E-04** |  | 1.06 | 1.02 | 1.11 | 2.98E-03 |
| Triglycerides in Very Large VLDL | **1.08** | **1.04** | **1.12** | **1.09E-04** |  | **1.07** | **1.03** | **1.11** | **7.52E-04** |
| ***Large VLDL*** |  |  |  |  |  |  |  |  |  |
| Concentration of Large VLDL Particles | 1.07 | 1.03 | 1.11 | 1.20E-03 |  | 1.06 | 1.02 | 1.10 | 4.51E-03 |
| Total Lipids in Large VLDL | 1.07 | 1.03 | 1.11 | 1.15E-03 |  | 1.06 | 1.02 | 1.10 | 4.24E-03 |
| Phospholipids in Large VLDL | 1.07 | 1.03 | 1.11 | 1.37E-03 |  | 1.06 | 1.02 | 1.10 | 5.11E-03 |
| Cholesterol in Large VLDL | 1.05 | 1.01 | 1.10 | 0.01 |  | 1.05 | 1.01 | 1.09 | 0.03 |
| Cholesteryl Esters in Large VLDL | 1.04 | 1.00 | 1.09 | 0.06 |  | 1.04 | 0.99 | 1.08 | 0.09 |
| Free Cholesterol in Large VLDL | 1.06 | 1.02 | 1.11 | 2.45E-03 |  | 1.06 | 1.01 | 1.10 | 7.81E-03 |
| Triglycerides in Large VLDL | **1.07** | **1.03** | **1.12** | **4.16E-04** |  | 1.06 | 1.02 | 1.11 | 1.87E-03 |
| ***Medium VLDL*** |  |  |  |  |  |  |  |  |  |
| Concentration of Medium VLDL Particles | 1.03 | 0.99 | 1.08 | 0.14 |  | 1.03 | 0.99 | 1.07 | 0.20 |
| Total Lipids in Medium VLDL | 1.04 | 1.00 | 1.09 | 0.05 |  | 1.04 | 1.00 | 1.08 | 0.08 |
| Phospholipids in Medium VLDL | 1.03 | 0.99 | 1.08 | 0.16 |  | 1.03 | 0.98 | 1.07 | 0.22 |
| Cholesterol in Medium VLDL | 1.00 | 0.95 | 1.04 | 0.92 |  | 1.00 | 0.95 | 1.04 | 0.94 |
| Cholesteryl Esters in Medium VLDL | 0.98 | 0.93 | 1.02 | 0.35 |  | 0.98 | 0.94 | 1.03 | 0.42 |
| Free Cholesterol in Medium VLDL | 1.02 | 0.98 | 1.07 | 0.32 |  | 1.02 | 0.98 | 1.06 | 0.38 |
| Triglycerides in Medium VLDL | 1.06 | 1.02 | 1.10 | 3.08E-03 |  | 1.05 | 1.01 | 1.10 | 9.88E-03 |
| ***Small VLDL*** |  |  |  |  |  |  |  |  |  |
| Concentration of Small VLDL Particles | 1.05 | 1.00 | 1.09 | 0.03 |  | 1.04 | 1.00 | 1.08 | 0.06 |
| Total Lipids in Small VLDL | 1.04 | 1.00 | 1.09 | 0.04 |  | 1.04 | 1.00 | 1.08 | 0.08 |
| Phospholipids in Small VLDL | 1.03 | 0.99 | 1.07 | 0.19 |  | 1.02 | 0.98 | 1.07 | 0.25 |
| Cholesterol in Small VLDL | 1.02 | 0.98 | 1.06 | 0.39 |  | 1.02 | 0.97 | 1.06 | 0.47 |
| Cholesteryl Esters in Small VLDL | 1.02 | 0.98 | 1.07 | 0.33 |  | 1.02 | 0.98 | 1.06 | 0.42 |
| Free Cholesterol in Small VLDL | 1.01 | 0.97 | 1.06 | 0.51 |  | 1.01 | 0.97 | 1.06 | 0.58 |
| Triglycerides in Small VLDL | 1.06 | 1.02 | 1.11 | 2.70E-03 |  | 1.06 | 1.01 | 1.10 | 8.79E-03 |
| ***Very Small VLDL*** |  |  |  |  |  |  |  |  |  |
| Concentration of Very Small VLDL Particles | 1.03 | 0.99 | 1.08 | 0.19 |  | 1.02 | 0.98 | 1.07 | 0.27 |
| Total Lipids in Very Small VLDL | 1.03 | 0.99 | 1.08 | 0.16 |  | 1.03 | 0.98 | 1.07 | 0.24 |
| Phospholipids in Very Small VLDL | 1.04 | 1.00 | 1.09 | 0.07 |  | 1.04 | 0.99 | 1.08 | 0.11 |
| Cholesterol in Very Small VLDL | 1.00 | 0.96 | 1.05 | 0.87 |  | 1.00 | 0.96 | 1.05 | 0.95 |
| Cholesteryl Esters in Very Small VLDL | 0.99 | 0.95 | 1.04 | 0.80 |  | 0.99 | 0.95 | 1.04 | 0.75 |
| Free Cholesterol in Very Small VLDL | 1.02 | 0.98 | 1.07 | 0.27 |  | 1.02 | 0.98 | 1.07 | 0.36 |
| Triglycerides in Very Small VLDL | 1.06 | 1.02 | 1.10 | 2.45E-03 |  | 1.05 | 1.01 | 1.10 | 0.01 |
| ***IDL*** |  |  |  |  |  |  |  |  |  |
| Concentration of IDL Particles | 0.99 | 0.95 | 1.04 | 0.75 |  | 0.99 | 0.95 | 1.04 | 0.66 |
| Total Lipids in IDL | 0.99 | 0.94 | 1.04 | 0.65 |  | 0.99 | 0.94 | 1.03 | 0.54 |
| Phospholipids in IDL | 1.00 | 0.95 | 1.04 | 0.86 |  | 0.99 | 0.95 | 1.04 | 0.79 |
| Cholesterol in IDL | 0.98 | 0.93 | 1.02 | 0.34 |  | 0.97 | 0.93 | 1.02 | 0.28 |
| Cholesteryl Esters in IDL | 0.98 | 0.93 | 1.02 | 0.35 |  | 0.97 | 0.93 | 1.02 | 0.28 |
| Free Cholesterol in IDL | 0.98 | 0.93 | 1.02 | 0.32 |  | 0.98 | 0.93 | 1.02 | 0.31 |
| Triglycerides in IDL | 1.06 | 1.02 | 1.10 | 3.39E-03 |  | 1.05 | 1.01 | 1.09 | 0.02 |
| ***Large LDL*** |  |  |  |  |  |  |  |  |  |
| Concentration of Large LDL Particles | 1.01 | 0.97 | 1.06 | 0.55 |  | 1.01 | 0.97 | 1.06 | 0.57 |
| Total Lipids in Large LDL | 1.00 | 0.96 | 1.04 | 0.96 |  | 1.00 | 0.95 | 1.04 | 0.84 |
| Phospholipids in Large LDL | 0.99 | 0.95 | 1.03 | 0.64 |  | 0.99 | 0.94 | 1.03 | 0.54 |
| Cholesterol in Large LDL | 0.99 | 0.95 | 1.04 | 0.81 |  | 0.99 | 0.95 | 1.04 | 0.73 |
| Cholesteryl Esters in Large LDL | 1.00 | 0.96 | 1.05 | 0.98 |  | 1.00 | 0.96 | 1.04 | 0.92 |
| Free Cholesterol in Large LDL | 0.98 | 0.93 | 1.02 | 0.33 |  | 0.98 | 0.93 | 1.02 | 0.31 |
| Triglycerides in Large LDL | 1.06 | 1.02 | 1.11 | 1.69E-03 |  | 1.05 | 1.01 | 1.10 | 9.88E-03 |
| ***Medium LDL*** |  |  |  |  |  |  |  |  |  |
| Concentration of Medium LDL Particles | 1.01 | 0.97 | 1.06 | 0.56 |  | 1.01 | 0.97 | 1.05 | 0.68 |
| Total Lipids in Medium LDL | 1.01 | 0.97 | 1.06 | 0.53 |  | 1.01 | 0.97 | 1.05 | 0.71 |
| Phospholipids in Medium LDL | 1.01 | 0.97 | 1.06 | 0.58 |  | 1.01 | 0.97 | 1.05 | 0.74 |
| Cholesterol in Medium LDL | 1.01 | 0.97 | 1.05 | 0.72 |  | 1.00 | 0.96 | 1.05 | 0.88 |
| Cholesteryl Esters in Medium LDL | 1.02 | 0.97 | 1.06 | 0.48 |  | 1.01 | 0.97 | 1.05 | 0.67 |
| Free Cholesterol in Medium LDL | 0.99 | 0.95 | 1.03 | 0.56 |  | 0.99 | 0.94 | 1.03 | 0.53 |
| Triglycerides in Medium LDL | **1.07** | **1.03** | **1.11** | **6.26E-04** |  | 1.06 | 1.02 | 1.10 | 3.98E-03 |
| ***Small LDL*** |  |  |  |  |  |  |  |  |  |
| Concentration of Small LDL Particles | 1.03 | 0.99 | 1.07 | 0.18 |  | 1.03 | 0.98 | 1.07 | 0.22 |
| Total Lipids in Small LDL | 1.03 | 0.98 | 1.07 | 0.24 |  | 1.02 | 0.98 | 1.07 | 0.32 |
| Phospholipids in Small LDL | 1.03 | 0.99 | 1.08 | 0.14 |  | 1.03 | 0.99 | 1.08 | 0.17 |
| Cholesterol in Small LDL | 1.01 | 0.97 | 1.06 | 0.55 |  | 1.01 | 0.97 | 1.05 | 0.65 |
| Cholesteryl Esters in Small LDL | 1.02 | 0.98 | 1.07 | 0.32 |  | 1.02 | 0.97 | 1.06 | 0.44 |
| Free Cholesterol in Small LDL | 0.99 | 0.95 | 1.03 | 0.62 |  | 0.99 | 0.95 | 1.03 | 0.66 |
| Triglycerides in Small LDL | **1.07** | **1.03** | **1.12** | **2.24E-04** |  | 1.06 | 1.02 | 1.11 | 1.37E-03 |
| ***Very Large HDL*** |  |  |  |  |  |  |  |  |  |
| Concentration of Very Large HDL Particles | 1.00 | 0.95 | 1.04 | 0.86 |  | 1.00 | 0.96 | 1.05 | 0.91 |
| Total Lipids in Very Large HDL | 0.99 | 0.95 | 1.04 | 0.75 |  | 1.00 | 0.95 | 1.05 | 1.00 |
| Phospholipids in Very Large HDL | 0.99 | 0.95 | 1.04 | 0.76 |  | 1.00 | 0.95 | 1.05 | 0.99 |
| Cholesterol in Very Large HDL | 0.99 | 0.94 | 1.03 | 0.56 |  | 0.99 | 0.95 | 1.04 | 0.81 |
| Cholesteryl Esters in Very Large HDL | 0.98 | 0.94 | 1.03 | 0.44 |  | 0.99 | 0.94 | 1.04 | 0.67 |
| Free Cholesterol in Very Large HDL | 1.00 | 0.96 | 1.05 | 0.90 |  | 1.01 | 0.97 | 1.06 | 0.62 |
| Triglycerides in Very Large HDL | 1.06 | 1.01 | 1.10 | 8.05E-03 |  | 1.05 | 1.01 | 1.09 | 0.02 |
| ***Large HDL*** |  |  |  |  |  |  |  |  |  |
| Concentration of Large HDL Particles | 0.97 | 0.92 | 1.02 | 0.23 |  | 0.97 | 0.93 | 1.02 | 0.28 |
| Total Lipids in Large HDL | 0.97 | 0.93 | 1.02 | 0.28 |  | 0.98 | 0.93 | 1.02 | 0.32 |
| Phospholipids in Large HDL | 0.98 | 0.93 | 1.03 | 0.38 |  | 0.98 | 0.93 | 1.03 | 0.38 |
| Cholesterol in Large HDL | 0.97 | 0.92 | 1.01 | 0.17 |  | 0.97 | 0.92 | 1.02 | 0.23 |
| Cholesteryl Esters in Large HDL | 0.96 | 0.92 | 1.01 | 0.13 |  | 0.97 | 0.92 | 1.02 | 0.19 |
| Free Cholesterol in Large HDL | 0.98 | 0.93 | 1.03 | 0.41 |  | 0.98 | 0.94 | 1.03 | 0.48 |
| Triglycerides in Large HDL | 1.02 | 0.98 | 1.07 | 0.31 |  | 1.02 | 0.97 | 1.06 | 0.48 |
| ***Medium HDL*** |  |  |  |  |  |  |  |  |  |
| Concentration of Medium HDL Particles | 0.48 | 0.94 | 1.02 | 0.36 |  | 0.97 | 0.92 | 1.01 | 0.16 |
| Total Lipids in Medium HDL | 0.99 | 0.94 | 1.03 | 0.54 |  | 0.97 | 0.93 | 1.02 | 0.25 |
| Phospholipids in Medium HDL | 1.00 | 0.95 | 1.04 | 0.85 |  | 0.98 | 0.94 | 1.03 | 0.44 |
| Cholesterol in Medium HDL | 0.97 | 0.93 | 1.02 | 0.19 |  | 0.96 | 0.91 | 1.01 | 0.08 |
| Cholesteryl Esters in Medium HDL | 0.97 | 0.92 | 1.01 | 0.15 |  | 0.96 | 0.91 | 1.00 | 0.06 |
| Free Cholesterol in Medium HDL | 0.98 | 0.94 | 1.03 | 0.48 |  | 0.97 | 0.93 | 1.02 | 0.25 |
| Triglycerides in Medium HDL | 1.04 | 1.00 | 1.09 | 0.04 |  | 1.03 | 0.99 | 1.07 | 0.16 |
| ***Small HDL*** |  |  |  |  |  |  |  |  |  |
| Concentration of Small HDL Particles | 0.99 | 0.95 | 1.03 | 0.58 |  | 0.98 | 0.94 | 1.02 | 0.24 |
| Total Lipids in Small HDL | 1.01 | 0.96 | 1.05 | 0.81 |  | 0.99 | 0.95 | 1.03 | 0.62 |
| Phospholipids in Small HDL | 1.01 | 0.97 | 1.05 | 0.62 |  | 0.99 | 0.95 | 1.04 | 0.77 |
| Cholesterol in Small HDL | 0.98 | 0.94 | 1.02 | 0.37 |  | 0.97 | 0.93 | 1.01 | 0.14 |
| Cholesteryl Esters in Small HDL | 0.97 | 0.93 | 1.01 | 0.21 |  | 0.96 | 0.92 | 1.00 | 0.07 |
| Free Cholesterol in Small HDL | 1.01 | 0.97 | 1.05 | 0.75 |  | 0.99 | 0.95 | 1.04 | 0.77 |
| Triglycerides in Small HDL | 1.06 | 1.02 | 1.10 | 5.11E-03 |  | 1.05 | 1.01 | 1.09 | 0.03 |
| **Relative lipoprotein lipid concentrations in 14 subclasses** |  |  |  |  |  |  |  |  |  |
| ***Chylomicrons and Extremely Large VLDL*** |  |  |  |  |  |  |  |  |  |
| Phospholipids to Total Lipids in Chylomicrons and Extremely Large VLDL (%) | 0.98 | 0.94 | 1.02 | 0.36 |  | 0.98 | 0.94 | 1.02 | 0.34 |
| Cholesterol to Total Lipids in Chylomicrons and Extremely Large VLDL (%) | 0.95 | 0.91 | 1.00 | 0.04 |  | 0.96 | 0.92 | 1.00 | 0.06 |
| Cholesteryl Esters to Total Lipids in Chylomicrons and Extremely Large VLDL (%) | 0.96 | 0.92 | 1.00 | 0.07 |  | 0.96 | 0.92 | 1.01 | 0.09 |
| Free Cholesterol to Total Lipids in Chylomicrons and Extremely Large VLDL (%) | 0.96 | 0.92 | 1.00 | 0.06 |  | 0.96 | 0.92 | 1.01 | 0.09 |
| Triglycerides to Total Lipids in Chylomicrons and Extremely Large VLDL (%) | 1.05 | 1.00 | 1.09 | 0.03 |  | 1.04 | 1.00 | 1.09 | 0.05 |
| ***Very Large VLDL*** |  |  |  |  |  |  |  |  |  |
| Phospholipids to Total Lipids in Very Large VLDL (%) | 1.02 | 0.97 | 1.06 | 0.48 |  | 1.02 | 0.97 | 1.07 | 0.45 |
| Cholesterol to Total Lipids in Very Large VLDL (%) | 0.95 | 0.91 | 1.00 | 0.07 |  | 0.97 | 0.92 | 1.01 | 0.16 |
| Cholesteryl Esters to Total Lipids in Very Large VLDL (%) | 0.95 | 0.90 | 1.00 | 0.04 |  | 0.96 | 0.91 | 1.01 | 0.10 |
| Free Cholesterol to Total Lipids in Very Large VLDL (%) | 0.98 | 0.94 | 1.03 | 0.48 |  | 0.99 | 0.95 | 1.04 | 0.79 |
| Triglycerides to Total Lipids in Very Large VLDL (%) | 1.04 | 0.99 | 1.09 | 0.11 |  | 1.03 | 0.98 | 1.08 | 0.25 |
| ***Large VLDL*** |  |  |  |  |  |  |  |  |  |
| Phospholipids to Total Lipids in Large VLDL (%) | 1.02 | 0.98 | 1.07 | 0.37 |  | 1.01 | 0.97 | 1.06 | 0.54 |
| Cholesterol to Total Lipids in Large VLDL (%) | 0.95 | 0.90 | 0.99 | 0.01 |  | 0.95 | 0.91 | 1.00 | 0.03 |
| Cholesteryl Esters to Total Lipids in Large VLDL (%) | 0.94 | 0.90 | 0.98 | 5.96E-03 |  | 0.95 | 0.90 | 0.99 | 0.02 |
| Free Cholesterol to Total Lipids in Large VLDL (%) | 0.98 | 0.94 | 1.02 | 0.33 |  | 0.98 | 0.94 | 1.02 | 0.33 |
| Triglycerides to Total Lipids in Large VLDL (%) | 1.03 | 0.99 | 1.08 | 0.14 |  | 1.03 | 0.99 | 1.07 | 0.18 |
| ***Medium VLDL*** |  |  |  |  |  |  |  |  |  |
| Phospholipids to Total Lipids in Medium VLDL (%) | 0.94 | 0.90 | 0.99 | 0.01 |  | 0.95 | 0.91 | 0.99 | 0.02 |
| Cholesterol to Total Lipids in Medium VLDL (%) | 0.93 | 0.89 | 0.97 | 1.28E-03 |  | 0.94 | 0.89 | 0.98 | 5.27E-03 |
| Cholesteryl Esters to Total Lipids in Medium VLDL (%) | **0.93** | **0.88** | **0.97** | **8.38E-04** |  | 0.93 | 0.89 | 0.98 | 3.85E-03 |
| Free Cholesterol to Total Lipids in Medium VLDL (%) | 0.94 | 0.90 | 0.98 | 5.61E-03 |  | 0.95 | 0.90 | 0.99 | 0.02 |
| Triglycerides to Total Lipids in Medium VLDL (%) | 1.08 | 1.03 | 1.13 | 1.75E-03 |  | 1.07 | 1.02 | 1.12 | 6.33E-03 |
| ***Small VLDL*** |  |  |  |  |  |  |  |  |  |
| Phospholipids to Total Lipids in Small VLDL (%) | 0.94 | 0.89 | 0.98 | 5.78E-03 |  | 0.94 | 0.90 | 0.99 | 0.02 |
| Cholesterol to Total Lipids in Small VLDL (%) | 0.94 | 0.90 | 0.98 | 4.95E-03 |  | 0.94 | 0.90 | 0.99 | 0.01 |
| Cholesteryl Esters to Total Lipids in Small VLDL (%) | 0.95 | 0.91 | 0.99 | 0.01 |  | 0.95 | 0.91 | 0.99 | 0.02 |
| Free Cholesterol to Total Lipids in Small VLDL (%) | 0.94 | 0.89 | 0.98 | 4.37E-03 |  | 0.94 | 0.90 | 0.99 | 0.01 |
| Triglycerides to Total Lipids in Small VLDL (%) | 1.07 | 1.02 | 1.12 | 4.37E-03 |  | 1.06 | 1.01 | 1.11 | 0.01 |
| ***Very Small VLDL*** |  |  |  |  |  |  |  |  |  |
| Phospholipids to Total Lipids in Very Small VLDL (%) | **1.08** | **1.03** | **1.12** | **6.99E-04** |  | **1.08** | **1.03** | **1.12** | **5.82E-04** |
| Cholesterol to Total Lipids in Very Small VLDL (%) | **0.92** | **0.88** | **0.96** | **3.71E-04** |  | 0.93 | 0.89 | 0.97 | 1.42E-03 |
| Cholesteryl Esters to Total Lipids in Very Small VLDL (%) | **0.92** | **0.88** | **0.96** | **2.33E-04** |  | **0.93** | **0.89** | **0.97** | **8.68E-04** |
| Free Cholesterol to Total Lipids in Very Small VLDL (%) | 0.96 | 0.92 | 1.00 | 0.07 |  | 0.97 | 0.93 | 1.01 | 0.15 |
| Triglycerides to Total Lipids in Very Small VLDL (%) | 1.08 | 1.03 | 1.12 | 1.42E-03 |  | 1.07 | 1.02 | 1.11 | 5.44E-03 |
| ***IDL*** |  |  |  |  |  |  |  |  |  |
| Phospholipids to Total Lipids in IDL (%) | 1.03 | 0.99 | 1.07 | 0.19 |  | 1.04 | 0.99 | 1.08 | 0.10 |
| Cholesterol to Total Lipids in IDL (%) | 0.93 | 0.90 | 0.97 | 1.15E-03 |  | 0.94 | 0.90 | 0.98 | 2.21E-03 |
| Cholesteryl Esters to Total Lipids in IDL (%) | 0.94 | 0.90 | 0.98 | 5.44E-03 |  | 0.94 | 0.90 | 0.98 | 5.78E-03 |
| Free Cholesterol to Total Lipids in IDL (%) | 0.95 | 0.92 | 0.99 | 0.018 |  | 0.96 | 0.92 | 1.00 | 0.05 |
| Triglycerides to Total Lipids in IDL (%) | **1.07** | **1.03** | **1.12** | **5.01E-04** |  | 1.07 | 1.02 | 1.11 | 2.00E-03 |
| ***Large LDL*** |  |  |  |  |  |  |  |  |  |
| Phospholipids to Total Lipids in Large LDL (%) | 0.96 | 0.92 | 1.00 | 0.07 |  | 0.96 | 0.92 | 1.00 | 0.08 |
| Cholesterol to Total Lipids in Large LDL (%) | 0.96 | 0.93 | 0.99 | 0.01 |  | 0.97 | 0.94 | 1.00 | 0.03 |
| Cholesteryl Esters to Total Lipids in Large LDL (%) | 1.01 | 0.96 | 1.05 | 0.77 |  | 1.01 | 0.97 | 1.06 | 0.68 |
| Free Cholesterol to Total Lipids in Large LDL (%) | **0.93** | **0.89** | **0.96** | **1.45E-04** |  | **0.93** | **0.90** | **0.97** | **7.52E-04** |
| Triglycerides to Total Lipids in Large LDL (%) | **1.06** | **1.03** | **1.10** | **1.28E-04** |  | 1.05 | 1.02 | 1.09 | 1.11E-03 |
| ***Medium LDL*** |  |  |  |  |  |  |  |  |  |
| Phospholipids to Total Lipids in Medium LDL (%) | 0.99 | 0.95 | 1.03 | 0.66 |  | 1.00 | 0.96 | 1.04 | 0.84 |
| Cholesterol to Total Lipids in Medium LDL (%) | **0.95** | **0.92** | **0.98** | **4.32E-04** |  | 0.95 | 0.93 | 0.98 | 1.52E-03 |
| Cholesteryl Esters to Total Lipids in Medium LDL (%) | 1.02 | 0.98 | 1.06 | 0.42 |  | 1.01 | 0.97 | 1.05 | 0.62 |
| Free Cholesterol to Total Lipids in Medium LDL (%) | 0.94 | 0.90 | 0.97 | 1.24E-03 |  | 0.95 | 0.91 | 0.98 | 6.73E-03 |
| Triglycerides to Total Lipids in Medium LDL (%) | **1.06** | **1.03** | **1.10** | **2.24E-04** |  | 1.06 | 1.02 | 1.09 | 1.33E-03 |
| ***Small LDL*** |  |  |  |  |  |  |  |  |  |
| Phospholipids to Total Lipids in Small LDL (%) | 1.02 | 0.98 | 1.06 | 0.36 |  | 1.03 | 0.99 | 1.07 | 0.21 |
| Cholesterol to Total Lipids in Small LDL (%) | **0.93** | **0.90** | **0.96** | **7.46E-06** |  | **0.93** | **0.90** | **0.96** | **1.19E-05** |
| Cholesteryl Esters to Total Lipids in Small LDL (%) | 0.99 | 0.95 | 1.03 | 0.62 |  | 0.98 | 0.94 | 1.02 | 0.39 |
| Free Cholesterol to Total Lipids in Small LDL (%) | **0.94** | **0.90** | **0.97** | **9.33E-04** |  | 0.95 | 0.91 | 0.98 | 4.65E-03 |
| Triglycerides to Total Lipids in Small LDL (%) | **1.07** | **1.03** | **1.11** | **3.31E-04** |  | 1.06 | 1.02 | 1.10 | 2.07E-03 |
| ***Very Large HDL*** |  |  |  |  |  |  |  |  |  |
| Phospholipids to Total Lipids in Very Large HDL (%) | 0.99 | 0.95 | 1.03 | 0.69 |  | 1.00 | 0.96 | 1.04 | 0.86 |
| Cholesterol to Total Lipids in Very Large HDL (%) | 0.98 | 0.94 | 1.02 | 0.41 |  | 0.98 | 0.94 | 1.02 | 0.41 |
| Cholesteryl Esters to Total Lipids in Very Large HDL (%) | 0.96 | 0.92 | 0.99 | 0.02 |  | 0.96 | 0.92 | 1.00 | 0.03 |
| Free Cholesterol to Total Lipids in Very Large HDL (%) | 1.02 | 0.99 | 1.07 | 0.21 |  | 1.02 | 0.98 | 1.06 | 0.28 |
| Triglycerides to Total Lipids in Very Large HDL (%) | 1.03 | 1.00 | 1.06 | 0.04 |  | 1.03 | 0.99 | 1.06 | 0.12 |
| ***Large HDL*** |  |  |  |  |  |  |  |  |  |
| Phospholipids to Total Lipids in Large HDL (%) | 1.06 | 1.02 | 1.11 | 4.80E-03 |  | 1.05 | 1.00 | 1.09 | 0.03 |
| Cholesterol to Total Lipids in Large HDL (%) | 0.95 | 0.91 | 0.98 | 5.96E-03 |  | 0.96 | 0.92 | 0.99 | 0.03 |
| Cholesteryl Esters to Total Lipids in Large HDL (%) | 0.94 | 0.90 | 0.97 | 1.04E-03 |  | 0.95 | 0.91 | 0.98 | 6.73E-03 |
| Free Cholesterol to Total Lipids in Large HDL (%) | 1.01 | 0.97 | 1.06 | 0.54 |  | 1.02 | 0.97 | 1.06 | 0.44 |
| Triglycerides to Total Lipids in Large HDL (%) | 1.04 | 1.00 | 1.07 | 0.04 |  | 1.03 | 1.00 | 1.07 | 0.08 |
| ***Medium HDL*** |  |  |  |  |  |  |  |  |  |
| Phospholipids to Total Lipids in Medium HDL (%) | **1.08** | **1.04** | **1.13** | **1.91E-04** |  | **1.08** | **1.03** | **1.12** | **6.50E-04** |
| Cholesterol to Total Lipids in Medium HDL (%) | 0.93 | 0.90 | 0.97 | 1.33E-03 |  | 0.94 | 0.90 | 0.98 | 3.98E-03 |
| Cholesteryl Esters to Total Lipids in Medium HDL (%) | **0.93** | **0.90** | **0.97** | **8.68E-04** |  | 0.94 | 0.90 | 0.98 | 3.28E-03 |
| Free Cholesterol to Total Lipids in Medium HDL (%) | 0.97 | 0.93 | 1.02 | 0.24 |  | 0.97 | 0.93 | 1.02 | 0.23 |
| Triglycerides to Total Lipids in Medium HDL (%) | 1.06 | 1.02 | 1.10 | 6.73E-03 |  | 1.05 | 1.01 | 1.09 | 0.02 |
| ***Small HDL*** |  |  |  |  |  |  |  |  |  |
| Phospholipids to Total Lipids in Small HDL (%) | 1.03 | 0.99 | 1.08 | 0.12 |  | 1.03 | 0.98 | 1.07 | 0.25 |
| Cholesterol to Total Lipids in Small HDL (%) | 0.94 | 0.90 | 0.98 | 2.00E-03 |  | 0.95 | 0.91 | 0.99 | 8.54E-03 |
| Cholesteryl Esters to Total Lipids in Small HDL (%) | 0.94 | 0.90 | 0.98 | 1.42E-03 |  | 0.94 | 0.91 | 0.98 | 5.11E-03 |
| Free Cholesterol to Total Lipids in Small HDL (%) | 1.01 | 0.96 | 1.05 | 0.82 |  | 1.01 | 0.97 | 1.06 | 0.59 |
| Triglycerides to Total Lipids in Small HDL (%) | 1.07 | 1.03 | 1.11 | 1.75E-03 |  | 1.06 | 1.02 | 1.11 | 4.51E-03 |

Abbreviations: CI, confidence interval; CRC, colorectal cancer; HDL, high-density lipoprotein; HR, hazard ratio; IDL, intermediate-density lipoprotein; LDL, low-density lipoprotein; SD, standard deviation; VLDL, very low-density lipoprotein.

^a^ Only results for biomarkers showing significant associations with CRC risk (*P*-value< 0.001) in the base Cox model were presented.

^b^ HRs and 95% CIs for 1-SD increase in biomarker levels were estimated using the base Cox model with the age as the time scale, adjusting for sex, race, educational attainment, fasting time, self-reported cholesterol-lowering medication use, and CRC family history and CRC screening history.

^c^ The full Cox model was additionally adjusted for alcohol drinking, cigarette smoking, fruits/vegetables intake, and processed/red meat intake.

Associations that were statistically significant [*P*-value <0.001 (0.05/50)] were shown in bold.

**Supplementary Table S4.** Associations of ten selected biomarkers with colorectal cancer risk after mutual adjustment in the final backward stepwise Cox model, the UK Biobank cohort^a^

| **Metabolic biomarker** | **HR (95% CI)** | ***P*-value** |
| --- | --- | --- |
| Triglycerides to Phosphoglycerides (ratio) | 1.17 (1.05-1.30) | 3.73×10^-3^ |
| Linoleic Acid to Total Fatty Acids (%) | 0.86 (0.77-0.96) | 8.79×10^-3^ |
| Saturated Fatty Acids to Total Fatty Acids (%) | 1.12 (1.05-1.20) | 1.24×10^-3^ |
| Omega-6 Fatty Acids to Total Fatty Acids (%) | 1.25 (1.06-1.48) | 8.79×10^-3^ |
| Glycoprotein Acetyls (mmol/L) | 1.09 (1.03-1.15) | 1.63×10^-3^ |
| 3-Hydroxybutyrate (mmol/L) | 1.07 (1.04-1.11) | 8.50×10^-5^ |
| Concentration of Chylomicrons and Extremely Large VLDL Particles (mmol/L) | 2.37 (1.09-5.15) | 0.03 |
| Triglycerides in Chylomicrons and Extremely Large VLDL (mmol/L) | 0.58 (0.37-0.91) | 0.02 |
| Cholesterol to Total Lipids in Small LDL (%) | 0.91 (0.86-0.96) | 6.04×10^-4^ |
| Triglycerides to Total Lipids in IDL (%) | 0.88 (0.80-0.97) | 6.73×10^-3^ |

Abbreviations: CI, confidence interval; CRC, colorectal cancer; HR, hazard ratio; IDL, intermediate-density lipoprotein; LDL, low-density lipoprotein; SD, standard deviation; VLDL, very low-density lipoprotein.

^a^ Biomarkers presented in the table were selected by the final backward stepwise Cox model (*P*-value <0.10). Association statistics were obtained from the final backward stepwise Cox model, adjusting for baseline age, sex, race, educational attainment, fasting time, self-reported cholesterol-lowering medication use, and CRC family history and CRC screening history.

**Supplementary Table S5.** Associations of ten selected metabolic biomarkers with colorectal cancer risk among non-users of cholesterol-lowering medications, the UK Biobank cohort^a^

| **Metabolic biomarker** | **HR^b^ (95% CI)** | ***P*-value** | **HR^c^ (95% CI)** | ***P*-value** |
| --- | --- | --- | --- | --- |
| Triglycerides to Phosphoglycerides (ratio) | 1.09 (1.04-1.15) | 5.02×10^-4^ | 1.09 (1.04-1.15) | 8.69×10^-4^ |
| Linoleic Acid to Total Fatty Acids (%) | 0.90 (0.86-0.95) | 4.70×10^-5^ | 0.92 (0.87-0.97) | 1.33×10^-3^ |
| Saturated Fatty Acids to Total Fatty Acids (%) | 1.11 (1.06-1.16) | 5.90×10^-6^ | 1.09 (1.04-1.14) | 3.44×10^-4^ |
| Omega-6 Fatty Acids to Total Fatty Acids (%) | 0.90 (0.86-0.95) | 3.96×10^-5^ | 0.92 (0.88-0.97) | 9.00×10^-4^ |
| Glycoprotein Acetyls (mmol/L) | 1.10 (1.05-1.15) | 7.82×10^-5^ | 1.09 (1.04-1.14) | 4.00×10^-4^ |
| 3-Hydroxybutyrate (mmol/L) | 1.07 (1.04-1.11) | 1.19×10^-5^ | 1.07 (1.03-1.10) | 5.58×10^-5^ |
| Concentration of Chylomicrons and Extremely Large VLDL Particles (mmol/L) | 1.09 (1.04-1.14) | 3.44×10^-4^ | 1.08 (1.03-1.13) | 1.63×10^-3^ |
| Triglycerides in Chylomicrons and Extremely Large VLDL (mmol/L) | 1.09 (1.04-1.14) | 1.92×10^-4^ | 1.08 (1.03-1.13) | 1.04×10^-3^ |
| Cholesterol to Total Lipids in Small LDL (%) | 0.91 (0.87-0.95) | 5.35×10^-5^ | 0.91 (0.87-0.95) | 6.07×10^-5^ |
| Triglycerides to Total Lipids in IDL (%) | 1.09 (1.04-1.15) | 4.32×10^-4^ | 1.09 (1.03-1.14) | 1.47×10^-3^ |

Abbreviations: CI, confidence interval; CRC, colorectal cancer; HR, hazard ratio; IDL, intermediate-density lipoprotein; LDL, low-density lipoprotein; SD, standard deviation; VLDL, very low-density lipoprotein.

^a^ Biomarkers presented in the table were selected by the final backward stepwise Cox model (*P*-value <0.10).

^b^ HRs and 95% CIs for 1-SD increase in biomarker levels were estimated using the base Cox model with the age as the time scale, adjusting for sex, race, educational attainment, fasting time, and CRC family history and CRC screening history.

^c^ The full Cox model was additionally adjusted for alcohol drinking, cigarette smoking, fruits/vegetables intake, and processed/red meat intake.

**Supplementary Table S6.** Associations of ten selected metabolic biomarkers with colorectal cancer risk after exclusion of biomarker outliers, the UK Biobank cohort^a^

| **Metabolic biomarker** | **# Outliers^b^ (%)** | **HR^c^ (95% CI)** | ***P*-value** | **HR^d^ (95% CI)** | ***P*-value** |
| --- | --- | --- | --- | --- | --- |
| Triglycerides to Phosphoglycerides (ratio) | 7 (0) | 1.09 (1.04-1.13) | 7.19×10^-5^ | 1.08 (1.04-1.13) | 2.33×10^-4^ |
| Linoleic Acid to Total Fatty Acids (%) | 2 (0) | 0.90 (0.86-0.94) | 5.18×10^-7^ | 0.91 (0.87-0.95) | 2.67×10^-5^ |
| Saturated Fatty Acids to Total Fatty Acids (%) | 34 (0.01) | 1.10 (1.06-1.14) | 1.24×10^-6^ | 1.08 (1.04-1.13) | 6.34×10^-5^ |
| Omega-6 Fatty Acids to Total Fatty Acids (%) | 8 (0) | 0.91 (0.87-0.95) | 2.74×10^-6^ | 0.92 (0.89-0.96) | 9.62×10^-5^ |
| Glycoprotein Acetyls (mmol/L) | 46 (0.02) | 1.09 (1.05-1.13) | 2.56×10^-5^ | 1.08 (1.04-1.12) | 2.43×10^-4^ |
| 3-Hydroxybutyrate (mmol/L) | 7,640 (3.32) | 1.05 (1.00-1.09) | 0.04 | 1.04 (1.00-1.08) | 0.06 |
| Concentration of Chylomicrons and Extremely Large VLDL Particles (mmol/L) | 415 (0.18) | 1.07 (1.03-1.11) | 5.82×10^-4^ | 1.06 (1.02-1.10) | 3.18×10^-3^ |
| Triglycerides in Chylomicrons and Extremely Large VLDL (mmol/L) | 651 (0.28) | 1.08 (1.04-1.12) | 9.62×10^-5^ | 1.07 (1.03-1.11) | 6.50×10^-4^ |
| Cholesterol to Total Lipids in Small LDL (%) | 2,406 (1.04) | 0.92 (0.88-0.95) | 2.67×10^-5^ | 0.92 (0.88-0.96) | 3.79×10^-5^ |
| Triglycerides to Total Lipids in IDL (%) | 1,150 (0.50) | 1.08 (1.03-1.12) | 4.48×10^-4^ | 1.07 (1.03-1.12) | 1.69×10^-3^ |

Abbreviations: CI, confidence interval; CRC, colorectal cancer; HR, hazard ratio; IDL, intermediate-density lipoprotein; LDL, low-density lipoprotein; SD, standard deviation; VLDL, very low-density lipoprotein.

^a^ Biomarkers presented in the table were selected by the final backward stepwise Cox model (*P*-value <0.10).

^b^ Biomarker levels outside of four IQRs from median were considered outliers and removed from analyses.

^c^ HRs and 95% CIs for 1-SD increase in biomarker levels were estimated using the base Cox model with the age as the time scale, adjusting for sex, race, educational attainment, fasting time, self-reported cholesterol-lowering medication use, and CRC family history and CRC screening history.

^d^ The full Cox model was additionally adjusted for alcohol drinking, cigarette smoking, fruits/vegetables intake, and processed/red meat intake.

**Supplementary Table S7.** Associations of ten selected metabolic biomarkers with colorectal cancer risk after inclusion of participants with missing measurements on any biomarkers, the UK Biobank cohort^a^

| **Metabolic biomarker** | **No. of cases** | **No. of participants** | **HR^b^ (95%CI)** | ***P*-value** | **HR^c^ (95%CI)** | ***P*-value** |
| --- | --- | --- | --- | --- | --- | --- |
| Triglycerides to Phosphoglycerides (ratio) | 2,650 | 254,379 | 1.09 (1.05-1.13) | 1.87×10^-5^ | 1.09 (1.04-1.13) | 4.91×10^-5^ |
| Linoleic Acid to Total Fatty Acids (%) | 2,650 | 254,378 | 0.90 (0.86-0.93) | 1.29×10^-7^ | 0.91 (0.87-0.95) | 1.43×10^-5^ |
| Saturated Fatty Acids to Total Fatty Acids (%) | 2,650 | 254,378 | 1.10 (1.06-1.14) | 3.23×10^-7^ | 1.08 (1.04-1.13) | 3.96×10^-5^ |
| Omega-6 Fatty Acids to Total Fatty Acids (%) | 2,650 | 254,378 | 0.91 (0.88-0.95) | 1.24×10^-6^ | 0.92 (0.89-0.96) | 7.19×10^-5^ |
| Glycoprotein Acetyls (mmol/L) | 2,653 | 254,600 | 1.08 (1.04-1.13) | 3.33×10^-5^ | 1.07 (1.03-1.12) | 2.83×10^-4^ |
| 3-Hydroxybutyrate (mmol/L) | 2,595 | 249,922 | 1.05 (1.02-1.08) | 1.11×10^-3^ | 1.04 (1.01-1.07) | 3.28×10^-3^ |
| Concentration of Chylomicrons and Extremely Large VLDL Particles (mmol/L) | 2,653 | 254,597 | 1.09 (1.05-1.13) | 1.56×10^-5^ | 1.08 (1.04-1.12) | 1.33×10^-4^ |
| Triglycerides in Chylomicrons and Extremely Large VLDL (mmol/L) | 2,653 | 254,597 | 1.09 (1.05-1.13) | 6.19×10^-6^ | 1.08 (1.04-1.12) | 6.07×10^-5^ |
| Cholesterol to Total Lipids in Small LDL (%) | 2,653 | 254,597 | 0.93 (0.90-0.96) | 2.14×10^-6^ | 0.93 (0.90-0.96) | 3.49×10^-6^ |
| Triglycerides to Total Lipids in IDL (%) | 2,653 | 254,597 | 1.08 (1.04-1.12) | 6.61×10^-5^ | 1.07 (1.03-1.11) | 3.44×10^-4^ |

Abbreviations: CI, confidence interval; CRC, colorectal cancer; HR, hazard ratio; IDL, intermediate-density lipoprotein; LDL, low-density lipoprotein; SD, standard deviation; VLDL, very low-density lipoprotein.

^a^ Biomarkers presented in the table were selected by the final backward stepwise Cox model (*P*-value <0.10).

^b^ HRs and 95% CIs for 1-SD increase in biomarker levels were estimated using the base Cox model with the age as the time scale, adjusting for sex, race, educational attainment, fasting time, self-reported cholesterol-lowering medication use, and CRC family history and CRC screening history.

^c^ The full Cox model was additionally adjusted for alcohol drinking, tobacco smoking, fruit/vegetables intake, and processed/red meat intake.

**Supplementary Table S8.** Associations of ten selected metabolic biomarkers with colorectal cancer risk with missing covariates imputed using the sex-specific median or mode, the UK Biobank cohort^a^

| **Metabolic biomarker** | **HR^b^ (95% CI)** | ***P*-value** | **HR^c^ (95% CI)** | ***P*-value** |
| --- | --- | --- | --- | --- |
| Triglycerides to Phosphoglycerides (ratio) | 1.09 (1.04-1.13) | 7.81×10^-5^ | 1.08 (1.04-1.13) | 2.62×10^-4^ |
| Linoleic Acid to Total Fatty Acids (%) | 0.90 (0.86-0.94) | 6.70×10^-7^ | 0.91 (0.87-0.95) | 3.18×10^-5^ |
| Saturated Fatty Acids to Total Fatty Acids (%) | 1.10 (1.06-1.14) | 2.14×10^-6^ | 1.08 (1.04-1.13) | 9.23×10^-5^ |
| Omega-6 Fatty Acids to Total Fatty Acids (%) | 0.91 (0.87-0.95) | 3.84×10^-6^ | 0.92 (0.89-0.96) | 1.23×10^-4^ |
| Glycoprotein Acetyls (mmol/L) | 1.09 (1.05-1.13) | 3.32×10^-5^ | 1.08 (1.03-1.12) | 3.06×10^-4^ |
| 3-Hydroxybutyrate (mmol/L) | 1.06 (1.03-1.10) | 8.85×10^-5^ | 1.06 (1.03-1.09) | 2.24×10^-4^ |
| Concentration of Chylomicrons and Extremely Large VLDL Particles (mmol/L) | 1.08 (1.04-1.12) | 8.85×10^-5^ | 1.07 (1.03-1.11) | 6.26×10^-4^ |
| Triglycerides in Chylomicrons and Extremely Large VLDL (mmol/L) | 1.08 (1.04-1.12) | 4.70×10^-5^ | 1.07 (1.03-1.11) | 3.71×10^-4^ |
| Cholesterol to Total Lipids in Small LDL (%) | 0.93 (0.90-0.96) | 7.46×10^-6^ | 0.93 (0.90-0.96) | 1.24×10^-5^ |
| Triglycerides to Total Lipids in IDL (%) | 1.07 (1.03-1.12) | 5.01×10^-4^ | 1.07 (1.02-1.11) | 2.07×10^-3^ |

Abbreviations: CI, confidence interval; CRC, colorectal cancer; HR, hazard ratio; IDL, intermediate-density lipoprotein; LDL, low-density lipoprotein; SD, standard deviation; VLDL, very low-density lipoprotein.

^a^ Biomarkers presented in the table were selected by the final backward stepwise Cox model (*P*-value <0.10).

^b^ HRs and 95% CIs for 1-SD increase in biomarker levels were estimated using the base Cox model with the age as the time scale, adjusting for sex, race, educational attainment, fasting time, and CRC family history and CRC screening history.

^c^ The full Cox model was additionally adjusted for alcohol drinking, cigarette smoking, fruits/vegetables intake, and processed/red meat intake.

**Supplementary Table S9.** Sex-specific associations between ten selected biomarkers and colorectal cancer risk, the UK Biobank cohort^a^

|  | **Female** | |  | **Male** | |  |
| --- | --- | --- | --- | --- | --- | --- |
| **No. of Events** | ***N* = 996** | |  | ***N* = 1,414** | |  |
| **Metabolic biomarker** | **HR^b^ (95%CI)** | ***P*-value** |  | **HR^b^ (95%CI)** | ***P*-value** | ***P_het_*^c^** |
| Triglycerides to Phosphoglycerides (ratio) | 1.05 (0.98-1.13) | 0.16 |  | 1.10 (1.05-1.16) | 2.43×10^-4^ | 0.32 |
| Linoleic Acid to Total Fatty Acids (%) | 0.99 (0.92-1.06) | 0.76 |  | 0.87 (0.82-0.92) | 3.59×10^-7^ | 0.002 |
| Saturated Fatty Acids to Total Fatty Acids (%) | 1.02 (0.95-1.09) | 0.53 |  | 1.12 (1.06-1.17) | 9.43×10^-6^ | 0.01 |
| Omega-6 Fatty Acids to Total Fatty Acids (%) | 0.97 (0.90-1.04) | 0.42 |  | 0.90 (0.85-0.94) | 1.56×10^-5^ | 0.02 |
| Glycoprotein Acetyls (mmol/L) | 1.07 (1.00-1.14) | 0.05 |  | 1.09 (1.04-1.15) | 1.15×10^-3^ | 0.45 |
| 3-Hydroxybutyrate (mmol/L) | 1.05 (1.00-1.11) | 0.03 |  | 1.06 (1.02-1.11) | 2.61×10^-3^ | 0.56 |
| Concentration of Chylomicrons and Extremely Large VLDL Particles (mmol/L) | 1.04 (0.97-1.11) | 0.24 |  | 1.09 (1.04-1.14) | 3.57×10^-4^ | 0.21 |
| Triglycerides in Chylomicrons and Extremely Large VLDL (mmol/L) | 1.04 (0.97-1.11) | 0.25 |  | 1.09 (1.04-1.14) | 2.16×10^-4^ | 0.23 |
| Cholesterol to Total Lipids in Small LDL (%) | 0.97 (0.91-1.03) | 0.25 |  | 0.92 (0.88-0.95) | 3.32×10^-6^ | 0.34 |
| Triglycerides to Total Lipids in IDL (%) | 1.00 (0.93-1.08) | 0.91 |  | 1.09 (1.04-1.14) | 2.95×10^-4^ | 0.12 |

Abbreviations: CI, confidence interval; CRC, colorectal cancer; HDL, high-density lipoprotein; HR, hazard ratio; IDL, intermediate-density lipoprotein; LDL, low-density lipoprotein; standard deviation.

^a^ Biomarkers presented in the table were selected by the final backward stepwise Cox model (*P*-value <0.10).

^b^ HRs and 95% CIs for 1-SD increase in biomarker levels were estimated using the Cox model with the age as the time scale, adjusting for sex, race, educational attainment, fasting time, self-reported cholesterol-lowering medication use, CRC family history and CRC screening history, alcohol drinking, cigarette smoking, fruits/vegetables intake, and processed/red meat intake.

^c^ *P*_heterogeneity_ for sex was calculated from the likelihood ratio test comparing models with and without an interaction term between each metabolic biomarker and sex.

**Supplementary Table S10.** Subsite-specific associations between ten selected biomarkers and colorectal cancer risk, the UK Biobank cohort^a^

|  | **Proximal colon** | |  | **Distal colon** | |  | **Rectum** | |  |
| --- | --- | --- | --- | --- | --- | --- | --- | --- | --- |
| **No. of Events** | ***N* = 908** | |  | ***N* = 644** | |  | ***N* = 792** | |  |
| **Metabolic biomarker** | **HR^b^ (95%CI)** | ***P*-value** |  | **HR^b^ (95%CI)** | ***P*-value** |  | **HR^b^ (95%CI)** | ***P*-value** | ***P_het_*^c^** |
| Triglycerides to Phosphoglycerides (ratio) | 1.14 (1.06-1.22) | 2.16×10^-4^ |  | 1.06 (0.97-1.14) | 0.19 |  | 1.04 (0.97-1.12) | 0.29 | 0.92 |
| Linoleic Acid to Total Fatty Acids (%) | 0.88 (0.82-0.95) | 9.67×10^-4^ |  | 0.93 (0.86-1.02) | 0.11 |  | 0.93 (0.86-1.00) | 0.06 | 0.89 |
| Saturated Fatty Acids to Total Fatty Acids (%) | 1.11 (1.04-1.19) | 2.00×10^-3^ |  | 1.10 (1.02-1.18) | 0.02 |  | 1.04 (0.97-1.11) | 0.24 | 0.79 |
| Omega-6 Fatty Acids to Total Fatty Acids (%) | 0.89 (0.84-0.96) | 1.33×10^-3^ |  | 0.93 (0.86-1.01) | 0.07 |  | 0.95 (0.89-1.02) | 0.17 | 0.99 |
| Glycoprotein Acetyls (mmol/L) | 1.10 (1.03-1.18) | 2.98×10^-3^ |  | 1.04 (0.96-1.13) | 0.29 |  | 1.07 (0.99-1.15) | 0.08 | 0.41 |
| 3-Hydroxybutyrate (mmol/L) | 1.03 (0.97-1.09) | 0.31 |  | 1.07 (1.02-1.13) | 0.01 |  | 1.08 (1.02-1.13) | 3.18×10^-3^ | 0.78 |
| Concentration of Chylomicrons and Extremely Large VLDL Particles (mmol/L) | 1.10 (1.03-1.17) | 3.08×10^-3^ |  | 1.06 (0.98-1.14) | 0.15 |  | 1.04 (0.98-1.12) | 0.21 | 0.96 |
| Triglycerides in Chylomicrons and Extremely Large VLDL (mmol/L) | 1.11 (1.04-1.18) | 9.67×10^-4^ |  | 1.05 (0.98-1.13) | 0.16 |  | 1.04 (0.98-1.11) | 0.22 | 0.97 |
| Cholesterol to Total Lipids in Small LDL (%) | 0.90 (0.86-0.94) | 2.00×10^-7^ |  | 0.97 (0.90-1.04) | 0.40 |  | 0.95 (0.89-1.00) | 0.07 | 0.35 |
| Triglycerides to Total Lipids in IDL (%) | 1.13 (1.06-1.21) | 1.28×10^-4^ |  | 1.01 (0.94-1.10) | 0.73 |  | 1.03 (0.96-1.10) | 0.41 | 0.54 |

Abbreviations: CI, confidence interval; CRC, colorectal cancer; HDL, high-density lipoprotein; HR, hazard ratio; IDL, intermediate-density lipoprotein; SD, standard deviation.

^a^ Biomarkers presented in the table were selected by the final backward stepwise Cox model (*P*-value <0.10).

^b^ HRs and 95% CIs for 1-SD increase in biomarker levels were estimated using the Cox model with the age as the time scale, adjusting for sex, race, educational attainment, fasting time, self-reported cholesterol-lowering medication use, CRC family history and CRC screening history, alcohol drinking, cigarette smoking, fruits/vegetables intake, and processed/red meat intake.

^c^ *P*_heterogeneity_ for cancer subsite was calculated from a global Wald test for an interaction term between each metabolic biomarker and an indicator of tumor site in the joint Cox model.

**Supplementary Table S11.** Summary of independent, genome-wide significant (*P*-value <5×10^-8^) genetic variants associated with the eight selected metabolic biomarkers, the UK Biobank cohort^a^

| **Metabolic biomarker^b^** | **No. of genetic variants** | **No. of novel variants (%)** |
| --- | --- | --- |
| Log_Triglycerides to Phosphoglycerides (ratio) | 192 | 68 (35%) |
| Linoleic Acid to Total Fatty Acids (%) | 107 | 61 (57%) |
| Saturated Fatty Acids to Total Fatty Acids (%) | 51 | 18 (35%) |
| Omega-6 Fatty Acids to Total Fatty Acids (%) | 142 | 63 (44%) |
| Glycoprotein Acetyls (mmol/L) | 141 | 58 (41%) |
| Log_3-Hydroxybutyrate (mmol/L) | 32 | 9 (28%) |
| Cholesterol to Total Lipids in Small LDL (%) | 64 | 23 (36%) |
| Log_Triglycerides to Total Lipids in IDL (%) | 186 | 68 (37%) |

^a^ Biomarkers presented in the table were selected by the final backward stepwise Cox model (*P*-value <0.01).

^b^ Levels of triglycerides to phosphoglycerides (ratio), 3-hydroxybutyrate (mmol/L), triglycerides to total lipids in IDL (%) were log-transformed before *z*-score standardization.

**Supplementary Table S12.** Summary of the 192 independent, genome-wide significant (*P*-value <5×10^-8^) variants associated with standardized levels of log-transformed triglycerides to phosphoglycerides ratio, the UK Biobank cohort^a^

| **Variant** | **Chr.** | **Position (GRCh37)** | **Effect allele** | **Other allele** | **EAF** | ***N*** | **β** | **SE** | ***P*-value** |
| --- | --- | --- | --- | --- | --- | --- | --- | --- | --- |
| rs2275910 | 1 | 7068679 | G | A | 0.382604 | 198609 | 0.0170054 | 0.00303921 | 2.20E-08 |
| rs193084249 | 1 | 26987646 | G | A | 0.0223444 | 198596 | 0.106732 | 0.0100253 | 1.85E-26 |
| rs72663503 | 1 | 39969059 | T | C | 0.228885 | 198689 | 0.0295006 | 0.00352085 | 5.38E-17 |
| rs213479 | 1 | 54861827 | T | G | 0.466485 | 196791 | -0.0173971 | 0.00298467 | 5.59E-09 |
| rs11207994 | 1 | 63047987 | T | C | 0.350913 | 199162 | -0.0481987 | 0.00309782 | 1.49E-54 |
| rs10889906 | 1 | 71509107 | G | A | 0.299646 | 199549 | 0.0175837 | 0.00321476 | 4.51E-08 |
| 1:93882912_GA_G | 1 | 93882912 | GA | G | 0.216086 | 197347 | -0.021904 | 0.00361116 | 1.32E-09 |
| rs2660302 | 1 | 98520219 | T | A | 0.187215 | 195118 | 0.021493 | 0.00382392 | 1.90E-08 |
| rs587685973 | 1 | 149940804 | AT | A | 0.0824726 | 194610 | -0.0364165 | 0.005432 | 2.03E-11 |
| rs3754212 | 1 | 150738200 | G | A | 0.356881 | 199732 | 0.0209917 | 0.00308261 | 9.81E-12 |
| 1:178502207_GTA_G | 1 | 178502207 | G | GTA | 0.342525 | 198447 | -0.0187449 | 0.00311954 | 1.87E-09 |
| rs4500327 | 1 | 219705205 | T | C | 0.339423 | 198518 | -0.0212258 | 0.00312103 | 1.04E-11 |
| rs17649913 | 1 | 220983402 | C | T | 0.217781 | 196851 | 0.0245477 | 0.00359965 | 9.16E-12 |
| rs2281721 | 1 | 230297136 | C | T | 0.387329 | 198986 | 0.0511189 | 0.00302951 | 7.79E-64 |
| rs2678379 | 2 | 21226560 | A | G | 0.203205 | 199537 | -0.10042 | 0.00365769 | 1.25E-165 |
| rs10193900 | 2 | 25919967 | A | G | 0.314752 | 199160 | 0.0175572 | 0.00318324 | 3.48E-08 |
| rs115268969 | 2 | 27176094 | T | C | 0.0207753 | 199732 | 0.0673889 | 0.0103544 | 7.62E-11 |
| rs6714780 | 2 | 27189063 | A | G | 0.0494939 | 194903 | 0.0585692 | 0.00691271 | 2.41E-17 |
| rs1260326 | 2 | 27730940 | T | C | 0.395933 | 199732 | 0.0846213 | 0.00301331 | 3.49E-173 |
| rs62140395 | 2 | 28244926 | C | G | 0.116709 | 199732 | 0.0466826 | 0.00459186 | 2.84E-24 |
| rs10180284 | 2 | 50716016 | T | C | 0.481408 | 197262 | -0.0165979 | 0.00297644 | 2.46E-08 |
| rs1009358 | 2 | 65276452 | C | T | 0.374942 | 199732 | -0.0172342 | 0.00304279 | 1.48E-08 |
| rs35932591 | 2 | 121310704 | T | C | 0.121182 | 199732 | 0.025381 | 0.00451706 | 1.92E-08 |
| rs13389219 | 2 | 165528876 | T | C | 0.391724 | 199648 | -0.0397452 | 0.00301962 | 1.50E-39 |
| rs148358468 | 2 | 219590348 | A | G | 0.0495169 | 199033 | 0.0373346 | 0.0068172 | 4.34E-08 |
| rs2943650 | 2 | 227105921 | C | T | 0.353034 | 199219 | -0.0470801 | 0.00308618 | 1.63E-52 |
| rs545210244 | 3 | 12337753 | CAAAAAAA | C | 0.14968 | 191235 | -0.0292012 | 0.00421758 | 4.41E-12 |
| rs17052058 | 3 | 52344680 | G | A | 0.180274 | 199474 | -0.0323579 | 0.00383438 | 3.22E-17 |
| rs11626 | 3 | 52873984 | T | C | 0.148819 | 199679 | -0.0269694 | 0.00413705 | 7.09E-11 |
| rs4678322 | 3 | 135812523 | T | G | 0.22889 | 197492 | -0.0293386 | 0.00353272 | 1.01E-16 |
| rs1471741 | 3 | 136328268 | T | C | 0.262234 | 197108 | -0.0305969 | 0.00337729 | 1.32E-19 |
| rs9844972 | 3 | 150097635 | C | G | 0.0671974 | 195878 | 0.0406972 | 0.00594383 | 7.57E-12 |
| rs9817452 | 3 | 156795414 | T | G | 0.386634 | 198336 | -0.0201147 | 0.003054 | 4.52E-11 |
| rs79287178 | 3 | 172294500 | A | G | 0.0254299 | 195321 | 0.0583352 | 0.00947622 | 7.48E-10 |
| rs13101828 | 4 | 965720 | G | A | 0.4523 | 197074 | -0.0185321 | 0.00298545 | 5.39E-10 |
| rs13108218 | 4 | 3443931 | A | G | 0.380849 | 193468 | 0.0229161 | 0.00308587 | 1.12E-13 |
| rs4450871 | 4 | 4990298 | G | A | 0.442851 | 199732 | -0.0165563 | 0.00296631 | 2.39E-08 |
| 4:15095077_AT_A | 4 | 15095077 | AT | A | 0.336445 | 191682 | -0.0200125 | 0.00318222 | 3.21E-10 |
| rs10019888 | 4 | 26062990 | G | A | 0.163981 | 197584 | 0.031294 | 0.00401074 | 6.10E-15 |
| rs7661844 | 4 | 87468625 | C | T | 0.492756 | 198860 | 0.0206975 | 0.00295437 | 2.46E-12 |
| rs1471251 | 4 | 87976359 | T | A | 0.398271 | 198238 | 0.0272113 | 0.00302805 | 2.58E-19 |
| rs10428504 | 4 | 89716933 | T | G | 0.462585 | 199079 | 0.019998 | 0.00296641 | 1.57E-11 |
| rs13107325 | 4 | 103188709 | T | C | 0.0750481 | 199732 | 0.0387024 | 0.00560671 | 5.11E-12 |
| rs6822892 | 4 | 157734675 | G | A | 0.327331 | 199732 | -0.0187539 | 0.00314324 | 2.43E-09 |
| rs72754154 | 5 | 55812380 | A | G | 0.0496712 | 199512 | -0.0499072 | 0.00680318 | 2.21E-13 |
| rs9687846 | 5 | 55861894 | A | G | 0.200023 | 199732 | 0.0524677 | 0.00368294 | 4.99E-46 |
| rs37538 | 5 | 57610069 | G | C | 0.397381 | 196298 | 0.0176854 | 0.00304233 | 6.14E-09 |
| rs4976033 | 5 | 67714246 | G | A | 0.399685 | 189857 | 0.0191511 | 0.00308726 | 5.54E-10 |
| rs559320993 | 5 | 78433154 | ATG | A | 0.348096 | 196295 | -0.0211863 | 0.00311587 | 1.05E-11 |
| rs1045241 | 5 | 118729286 | T | C | 0.269662 | 196880 | -0.0216744 | 0.00335255 | 1.01E-10 |
| rs72801474 | 5 | 132444128 | A | G | 0.0923487 | 199732 | -0.0311584 | 0.00510091 | 1.01E-09 |
| rs1501908 | 5 | 156398169 | G | C | 0.364466 | 199732 | -0.0279546 | 0.00306528 | 7.60E-20 |
| rs1428445 | 5 | 157995803 | A | G | 0.211198 | 195442 | 0.0255704 | 0.00364493 | 2.30E-12 |
| rs6939900 | 6 | 31228357 | T | C | 0.354755 | 196916 | 0.0263287 | 0.00310973 | 2.54E-17 |
| rs187050905 | 6 | 31975259 | A | G | 0.0153105 | 199569 | 0.110488 | 0.0120357 | 4.35E-20 |
| rs11755694 | 6 | 32586408 | T | A | 0.0384814 | 199564 | 0.0798381 | 0.00768049 | 2.65E-25 |
| rs114760566 | 6 | 34192036 | A | C | 0.0449393 | 199191 | 0.0557635 | 0.00712595 | 5.08E-15 |
| rs998584 | 6 | 43757896 | A | C | 0.482444 | 197653 | 0.0358146 | 0.00296785 | 1.61E-33 |
| rs881858 | 6 | 43806609 | G | A | 0.306819 | 199732 | -0.019362 | 0.00319971 | 1.44E-09 |
| rs4715317 | 6 | 52629010 | T | G | 0.35583 | 198817 | 0.0181256 | 0.00309235 | 4.60E-09 |
| rs2186037 | 6 | 127455029 | A | G | 0.481809 | 199715 | -0.0308696 | 0.00295256 | 1.41E-25 |
| rs113385622 | 6 | 130402567 | AT | A | 0.263656 | 195211 | -0.0253189 | 0.00337829 | 6.68E-14 |
| rs5880430 | 6 | 139829695 | T | TTGAA | 0.369898 | 197303 | 0.03332 | 0.00307334 | 2.22E-27 |
| rs9986540 | 6 | 160242487 | T | G | 0.0146715 | 199093 | -0.149233 | 0.0122846 | 6.05E-34 |
| rs614754 | 6 | 160505199 | C | G | 0.0124985 | 196704 | -0.0830489 | 0.0133816 | 5.44E-10 |
| rs12208357 | 6 | 160543148 | T | C | 0.0693072 | 198890 | 0.0490284 | 0.00581638 | 3.50E-17 |
| rs9295128 | 6 | 160751531 | T | G | 0.015129 | 198824 | -0.256969 | 0.012128 | 1.58E-99 |
| rs10455872 | 6 | 161010118 | G | A | 0.0781597 | 199732 | -0.145327 | 0.00549154 | 4.67E-154 |
| rs143843429 | 6 | 161383079 | G | A | 0.0132337 | 197790 | -0.194621 | 0.0129832 | 9.07E-51 |
| rs61735260 | 6 | 161560461 | A | G | 0.0123666 | 199732 | -0.105178 | 0.0133451 | 3.25E-15 |
| rs38205 | 7 | 15913588 | A | C | 0.37612 | 193405 | 0.0197576 | 0.00309444 | 1.72E-10 |
| rs10242866 | 7 | 17920613 | T | C | 0.398976 | 199042 | 0.0205919 | 0.00301554 | 8.60E-12 |
| rs10642257 | 7 | 25992323 | AAGGCC | A | 0.158993 | 198528 | -0.0324846 | 0.00404505 | 9.74E-16 |
| rs2699814 | 7 | 26406800 | A | T | 0.490221 | 198442 | -0.0176255 | 0.00295291 | 2.39E-09 |
| rs878521 | 7 | 44255643 | A | G | 0.253601 | 198211 | 0.0218119 | 0.00340805 | 1.56E-10 |
| rs71551223 | 7 | 71256489 | C | A | 0.0303886 | 196982 | -0.0494677 | 0.0086527 | 1.09E-08 |
| rs36104871 | 7 | 71808047 | T | G | 0.0278364 | 197906 | -0.0582915 | 0.00901118 | 9.90E-11 |
| rs35753501 | 7 | 72330303 | T | C | 0.0260739 | 197439 | -0.092649 | 0.00931506 | 2.65E-23 |
| rs147767686 | 7 | 72389537 | T | C | 0.0257306 | 197702 | -0.0754642 | 0.00937444 | 8.33E-16 |
| rs13234131 | 7 | 73025975 | G | A | 0.128098 | 199660 | -0.120283 | 0.00440855 | 1.31E-163 |
| rs28494095 | 7 | 73097720 | C | G | 0.0724896 | 199732 | 0.0355026 | 0.00569795 | 4.65E-10 |
| rs3996350 | 7 | 130427057 | C | G | 0.492223 | 193595 | -0.0371323 | 0.00299872 | 3.34E-35 |
| rs570759412 | 7 | 150307215 | TAGA | T | 0.228526 | 198958 | 0.0218085 | 0.00352011 | 5.83E-10 |
| rs2980755 | 8 | 8363683 | G | A | 0.457295 | 193758 | -0.0259073 | 0.00301131 | 7.80E-18 |
| rs7012814 | 8 | 9173358 | A | G | 0.472908 | 195059 | -0.030415 | 0.00299387 | 3.06E-24 |
| rs7833387 | 8 | 10009533 | A | G | 0.48495 | 194815 | -0.0224685 | 0.00298448 | 5.16E-14 |
| rs7821812 | 8 | 10644101 | C | G | 0.207065 | 199350 | 0.0373167 | 0.00364545 | 1.38E-24 |
| rs4500049 | 8 | 11521079 | A | T | 0.475884 | 198564 | 0.0272242 | 0.00296112 | 3.82E-20 |
| rs35246381 | 8 | 18272535 | C | T | 0.221226 | 199563 | 0.0244609 | 0.00355288 | 5.80E-12 |
| rs59347135 | 8 | 19750044 | G | C | 0.041798 | 196002 | 0.0871832 | 0.00744695 | 1.20E-31 |
| rs117026536 | 8 | 19818773 | T | G | 0.098912 | 199273 | -0.214381 | 0.00492187 | 1.75E-412 |
| rs143298923 | 8 | 19919713 | A | T | 0.010852 | 199549 | -0.0984918 | 0.0142488 | 4.78E-12 |
| rs117174179 | 8 | 19941078 | T | G | 0.0191311 | 199257 | 0.158444 | 0.0107821 | 7.35E-49 |
| rs146449080 | 8 | 19969742 | G | A | 0.0151501 | 199206 | 0.0842693 | 0.0120952 | 3.24E-12 |
| rs13253288 | 8 | 59338856 | G | C | 0.336482 | 198480 | 0.0196812 | 0.00313206 | 3.31E-10 |
| rs28446899 | 8 | 72396213 | T | C | 0.076018 | 199124 | 0.0365974 | 0.00557499 | 5.23E-11 |
| 8:116636377_CA_C | 8 | 116636377 | CA | C | 0.398442 | 197804 | 0.0193947 | 0.00303216 | 1.60E-10 |
| rs28601761 | 8 | 126500031 | G | C | 0.415999 | 190634 | -0.0714628 | 0.00306097 | 2.21E-120 |
| rs11997051 | 8 | 126648243 | C | T | 0.220567 | 196471 | 0.0223436 | 0.00358777 | 4.74E-10 |
| rs1567353 | 9 | 1033773 | G | C | 0.30579 | 197508 | 0.0191139 | 0.0032302 | 3.28E-09 |
| rs296884 | 9 | 86582923 | T | G | 0.254863 | 198046 | -0.0210354 | 0.00340435 | 6.46E-10 |
| rs62565259 | 9 | 102162570 | T | C | 0.169132 | 196359 | -0.0224127 | 0.00396812 | 1.62E-08 |
| rs7924036 | 10 | 65191645 | G | T | 0.497248 | 199655 | 0.0368159 | 0.00295267 | 1.14E-35 |
| rs703974 | 10 | 80948593 | G | A | 0.415412 | 199732 | -0.0175016 | 0.00299486 | 5.11E-09 |
| rs4418728 | 10 | 94839724 | T | G | 0.450046 | 199505 | -0.0270643 | 0.00296309 | 6.67E-20 |
| rs8181419 | 10 | 95340811 | G | T | 0.154187 | 195250 | 0.0233292 | 0.00412714 | 1.58E-08 |
| rs2803619 | 10 | 113934384 | G | C | 0.275634 | 199654 | -0.0279821 | 0.00330811 | 2.72E-17 |
| rs12786130 | 11 | 27505954 | C | T | 0.236864 | 196300 | 0.0209032 | 0.00350722 | 2.53E-09 |
| rs4603265 | 11 | 46708196 | T | C | 0.13745 | 199450 | -0.0349784 | 0.00429055 | 3.59E-16 |
| rs10838681 | 11 | 47275064 | A | G | 0.266983 | 199732 | -0.0281595 | 0.00332688 | 2.60E-17 |
| rs12798109 | 11 | 47889850 | T | C | 0.347548 | 199377 | 0.021644 | 0.0030922 | 2.58E-12 |
| rs174564 | 11 | 61588305 | G | A | 0.351172 | 199381 | 0.0619716 | 0.00309141 | 2.66E-89 |
| rs796771530 | 11 | 62386647 | GA | G | 0.375453 | 189751 | 0.0172686 | 0.00312354 | 3.23E-08 |
| rs71468663 | 11 | 64018104 | AC | A | 0.0454995 | 199189 | 0.0720307 | 0.0070818 | 2.70E-24 |
| rs678614 | 11 | 64799894 | A | C | 0.278072 | 199173 | 0.0206192 | 0.00329977 | 4.15E-10 |
| rs10750766 | 11 | 65473798 | C | A | 0.288669 | 198617 | -0.0217742 | 0.00326485 | 2.58E-11 |
| rs10793130 | 11 | 75459869 | A | G | 0.0877607 | 197127 | 0.0357504 | 0.00525726 | 1.05E-11 |
| rs117059907 | 11 | 79591327 | T | C | 0.0241198 | 199732 | 0.053911 | 0.00961078 | 2.03E-08 |
| rs117794084 | 11 | 116433496 | T | G | 0.0163269 | 199732 | 0.11578 | 0.0116241 | 2.30E-23 |
| rs964184 | 11 | 116648917 | G | C | 0.133884 | 199732 | 0.204539 | 0.00430767 | 2.46E-489 |
| rs12721043 | 11 | 116692293 | A | C | 0.0103939 | 199732 | -0.2101 | 0.0145602 | 3.55E-47 |
| rs76604009 | 11 | 117085261 | T | C | 0.0353961 | 199584 | -0.0614047 | 0.00799405 | 1.58E-14 |
| rs116987336 | 11 | 117175658 | A | G | 0.0264705 | 199732 | 0.149301 | 0.0091637 | 1.22E-59 |
| rs117619191 | 11 | 117248283 | C | T | 0.0213211 | 199732 | 0.0566338 | 0.0102109 | 2.92E-08 |
| rs17120765 | 11 | 117316546 | A | G | 0.0959873 | 197573 | 0.0281776 | 0.00504766 | 2.38E-08 |
| 11:118935420_AAAAAT_A | 11 | 118935420 | A | AAAAAT | 0.395048 | 192693 | 0.0181701 | 0.00307325 | 3.38E-09 |
| rs76895963 | 12 | 4384844 | G | T | 0.0137608 | 193848 | -0.0841732 | 0.0128422 | 5.60E-11 |
| rs11045172 | 12 | 20470221 | C | A | 0.196462 | 197369 | -0.0326331 | 0.00373333 | 2.33E-18 |
| rs11045247 | 12 | 20592624 | A | G | 0.0736587 | 199732 | 0.0315165 | 0.00565335 | 2.48E-08 |
| rs68147365 | 12 | 57789359 | A | G | 0.239991 | 199389 | -0.0299748 | 0.00345639 | 4.27E-18 |
| 12:58318167_CTT_C | 12 | 58318167 | C | CTT | 0.0687657 | 196675 | -0.0336782 | 0.00587619 | 9.98E-09 |
| rs755820106 | 12 | 107194132 | G | GAA | 0.233044 | 199591 | 0.0206921 | 0.00349286 | 3.15E-09 |
| rs653178 | 12 | 112007756 | C | T | 0.484657 | 199732 | 0.0166817 | 0.00295381 | 1.63E-08 |
| rs11065363 | 12 | 121388498 | T | C | 0.157575 | 198921 | 0.0258227 | 0.00406706 | 2.17E-10 |
| rs11058058 | 12 | 122635983 | A | G | 0.168023 | 198041 | 0.0271516 | 0.00396268 | 7.31E-12 |
| rs642895 | 12 | 123158291 | T | C | 0.125122 | 197998 | -0.02574 | 0.00447523 | 8.85E-09 |
| rs580063 | 12 | 123206340 | C | T | 0.209801 | 199174 | -0.0301371 | 0.00362216 | 8.84E-17 |
| rs10773000 | 12 | 123736084 | T | G | 0.331877 | 199732 | -0.0233622 | 0.00314054 | 1.02E-13 |
| rs7133378 | 12 | 124409502 | A | G | 0.318511 | 198522 | -0.0367078 | 0.00317527 | 6.68E-31 |
| rs838886 | 12 | 125264287 | T | G | 0.297294 | 190004 | -0.0187923 | 0.00331527 | 1.44E-08 |
| rs7140110 | 13 | 114544024 | C | T | 0.298031 | 198902 | 0.0202878 | 0.00323651 | 3.66E-10 |
| rs12897637 | 14 | 64239351 | C | T | 0.158806 | 198462 | 0.0224476 | 0.00405773 | 3.17E-08 |
| 14:71457281_CA_C | 14 | 71457281 | C | CA | 0.335466 | 195486 | -0.020513 | 0.00315711 | 8.19E-11 |
| rs61704439 | 15 | 40755812 | CAA | C | 0.0848588 | 196438 | -0.0296576 | 0.00533745 | 2.76E-08 |
| rs7167078 | 15 | 41972392 | G | C | 0.311892 | 199067 | -0.0231357 | 0.00319101 | 4.17E-13 |
| rs146131796 | 15 | 42450180 | T | A | 0.0179171 | 199000 | 0.0674756 | 0.0111485 | 1.43E-09 |
| rs149492745 | 15 | 43099550 | T | C | 0.0183066 | 196896 | 0.110417 | 0.0110999 | 2.62E-23 |
| rs150844304 | 15 | 43726625 | C | A | 0.0255049 | 199687 | 0.133271 | 0.00935993 | 5.57E-46 |
| rs144022883 | 15 | 44363739 | A | G | 0.0248253 | 199675 | 0.118926 | 0.0094793 | 4.32E-36 |
| rs4273010 | 15 | 44947434 | C | T | 0.0239015 | 198732 | 0.103112 | 0.00968001 | 1.73E-26 |
| rs1601934 | 15 | 58671721 | G | A | 0.310619 | 197454 | -0.025978 | 0.00320855 | 5.69E-16 |
| rs11638671 | 15 | 63795628 | C | T | 0.343596 | 199679 | 0.0203622 | 0.00310639 | 5.58E-11 |
| rs4776793 | 15 | 66872114 | T | C | 0.354206 | 199720 | 0.0192164 | 0.00308612 | 4.77E-10 |
| rs11075253 | 16 | 15148646 | A | C | 0.295646 | 199732 | -0.0274174 | 0.00323812 | 2.53E-17 |
| rs575803453 | 16 | 56389888 | T | C | 0.0108349 | 198386 | 0.0941979 | 0.014257 | 3.93E-11 |
| rs36229491 | 16 | 56994244 | TA | T | 0.322362 | 199223 | -0.074629 | 0.0031624 | 5.85E-123 |
| rs2408345 | 16 | 62807401 | A | G | 0.481406 | 198510 | -0.016997 | 0.00296196 | 9.57E-09 |
| rs862320 | 16 | 69651866 | T | C | 0.409605 | 198744 | -0.0188064 | 0.0030069 | 4.00E-10 |
| rs35524855 | 16 | 72213494 | TA | T | 0.102309 | 197891 | 0.0301265 | 0.00488197 | 6.80E-10 |
| rs2925979 | 16 | 81534790 | T | C | 0.300107 | 199732 | 0.0379562 | 0.00321901 | 4.44E-32 |
| rs11658311 | 17 | 17470526 | T | C | 0.0599727 | 199424 | 0.0404799 | 0.00621867 | 7.56E-11 |
| rs12945575 | 17 | 40713071 | T | C | 0.247814 | 198752 | 0.0223899 | 0.00342463 | 6.25E-11 |
| 17:41166479_TTC_T | 17 | 41166479 | T | TTC | 0.0124303 | 198386 | 0.0810887 | 0.0133404 | 1.22E-09 |
| rs72836561 | 17 | 41926126 | T | C | 0.0311593 | 199732 | 0.177156 | 0.00849352 | 1.64E-96 |
| rs78027755 | 17 | 41956178 | A | G | 0.0321853 | 198740 | 0.0507688 | 0.00837243 | 1.33E-09 |
| rs8064331 | 17 | 42858950 | T | C | 0.258147 | 198650 | -0.0189371 | 0.00338009 | 2.12E-08 |
| 17:46972479_ATCTC_A | 17 | 46972479 | ATCTC | A | 0.469406 | 198783 | -0.0171067 | 0.00296066 | 7.57E-09 |
| rs2671654 | 17 | 47468011 | G | A | 0.423654 | 196016 | -0.0178541 | 0.00301919 | 3.35E-09 |
| rs7211380 | 17 | 64206768 | G | A | 0.0672596 | 198968 | -0.0368749 | 0.00590904 | 4.37E-10 |
| rs149394327 | 17 | 64228995 | C | G | 0.0295992 | 199448 | -0.0722496 | 0.0087078 | 1.07E-16 |
| rs62084237 | 17 | 65854807 | A | G | 0.174872 | 193336 | 0.0249539 | 0.00395284 | 2.74E-10 |
| rs12601079 | 17 | 76400329 | G | A | 0.398427 | 194245 | 0.0275683 | 0.00306069 | 2.13E-19 |
| rs8083730 | 18 | 293519 | A | G | 0.0838223 | 199732 | -0.0297217 | 0.0053375 | 2.57E-08 |
| rs11081879 | 18 | 19909866 | A | G | 0.459733 | 197619 | 0.0178603 | 0.00297905 | 2.03E-09 |
| rs77960347 | 18 | 47109955 | G | A | 0.0136383 | 199732 | -0.0784406 | 0.0127128 | 6.83E-10 |
| rs6507937 | 18 | 47164926 | T | C | 0.176357 | 199354 | 0.0235105 | 0.00387702 | 1.33E-09 |
| rs7238484 | 18 | 57735552 | T | G | 0.267917 | 199666 | 0.0219849 | 0.00333439 | 4.31E-11 |
| rs770972940 | 19 | 4968092 | C | CT | 0.345115 | 197517 | -0.017355 | 0.00312755 | 2.88E-08 |
| rs66921136 | 19 | 7197880 | T | C | 0.287635 | 197603 | 0.0225969 | 0.00327723 | 5.40E-12 |
| rs4804833 | 19 | 7970635 | A | G | 0.383759 | 199732 | 0.019309 | 0.00302608 | 1.76E-10 |
| rs116843064 | 19 | 8429323 | A | G | 0.0191381 | 199732 | -0.263319 | 0.0107497 | 2.58E-132 |
| rs116483979 | 19 | 8456738 | T | C | 0.0299523 | 198833 | -0.0588135 | 0.00868916 | 1.30E-11 |
| rs58542926 | 19 | 19379549 | T | C | 0.0745199 | 199732 | -0.0714033 | 0.00562473 | 6.55E-37 |
| rs62102718 | 19 | 33891013 | T | A | 0.285057 | 198246 | 0.0273999 | 0.00328128 | 6.85E-17 |
| rs1065853 | 19 | 45413233 | T | G | 0.0804058 | 199482 | 0.119925 | 0.00542445 | 3.56E-108 |
| rs2902745 | 19 | 56093748 | T | G | 0.116695 | 197339 | 0.0276079 | 0.00462615 | 2.41E-09 |
| rs6073958 | 20 | 44551855 | C | T | 0.195942 | 199462 | 0.0653026 | 0.00371884 | 5.63E-69 |
| rs6124885 | 20 | 45534053 | G | A | 0.0545204 | 198733 | 0.0388085 | 0.00650181 | 2.39E-09 |
| rs66806308 | 20 | 46360465 | T | C | 0.207541 | 192988 | -0.020771 | 0.00369681 | 1.93E-08 |
| rs8121509 | 20 | 62712053 | C | T | 0.452406 | 199732 | -0.0187397 | 0.00296434 | 2.59E-10 |
| rs2267375 | 22 | 38601231 | G | T | 0.419039 | 199213 | -0.0240485 | 0.00299682 | 1.02E-15 |
| rs763121 | 22 | 38879940 | G | A | 0.343539 | 199392 | 0.0184015 | 0.00310787 | 3.21E-09 |

Abbreviations: Chr., chromosome; EAF, effect allele frequency; LD, linkage disequilibrium; SE, standard error.

^a^ Linear regression model was used to obtain effect estimates and SEs, adjusting for age, sex, genotyping arrays, and the first ten principal components for population structure. LD clumping was then performed to identify independently associated variants (*r*^2^ <1×10^-3^ in a 500kb window).

**Supplementary Table S13.** Summary of the 107 independent, genome-wide significant (*P*-value <5×10^-8^) variants associated with standardized levels of linoleic acid relative to total fatty acids, the UK Biobank cohort^a^

| **Variant** | **Chr.** | **Position (GRCh37)** | **Effect allele** | **Other allele** | **EAF** | ***N*** | **β** | **SE** | ***P*-value** |
| --- | --- | --- | --- | --- | --- | --- | --- | --- | --- |
| rs2843128 | 1 | 2315680 | A | G | 0.483888 | 199,732 | -0.02398 | 0.003044 | 3.40E-15 |
| rs11249249 | 1 | 25758134 | A | G | 0.440982 | 195,931 | -0.01827 | 0.003093 | 3.45E-09 |
| rs3768321 | 1 | 40035928 | T | G | 0.196947 | 199,064 | -0.0251 | 0.003835 | 5.94E-11 |
| rs139500832 | 1 | 51111506 | GA | G | 0.360958 | 189,914 | 0.0188 | 0.003252 | 7.47E-09 |
| rs11591147 | 1 | 55505647 | T | G | 0.017178 | 199,732 | -0.08183 | 0.01173 | 3.04E-12 |
| rs472495 | 1 | 55521313 | G | T | 0.349945 | 199,257 | -0.01828 | 0.003193 | 1.03E-08 |
| rs59770119 | 1 | 219664627 | G | A | 0.432012 | 195,747 | 0.017137 | 0.003104 | 3.39E-08 |
| rs62129551 | 2 | 26940912 | T | C | 0.059532 | 194,249 | -0.04447 | 0.006534 | 1.00E-11 |
| rs6714780 | 2 | 27189063 | A | G | 0.049494 | 194,903 | -0.05842 | 0.007135 | 2.66E-16 |
| rs1260326 | 2 | 27730940 | T | C | 0.395933 | 199,732 | -0.08869 | 0.00311 | 1.70E-178 |
| rs74873433 | 2 | 28277414 | C | T | 0.060941 | 197,789 | -0.04525 | 0.006407 | 1.64E-12 |
| rs7349418 | 2 | 28443050 | C | T | 0.44351 | 193,414 | -0.03275 | 0.003117 | 8.13E-26 |
| rs4614977 | 2 | 44087024 | G | C | 0.060366 | 199,482 | -0.0359 | 0.006411 | 2.15E-08 |
| rs359254 | 2 | 60482427 | A | G | 0.361161 | 199,202 | 0.018254 | 0.003176 | 9.06E-09 |
| rs10195252 | 2 | 165513091 | C | T | 0.404394 | 199,732 | 0.026739 | 0.003103 | 6.90E-18 |
| rs2972137 | 2 | 227106361 | G | A | 0.352294 | 199,356 | 0.027941 | 0.003187 | 1.82E-18 |
| rs9862795 | 3 | 49915506 | A | T | 0.471077 | 199,216 | 0.019808 | 0.003061 | 9.74E-11 |
| rs6796333 | 3 | 52359678 | C | T | 0.180275 | 199,254 | 0.022396 | 0.00396 | 1.55E-08 |
| rs4678428 | 3 | 135988412 | G | A | 0.249981 | 198,007 | 0.027554 | 0.003534 | 6.38E-15 |
| rs6439657 | 3 | 136494639 | T | C | 0.255278 | 199,336 | 0.020773 | 0.003496 | 2.83E-09 |
| rs203440 | 4 | 16242574 | C | T | 0.143052 | 196,159 | 0.024204 | 0.004389 | 3.50E-08 |
| rs538717442 | 4 | 17883603 | GA | G | 0.144233 | 189,846 | -0.0256 | 0.004444 | 8.36E-09 |
| rs11735092 | 4 | 88226231 | C | T | 0.436966 | 198,053 | -0.01885 | 0.003084 | 9.84E-10 |
| rs809955 | 4 | 140874760 | A | G | 0.365234 | 198,974 | 0.01975 | 0.003171 | 4.72E-10 |
| rs40270 | 5 | 55804552 | A | C | 0.226668 | 199,371 | 0.029307 | 0.003637 | 7.74E-16 |
| rs34966499 | 5 | 74621574 | AT | A | 0.393768 | 198,029 | 0.034912 | 0.003129 | 6.85E-29 |
| rs10056543 | 5 | 75143624 | C | A | 0.318836 | 197,340 | 0.01892 | 0.003289 | 8.77E-09 |
| rs1717565 | 5 | 78440369 | C | T | 0.351604 | 199,732 | 0.018139 | 0.003181 | 1.19E-08 |
| rs111676697 | 6 | 11089529 | T | G | 0.225161 | 199,009 | -0.02061 | 0.003659 | 1.76E-08 |
| rs79220007 | 6 | 26098474 | C | T | 0.076642 | 199,499 | -0.03332 | 0.005728 | 5.99E-09 |
| 6:30066988_GGAAA_G | 6 | 30066988 | G | GGAAA | 0.1951 | 197,676 | 0.022823 | 0.003856 | 3.25E-09 |
| rs9266834 | 6 | 31383130 | A | T | 0.10652 | 192,672 | -0.03355 | 0.005026 | 2.46E-11 |
| rs998584 | 6 | 43757896 | A | C | 0.482444 | 197,653 | -0.02122 | 0.003063 | 4.30E-12 |
| rs9370162 | 6 | 52728779 | G | A | 0.42876 | 198,133 | -0.02378 | 0.003092 | 1.47E-14 |
| rs199607859 | 6 | 139835418 | G | T | 0.404853 | 197,441 | -0.02588 | 0.003117 | 1.00E-16 |
| rs112939444 | 6 | 161073048 | C | CA | 0.39733 | 194,984 | -0.0303 | 0.00315 | 6.85E-22 |
| rs17138358 | 7 | 17920253 | C | G | 0.398832 | 198,937 | -0.02146 | 0.003114 | 5.51E-12 |
| rs4719841 | 7 | 25997536 | G | A | 0.407233 | 199,732 | -0.02567 | 0.003097 | 1.14E-16 |
| rs34881399 | 7 | 72234176 | C | T | 0.030819 | 199,732 | 0.051361 | 0.008811 | 5.58E-09 |
| rs13234378 | 7 | 73026151 | T | A | 0.128125 | 199,668 | 0.070789 | 0.004556 | 2.05E-54 |
| rs10232076 | 7 | 98948904 | T | C | 0.080966 | 199,633 | 0.031767 | 0.005582 | 1.27E-08 |
| rs200015011 | 7 | 130426554 | CTCG | C | 0.491601 | 191,270 | 0.018445 | 0.003115 | 3.21E-09 |
| rs17410962 | 8 | 19848080 | A | G | 0.117587 | 199,687 | 0.069362 | 0.004727 | 1.02E-48 |
| rs117174179 | 8 | 19941078 | T | G | 0.019131 | 199,257 | -0.06528 | 0.011132 | 4.52E-09 |
| rs28601761 | 8 | 126500031 | G | C | 0.415999 | 190,634 | 0.06862 | 0.00316 | 2.00E-104 |
| rs16931853 | 9 | 14495337 | A | G | 0.123024 | 197,234 | -0.02757 | 0.004658 | 3.25E-09 |
| rs113904294 | 9 | 86582925 | TAATA | T | 0.248948 | 197,945 | 0.026709 | 0.003542 | 4.72E-14 |
| rs4149307 | 9 | 107589744 | T | C | 0.1539 | 199,500 | 0.023953 | 0.004219 | 1.37E-08 |
| rs13284054 | 9 | 107669073 | C | T | 0.117793 | 193,870 | -0.03301 | 0.0048 | 6.16E-12 |
| 9:136149709_AC_A | 9 | 136149709 | A | AC | 0.175116 | 196,476 | 0.031307 | 0.004047 | 1.03E-14 |
| rs12257549 | 10 | 46029631 | T | C | 0.238764 | 195,111 | 0.019847 | 0.003607 | 3.76E-08 |
| 10:93751550_GTGTGTATATATATATATATATATA_G | 10 | 93751550 | GTGTGTATATATATATATATATATA | G | 0.354769 | 194,889 | 0.018933 | 0.003225 | 4.33E-09 |
| rs4418728 | 10 | 94839724 | T | G | 0.450046 | 199,505 | 0.020694 | 0.003058 | 1.33E-11 |
| rs2146017 | 10 | 96677971 | G | T | 0.435785 | 198,662 | 0.019199 | 0.003081 | 4.62E-10 |
| rs17309874 | 11 | 27667236 | A | G | 0.260025 | 198,462 | -0.0225 | 0.003488 | 1.13E-10 |
| rs552238593 | 11 | 43788754 | TA | T | 0.396466 | 194,313 | 0.020667 | 0.00316 | 6.16E-11 |
| rs3136449 | 11 | 46744470 | A | G | 0.13749 | 199,673 | 0.035293 | 0.004424 | 1.50E-15 |
| rs78290566 | 11 | 47249919 | CG | C | 0.155386 | 198,567 | 0.029781 | 0.004215 | 1.61E-12 |
| rs115713382 | 11 | 49462519 | G | A | 0.180419 | 198,438 | -0.02303 | 0.003974 | 6.77E-09 |
| rs61742921 | 11 | 61025737 | T | C | 0.031181 | 196,336 | -0.04999 | 0.008844 | 1.59E-08 |
| 11:61542006_GA_G | 11 | 61542006 | G | GA | 0.350595 | 194,234 | 0.180508 | 0.003211 | 3.64E-683 |
| rs35169799 | 11 | 64031241 | T | C | 0.063483 | 199,732 | -0.03786 | 0.006241 | 1.31E-09 |
| rs10750766 | 11 | 65473798 | C | A | 0.288669 | 198,617 | 0.025265 | 0.00337 | 6.51E-14 |
| rs10160784 | 11 | 75456055 | C | T | 0.156754 | 199,178 | 0.035102 | 0.004202 | 6.64E-17 |
| rs964184 | 11 | 116648917 | G | C | 0.133884 | 199,732 | -0.05403 | 0.00447 | 1.25E-33 |
| rs12721043 | 11 | 116692293 | A | C | 0.010394 | 199,732 | 0.091634 | 0.015035 | 1.10E-09 |
| rs76895963 | 12 | 4384844 | G | T | 0.013761 | 193,848 | 0.074138 | 0.013266 | 2.29E-08 |
| rs10774625 | 12 | 111910219 | A | G | 0.497812 | 199,732 | -0.01899 | 0.003048 | 4.61E-10 |
| rs73412716 | 12 | 112545197 | T | G | 0.065832 | 197,031 | 0.038801 | 0.006184 | 3.51E-10 |
| rs34680764 | 12 | 124513777 | G | T | 0.119867 | 199,220 | 0.025496 | 0.004677 | 4.99E-08 |
| rs12590259 | 14 | 58741125 | C | A | 0.415085 | 197,486 | -0.01757 | 0.003108 | 1.58E-08 |
| rs17108967 | 14 | 71542168 | C | T | 0.336476 | 198,579 | 0.017676 | 0.003229 | 4.40E-08 |
| rs7156419 | 14 | 102189963 | A | G | 0.054404 | 194,490 | 0.038355 | 0.006803 | 1.72E-08 |
| rs2412710 | 15 | 42683787 | A | G | 0.018024 | 199,732 | -0.06543 | 0.011451 | 1.11E-08 |
| rs143875230 | 15 | 43278726 | A | G | 0.023723 | 198,566 | -0.08445 | 0.010035 | 3.93E-17 |
| rs139974673 | 15 | 44027885 | C | T | 0.025504 | 199,540 | -0.1074 | 0.009666 | 1.13E-28 |
| rs144972973 | 15 | 44564692 | G | A | 0.024372 | 198,961 | -0.08774 | 0.009894 | 7.47E-19 |
| rs753768034 | 15 | 58681533 | G | GT | 0.383455 | 198,631 | -0.01918 | 0.003141 | 1.02E-09 |
| rs261342 | 15 | 58731153 | G | C | 0.216682 | 196,608 | -0.02061 | 0.003726 | 3.17E-08 |
| rs16966947 | 16 | 15141045 | C | T | 0.306233 | 198,997 | 0.02458 | 0.003305 | 1.03E-13 |
| rs12103006 | 16 | 24726237 | A | G | 0.429869 | 198,471 | 0.0181 | 0.003084 | 4.40E-09 |
| rs2288004 | 16 | 31054040 | C | G | 0.380567 | 199,732 | 0.017889 | 0.003133 | 1.13E-08 |
| rs11508026 | 16 | 56999328 | T | C | 0.430885 | 198,047 | 0.030702 | 0.003091 | 3.07E-23 |
| rs9906673 | 17 | 17746197 | C | T | 0.348619 | 198,764 | -0.02451 | 0.0032 | 1.88E-14 |
| rs12945575 | 17 | 40713071 | T | C | 0.247814 | 198,752 | -0.01997 | 0.003535 | 1.60E-08 |
| rs72836561 | 17 | 41926126 | T | C | 0.031159 | 199,732 | -0.05381 | 0.008775 | 8.67E-10 |
| rs60856912 | 17 | 65892343 | T | G | 0.161754 | 197,331 | -0.02339 | 0.004161 | 1.89E-08 |
| rs77542162 | 17 | 67081278 | G | A | 0.02264 | 199,732 | 0.058516 | 0.010243 | 1.11E-08 |
| rs4789182 | 17 | 73374945 | G | A | 0.269256 | 199,106 | 0.021002 | 0.003435 | 9.71E-10 |
| rs4969187 | 17 | 76405837 | T | C | 0.371659 | 193,169 | -0.01903 | 0.003206 | 2.93E-09 |
| rs150703258 | 18 | 21079921 | C | CTGTATGTA | 0.458927 | 193,243 | 0.018287 | 0.00311 | 4.09E-09 |
| rs58324296 | 19 | 4969199 | C | A | 0.350928 | 196,116 | 0.019863 | 0.003228 | 7.63E-10 |
| rs12610987 | 19 | 7219697 | T | C | 0.399446 | 196,163 | 0.022033 | 0.003145 | 2.45E-12 |
| rs116843064 | 19 | 8429323 | A | G | 0.019138 | 199,732 | 0.102653 | 0.01111 | 2.48E-20 |
| rs151113958 | 19 | 11191201 | AG | A | 0.117182 | 198,393 | -0.04694 | 0.004744 | 4.47E-23 |
| rs58542926 | 19 | 19379549 | T | C | 0.07452 | 199,732 | 0.047714 | 0.005807 | 2.10E-16 |
| rs62102718 | 19 | 33891013 | T | A | 0.285057 | 198,246 | -0.0205 | 0.003386 | 1.43E-09 |
| rs62116778 | 19 | 44894070 | T | G | 0.038867 | 199,732 | -0.04376 | 0.007876 | 2.76E-08 |
| rs7412 | 19 | 45412079 | T | C | 0.080806 | 199,732 | -0.17685 | 0.00558 | 7.20E-220 |
| rs2238691 | 19 | 46179043 | A | G | 0.193675 | 199,435 | -0.0236 | 0.003854 | 9.13E-10 |
| rs419772 | 19 | 54794205 | C | T | 0.21652 | 197,598 | 0.020782 | 0.003731 | 2.56E-08 |
| rs6073972 | 20 | 44590298 | G | C | 0.18384 | 199,587 | -0.04923 | 0.003933 | 6.06E-36 |
| rs6063046 | 20 | 45596378 | G | A | 0.281783 | 199,510 | 0.020961 | 0.003386 | 6.04E-10 |
| rs11697876 | 20 | 62444662 | C | T | 0.372135 | 199,468 | 0.018097 | 0.003148 | 9.02E-09 |
| rs9637192 | 21 | 46637413 | T | C | 0.44225 | 195,152 | 0.017029 | 0.003107 | 4.22E-08 |
| 22:38603571_CCAGTAGCTGGGACTA_C | 22 | 38603571 | CCAGTAGCTGGGACTA | C | 0.462527 | 197,033 | 0.020166 | 0.003076 | 5.57E-11 |
| rs3747207 | 22 | 44324855 | A | G | 0.215165 | 199,291 | 0.036338 | 0.003706 | 1.09E-22 |

Abbreviations: Chr., chromosome; EAF, effect allele frequency; LD, linkage disequilibrium; SE, standard error.

^a^ Linear regression model was used to obtain effect estimates and SEs, adjusting for age, sex, genotyping arrays, and the first ten principal components for population structure. LD clumping was then performed to identify independently associated variants (*r*^2^ <1×10^-3^ in a 500kb window).

**Supplementary Table S14.** Summary of the 51 independent, genome-wide significant (*P*-value <5×10^-8^) variants associated with standardized levels of saturated fatty acids relative to total fatty acids, the UK Biobank cohort^a^

| **Variant** | **Chr.** | **Position (GRCh37)** | **Effect allele** | **Other allele** | **EAF** | ***N*** | **β** | **SE** | ***P*-value** |
| --- | --- | --- | --- | --- | --- | --- | --- | --- | --- |
| rs12045101 | 1 | 110267651 | T | C | 0.239174 | 198,222 | -0.02274 | 0.003653 | 4.79E-10 |
| rs113520997 | 1 | 178523678 | G | GGGT | 0.47021 | 196,796 | 0.019318 | 0.003135 | 7.16E-10 |
| rs2642438 | 1 | 220970028 | A | G | 0.29697 | 199,732 | -0.0445 | 0.003395 | 3.03E-39 |
| rs62129551 | 2 | 26940912 | T | C | 0.059532 | 194,249 | 0.043551 | 0.006661 | 6.22E-11 |
| rs1260326 | 2 | 27730940 | T | C | 0.395933 | 199,732 | 0.068247 | 0.003173 | 1.70E-102 |
| rs62140396 | 2 | 28246841 | A | G | 0.116513 | 199,708 | 0.039485 | 0.004834 | 3.17E-16 |
| rs13389219 | 2 | 165528876 | T | C | 0.391724 | 199,648 | -0.02564 | 0.003178 | 7.13E-16 |
| rs2972146 | 2 | 227100698 | G | T | 0.351806 | 199,732 | -0.02325 | 0.003246 | 7.89E-13 |
| rs1100396 | 3 | 132021906 | C | T | 0.279643 | 197,021 | 0.019263 | 0.003478 | 3.04E-08 |
| rs4918 | 3 | 186338382 | G | C | 0.355105 | 199,607 | 0.022192 | 0.003249 | 8.47E-12 |
| rs13150834 | 4 | 88222673 | G | T | 0.436843 | 198,759 | 0.017586 | 0.003139 | 2.11E-08 |
| rs1229863 | 4 | 100252386 | A | T | 0.173375 | 199,717 | 0.027675 | 0.004107 | 1.61E-11 |
| rs13102909 | 4 | 111110678 | A | G | 0.385139 | 198,413 | -0.02121 | 0.003199 | 3.39E-11 |
| rs40270 | 5 | 55804552 | A | C | 0.226668 | 199,371 | -0.02436 | 0.003707 | 5.01E-11 |
| rs2893910 | 6 | 27283254 | T | A | 0.194693 | 199,732 | 0.021471 | 0.003925 | 4.48E-08 |
| rs3130561 | 6 | 31100768 | T | C | 0.287628 | 199,153 | -0.02366 | 0.003433 | 5.52E-12 |
| rs1077394 | 6 | 31610384 | C | T | 0.326733 | 199,732 | -0.02375 | 0.003314 | 7.67E-13 |
| rs2269423 | 6 | 32145707 | A | C | 0.375038 | 199,732 | -0.03429 | 0.003212 | 1.38E-26 |
| rs34531599 | 6 | 32663978 | T | C | 0.115856 | 198,224 | -0.03094 | 0.00487 | 2.10E-10 |
| rs540973884 | 6 | 139837431 | T | G | 0.397487 | 190,191 | 0.019057 | 0.003251 | 4.57E-09 |
| rs9295128 | 6 | 160751531 | T | G | 0.015129 | 198,824 | -0.10075 | 0.012773 | 3.10E-15 |
| rs55730499 | 6 | 161005610 | T | C | 0.078714 | 199,489 | -0.07397 | 0.00577 | 1.32E-37 |
| rs143843429 | 6 | 161383079 | G | A | 0.013234 | 197,790 | -0.0834 | 0.013662 | 1.03E-09 |
| rs200015011 | 7 | 130426554 | CTCG | C | 0.491601 | 191,270 | -0.02508 | 0.003176 | 2.90E-15 |
| rs7018333 | 8 | 59402570 | A | G | 0.32796 | 197,448 | 0.022126 | 0.003328 | 2.95E-11 |
| rs28601761 | 8 | 126500031 | G | C | 0.415999 | 190,634 | -0.05204 | 0.003223 | 1.33E-58 |
| rs563294622 | 9 | 86548308 | CT | C | 0.243469 | 193,612 | -0.03279 | 0.003679 | 5.01E-19 |
| rs12263369 | 10 | 94823343 | C | T | 0.40771 | 199,188 | -0.01832 | 0.003161 | 6.77E-09 |
| rs773231679 | 10 | 101405507 | C | CCTTT | 0.049266 | 199,093 | 0.042973 | 0.007177 | 2.13E-09 |
| rs603424 | 10 | 102075479 | A | G | 0.173245 | 199,732 | 0.063743 | 0.004106 | 2.51E-54 |
| rs4917902 | 10 | 102274998 | C | G | 0.219493 | 199,489 | -0.03985 | 0.003745 | 1.97E-26 |
| rs10787429 | 10 | 113949664 | T | C | 0.274175 | 199,132 | 0.038166 | 0.00349 | 8.04E-28 |
| 11:61596322_CA_C | 11 | 61596322 | C | CA | 0.347615 | 199,295 | -0.04673 | 0.003264 | 1.87E-46 |
| rs7943745 | 11 | 68605828 | T | C | 0.450189 | 198,552 | 0.028923 | 0.003133 | 2.70E-20 |
| rs3135506 | 11 | 116662407 | C | G | 0.063077 | 199,732 | -0.03828 | 0.006372 | 1.89E-09 |
| rs229798 | 14 | 83757483 | G | A | 0.289729 | 195,659 | -0.01945 | 0.003454 | 1.78E-08 |
| rs10468017 | 15 | 58678512 | T | C | 0.295023 | 199,732 | 0.04999 | 0.003405 | 8.86E-49 |
| rs1800588 | 15 | 58723675 | T | C | 0.21454 | 199,732 | 0.045596 | 0.003781 | 1.76E-33 |
| rs34779241 | 17 | 40824834 | G | A | 0.287888 | 198,301 | 0.026172 | 0.003441 | 2.84E-14 |
| rs77381129 | 17 | 41596947 | A | G | 0.05524 | 198,616 | 0.04206 | 0.006803 | 6.30E-10 |
| rs62089321 | 17 | 73272551 | C | T | 0.277423 | 195,386 | -0.02135 | 0.003501 | 1.07E-09 |
| rs2228305 | 17 | 80042792 | T | C | 0.029675 | 199,732 | -0.05193 | 0.009175 | 1.51E-08 |
| rs10659627 | 19 | 7193978 | G | GCTT | 0.289165 | 197,045 | 0.01913 | 0.00345 | 2.94E-08 |
| rs8105174 | 19 | 10347032 | T | C | 0.18674 | 199,732 | -0.02305 | 0.003982 | 7.16E-09 |
| rs7412 | 19 | 45412079 | T | C | 0.080806 | 199,732 | 0.055155 | 0.005701 | 3.92E-22 |
| rs56784978 | 19 | 45499388 | C | T | 0.316538 | 198,820 | -0.01862 | 0.003343 | 2.56E-08 |
| rs452270 | 19 | 49239053 | C | T | 0.495443 | 196,733 | 0.028274 | 0.003136 | 1.98E-19 |
| rs6073958 | 20 | 44551855 | C | T | 0.195942 | 199,462 | 0.033837 | 0.003914 | 5.45E-18 |
| rs5746641 | 22 | 18911733 | C | T | 0.0422 | 199,457 | -0.06093 | 0.007717 | 2.90E-15 |
| rs574686543 | 22 | 18976317 | A | T | 0.110893 | 197,966 | -0.03107 | 0.004946 | 3.35E-10 |
| rs738409 | 22 | 44324727 | G | C | 0.217169 | 199,732 | -0.0439 | 0.003761 | 1.79E-31 |

Abbreviations: Chr., chromosome; EAF: effect allele frequency; LD, linkage disequilibrium; SE, standard error.

^a^ Linear regression model was used to obtain effect estimates and SEs, adjusting for age, sex, genotyping arrays, and the first ten principal components for population structure. LD clumping was then performed to identify independently associated variants (*r*^2^ <1×10^-3^ in a 500kb window).

**Supplementary Table S15.** Summary of the 142 independent, genome-wide significant (*P*-value <5×10^-8^) variants associated with standardized levels of omega-6 fatty acids relative to total fatty acids, the UK Biobank cohort^a^

| **Variant** | **Chr.** | **Position (GRCh37)** | **Effect allele** | **Other allele** | **EAF** | ***N*** | **β** | **SE** | ***P*-value** |
| --- | --- | --- | --- | --- | --- | --- | --- | --- | --- |
| rs2843128 | 1 | 2315680 | A | G | 0.483888 | 199,732 | -0.01791 | 0.003063 | 5.00E-09 |
| rs193084249 | 1 | 26987646 | G | A | 0.0223444 | 198,596 | -0.06337 | 0.010413 | 1.16E-09 |
| 1:39948182_TTTTG_T | 1 | 39948182 | T | TTTTG | 0.228729 | 195,382 | -0.02578 | 0.003685 | 2.67E-12 |
| rs139500832 | 1 | 51111506 | GA | G | 0.360958 | 189,914 | 0.019252 | 0.003272 | 4.03E-09 |
| rs10889334 | 1 | 62957199 | G | C | 0.351044 | 199,106 | 0.045496 | 0.003218 | 2.28E-45 |
| rs6658257 | 1 | 93820684 | A | G | 0.397179 | 199,502 | 0.021484 | 0.003131 | 6.80E-12 |
| rs2660302 | 1 | 98520219 | T | A | 0.187215 | 195,118 | -0.02243 | 0.003971 | 1.63E-08 |
| rs16844101 | 1 | 199011539 | T | G | 0.170117 | 197,591 | 0.022965 | 0.004101 | 2.14E-08 |
| rs2820426 | 1 | 219660535 | A | G | 0.394678 | 196,587 | 0.021572 | 0.003159 | 8.61E-12 |
| rs59484402 | 1 | 230302838 | C | CT | 0.388763 | 190,552 | -0.02188 | 0.003216 | 1.02E-11 |
| rs4564803 | 2 | 21205502 | T | G | 0.225164 | 199,648 | 0.043794 | 0.003667 | 7.33E-33 |
| rs10193900 | 2 | 25919967 | A | G | 0.314752 | 199,160 | -0.02205 | 0.003306 | 2.55E-11 |
| rs62129551 | 2 | 26940912 | T | C | 0.0595318 | 194,249 | -0.05326 | 0.006574 | 5.48E-16 |
| rs114856131 | 2 | 27156721 | T | C | 0.0128661 | 197,341 | -0.07622 | 0.013677 | 2.51E-08 |
| rs143915183 | 2 | 27174079 | A | G | 0.0418133 | 198,932 | -0.05661 | 0.007659 | 1.46E-13 |
| rs6714780 | 2 | 27189063 | A | G | 0.0494939 | 194,903 | -0.07278 | 0.007176 | 3.65E-24 |
| rs1260326 | 2 | 27730940 | T | C | 0.395933 | 199,732 | -0.12056 | 0.003124 | 1.24E-324 |
| rs62140395 | 2 | 28244926 | C | G | 0.116709 | 199,732 | -0.06748 | 0.004768 | 1.85E-45 |
| rs111750756 | 2 | 28476360 | T | C | 0.0153156 | 198,327 | -0.07042 | 0.01252 | 1.87E-08 |
| rs359252 | 2 | 60481644 | T | C | 0.383499 | 199,278 | 0.017412 | 0.003157 | 3.47E-08 |
| rs2165107 | 2 | 111931350 | G | C | 0.462691 | 199,361 | 0.021136 | 0.003078 | 6.60E-12 |
| rs13389219 | 2 | 165528876 | T | C | 0.391724 | 199,648 | 0.034746 | 0.003136 | 1.63E-28 |
| rs151200853 | 2 | 219291481 | T | C | 0.0444219 | 197,234 | -0.04477 | 0.007483 | 2.21E-09 |
| rs2972137 | 2 | 227106361 | G | A | 0.352294 | 199,356 | 0.036181 | 0.003206 | 1.56E-29 |
| rs6800707 | 3 | 52516293 | C | G | 0.188152 | 198,645 | 0.02607 | 0.003926 | 3.15E-11 |
| rs2564921 | 3 | 53125585 | T | C | 0.43741 | 199,178 | 0.018524 | 0.003087 | 1.97E-09 |
| rs684773 | 3 | 135956305 | A | C | 0.232557 | 199,732 | 0.032366 | 0.003624 | 4.22E-19 |
| rs7427111 | 3 | 136495041 | C | T | 0.255732 | 199,283 | 0.025743 | 0.003516 | 2.46E-13 |
| rs9844972 | 3 | 150097635 | C | G | 0.0671974 | 195,878 | -0.03491 | 0.006171 | 1.55E-08 |
| rs9817452 | 3 | 156795414 | T | G | 0.386634 | 198,336 | 0.017879 | 0.003171 | 1.72E-08 |
| rs5402 | 3 | 170727739 | A | T | 0.11902 | 197,522 | -0.0274 | 0.004759 | 8.49E-09 |
| rs7700107 | 4 | 17880416 | C | A | 0.138036 | 199,126 | -0.0274 | 0.004446 | 7.15E-10 |
| rs7661844 | 4 | 87468625 | C | T | 0.492756 | 198,860 | -0.0214 | 0.003068 | 3.04E-12 |
| rs1471251 | 4 | 87976359 | T | A | 0.398271 | 198,238 | -0.02871 | 0.003145 | 7.10E-20 |
| rs6825776 | 4 | 89710789 | T | A | 0.371341 | 198,237 | -0.01807 | 0.003181 | 1.35E-08 |
| rs57800857 | 4 | 140863365 | C | A | 0.362494 | 196,373 | 0.017783 | 0.003218 | 3.29E-08 |
| 5:43217142_CAG_C | 5 | 43217142 | C | CAG | 0.0515756 | 197,991 | -0.03922 | 0.006955 | 1.71E-08 |
| rs111366116 | 5 | 53295546 | T | C | 0.111926 | 198,958 | -0.02913 | 0.004872 | 2.25E-09 |
| rs72754154 | 5 | 55812380 | A | G | 0.0496712 | 199,512 | 0.04084 | 0.007066 | 7.47E-09 |
| rs3936511 | 5 | 55860781 | G | A | 0.191429 | 199,620 | -0.03914 | 0.003895 | 9.44E-24 |
| rs1316776 | 5 | 78430607 | A | C | 0.349116 | 199,633 | 0.02095 | 0.003206 | 6.42E-11 |
| rs6882345 | 5 | 156397673 | G | A | 0.365464 | 199,467 | 0.021194 | 0.003183 | 2.75E-11 |
| rs2914231 | 5 | 158011435 | C | G | 0.232923 | 195,097 | -0.02052 | 0.003664 | 2.16E-08 |
| rs7756992 | 6 | 20679709 | G | A | 0.265383 | 199,732 | -0.01976 | 0.003471 | 1.24E-08 |
| rs9266834 | 6 | 31383130 | A | T | 0.10652 | 192,672 | -0.04143 | 0.005056 | 2.55E-16 |
| rs437179 | 6 | 31929014 | A | C | 0.322252 | 199,732 | 0.020967 | 0.003289 | 1.83E-10 |
| rs13191862 | 6 | 32579653 | G | A | 0.456329 | 198,691 | -0.02491 | 0.003086 | 7.03E-16 |
| rs998584 | 6 | 43757896 | A | C | 0.482444 | 197,653 | -0.02903 | 0.003082 | 4.62E-21 |
| rs9370162 | 6 | 52728779 | G | A | 0.42876 | 198,133 | -0.02812 | 0.00311 | 1.56E-19 |
| rs577721086 | 6 | 127440047 | C | T | 0.0478629 | 198,139 | -0.04763 | 0.007202 | 3.75E-11 |
| 6:130389707_TA_T | 6 | 130389707 | T | TA | 0.257301 | 197,230 | 0.020922 | 0.00352 | 2.81E-09 |
| rs199607859 | 6 | 139835418 | G | T | 0.404853 | 197,441 | -0.03007 | 0.003136 | 8.92E-22 |
| rs9986540 | 6 | 160242487 | T | G | 0.0146715 | 199,093 | 0.107143 | 0.01276 | 4.63E-17 |
| rs150856119 | 6 | 160633223 | A | G | 0.0222241 | 195,936 | -0.0637 | 0.010513 | 1.37E-09 |
| rs9295128 | 6 | 160751531 | T | G | 0.015129 | 198,824 | 0.184802 | 0.012602 | 1.16E-48 |
| rs55730499 | 6 | 161005610 | T | C | 0.0787136 | 199,489 | 0.115295 | 0.005692 | 4.03E-91 |
| rs143843429 | 6 | 161383079 | G | A | 0.0132337 | 197,790 | 0.138501 | 0.013484 | 9.63E-25 |
| rs61735260 | 6 | 161560461 | A | G | 0.0123666 | 199,732 | 0.085178 | 0.01386 | 7.99E-10 |
| rs17138358 | 7 | 17920253 | C | G | 0.398832 | 198,937 | -0.0195 | 0.003134 | 4.90E-10 |
| rs4719841 | 7 | 25997536 | G | A | 0.407233 | 199,732 | -0.02628 | 0.003116 | 3.36E-17 |
| rs2699809 | 7 | 26402747 | T | C | 0.483608 | 198,667 | -0.01726 | 0.003065 | 1.77E-08 |
| rs2908298 | 7 | 44192552 | A | G | 0.154488 | 199,601 | -0.02623 | 0.004233 | 5.72E-10 |
| rs35753501 | 7 | 72330303 | T | C | 0.0260739 | 197,439 | 0.085084 | 0.009674 | 1.44E-18 |
| rs147767686 | 7 | 72389537 | T | C | 0.0257306 | 197,702 | 0.070968 | 0.009736 | 3.12E-13 |
| rs13234378 | 7 | 73026151 | T | A | 0.128125 | 199,668 | 0.107436 | 0.00458 | 1.60E-121 |
| rs28494095 | 7 | 73097720 | C | G | 0.0724896 | 199,732 | -0.03703 | 0.005918 | 3.91E-10 |
| rs11762784 | 7 | 130424646 | A | G | 0.492095 | 191,531 | 0.026607 | 0.003132 | 1.99E-17 |
| rs2980755 | 8 | 8363683 | G | A | 0.457295 | 193,758 | 0.017562 | 0.003127 | 1.96E-08 |
| rs28588745 | 8 | 10647044 | T | A | 0.204905 | 198,699 | -0.02814 | 0.003807 | 1.45E-13 |
| rs55865839 | 8 | 11625372 | C | A | 0.223636 | 195,510 | -0.02373 | 0.003715 | 1.69E-10 |
| rs4921913 | 8 | 18272377 | C | T | 0.220846 | 199,657 | -0.0292 | 0.003692 | 2.63E-15 |
| rs59347135 | 8 | 19750044 | G | C | 0.041798 | 196,002 | -0.05636 | 0.007737 | 3.23E-13 |
| rs328 | 8 | 19819724 | G | C | 0.0999114 | 199,732 | 0.138327 | 0.005099 | 9.00E-162 |
| rs117174179 | 8 | 19941078 | T | G | 0.0191311 | 199,257 | -0.10621 | 0.011201 | 2.51E-21 |
| rs11781439 | 8 | 20573754 | A | C | 0.188577 | 199,277 | -0.02203 | 0.003919 | 1.90E-08 |
| rs1128435 | 8 | 27301725 | G | C | 0.0970995 | 198,245 | -0.0285 | 0.005203 | 4.30E-08 |
| rs28601761 | 8 | 126500031 | G | C | 0.415999 | 190,634 | 0.092045 | 0.003177 | 3.80E-184 |
| rs897161 | 8 | 126606217 | A | C | 0.46758 | 198,689 | 0.01764 | 0.003081 | 1.03E-08 |
| rs16931853 | 9 | 14495337 | A | G | 0.123024 | 197,234 | -0.02625 | 0.004688 | 2.16E-08 |
| rs796004 | 9 | 86594798 | T | C | 0.255821 | 199,593 | 0.030493 | 0.003515 | 4.17E-18 |
| 10:5238542_CAGAG_C | 10 | 5238542 | C | CAGAG | 0.157689 | 196,970 | 0.025063 | 0.004246 | 3.59E-09 |
| rs10761785 | 10 | 65318766 | G | T | 0.488788 | 198,320 | -0.02216 | 0.00308 | 6.33E-13 |
| rs1892501 | 10 | 81097266 | G | A | 0.19356 | 199,052 | -0.02155 | 0.003881 | 2.81E-08 |
| rs2792022 | 10 | 93740429 | C | T | 0.275727 | 196,800 | 0.021028 | 0.003457 | 1.18E-09 |
| rs4418728 | 10 | 94839724 | T | G | 0.450046 | 199,505 | 0.030092 | 0.003077 | 1.39E-22 |
| rs2896635 | 11 | 13359745 | T | A | 0.312533 | 199,206 | 0.018934 | 0.003309 | 1.05E-08 |
| rs11030088 | 11 | 27646247 | A | G | 0.251293 | 198,141 | -0.02225 | 0.003553 | 3.79E-10 |
| rs3136449 | 11 | 46744470 | A | G | 0.13749 | 199,673 | 0.035097 | 0.004451 | 3.16E-15 |
| rs10838681 | 11 | 47275064 | A | G | 0.266983 | 199,732 | 0.024473 | 0.003455 | 1.42E-12 |
| rs115713382 | 11 | 49462519 | G | A | 0.180419 | 198,438 | -0.02195 | 0.003998 | 4.03E-08 |
| 11:61596322_CA_C | 11 | 61596322 | C | CA | 0.347615 | 199,295 | 0.030841 | 0.003223 | 1.08E-21 |
| rs71468663 | 11 | 64018104 | AC | A | 0.0454995 | 199,189 | -0.05794 | 0.007357 | 3.39E-15 |
| rs10750766 | 11 | 65473798 | C | A | 0.288669 | 198,617 | 0.023982 | 0.003391 | 1.53E-12 |
| rs117794084 | 11 | 116433496 | T | G | 0.0163269 | 199,732 | -0.083 | 0.012074 | 6.25E-12 |
| rs964184 | 11 | 116648917 | G | C | 0.133884 | 199,732 | -0.15219 | 0.004486 | 1.50E-251 |
| rs12721043 | 11 | 116692293 | A | C | 0.0103939 | 199,732 | 0.135208 | 0.015126 | 3.97E-19 |
| rs116987336 | 11 | 117175658 | A | G | 0.0264705 | 199,732 | -0.11388 | 0.00952 | 5.71E-33 |
| rs76895963 | 12 | 4384844 | G | T | 0.0137608 | 193,848 | 0.09595 | 0.013343 | 6.46E-13 |
| rs11045172 | 12 | 20470221 | C | A | 0.196462 | 197,369 | 0.021257 | 0.003879 | 4.24E-08 |
| rs55695203 | 12 | 21357753 | C | G | 0.150481 | 199,184 | -0.02918 | 0.00429 | 1.05E-11 |
| rs68147365 | 12 | 57789359 | A | G | 0.239991 | 199,389 | 0.023775 | 0.00359 | 3.53E-11 |
| 12:112958774_GTTT_G | 12 | 112958774 | G | GTTT | 0.0982896 | 196,567 | 0.029289 | 0.005182 | 1.59E-08 |
| rs7133378 | 12 | 124409502 | A | G | 0.318511 | 198,522 | 0.022773 | 0.003298 | 5.05E-12 |
| rs112740904 | 13 | 114631075 | G | T | 0.143268 | 198,436 | 0.027517 | 0.00439 | 3.66E-10 |
| rs1951203 | 14 | 58699353 | T | C | 0.471039 | 194,627 | 0.018706 | 0.003104 | 1.67E-09 |
| rs67981189 | 14 | 71472226 | G | A | 0.328712 | 198,680 | 0.021183 | 0.003268 | 9.07E-11 |
| rs7170463 | 15 | 41888918 | G | A | 0.309865 | 198,462 | 0.019456 | 0.003327 | 4.98E-09 |
| rs184334219 | 15 | 42721845 | A | G | 0.0185352 | 199,404 | -0.08838 | 0.011374 | 7.86E-15 |
| rs143875230 | 15 | 43278726 | A | G | 0.0237226 | 198,566 | -0.11855 | 0.010093 | 7.62E-32 |
| rs139974673 | 15 | 44027885 | C | T | 0.0255037 | 199,540 | -0.14483 | 0.009723 | 3.74E-50 |
| rs148489550 | 15 | 44581461 | A | G | 0.0253889 | 199,241 | -0.11303 | 0.009749 | 4.53E-31 |
| rs10468017 | 15 | 58678512 | T | C | 0.295023 | 199,732 | -0.05874 | 0.00336 | 2.16E-68 |
| rs1800588 | 15 | 58723675 | T | C | 0.21454 | 199,732 | -0.07127 | 0.00373 | 2.63E-81 |
| rs2871866 | 15 | 99221888 | C | T | 0.230568 | 196,541 | 0.020505 | 0.003673 | 2.36E-08 |
| rs12103006 | 16 | 24726237 | A | G | 0.429869 | 198,471 | 0.017746 | 0.003103 | 1.08E-08 |
| rs1532625 | 16 | 57005301 | T | C | 0.43923 | 199,516 | 0.02973 | 0.003093 | 7.17E-22 |
| rs34682685 | 16 | 72096227 | A | G | 0.10265 | 198,301 | -0.02823 | 0.005057 | 2.37E-08 |
| rs2925979 | 16 | 81534790 | T | C | 0.300107 | 199,732 | -0.02194 | 0.003344 | 5.31E-11 |
| rs12945575 | 17 | 40713071 | T | C | 0.247814 | 198,752 | -0.02623 | 0.003557 | 1.64E-13 |
| rs113186018 | 17 | 41422469 | A | G | 0.0538171 | 199,732 | -0.03897 | 0.00679 | 9.56E-09 |
| rs72836561 | 17 | 41926126 | T | C | 0.0311593 | 199,732 | -0.08538 | 0.008828 | 4.04E-22 |
| rs116878033 | 17 | 42031770 | T | C | 0.0356224 | 198,120 | -0.04529 | 0.008296 | 4.79E-08 |
| rs4793155 | 17 | 42888381 | T | C | 0.131742 | 199,193 | -0.02538 | 0.004536 | 2.22E-08 |
| rs10775406 | 17 | 46197755 | A | G | 0.239994 | 199,726 | 0.020513 | 0.003592 | 1.12E-08 |
| rs149394327 | 17 | 64228995 | C | G | 0.0295992 | 199,448 | 0.050234 | 0.009044 | 2.79E-08 |
| rs60856912 | 17 | 65892343 | T | G | 0.161754 | 197,331 | -0.02706 | 0.004187 | 1.03E-10 |
| rs12601079 | 17 | 76400329 | G | A | 0.398427 | 194,245 | -0.0201 | 0.003179 | 2.60E-10 |
| rs12979771 | 19 | 4963443 | T | C | 0.314347 | 195,448 | 0.020879 | 0.003344 | 4.26E-10 |
| rs10405423 | 19 | 7211311 | C | A | 0.337965 | 192,875 | 0.022613 | 0.003303 | 7.65E-12 |
| rs116843064 | 19 | 8429323 | A | G | 0.0191381 | 199,732 | 0.177225 | 0.011174 | 1.28E-56 |
| rs58542926 | 19 | 19379549 | T | C | 0.0745199 | 199,732 | 0.080541 | 0.005841 | 3.12E-43 |
| rs62102718 | 19 | 33891013 | T | A | 0.285057 | 198,246 | -0.02364 | 0.003407 | 3.97E-12 |
| rs1065853 | 19 | 45413233 | T | G | 0.0804058 | 199,482 | -0.14507 | 0.005631 | 3.90E-146 |
| rs2238691 | 19 | 46179043 | A | G | 0.193675 | 199,435 | -0.02207 | 0.003878 | 1.27E-08 |
| rs633236 | 19 | 49274975 | T | C | 0.353126 | 196,107 | 0.020569 | 0.003236 | 2.06E-10 |
| rs2902745 | 19 | 56093748 | T | G | 0.116695 | 197,339 | -0.02641 | 0.004805 | 3.88E-08 |
| rs6073958 | 20 | 44551855 | C | T | 0.195942 | 199,462 | -0.06058 | 0.003862 | 2.06E-55 |
| rs6063046 | 20 | 45596378 | G | A | 0.281783 | 199,510 | 0.019072 | 0.003407 | 2.18E-08 |
| rs8115058 | 20 | 62432709 | G | A | 0.370226 | 199,038 | 0.018047 | 0.003175 | 1.32E-08 |
| rs59612585 | 21 | 40682529 | CA | C | 0.370614 | 196,718 | -0.01769 | 0.003202 | 3.27E-08 |
| rs4818766 | 21 | 46635351 | A | G | 0.431862 | 197,150 | 0.018773 | 0.003117 | 1.71E-09 |
| 22:38603571_CCAGTAGCTGGGACTA_C | 22 | 38603571 | CCAGTAGCTGGGACTA | C | 0.462527 | 197,033 | 0.023183 | 0.003095 | 6.95E-14 |

Abbreviations: Chr., chromosome; EAF, effect allele frequency; LD, linkage disequilibrium; SE, standard error.

^a^ Linear regression model was used to obtain effect estimates and SEs, adjusting for age, sex, genotyping arrays, and the first ten principal components for population structure. LD clumping was then performed to identify independently associated variants (*r*^2^ <1×10^-3^ in a 500kb window).

**Supplementary Table S16.** Summary of the 141 independent, genome-wide significant (*P*-value <5×10^-8^) variants associated with standardized levels of glycoprotein acetyls (mmol/L), the UK Biobank cohort^a^

| **Variant** | **Chr.** | **Position (GRCh37)** | **Effect allele** | **Other allele** | **EAF** | ***N*** | **β** | **SE** | ***P*-value** |
| --- | --- | --- | --- | --- | --- | --- | --- | --- | --- |
| rs909537 | 1 | 23810063 | C | G | 0.101112 | 198913 | -0.03159 | 0.005206 | 1.29E-09 |
| rs188468174 | 1 | 25291697 | T | C | 0.013766 | 199084 | -0.1168 | 0.01346 | 4.09E-18 |
| rs114165349 | 1 | 27021913 | C | G | 0.022844 | 199355 | 0.093366 | 0.010505 | 6.26E-19 |
| rs1768808 | 1 | 46503217 | C | T | 0.365213 | 192719 | -0.02253 | 0.003311 | 1.02E-11 |
| rs2131925 | 1 | 63025942 | G | T | 0.354044 | 199732 | -0.03281 | 0.003284 | 1.69E-23 |
| 1:66166933_TA_T | 1 | 66166933 | TA | T | 0.347645 | 198835 | -0.02674 | 0.003298 | 5.10E-16 |
| rs907662 | 1 | 117848822 | A | G | 0.237649 | 197272 | -0.02125 | 0.003706 | 9.88E-09 |
| rs9427403 | 1 | 161494322 | C | A | 0.181216 | 195513 | 0.024568 | 0.004105 | 2.18E-09 |
| 1:205051828_TC_T | 1 | 205051828 | T | TC | 0.190655 | 198720 | 0.026493 | 0.003996 | 3.37E-11 |
| rs17008806 | 1 | 220976257 | C | A | 0.280141 | 197367 | -0.02318 | 0.003505 | 3.74E-11 |
| rs56188865 | 1 | 247606276 | C | T | 0.371882 | 198087 | -0.02478 | 0.003258 | 2.79E-14 |
| rs10184054 | 2 | 21203877 | G | C | 0.221004 | 199392 | -0.02389 | 0.003776 | 2.50E-10 |
| rs72804880 | 2 | 27173314 | A | G | 0.044167 | 199732 | 0.070385 | 0.007607 | 2.21E-20 |
| rs1260326 | 2 | 27730940 | T | C | 0.395933 | 199732 | 0.104722 | 0.003196 | 7.57E-235 |
| rs62140395 | 2 | 28244926 | C | G | 0.116709 | 199732 | 0.062531 | 0.004873 | 1.12E-37 |
| rs59537151 | 2 | 48293911 | CG | C | 0.288611 | 198814 | 0.019426 | 0.003462 | 2.01E-08 |
| rs10931933 | 2 | 202112471 | G | T | 0.390416 | 194869 | -0.01871 | 0.003251 | 8.67E-09 |
| 2:203780885_TTTTA_T | 2 | 203780885 | TTTTA | T | 0.186543 | 199209 | -0.02579 | 0.004023 | 1.46E-10 |
| rs13065730 | 3 | 18710865 | T | C | 0.426827 | 199260 | 0.018274 | 0.003163 | 7.60E-09 |
| rs62260788 | 3 | 48068610 | T | C | 0.09351 | 199288 | 0.032278 | 0.005377 | 1.94E-09 |
| rs4563439 | 3 | 49383779 | T | C | 0.428693 | 198607 | 0.019981 | 0.00317 | 2.90E-10 |
| rs13316065 | 3 | 49884913 | T | C | 0.320757 | 198518 | 0.028874 | 0.003366 | 9.66E-18 |
| rs79287178 | 3 | 172294500 | A | G | 0.02543 | 195321 | 0.082219 | 0.010055 | 2.92E-16 |
| rs2070632 | 3 | 186334004 | A | C | 0.275253 | 196570 | -0.04141 | 0.003539 | 1.28E-31 |
| rs71603401 | 4 | 18034463 | G | A | 0.131983 | 195643 | 0.027182 | 0.004676 | 6.12E-09 |
| rs13108218 | 4 | 3443931 | A | G | 0.380849 | 193468 | 0.038764 | 0.003275 | 2.62E-32 |
| rs57043448 | 4 | 88022736 | G | GAA | 0.405866 | 196396 | -0.01758 | 0.003218 | 4.71E-08 |
| rs7697204 | 4 | 148980174 | C | T | 0.257613 | 199732 | -0.02327 | 0.003586 | 8.61E-11 |
| 5:95252452_GA_G | 5 | 95252452 | G | GA | 0.279054 | 195170 | 0.020405 | 0.003532 | 7.63E-09 |
| rs72801474 | 5 | 132444128 | A | G | 0.092349 | 199732 | -0.0388 | 0.005414 | 7.69E-13 |
| rs2731673 | 5 | 176839898 | T | C | 0.255296 | 198697 | -0.02357 | 0.003601 | 5.92E-11 |
| rs2523583 | 6 | 31327895 | G | A | 0.29058 | 199558 | -0.05183 | 0.003459 | 9.67E-51 |
| rs5875374 | 6 | 32427005 | A | AAC | 0.331403 | 197035 | -0.04861 | 0.003353 | 1.35E-47 |
| rs2763981 | 6 | 31840021 | T | A | 0.332463 | 198860 | -0.04807 | 0.003333 | 3.95E-47 |
| rs16896742 | 6 | 29922740 | G | A | 0.360333 | 199732 | 0.035657 | 0.003262 | 8.30E-28 |
| rs10455872 | 6 | 161010118 | G | A | 0.07816 | 199732 | -0.06274 | 0.005837 | 6.12E-27 |
| 6:30822002_GAC_G | 6 | 30822002 | G | GAC | 0.149852 | 199704 | -0.03912 | 0.004398 | 5.98E-19 |
| rs1800562 | 6 | 26093141 | A | G | 0.07724 | 199411 | -0.05155 | 0.00587 | 1.61E-18 |
| rs1042133 | 6 | 33048606 | C | G | 0.15257 | 192391 | 0.036935 | 0.004437 | 8.47E-17 |
| rs9295128 | 6 | 160751531 | T | G | 0.015129 | 198824 | -0.10296 | 0.012885 | 1.35E-15 |
| rs1535039 | 6 | 29411432 | C | T | 0.122712 | 199732 | -0.03075 | 0.004783 | 1.29E-10 |
| rs9368222 | 6 | 20686996 | A | C | 0.261731 | 199732 | 0.022028 | 0.003564 | 6.40E-10 |
| rs12208357 | 6 | 160543148 | T | C | 0.069307 | 198890 | 0.037857 | 0.006174 | 8.72E-10 |
| rs6915913 | 6 | 30257751 | C | T | 0.018385 | 199732 | 0.068771 | 0.011635 | 3.41E-09 |
| rs13214703 | 6 | 27941387 | C | T | 0.090784 | 199732 | -0.03173 | 0.00545 | 5.81E-09 |
| rs998584 | 6 | 43757896 | A | C | 0.482444 | 197653 | 0.018279 | 0.003151 | 6.63E-09 |
| rs9374591 | 6 | 116410614 | C | T | 0.399913 | 199341 | 0.018523 | 0.003205 | 7.53E-09 |
| rs12190724 | 6 | 130395960 | G | C | 0.259213 | 199610 | -0.0205 | 0.003568 | 9.20E-09 |
| rs35901019 | 6 | 28494225 | A | G | 0.089904 | 199674 | -0.03132 | 0.005476 | 1.08E-08 |
| rs7746553 | 6 | 31895973 | G | C | 0.151718 | 199732 | 0.024344 | 0.00436 | 2.37E-08 |
| rs3812316 | 7 | 73020337 | G | C | 0.128029 | 199732 | -0.10646 | 0.004683 | 3.01E-114 |
| rs34052301 | 7 | 72340785 | T | G | 0.028668 | 196874 | -0.08513 | 0.009452 | 2.15E-19 |
| rs139392920 | 7 | 72246350 | C | G | 0.023159 | 196277 | -0.08174 | 0.010493 | 6.72E-15 |
| rs1229480 | 7 | 81552011 | T | C | 0.244255 | 194002 | 0.025647 | 0.003697 | 4.01E-12 |
| rs4719841 | 7 | 25997536 | G | A | 0.407233 | 199732 | 0.020087 | 0.003185 | 2.84E-10 |
| rs2070971 | 7 | 44197583 | T | G | 0.135644 | 198132 | 0.026466 | 0.004586 | 7.87E-09 |
| rs10254101 | 7 | 151415536 | T | C | 0.28746 | 199113 | 0.019571 | 0.003463 | 1.60E-08 |
| rs138222746 | 7 | 36258218 | A | G | 0.011795 | 198517 | -0.0819 | 0.014558 | 1.85E-08 |
| rs36104871 | 7 | 71808047 | T | G | 0.027836 | 197906 | -0.05262 | 0.009567 | 3.81E-08 |
| rs28601761 | 8 | 126500031 | G | C | 0.415999 | 190634 | -0.06663 | 0.003249 | 2.35E-93 |
| rs117199990 | 8 | 19820916 | T | C | 0.09972 | 199649 | -0.09037 | 0.005223 | 5.07E-67 |
| rs4841132 | 8 | 9183596 | A | G | 0.091055 | 199732 | -0.05546 | 0.005466 | 3.50E-24 |
| rs2721961 | 8 | 116657911 | G | T | 0.279558 | 197444 | -0.02838 | 0.003507 | 5.86E-16 |
| rs876954 | 8 | 8310923 | A | G | 0.476868 | 197430 | -0.02155 | 0.003154 | 8.36E-12 |
| rs11784312 | 8 | 10872767 | A | G | 0.359224 | 192394 | 0.022489 | 0.003328 | 1.40E-11 |
| rs1132195 | 8 | 11850995 | G | C | 0.45855 | 198783 | 0.019767 | 0.003146 | 3.32E-10 |
| rs670044 | 8 | 9892529 | A | C | 0.333832 | 197704 | -0.02027 | 0.00334 | 1.28E-09 |
| rs150611042 | 9 | 117083803 | A | C | 0.065392 | 193197 | 0.11953 | 0.006439 | 7.45E-77 |
| rs529565 | 9 | 136149500 | C | T | 0.317619 | 199552 | 0.027832 | 0.00337 | 1.49E-16 |
| rs7924036 | 10 | 65191645 | G | T | 0.497248 | 199655 | 0.024918 | 0.003135 | 1.89E-15 |
| rs4418728 | 10 | 94839724 | T | G | 0.450046 | 199505 | -0.02277 | 0.003145 | 4.45E-13 |
| rs1892501 | 10 | 81097266 | G | A | 0.19356 | 199052 | 0.028383 | 0.003966 | 8.29E-13 |
| rs1412445 | 10 | 91002804 | T | C | 0.337473 | 199253 | 0.021235 | 0.003318 | 1.55E-10 |
| rs10430642 | 10 | 89756556 | T | C | 0.290991 | 199001 | 0.019498 | 0.003461 | 1.76E-08 |
| rs201477480 | 10 | 17889023 | C | T | 0.235435 | 199412 | -0.02077 | 0.003697 | 1.93E-08 |
| rs17476364 | 10 | 71094504 | C | T | 0.108053 | 199268 | -0.02755 | 0.005047 | 4.83E-08 |
| rs964184 | 11 | 116648917 | G | C | 0.133884 | 199732 | 0.112675 | 0.004591 | 8.17E-133 |
| rs59379014 | 11 | 126228000 | T | C | 0.072412 | 199579 | 0.047607 | 0.006033 | 3.01E-15 |
| rs12575474 | 11 | 122521021 | C | T | 0.384033 | 199440 | 0.024633 | 0.003225 | 2.22E-14 |
| rs116987336 | 11 | 117175658 | A | G | 0.026471 | 199732 | 0.072139 | 0.009731 | 1.24E-13 |
| rs1519125 | 11 | 16244588 | C | G | 0.387793 | 199484 | 0.021778 | 0.003215 | 1.25E-11 |
| rs2896635 | 11 | 13359745 | T | A | 0.312533 | 199206 | -0.02283 | 0.003381 | 1.46E-11 |
| rs10750766 | 11 | 65473798 | C | A | 0.288669 | 198617 | -0.01977 | 0.003465 | 1.16E-08 |
| rs11231721 | 11 | 63957835 | A | G | 0.058497 | 199327 | 0.036469 | 0.006677 | 4.71E-08 |
| rs3741414 | 12 | 57844049 | T | C | 0.241385 | 199619 | -0.02865 | 0.003661 | 5.04E-15 |
| rs76895963 | 12 | 4384844 | G | T | 0.013761 | 193848 | -0.09867 | 0.013631 | 4.54E-13 |
| rs35762468 | 12 | 83994255 | CA | C | 0.370452 | 195510 | -0.01951 | 0.003282 | 2.79E-09 |
| rs2583949 | 12 | 66194243 | T | C | 0.10364 | 196594 | 0.02952 | 0.005172 | 1.15E-08 |
| rs12300845 | 12 | 24195798 | T | G | 0.033054 | 199520 | 0.048481 | 0.008748 | 3.00E-08 |
| rs9604570 | 13 | 114631940 | G | A | 0.183938 | 196949 | -0.02286 | 0.004068 | 1.91E-08 |
| rs28929474 | 14 | 94844947 | T | C | 0.019569 | 199732 | -0.27721 | 0.011315 | 2.41E-132 |
| rs17580 | 14 | 94847262 | A | T | 0.047301 | 199732 | -0.06001 | 0.007382 | 4.34E-16 |
| rs1890942 | 14 | 69765644 | A | G | 0.381384 | 194658 | -0.02058 | 0.003264 | 2.90E-10 |
| rs2332536 | 14 | 71634952 | G | A | 0.352712 | 198770 | -0.01969 | 0.003286 | 2.06E-09 |
| rs150844304 | 15 | 43726625 | C | A | 0.025505 | 199687 | 0.078374 | 0.009938 | 3.13E-15 |
| rs138893177 | 15 | 44297617 | T | C | 0.024741 | 199648 | 0.0696 | 0.010083 | 5.12E-12 |
| rs149492745 | 15 | 43099550 | T | C | 0.018307 | 196896 | 0.075504 | 0.011785 | 1.49E-10 |
| rs151291132 | 15 | 44842210 | G | A | 0.023881 | 198529 | 0.061164 | 0.010294 | 2.82E-09 |
| rs77303550 | 16 | 72079657 | T | C | 0.191792 | 198953 | 0.160903 | 0.003967 | 3.19E-358 |
| rs8044476 | 16 | 72800567 | G | A | 0.148869 | 199732 | -0.07284 | 0.004398 | 1.42E-61 |
| rs9935618 | 16 | 71414059 | T | G | 0.375866 | 196929 | 0.053291 | 0.003256 | 3.64E-60 |
| rs11646091 | 16 | 70825649 | C | T | 0.041688 | 199732 | 0.091918 | 0.007839 | 9.66E-32 |
| rs111849318 | 16 | 72695058 | G | A | 0.043867 | 190282 | 0.072272 | 0.00784 | 3.05E-20 |
| rs1549292 | 16 | 71983664 | G | T | 0.115564 | 199478 | -0.04272 | 0.004902 | 2.93E-18 |
| rs193281563 | 16 | 70646632 | T | C | 0.024936 | 196283 | -0.07404 | 0.010118 | 2.53E-13 |
| rs17241731 | 16 | 69893821 | T | C | 0.0319 | 199732 | -0.06479 | 0.008893 | 3.22E-13 |
| rs139739055 | 16 | 71237119 | A | G | 0.010093 | 198703 | -0.1137 | 0.015673 | 4.05E-13 |
| rs17881236 | 16 | 70514382 | A | G | 0.029383 | 198996 | 0.05734 | 0.009295 | 6.89E-10 |
| rs113754130 | 16 | 70816361 | C | G | 0.012819 | 198840 | 0.085924 | 0.013944 | 7.20E-10 |
| rs2925979 | 16 | 81534790 | T | C | 0.300107 | 199732 | 0.020517 | 0.003418 | 1.93E-09 |
| rs80229424 | 16 | 70384434 | T | C | 0.033112 | 199732 | 0.051977 | 0.008755 | 2.91E-09 |
| rs7190009 | 16 | 72996813 | C | T | 0.035225 | 199732 | 0.049857 | 0.008525 | 4.97E-09 |
| rs35207980 | 16 | 85271686 | A | G | 0.211097 | 195216 | -0.02236 | 0.003891 | 9.09E-09 |
| rs10673343 | 16 | 373776 | CCA | C | 0.227175 | 191438 | -0.02169 | 0.003827 | 1.46E-08 |
| rs140937328 | 16 | 69499981 | CT | C | 0.058438 | 199544 | -0.03684 | 0.006685 | 3.59E-08 |
| rs145947882 | 17 | 41809207 | C | A | 0.024516 | 198487 | 0.068045 | 0.010158 | 2.10E-11 |
| rs9303533 | 17 | 45386540 | G | A | 0.483432 | 198248 | 0.0205 | 0.003145 | 7.10E-11 |
| rs149394327 | 17 | 64228995 | C | G | 0.029599 | 199448 | -0.05876 | 0.009243 | 2.06E-10 |
| rs34893138 | 17 | 57850763 | AT | A | 0.209492 | 196019 | 0.024365 | 0.003883 | 3.51E-10 |
| rs11655056 | 17 | 47364107 | C | T | 0.458735 | 196145 | 0.019684 | 0.003177 | 5.81E-10 |
| rs11078597 | 17 | 1618363 | C | T | 0.185784 | 199732 | 0.024046 | 0.004022 | 2.26E-09 |
| rs78579961 | 17 | 79425141 | T | C | 0.186461 | 194995 | 0.024055 | 0.004074 | 3.55E-09 |
| rs62086903 | 17 | 66016006 | C | T | 0.228891 | 195425 | 0.021884 | 0.003771 | 6.53E-09 |
| 17:17982339_CA_C | 17 | 17982339 | C | CA | 0.299917 | 191943 | 0.020148 | 0.003485 | 7.42E-09 |
| rs59774409 | 19 | 50016748 | T | C | 0.081851 | 199631 | 0.068987 | 0.005707 | 1.25E-33 |
| rs58542926 | 19 | 19379549 | T | C | 0.07452 | 199732 | -0.05819 | 0.005971 | 1.96E-22 |
| rs116843064 | 19 | 8429323 | A | G | 0.019138 | 199732 | -0.10394 | 0.011424 | 9.30E-20 |
| rs141622900 | 19 | 45426792 | A | G | 0.0488 | 195944 | 0.054963 | 0.007335 | 6.76E-14 |
| rs10405357 | 19 | 54759666 | C | T | 0.438815 | 195417 | 0.022655 | 0.003185 | 1.14E-12 |
| rs12609794 | 19 | 35555585 | G | A | 0.25207 | 199732 | -0.0234 | 0.003605 | 8.58E-11 |
| rs2779165 | 19 | 4915447 | G | C | 0.182149 | 196180 | -0.02648 | 0.004098 | 1.05E-10 |
| rs12459419 | 19 | 51728477 | T | C | 0.323674 | 199732 | -0.02055 | 0.003345 | 8.03E-10 |
| rs7250869 | 19 | 33887405 | T | C | 0.310782 | 199238 | 0.019723 | 0.003388 | 5.85E-09 |
| rs56113850 | 19 | 41353107 | T | C | 0.421531 | 198085 | -0.01767 | 0.003177 | 2.64E-08 |
| rs516246 | 19 | 49206172 | C | T | 0.492855 | 199732 | -0.01726 | 0.003145 | 4.10E-08 |
| rs6065904 | 20 | 44534651 | A | G | 0.212492 | 199732 | 0.02601 | 0.003826 | 1.06E-11 |
| rs13036892 | 20 | 38917695 | G | T | 0.106653 | 199506 | -0.02928 | 0.005075 | 7.96E-09 |
| rs4297946 | 20 | 39811275 | C | G | 0.467981 | 198336 | 0.017285 | 0.003148 | 4.01E-08 |
| rs62222988 | 21 | 40569508 | C | T | 0.374725 | 198284 | 0.018551 | 0.003251 | 1.15E-08 |
| rs738409 | 22 | 44324727 | G | C | 0.217169 | 199732 | -0.03242 | 0.003795 | 1.30E-17 |
| rs9618180 | 22 | 18479072 | C | T | 0.251285 | 196317 | -0.02094 | 0.00364 | 8.76E-09 |

Abbreviations: Chr., chromosome; EAF, effect allele frequency; LD, linkage disequilibrium; SE, standard error.

^a^ Linear regression model was used to obtain effect estimates and SEs, adjusting for age, sex, genotyping arrays, and the first ten principal components for population structure. LD clumping was then performed to identify independently associated variants (*r*^2^ <1×10^-3^ in a 500kb window).

**Supplementary Table S17.** Summary of the 32 independent, genome-wide significant (*P*-value <5×10^-8^) variants associated with standardized levels of log-transformed 3-hydroxybutyrate (mmol/L), the UK Biobank cohort^a^

| **Variant** | **Chr.** | **Position (GRCh37)** | **Effect allele** | **Other allele** | **EAF** | ***N*** | **β** | **SE** | ***P*-value** |
| --- | --- | --- | --- | --- | --- | --- | --- | --- | --- |
| rs59484402 | 1 | 230302838 | C | CT | 0.388763 | 190552 | 0.028938 | 0.003291 | 1.46E-18 |
| rs796734259 | 1 | 220973760 | A | ACAAG | 0.317486 | 191402 | 0.020209 | 0.003444 | 4.40E-09 |
| rs200299301 | 4 | 3470056 | A | AGT | 0.261626 | 196127 | 0.021984 | 0.003614 | 1.18E-09 |
| rs2910628 | 5 | 41659364 | T | A | 0.242469 | 199083 | -0.03531 | 0.003676 | 7.75E-22 |
| rs905095 | 5 | 42168124 | G | A | 0.33804 | 194332 | -0.02055 | 0.003359 | 9.52E-10 |
| rs72757003 | 5 | 42742377 | T | A | 0.054241 | 197083 | -0.04014 | 0.006962 | 8.17E-09 |
| rs71552099 | 6 | 31943418 | A | AT | 0.334789 | 196637 | 0.018378 | 0.003367 | 4.80E-08 |
| rs2126259 | 8 | 9185146 | T | C | 0.101283 | 199732 | -0.05533 | 0.005211 | 2.47E-26 |
| rs1736060 | 8 | 11664738 | C | T | 0.417241 | 199393 | 0.022101 | 0.003187 | 4.07E-12 |
| 8:10821056_CT_C | 8 | 10821056 | CT | C | 0.465665 | 194130 | -0.0211 | 0.003199 | 4.27E-11 |
| rs7460226 | 8 | 10197718 | G | A | 0.408263 | 194746 | -0.01899 | 0.003236 | 4.36E-09 |
| rs7005904 | 8 | 8218597 | T | C | 0.496305 | 194159 | 0.018743 | 0.003196 | 4.49E-09 |
| rs2954021 | 8 | 126482077 | A | G | 0.495722 | 199615 | -0.01838 | 0.003145 | 5.14E-09 |
| rs72691630 | 8 | 144297787 | A | G | 0.184037 | 199384 | -0.02278 | 0.004053 | 1.92E-08 |
| rs1883025 | 9 | 107664301 | T | C | 0.254433 | 199732 | -0.02868 | 0.003612 | 2.03E-15 |
| rs4149310 | 9 | 107589134 | T | A | 0.156355 | 199732 | 0.030138 | 0.004321 | 3.07E-12 |
| rs2297991 | 10 | 113913222 | T | C | 0.281412 | 199732 | -0.03548 | 0.003499 | 3.72E-24 |
| 11:116623213_TA_T | 11 | 116623213 | TA | T | 0.14129 | 199143 | 0.036973 | 0.004511 | 2.50E-16 |
| rs499974 | 11 | 75455021 | A | C | 0.15676 | 199732 | 0.030211 | 0.00433 | 3.02E-12 |
| rs73228032 | 12 | 123322415 | A | C | 0.142786 | 199732 | -0.02907 | 0.004501 | 1.06E-10 |
| rs1169288 | 12 | 121416650 | C | A | 0.313808 | 196314 | -0.02118 | 0.003425 | 6.25E-10 |
| rs11058058 | 12 | 122635983 | A | G | 0.168023 | 198041 | -0.02379 | 0.004218 | 1.70E-08 |
| rs28929474 | 14 | 94844947 | T | C | 0.019569 | 199732 | -0.11456 | 0.011368 | 7.03E-24 |
| rs145730801 | 14 | 94768196 | C | T | 0.040922 | 197033 | -0.04936 | 0.008007 | 7.07E-10 |
| rs10468017 | 15 | 58678512 | T | C | 0.295023 | 199732 | -0.02883 | 0.003448 | 6.12E-17 |
| rs588136 | 15 | 58730498 | C | T | 0.209923 | 197275 | -0.02767 | 0.003885 | 1.06E-12 |
| rs11075921 | 16 | 72132129 | T | C | 0.159124 | 199212 | 0.048689 | 0.004297 | 9.35E-30 |
| rs9939224 | 16 | 57002732 | T | G | 0.20913 | 199732 | -0.02621 | 0.003865 | 1.20E-11 |
| rs56177707 | 16 | 72877335 | A | G | 0.055418 | 195714 | 0.043323 | 0.006941 | 4.35E-10 |
| rs117643180 | 17 | 7185779 | A | C | 0.025184 | 199194 | -0.08741 | 0.010018 | 2.67E-18 |
| rs429358 | 19 | 45411941 | C | T | 0.155085 | 199732 | 0.052987 | 0.004345 | 3.36E-34 |
| rs11672660 | 19 | 46180184 | T | C | 0.193872 | 199696 | -0.02226 | 0.003972 | 2.08E-08 |

Abbreviations: Chr., chromosome; EAF: effect allele frequency; LD, linkage disequilibrium; SE, standard error.

^a^ Linear regression model was used to obtain effect estimates and SEs, adjusting for age, sex, genotyping arrays, and the first ten principal components for population structure. LD clumping was then performed to identify independently associated variants (*r*^2^ <1×10^-3^ in a 500kb window).

**Supplementary Table S18.** Summary of the 64 independent, genome-wide significant (*P*-value <5×10^-8^) variants associated with standardized levels of cholesterol relative to total lipids in small LDL, the UK Biobank cohort^a^

| **Variant** | **Chr.** | **Position (GRCh37)** | **Effect allele** | **Other allele** | **EAF** | ***N*** | **β** | **SE** | ***P*-value** | |
| --- | --- | --- | --- | --- | --- | --- | --- | --- | --- | --- |
| rs11591147 | 1 | 55505647 | T | G | 0.017178 | 199,732 | -0.15226 | 0.012111 | 3.11E-36 |  |
| rs3832016 | 1 | 109818158 | C | CT | 0.215015 | 199,644 | -0.03587 | 0.003826 | 6.97E-21 |  |
| rs472495 | 1 | 55521313 | G | T | 0.349945 | 199,257 | -0.02373 | 0.003298 | 6.17E-13 |  |
| rs59484402 | 1 | 230302838 | C | CT | 0.388763 | 190,552 | -0.02317 | 0.003295 | 2.04E-12 |  |
| rs3768321 | 1 | 40035928 | T | G | 0.196947 | 199,064 | -0.02764 | 0.003961 | 2.97E-12 |  |
| rs2642438 | 1 | 220970028 | A | G | 0.29697 | 199,732 | -0.02244 | 0.003441 | 6.91E-11 |  |
| rs4656292 | 1 | 161194641 | A | G | 0.37946 | 195,649 | -0.02101 | 0.00327 | 1.34E-10 |  |
| rs321236 | 1 | 96478860 | A | C | 0.362329 | 196,952 | 0.019166 | 0.003297 | 6.13E-09 |  |
| rs1367117 | 2 | 21263900 | A | G | 0.336721 | 199,732 | 0.03084 | 0.003323 | 1.69E-20 |  |
| rs1047891 | 2 | 211540507 | A | C | 0.316419 | 199,732 | -0.02282 | 0.003383 | 1.52E-11 |  |
| rs4299376 | 2 | 44072576 | G | T | 0.322673 | 199,205 | 0.022666 | 0.003367 | 1.69E-11 |  |
| rs7578326 | 2 | 227020653 | G | A | 0.33947 | 199,732 | 0.021102 | 0.003321 | 2.11E-10 |  |
| rs1154988 | 3 | 135925191 | T | A | 0.226272 | 199,636 | 0.021089 | 0.003761 | 2.05E-08 |  |
| rs763938651 | 5 | 74639544 | C | CTTGTA | 0.363505 | 196,904 | 0.030671 | 0.003292 | 1.20E-20 |  |
| rs74186130 | 6 | 32632859 | G | C | 0.141763 | 196,674 | 0.029644 | 0.004541 | 6.65E-11 |  |
| rs79220007 | 6 | 26098474 | C | T | 0.076642 | 199,499 | -0.0325 | 0.005916 | 3.95E-08 |  |
| rs62466318 | 7 | 73042085 | T | C | 0.201809 | 198,086 | 0.025285 | 0.003943 | 1.44E-10 |  |
| rs217381 | 7 | 44606217 | G | C | 0.425779 | 199,600 | -0.01917 | 0.003176 | 1.58E-09 |  |
| rs4719925 | 7 | 28188995 | G | A | 0.084774 | 199,732 | 0.033477 | 0.005644 | 3.00E-09 |  |
| rs328 | 8 | 19819724 | G | C | 0.099911 | 199,732 | 0.062305 | 0.005242 | 1.42E-32 |  |
| rs4240624 | 8 | 9184231 | G | A | 0.090918 | 199,659 | -0.05558 | 0.005478 | 3.44E-24 |  |
| rs139894090 | 8 | 19771510 | A | G | 0.012112 | 197,947 | -0.09673 | 0.014429 | 2.04E-11 |  |
| rs2980867 | 8 | 126487691 | T | G | 0.300885 | 199,706 | -0.0216 | 0.00343 | 3.04E-10 |  |
| rs532436 | 9 | 136149830 | A | G | 0.18429 | 199,693 | 0.06324 | 0.004063 | 1.37E-54 |  |
| rs4149310 | 9 | 107589134 | T | A | 0.156355 | 199,732 | 0.028139 | 0.004325 | 7.72E-11 |  |
| rs13284054 | 9 | 107669073 | C | T | 0.117793 | 193,870 | -0.02926 | 0.004961 | 3.70E-09 |  |
| rs11146021 | 9 | 139327858 | A | G | 0.287095 | 199,162 | -0.02037 | 0.003477 | 4.65E-09 |  |
| rs7896518 | 10 | 65104500 | G | A | 0.431597 | 189,831 | 0.028063 | 0.003263 | 8.07E-18 |  |
| rs796921021 | 10 | 52183063 | T | TCC | 0.273628 | 198,258 | -0.02044 | 0.003545 | 8.23E-09 |  |
| rs2792751 | 10 | 113940329 | T | C | 0.275289 | 199,732 | 0.019636 | 0.003527 | 2.59E-08 |  |
| rs964184 | 11 | 116648917 | G | C | 0.133884 | 199,732 | -0.04152 | 0.004617 | 2.41E-19 |  |
| rs10750766 | 11 | 65473798 | C | A | 0.288669 | 198,617 | 0.024089 | 0.00348 | 4.47E-12 |  |
| rs1219549 | 11 | 75454622 | C | T | 0.157195 | 199,640 | -0.02709 | 0.00433 | 3.98E-10 |  |
| rs76970536 | 11 | 126250680 | A | G | 0.070954 | 199,008 | 0.03799 | 0.006127 | 5.64E-10 |  |
| rs2923099 | 11 | 10369340 | A | C | 0.414336 | 197,451 | 0.018913 | 0.00321 | 3.83E-09 |  |
| rs28883710 | 12 | 53506096 | C | T | 0.154338 | 199,446 | 0.029752 | 0.004356 | 8.54E-12 |  |
| rs1045853 | 14 | 106208086 | A | C | 0.337671 | 195,147 | 0.026388 | 0.003372 | 5.09E-15 |  |
| rs2494747 | 14 | 105258437 | G | T | 0.383585 | 197,763 | 0.019257 | 0.003255 | 3.30E-09 |  |
| rs2070895 | 15 | 58723939 | A | G | 0.21715 | 199,127 | -0.10152 | 0.003815 | 8.90E-156 |  |
| rs261290 | 15 | 58678720 | T | C | 0.344377 | 199,081 | -0.08659 | 0.003312 | 2.30E-150 |  |
| rs147233090 | 15 | 44028047 | T | C | 0.023983 | 199,308 | -0.08972 | 0.010295 | 2.92E-18 |  |
| rs34890778 | 15 | 58549978 | G | A | 0.497989 | 196,217 | 0.02164 | 0.003172 | 8.95E-12 |  |
| rs143875230 | 15 | 43278726 | A | G | 0.023723 | 198,566 | -0.06868 | 0.010361 | 3.39E-11 |  |
| rs144972973 | 15 | 44564692 | G | A | 0.024372 | 198,961 | -0.0673 | 0.010218 | 4.50E-11 |  |
| rs6078 | 15 | 58833993 | A | G | 0.02822 | 199,732 | -0.05236 | 0.009507 | 3.63E-08 |  |
| rs821840 | 16 | 56993886 | G | A | 0.323526 | 199,378 | -0.07784 | 0.003365 | 3.20E-118 |  |
| rs1566455 | 16 | 69900164 | G | T | 0.421854 | 199,255 | 0.017798 | 0.003188 | 2.38E-08 |  |
| rs7219625 | 17 | 76390080 | T | C | 0.496084 | 197,029 | 0.023724 | 0.003168 | 6.99E-14 |  |
| rs62061425 | 17 | 7073091 | G | A | 0.20058 | 198,629 | -0.02566 | 0.003938 | 7.29E-11 |  |
| rs77542162 | 17 | 67081278 | G | A | 0.02264 | 199,732 | 0.061958 | 0.010578 | 4.72E-09 |  |
| rs77960347 | 18 | 47109955 | G | A | 0.013638 | 199,732 | 0.129462 | 0.01355 | 1.25E-21 |  |
| rs4939883 | 18 | 47167214 | T | C | 0.179208 | 199,732 | -0.0369 | 0.004101 | 2.32E-19 |  |
| rs74489351 | 18 | 46578242 | A | C | 0.010465 | 199,140 | 0.13611 | 0.015473 | 1.42E-18 |  |
| rs1065853 | 19 | 45413233 | T | G | 0.080406 | 199,482 | -0.17003 | 0.005776 | 4.40E-190 |  |
| 19:11196356_AC_A | 19 | 11196356 | A | AC | 0.11878 | 199,461 | -0.0744 | 0.004856 | 5.96E-53 |  |
| rs58542926 | 19 | 19379549 | T | C | 0.07452 | 199,732 | -0.05412 | 0.005997 | 1.82E-19 |  |
| rs367070 | 19 | 54800500 | G | A | 0.22466 | 197,545 | 0.032951 | 0.003802 | 4.46E-18 |  |
| rs116843064 | 19 | 8429323 | A | G | 0.019138 | 199,732 | 0.078791 | 0.011475 | 6.60E-12 |  |
| rs3810291 | 19 | 47569003 | G | A | 0.32439 | 199,732 | 0.019122 | 0.003356 | 1.21E-08 |  |
| rs188247550 | 19 | 19396616 | T | C | 0.011246 | 197,894 | -0.08226 | 0.014985 | 4.04E-08 |  |
| rs111602331 | 20 | 44557474 | C | T | 0.184008 | 199,524 | 0.081144 | 0.004058 | 7.18E-89 |  |
| rs1800961 | 20 | 43042364 | T | C | 0.030811 | 199,732 | -0.06393 | 0.009095 | 2.08E-12 |  |
| rs364585 | 20 | 12962718 | A | G | 0.390774 | 199,732 | -0.02085 | 0.003228 | 1.07E-10 |  |
| rs117113213 | 20 | 39165692 | A | G | 0.03199 | 198,812 | 0.0528 | 0.008966 | 3.89E-09 |  |

Abbreviations: Chr., chromosome; EAF, effect allele frequency; LD, linkage disequilibrium; LDL, low-density lipoprotein; SE, standard error.

^a^ Linear regression model was used to obtain effect estimates and SEs, adjusting for age, sex, genotyping arrays, and the first ten principal components for population structure. LD clumping was then performed to identify independently associated variants (*r*^2^ <1×10^-3^ in a 500kb window).

**Supplementary Table S19.** Summary of the 186 independent, genome-wide significant (*P*-value <5×10^-8^) variants associated with standardized levels of log-transformed triglycerides relative to total lipids in IDL, the UK Biobank cohort^a^

| **Variant** | **Chr.** | **Position (GRCh37)** | **Effect allele** | **Other allele** | **EAF** | ***N*** | **β** | **SE** | ***P*-value** |
| --- | --- | --- | --- | --- | --- | --- | --- | --- | --- |
| rs193084249 | 1 | 26987646 | G | A | 0.0223444 | 198596 | 0.0752617 | 0.010382 | 4.21E-13 |
| rs72663503 | 1 | 39969059 | T | C | 0.228885 | 198689 | 0.0324166 | 0.00364526 | 6.01E-19 |
| 1:51216822_TA_T | 1 | 51216822 | T | TA | 0.364575 | 195333 | -0.0212467 | 0.00321035 | 3.65E-11 |
| rs11591147 | 1 | 55505647 | T | G | 0.017178 | 199732 | 0.190458 | 0.0117606 | 6.01E-59 |
| rs472495 | 1 | 55521313 | G | T | 0.349945 | 199257 | 0.0278649 | 0.00320279 | 3.34E-18 |
| 1:62906034_TTA_T | 1 | 62906034 | T | TTA | 0.354332 | 197350 | -0.0366736 | 0.00321753 | 4.37E-30 |
| rs10874778 | 1 | 93866169 | G | A | 0.433835 | 197143 | -0.0179244 | 0.003105 | 7.81E-09 |
| rs7528419 | 1 | 109817192 | G | A | 0.222869 | 199732 | 0.0448327 | 0.00367111 | 2.75E-34 |
| rs587685973 | 1 | 149940804 | AT | A | 0.0824726 | 194610 | -0.0313523 | 0.00562043 | 2.43E-08 |
| 1:154556761_CCT_C | 1 | 154556761 | C | CCT | 0.299937 | 197658 | -0.0192245 | 0.00334601 | 9.18E-09 |
| rs2820426 | 1 | 219660535 | A | G | 0.394678 | 196587 | -0.0178562 | 0.00314941 | 1.43E-08 |
| rs2642438 | 1 | 220970028 | A | G | 0.29697 | 199732 | 0.0321449 | 0.00334152 | 6.67E-22 |
| rs2281721 | 1 | 230297136 | C | T | 0.387329 | 198986 | 0.0401712 | 0.00313787 | 1.65E-37 |
| rs4665710 | 2 | 21221035 | A | C | 0.204003 | 199019 | -0.0745779 | 0.0037883 | 3.41E-86 |
| rs11689541 | 2 | 21372547 | C | T | 0.0613407 | 198457 | 0.0397939 | 0.00637879 | 4.43E-10 |
| rs6714780 | 2 | 27189063 | A | G | 0.0494939 | 194903 | 0.0615379 | 0.00715585 | 8.05E-18 |
| rs1260326 | 2 | 27730940 | T | C | 0.395933 | 199732 | 0.0884531 | 0.00311988 | 1.80E-176 |
| rs62140395 | 2 | 28244926 | C | G | 0.116709 | 199732 | 0.0532327 | 0.00475417 | 4.30E-29 |
| rs4245791 | 2 | 44074431 | C | T | 0.324653 | 199732 | -0.0268781 | 0.00326042 | 1.68E-16 |
| rs6708784 | 2 | 111927379 | G | A | 0.494897 | 198800 | -0.0182273 | 0.00306354 | 2.69E-09 |
| rs12692735 | 2 | 165504565 | T | G | 0.359486 | 199329 | -0.0286977 | 0.00318319 | 1.98E-19 |
| rs148358468 | 2 | 219590348 | A | G | 0.0495169 | 199033 | 0.039703 | 0.00705887 | 1.86E-08 |
| rs2972156 | 2 | 227117778 | C | G | 0.351861 | 198276 | -0.0420573 | 0.00320476 | 2.51E-39 |
| rs117350179 | 3 | 12374332 | G | C | 0.0257585 | 199468 | 0.0537161 | 0.00965527 | 2.65E-08 |
| rs6809248 | 3 | 52309338 | G | A | 0.130152 | 199525 | -0.0291691 | 0.00453619 | 1.28E-10 |
| rs684773 | 3 | 135956305 | A | C | 0.232557 | 199732 | -0.0405247 | 0.00361227 | 3.37E-29 |
| rs6439657 | 3 | 136494639 | T | C | 0.255278 | 199336 | -0.0336133 | 0.00350719 | 9.43E-22 |
| rs9844972 | 3 | 150097635 | C | G | 0.0671974 | 195878 | 0.0441139 | 0.00615235 | 7.51E-13 |
| rs9817452 | 3 | 156795414 | T | G | 0.386634 | 198336 | -0.0200082 | 0.00316165 | 2.48E-10 |
| rs3215234 | 3 | 170724091 | GT | G | 0.124774 | 199485 | 0.0349022 | 0.00462698 | 4.61E-14 |
| rs13316580 | 3 | 172262053 | C | T | 0.129913 | 199399 | 0.0249729 | 0.00455689 | 4.25E-08 |
| rs73243877 | 4 | 26047616 | G | A | 0.168638 | 199732 | 0.0250786 | 0.00408573 | 8.37E-10 |
| 4:39682825_CA_C | 4 | 39682825 | CA | C | 0.490952 | 190376 | 0.0197263 | 0.00312464 | 2.74E-10 |
| rs7675258 | 4 | 77413179 | G | A | 0.462178 | 199567 | -0.0183631 | 0.00306632 | 2.12E-09 |
| rs7661844 | 4 | 87468625 | C | T | 0.492756 | 198860 | 0.0199588 | 0.00305896 | 6.83E-11 |
| rs1471251 | 4 | 87976359 | T | A | 0.398271 | 198238 | 0.0255663 | 0.0031359 | 3.58E-16 |
| rs13107325 | 4 | 103188709 | T | C | 0.0750481 | 199732 | 0.0571084 | 0.00580451 | 7.77E-23 |
| rs61335963 | 5 | 55809035 | C | G | 0.0646281 | 199565 | -0.0387468 | 0.00622696 | 4.90E-10 |
| 5:55860907_GC_G | 5 | 55860907 | G | GC | 0.185833 | 199523 | 0.040139 | 0.00392723 | 1.62E-24 |
| rs37538 | 5 | 57610069 | G | C | 0.397381 | 196298 | 0.0175357 | 0.00315084 | 2.62E-08 |
| 5:74276898_CT_C | 5 | 74276898 | CT | C | 0.47019 | 191717 | 0.0200942 | 0.00312272 | 1.24E-10 |
| rs5744552 | 5 | 74816944 | G | C | 0.38431 | 199240 | -0.0396939 | 0.00314273 | 1.48E-36 |
| rs146313134 | 5 | 78433740 | A | C | 0.370941 | 195144 | -0.0240281 | 0.00318601 | 4.66E-14 |
| rs10075841 | 5 | 122668002 | G | A | 0.494941 | 191755 | 0.0171353 | 0.00312338 | 4.11E-08 |
| rs272838 | 5 | 131638817 | T | C | 0.163439 | 195608 | 0.0304696 | 0.00418262 | 3.23E-13 |
| 5:132426851_GTT_G | 5 | 132426851 | G | GTT | 0.0920755 | 199369 | -0.0309634 | 0.0052943 | 4.97E-09 |
| rs1363232 | 5 | 156383422 | A | G | 0.339102 | 198719 | -0.0176208 | 0.0032322 | 5.00E-08 |
| rs2963468 | 5 | 158003020 | G | A | 0.231905 | 195354 | 0.020384 | 0.00365661 | 2.48E-08 |
| rs7773004 | 6 | 26267755 | G | A | 0.495175 | 199388 | -0.0260158 | 0.00305589 | 1.70E-17 |
| rs780222578 | 6 | 28677045 | C | CA | 0.14966 | 191688 | -0.0263532 | 0.00437693 | 1.74E-09 |
| 6:29817617_A_G | 6 | 29817617 | G | A | 0.335068 | 199361 | -0.0244856 | 0.00323519 | 3.79E-14 |
| rs372771873 | 6 | 30348458 | C | CAT | 0.142889 | 199245 | -0.0270224 | 0.00437222 | 6.40E-10 |
| rs35193884 | 6 | 31249611 | A | G | 0.0769061 | 199607 | 0.0574403 | 0.00574609 | 1.60E-23 |
| rs622871 | 6 | 31878495 | A | G | 0.308058 | 196976 | -0.0273227 | 0.00333515 | 2.58E-16 |
| rs28752510 | 6 | 32579626 | A | G | 0.456312 | 198670 | 0.0308715 | 0.0030766 | 1.09E-23 |
| rs9275613 | 6 | 32684105 | T | C | 0.111981 | 199722 | 0.0293178 | 0.00485395 | 1.54E-09 |
| rs998584 | 6 | 43757896 | A | C | 0.482444 | 197653 | 0.0269725 | 0.00307315 | 1.69E-18 |
| rs9370162 | 6 | 52728779 | G | A | 0.42876 | 198133 | 0.0198522 | 0.0031011 | 1.54E-10 |
| rs577721086 | 6 | 127440047 | C | T | 0.0478629 | 198139 | 0.0588421 | 0.00717925 | 2.50E-16 |
| rs199607859 | 6 | 139835418 | G | T | 0.404853 | 197441 | 0.029048 | 0.00312566 | 1.51E-20 |
| rs9457827 | 6 | 160503656 | T | C | 0.0161825 | 198239 | -0.0791792 | 0.0121482 | 7.15E-11 |
| rs571848809 | 6 | 161005388 | A | G | 0.105867 | 199024 | 0.0565276 | 0.00497066 | 5.87E-30 |
| rs79237498 | 7 | 6436865 | CT | C | 0.266286 | 196989 | -0.020119 | 0.00348077 | 7.48E-09 |
| rs38205 | 7 | 15913588 | A | C | 0.37612 | 193405 | 0.0194365 | 0.00320399 | 1.31E-09 |
| rs6461354 | 7 | 17914600 | T | C | 0.398469 | 199057 | 0.0229518 | 0.00312236 | 1.98E-13 |
| rs4722551 | 7 | 25991826 | C | T | 0.157919 | 199732 | -0.0365922 | 0.00418809 | 2.41E-18 |
| rs112577710 | 7 | 44200715 | TTAATTA | T | 0.15292 | 196724 | 0.0252439 | 0.00426716 | 3.31E-09 |
| rs34881399 | 7 | 72234176 | C | T | 0.0308188 | 199732 | -0.0786538 | 0.008838 | 5.66E-19 |
| rs147767686 | 7 | 72389537 | T | C | 0.0257306 | 197702 | -0.0821568 | 0.00970598 | 2.59E-17 |
| rs141136126 | 7 | 72852007 | A | G | 0.0269908 | 199142 | -0.0541845 | 0.00944893 | 9.80E-09 |
| rs13234378 | 7 | 73026151 | T | A | 0.128125 | 199668 | -0.121587 | 0.00456442 | 4.64E-156 |
| rs3177697 | 7 | 100804542 | T | G | 0.239297 | 199518 | 0.0213382 | 0.00357926 | 2.50E-09 |
| 7:130451984_CTTTA_C | 7 | 130451984 | C | CTTTA | 0.487994 | 197701 | -0.0259231 | 0.00307399 | 3.39E-17 |
| rs2980755 | 8 | 8363683 | G | A | 0.457295 | 193758 | -0.0217912 | 0.00311802 | 2.78E-12 |
| rs7012814 | 8 | 9173358 | A | G | 0.472908 | 195059 | -0.0373209 | 0.00310037 | 2.32E-33 |
| rs36102779 | 8 | 9749609 | G | A | 0.305853 | 196967 | -0.0227896 | 0.00333245 | 8.02E-12 |
| rs7821812 | 8 | 10644101 | C | G | 0.207065 | 199350 | 0.0309391 | 0.00377461 | 2.49E-16 |
| rs13280055 | 8 | 11522353 | A | G | 0.126394 | 191595 | 0.0353876 | 0.00468656 | 4.34E-14 |
| rs35246381 | 8 | 18272535 | C | T | 0.221226 | 199563 | 0.0238986 | 0.00367883 | 8.25E-11 |
| rs59347135 | 8 | 19750044 | G | C | 0.041798 | 196002 | 0.0728837 | 0.00771101 | 3.36E-21 |
| rs328 | 8 | 19819724 | G | C | 0.0999114 | 199732 | -0.17552 | 0.00507785 | 4.91E-261 |
| rs143298923 | 8 | 19919713 | A | T | 0.010852 | 199549 | -0.0812478 | 0.0147541 | 3.66E-08 |
| rs117174179 | 8 | 19941078 | T | G | 0.0191311 | 199257 | 0.144913 | 0.0111623 | 1.60E-38 |
| rs17092008 | 8 | 19961274 | T | C | 0.0624167 | 198889 | 0.0357322 | 0.00633109 | 1.66E-08 |
| rs117452590 | 8 | 41688190 | A | G | 0.0142441 | 199732 | -0.0721336 | 0.0129124 | 2.32E-08 |
| rs28446899 | 8 | 72396213 | T | C | 0.076018 | 199124 | 0.0333994 | 0.00577274 | 7.23E-09 |
| rs1574542 | 8 | 117122598 | A | G | 0.381818 | 198787 | -0.0172698 | 0.00315208 | 4.29E-08 |
| rs2980888 | 8 | 126507308 | T | C | 0.302833 | 199572 | 0.0644196 | 0.0033245 | 1.43E-83 |
| rs72724627 | 8 | 126635162 | G | A | 0.156655 | 199732 | 0.0281222 | 0.00419816 | 2.11E-11 |
| rs796004 | 9 | 86594798 | T | C | 0.255821 | 199593 | -0.0204653 | 0.00350467 | 5.25E-09 |
| rs782134971 | 9 | 136139907 | G | GAAACTGCC | 0.253407 | 199205 | -0.0266725 | 0.00352419 | 3.79E-14 |
| rs3841602 | 10 | 65225899 | AGGCGGC | A | 0.470967 | 198410 | -0.0429857 | 0.00307177 | 1.79E-44 |
| rs538931786 | 10 | 93729719 | G | GA | 0.363834 | 197502 | -0.0188224 | 0.00319573 | 3.87E-09 |
| rs4418728 | 10 | 94839724 | T | G | 0.450046 | 199505 | -0.0234184 | 0.00306781 | 2.29E-14 |
| rs2803619 | 10 | 113934384 | G | C | 0.275634 | 199654 | -0.0329709 | 0.0034249 | 6.23E-22 |
| rs7074440 | 10 | 114785424 | A | G | 0.298936 | 198521 | 0.0207824 | 0.00333653 | 4.71E-10 |
| rs11030105 | 11 | 27690566 | G | T | 0.262011 | 199455 | 0.021507 | 0.00348159 | 6.53E-10 |
| rs4603265 | 11 | 46708196 | T | C | 0.13745 | 199450 | -0.0378141 | 0.00444207 | 1.71E-17 |
| rs78290566 | 11 | 47249919 | CG | C | 0.155386 | 198567 | -0.0332813 | 0.00422875 | 3.56E-15 |
| rs113253936 | 11 | 48160609 | T | C | 0.113728 | 192973 | -0.02701 | 0.00488808 | 3.29E-08 |
| rs11229003 | 11 | 57108291 | A | G | 0.11494 | 199182 | 0.0339822 | 0.00480653 | 1.55E-12 |
| rs102275 | 11 | 61557803 | C | T | 0.353651 | 199732 | 0.0832502 | 0.0031904 | 7.62E-150 |
| rs11605489 | 11 | 62472357 | G | A | 0.365168 | 191260 | 0.0189462 | 0.00324373 | 5.20E-09 |
| rs35169799 | 11 | 64031241 | T | C | 0.0634826 | 199732 | 0.0482056 | 0.00626082 | 1.37E-14 |
| rs10750766 | 11 | 65473798 | C | A | 0.288669 | 198617 | -0.0314942 | 0.00337985 | 1.19E-20 |
| rs673335 | 11 | 75450576 | C | T | 0.1585 | 199318 | 0.0288926 | 0.00419528 | 5.72E-12 |
| rs117794084 | 11 | 116433496 | T | G | 0.0163269 | 199732 | 0.117088 | 0.0120358 | 2.31E-22 |
| 11:116623213_TA_T | 11 | 116623213 | TA | T | 0.14129 | 199143 | 0.181786 | 0.0043676 | 5.48E-377 |
| rs12721043 | 11 | 116692293 | A | C | 0.0103939 | 199732 | -0.140889 | 0.0150803 | 9.49E-21 |
| rs76604009 | 11 | 117085261 | T | C | 0.0353961 | 199584 | -0.0470724 | 0.00827798 | 1.30E-08 |
| rs116987336 | 11 | 117175658 | A | G | 0.0264705 | 199732 | 0.14553 | 0.00948887 | 4.64E-53 |
| rs11045172 | 12 | 20470221 | C | A | 0.196462 | 197369 | -0.0244396 | 0.0038666 | 2.61E-10 |
| rs58310495 | 12 | 21357711 | T | C | 0.150472 | 199193 | 0.0330993 | 0.00427702 | 1.01E-14 |
| rs2229357 | 12 | 57843711 | A | G | 0.241521 | 199732 | -0.0262024 | 0.00356993 | 2.15E-13 |
| rs4378452 | 12 | 111504033 | C | T | 0.351854 | 199732 | 0.0182655 | 0.00319978 | 1.14E-08 |
| rs653178 | 12 | 112007756 | C | T | 0.484657 | 199732 | 0.0296067 | 0.00305792 | 3.64E-22 |
| rs17630235 | 12 | 112591686 | A | G | 0.409619 | 199732 | 0.0252964 | 0.00311118 | 4.29E-16 |
| 12:113176003_AT_A | 12 | 113176003 | AT | A | 0.441492 | 198837 | 0.0208258 | 0.00308643 | 1.51E-11 |
| rs3922628 | 12 | 123209295 | A | T | 0.208426 | 196873 | -0.0247762 | 0.00378278 | 5.78E-11 |
| rs10773000 | 12 | 123736084 | T | G | 0.331877 | 199732 | -0.0186169 | 0.00325192 | 1.04E-08 |
| rs7133378 | 12 | 124409502 | A | G | 0.318511 | 198522 | -0.0224782 | 0.00328815 | 8.16E-12 |
| rs41284486 | 13 | 114623821 | A | G | 0.104431 | 193831 | -0.0316931 | 0.0050959 | 5.00E-10 |
| rs774893527 | 14 | 64232517 | T | TAA | 0.159644 | 199315 | 0.0314962 | 0.00418124 | 4.99E-14 |
| rs61704439 | 15 | 40755812 | CAA | C | 0.0848588 | 196438 | -0.0347584 | 0.00552677 | 3.20E-10 |
| rs17716350 | 15 | 41259910 | G | A | 0.116461 | 197233 | -0.0264804 | 0.00478842 | 3.20E-08 |
| rs7170463 | 15 | 41888918 | G | A | 0.309865 | 198462 | -0.0242154 | 0.00331733 | 2.90E-13 |
| rs184334219 | 15 | 42721845 | A | G | 0.0185352 | 199404 | 0.118272 | 0.0113388 | 1.82E-25 |
| rs143875230 | 15 | 43278726 | A | G | 0.0237226 | 198566 | 0.148649 | 0.010061 | 2.26E-49 |
| rs139974673 | 15 | 44027885 | C | T | 0.0255037 | 199540 | 0.173819 | 0.00969078 | 6.96E-72 |
| rs148489550 | 15 | 44581461 | A | G | 0.0253889 | 199241 | 0.135194 | 0.00971825 | 5.67E-44 |
| rs11854318 | 15 | 58571982 | A | G | 0.272574 | 195923 | -0.050097 | 0.00345919 | 1.66E-47 |
| rs1601934 | 15 | 58671721 | G | A | 0.310619 | 197454 | 0.137014 | 0.00330828 | 2.72E-373 |
| rs35980001 | 15 | 58722590 | GC | G | 0.209401 | 195992 | 0.162346 | 0.00377458 | 2.86E-402 |
| rs6078 | 15 | 58833993 | A | G | 0.0282203 | 199732 | 0.0761885 | 0.0092331 | 1.57E-16 |
| 15:59155043_CTG_C | 15 | 59155043 | C | CTG | 0.0631449 | 198860 | -0.0403055 | 0.00629729 | 1.55E-10 |
| rs4776793 | 15 | 66872114 | T | C | 0.354206 | 199720 | 0.0193071 | 0.00319536 | 1.52E-09 |
| rs7175132 | 15 | 101891508 | G | A | 0.387004 | 198242 | -0.01735 | 0.00315284 | 3.74E-08 |
| rs12918213 | 16 | 1249494 | G | A | 0.462022 | 196944 | 0.0173344 | 0.00308806 | 1.99E-08 |
| rs11644601 | 16 | 15172118 | C | T | 0.296173 | 198884 | -0.0259887 | 0.00335714 | 9.89E-15 |
| rs12720918 | 16 | 56994212 | C | T | 0.282767 | 199258 | 0.0196802 | 0.00339492 | 6.76E-09 |
| rs12720926 | 16 | 56998918 | G | A | 0.430966 | 198084 | -0.0248632 | 0.00310116 | 1.09E-15 |
| rs113563886 | 16 | 67947158 | C | T | 0.115985 | 199121 | -0.0300556 | 0.00477746 | 3.16E-10 |
| rs244418 | 16 | 69622762 | A | G | 0.410122 | 199121 | -0.024815 | 0.00311028 | 1.49E-15 |
| rs2925979 | 16 | 81534790 | T | C | 0.300107 | 199732 | 0.0330814 | 0.00333332 | 3.30E-23 |
| rs34460487 | 17 | 4685228 | A | G | 0.346725 | 197342 | 0.0187294 | 0.00323073 | 6.75E-09 |
| rs12945575 | 17 | 40713071 | T | C | 0.247814 | 198752 | 0.0203401 | 0.00354596 | 9.70E-09 |
| rs72836561 | 17 | 41926126 | T | C | 0.0311593 | 199732 | 0.121194 | 0.00879964 | 3.90E-43 |
| rs75134409 | 17 | 42911697 | C | A | 0.385987 | 194272 | -0.0184635 | 0.00318347 | 6.65E-09 |
| rs12185242 | 17 | 47407071 | C | A | 0.454451 | 198908 | 0.0169578 | 0.00307929 | 3.65E-08 |
| rs77542162 | 17 | 67081278 | G | A | 0.0226403 | 199732 | -0.0938356 | 0.0102736 | 6.68E-20 |
| rs12601079 | 17 | 76400329 | G | A | 0.398427 | 194245 | 0.0256845 | 0.00316919 | 5.33E-16 |
| rs12958584 | 18 | 19632380 | T | C | 0.126209 | 199364 | 0.0292237 | 0.0046071 | 2.26E-10 |
| rs74489351 | 18 | 46578242 | A | C | 0.010465 | 199140 | -0.0912091 | 0.0150285 | 1.29E-09 |
| rs77960347 | 18 | 47109955 | G | A | 0.0136383 | 199732 | -0.0838995 | 0.0131629 | 1.85E-10 |
| rs12966550 | 18 | 57911330 | G | A | 0.261562 | 196477 | 0.0213922 | 0.00349982 | 9.83E-10 |
| rs34232444 | 19 | 4965404 | C | T | 0.346256 | 199104 | -0.0194513 | 0.0032231 | 1.59E-09 |
| rs4804833 | 19 | 7970635 | A | G | 0.383759 | 199732 | 0.0173012 | 0.0031333 | 3.36E-08 |
| rs116843064 | 19 | 8429323 | A | G | 0.0191381 | 199732 | -0.245703 | 0.0111334 | 8.46E-108 |
| rs116483979 | 19 | 8456738 | T | C | 0.0299523 | 198833 | -0.0507976 | 0.00899637 | 1.64E-08 |
| rs10412048 | 19 | 11193949 | G | A | 0.119929 | 199448 | 0.0906362 | 0.00469548 | 6.05E-83 |
| rs2738447 | 19 | 11227480 | A | C | 0.407074 | 199292 | 0.0207805 | 0.00311561 | 2.57E-11 |
| rs8112221 | 19 | 16094321 | A | T | 0.30353 | 192841 | -0.0186952 | 0.0033813 | 3.23E-08 |
| rs62102718 | 19 | 33891013 | T | A | 0.285057 | 198246 | 0.0264437 | 0.00339699 | 7.03E-15 |
| rs62115559 | 19 | 44649737 | G | A | 0.0201278 | 198730 | 0.119329 | 0.0109419 | 1.10E-27 |
| rs185920692 | 19 | 44718619 | T | C | 0.0136858 | 199696 | 0.0900434 | 0.0131629 | 7.90E-12 |
| rs62117160 | 19 | 45232161 | A | G | 0.0453182 | 199732 | 0.324978 | 0.00730038 | 1.19E-430 |
| rs112450640 | 19 | 45296364 | A | G | 0.0193215 | 199338 | 0.424904 | 0.0110718 | 4.79E-321 |
| rs41290102 | 19 | 45371188 | T | C | 0.0136308 | 199732 | -0.0718778 | 0.0131676 | 4.80E-08 |
| rs79701229 | 19 | 45384931 | A | G | 0.0129617 | 198933 | -0.08645 | 0.0135155 | 1.59E-10 |
| rs12691088 | 19 | 45418486 | A | G | 0.0147156 | 194929 | -0.104756 | 0.0128299 | 3.23E-16 |
| rs140480140 | 19 | 45421650 | A | G | 0.0279737 | 199080 | -0.0690616 | 0.0092777 | 9.82E-14 |
| rs62117257 | 19 | 45550013 | T | C | 0.0781621 | 199163 | -0.0343881 | 0.00569619 | 1.57E-09 |
| rs17875609 | 19 | 45820144 | T | C | 0.0212385 | 197213 | 0.0961149 | 0.0106706 | 2.13E-19 |
| rs540662190 | 19 | 45913250 | T | C | 0.0130327 | 197542 | 0.197952 | 0.0135501 | 2.61E-48 |
| rs150262789 | 19 | 46436564 | T | C | 0.0143484 | 198071 | 0.0856931 | 0.0129101 | 3.19E-11 |
| rs10408163 | 19 | 47597102 | T | C | 0.286587 | 199732 | -0.0227517 | 0.0033775 | 1.63E-11 |
| rs416867 | 19 | 54796630 | A | G | 0.224094 | 199646 | -0.0247197 | 0.00367749 | 1.80E-11 |
| rs2902745 | 19 | 56093748 | T | G | 0.116695 | 197339 | 0.030933 | 0.00479029 | 1.07E-10 |
| rs686548 | 20 | 12973521 | A | T | 0.391521 | 199661 | 0.0292978 | 0.00313489 | 9.22E-21 |
| rs2618566 | 20 | 17844684 | G | T | 0.33938 | 199732 | -0.0176442 | 0.00322574 | 4.51E-08 |
| rs68051854 | 20 | 32701956 | T | C | 0.426614 | 199316 | -0.0175321 | 0.00309784 | 1.52E-08 |
| rs1800961 | 20 | 43042364 | T | C | 0.0308113 | 199732 | 0.067369 | 0.00883414 | 2.43E-14 |
| rs7679 | 20 | 44576502 | C | T | 0.184294 | 199732 | 0.0675998 | 0.00394301 | 7.74E-66 |
| rs8126001 | 20 | 62711459 | T | C | 0.489137 | 197506 | -0.0186869 | 0.00307116 | 1.17E-09 |
| rs2267373 | 22 | 38600542 | C | T | 0.418923 | 199371 | -0.0245474 | 0.00310209 | 2.52E-15 |
| rs738409 | 22 | 44324727 | G | C | 0.217169 | 199732 | 0.022772 | 0.00370196 | 7.70E-10 |

Abbreviations: Chr., chromosome; EAF, effect allele frequency; LD, linkage disequilibrium; LDL, intermediate-density lipoprotein; SE, standard error.

^a^ Linear regression model was used to obtain effect estimates and SEs, adjusting for age, sex, genotyping arrays, and the first ten principal components for population structure. LD clumping was then performed to identify independently associated variants (*r*^2^ <1×10^-3^ in a 500kb window).

**Supplementary Table S20.** Complete Mendelian randomization analysis results for the selected eight metabolic biomarkers

| **Methods** | **# Variants** | ***F*-statistic** | **OR (95% CI)^a^** | ***P*-value** |
| --- | --- | --- | --- | --- |
| **Log_Triglycerides to Phosphoglycerides (ratio)** | | | | |
| IVW | 146 | 117.3 | 1.04 (1.00-1.09) | 0.03 |
| IVW_strict^b^ | 80 | 184.9 | 1.04 (1.00-1.09) | 0.05 |
| Weighted median |  |  | 1.03 (0.97-1.10) | 0.32 |
| MR-PRESSO |  |  | 1.04 (1.01-1.08) | 0.02 |
| MR-RAPS |  |  | 1.05 (1.01-1.09) | 0.02 |
| MR-Egger |  |  | 1.05 (0.99-1.11) | 0.09 |
| **Linoleic Acid to Total Fatty Acids (%)** | | | | |
| IVW | 75 | 56.4 | 0.89 (0.83-0.96) | 0.003 |
| IVW_strict^b^ | 30 | 89.8 | 0.85 (0.78-0.94) | 0.001 |
| Weighted median |  |  | 0.84 (0.75-0.94) | 0.003 |
| MR-PRESSO |  |  | 0.89 (0.83-0.95) | 0.001 |
| MR-RAPS |  |  | 0.89 (0.82-0.96) | 0.003 |
| MR-Egger |  |  | 0.79 (0.66-0.93) | 0.005 |
| **Saturated Fatty Acids to Total Fatty Acids (%)** | | | | |
| IVW | 32 | 78.5 | 1.14 (1.03-1.25) | 0.009 |
| IVW_strict^b^ | 18 | 112.4 | 1.12 (1.01-1.25) | 0.03 |
| Weighted median |  |  | 1.08 (0.93-1.25) | 0.30 |
| MR-PRESSO |  |  | 1.14 (1.04-1.24) | 0.007 |
| MR-RAPS |  |  | 1.13 (1.02-1.24) | 0.02 |
| MR-Egger |  |  | 1.16 (0.92-1.46) | 0.22 |
| **Omega-6 Fatty Acids to Total Fatty Acids (%)** | | | | |
| IVW | 110 | 82.0 | 0.96 (0.91-1.01) | 0.14 |
| IVW_strict^b^ | 53 | 133.2 | 0.94 (0.89-1.00) | 0.04 |
| Weighted median |  |  | 0.99 (0.90-1.08) | 0.75 |
| MR-PRESSO |  |  | 0.96 (0.91-1.01) | 0.14 |
| MR-RAPS |  |  | 0.96 (0.91-1.01) | 0.10 |
| MR-Egger |  |  | 0.97 (0.89-1.06) | 0.49 |
| **Glycoprotein Acetyls (mmol/L)** | | | | |
| IVW | 97 | 87.5 | 0.97 (0.92-1.02) | 0.21 |
| IVW_strict^b^ | 49 | 138.1 | 0.95 (0.90-1.01) | 0.09 |
| Weighted median |  |  | 1.02 (0.93-1.11) | 0.72 |
| MR-PRESSO |  |  | 0.97 (0.92-1.02) | 0.21 |
| MR-RAPS |  |  | 0.97 (0.91-1.03) | 0.31 |
| MR-Egger |  |  | 0.98 (0.90-1.08) | 0.72 |
| **Log_3-Hydroxybutyrate (mmol/L)** | | | | |
| IVW | 23 | 53.1 | 1.07 (0.93-1.23) | 0.34 |
| IVW_strict^b^ | 11 | 72.5 | 1.21 (1.03-1.44) | 0.02 |
| Weighted median |  |  | 1.08 (0.89-1.32) | 0.42 |
| MR-PRESSO |  |  | 1.07 (0.94-1.21) | 0.30 |
| MR-RAPS |  |  | 1.07 (0.92-1.24) | 0.36 |
| MR-Egger |  |  | 1.84 (1.14-2.96) | 0.01 |
| **Cholesterol to Total Lipids in Small LDL (%)** | | | | |
| IVW | 51 | 113.2 | 1.01 (0.94-1.08) | 0.79 |
| IVW_strict^b^ | 27 | 181.9 | 1.02 (0.96-1.10) | 0.49 |
| Weighted median |  |  | 1.05 (0.95-1.16) | 0.35 |
| MR-PRESSO |  |  | 1.01 (0.95-1.07) | 0.77 |
| MR-RAPS |  |  | 1.00 (0.93-1.07) | 0.96 |
| MR-Egger |  |  | 1.07 (0.96-1.19) | 0.20 |
| **Log_Triglycerides to Total Lipids in IDL (%)** | | | | |
| IVW | 145 | 140.3 | 0.99 (0.95-1.02) | 0.49 |
| IVW_strict^b^ | 88 | 208.5 | 0.99 (0.95-1.02) | 0.49 |
| Weighted median |  |  | 1.01 (0.95-1.07) | 0.83 |
| MR-PRESSO |  |  | 0.99 (0.96-1.02) | 0.48 |
| MR-RAPS |  |  | 1.00 (0.96-1.03) | 0.80 |
| MR-Egger |  |  | 0.99 (0.94-1.04) | 0.55 |

Abbreviations: CI, conference interval; IDL, intermediate-density lipoprotein; IVW, inverse-variance weighting; LDL, low-density lipoprotein; MR, Mendelian randomization; OR, odds ratio; PRESSO, pleiotropy residual sum and outlier; RAPS, robust adjusted profile score.

^a^ Odds ratios (ORs) and 95% confidence intervals (CIs) for 1 standard deviation (SD) increase in biomarker levels were estimated using the IVW approach as the primary analysis. Levels of triglycerides to phosphoglycerides (ratio), 3-hydroxybutyrate (mmol/L), triglycerides to total lipids in IDL (%) were log-transformed before *z*-score standardization.

^b^ In the strict IVW analysis (IVW_strict), a more stringent threshold (*P*-value <5×10^-11^) was used to select genetic instruments.

**Supplementary Table S21**. Summary of 146 genetic variants used to construct the instrumental variables for log-transformed triglycerides to phosphoglycerides ratio in the two-sample Mendelian randomization analyses

| **Variant** | **Chr.** | **Position (GRCh37)** | **Effect allele** | **Other allele** |  | **Log_triglycerides to phosphoglycerides ratio**  **(current study)** | | |  | **Colorectal cancer**  **(Fernandez-Rozadilla, 2023)** | | |
| --- | --- | --- | --- | --- | --- | --- | --- | --- | --- | --- | --- | --- |
|  |  |  |  |  |  | **β** | **SE** | ***P*-value** |  | **β** | **SE** | ***P*-value** |
| rs2275910 | 1 | 7068679 | G | A |  | 0.017005 | 0.003039 | 2.20E-08 |  | 0.004523 | 0.007522 | 0.55 |
| rs193084249 | 1 | 26987646 | G | A |  | 0.106732 | 0.010025 | 1.85E-26 |  | -0.02801 | 0.025412 | 0.27 |
| rs72663503 | 1 | 39969059 | T | C |  | 0.029501 | 0.003521 | 5.38E-17 |  | 0.000798 | 0.008706 | 0.93 |
| rs213479 | 1 | 54861827 | T | G |  | -0.0174 | 0.002985 | 5.59E-09 |  | 0.006524 | 0.013975 | 0.64 |
| rs11207994 | 1 | 63047987 | T | C |  | -0.0482 | 0.003098 | 1.49E-54 |  | -0.00203 | 0.014577 | 0.89 |
| rs10889906 | 1 | 71509107 | G | A |  | 0.017584 | 0.003215 | 4.51E-08 |  | -0.00096 | 0.008007 | 0.90 |
| rs2660302 | 1 | 98520219 | T | A |  | 0.021493 | 0.003824 | 1.90E-08 |  | -0.01397 | 0.009427 | 0.14 |
| rs3754212 | 1 | 150738200 | G | A |  | 0.020992 | 0.003083 | 9.81E-12 |  | -0.0077 | 0.00765 | 0.31 |
| rs58877113^a^ | 1 | 178504590 | A | G |  | -0.01833 | 0.003114 | 4.00E-09 |  | 0.00107 | 0.007691 | 0.89 |
| rs4500327 | 1 | 219705205 | T | C |  | -0.02123 | 0.003121 | 1.04E-11 |  | 0.001624 | 0.00784 | 0.84 |
| rs17649913 | 1 | 220983402 | C | T |  | 0.024548 | 0.0036 | 9.16E-12 |  | 0.006158 | 0.008929 | 0.49 |
| rs2281721 | 1 | 230297136 | C | T |  | 0.051119 | 0.00303 | 7.79E-64 |  | 0.004489 | 0.007537 | 0.55 |
| rs2678379 | 2 | 21226560 | A | G |  | -0.10042 | 0.003658 | 1.25E-165 |  | -0.00388 | 0.008898 | 0.66 |
| rs10193900 | 2 | 25919967 | A | G |  | 0.017557 | 0.003183 | 3.48E-08 |  | 0.004226 | 0.007863 | 0.59 |
| rs115268969 | 2 | 27176094 | T | C |  | 0.067389 | 0.010354 | 7.62E-11 |  | -0.05254 | 0.029036 | 0.07 |
| rs6714780 | 2 | 27189063 | A | G |  | 0.058569 | 0.006913 | 2.41E-17 |  | 0.00755 | 0.018216 | 0.68 |
| rs10180284 | 2 | 50716016 | T | C |  | -0.0166 | 0.002976 | 2.46E-08 |  | -0.00548 | 0.007343 | 0.46 |
| rs1009358 | 2 | 65276452 | C | T |  | -0.01723 | 0.003043 | 1.48E-08 |  | 0.006814 | 0.00756 | 0.37 |
| rs35932591 | 2 | 121310704 | T | C |  | 0.025381 | 0.004517 | 1.92E-08 |  | -0.00644 | 0.011843 | 0.59 |
| rs13389219 | 2 | 165528876 | T | C |  | -0.03975 | 0.00302 | 1.50E-39 |  | -0.00296 | 0.007489 | 0.69 |
| rs4678322 | 3 | 135812523 | T | G |  | -0.02934 | 0.003533 | 1.01E-16 |  | -0.00072 | 0.008899 | 0.94 |
| rs1471741 | 3 | 136328268 | T | C |  | -0.0306 | 0.003377 | 1.32E-19 |  | -0.00202 | 0.008417 | 0.81 |
| rs9844972 | 3 | 150097635 | C | G |  | 0.040697 | 0.005944 | 7.57E-12 |  | -0.00419 | 0.014857 | 0.78 |
| rs9817452 | 3 | 156795414 | T | G |  | -0.02011 | 0.003054 | 4.52E-11 |  | 0.006252 | 0.007576 | 0.41 |
| rs79287178 | 3 | 172294500 | A | G |  | 0.058335 | 0.009476 | 7.48E-10 |  | -0.00569 | 0.022441 | 0.80 |
| rs13101828 | 4 | 965720 | G | A |  | -0.01853 | 0.002985 | 5.39E-10 |  | -0.00317 | 0.007485 | 0.67 |
| rs13108218 | 4 | 3443931 | A | G |  | 0.022916 | 0.003086 | 1.12E-13 |  | -0.01061 | 0.007716 | 0.17 |
| rs4450871 | 4 | 4990298 | G | A |  | -0.01656 | 0.002966 | 2.39E-08 |  | -0.00104 | 0.007995 | 0.90 |
| rs10019888 | 4 | 26062990 | G | A |  | 0.031294 | 0.004011 | 6.10E-15 |  | 0.015818 | 0.009816 | 0.11 |
| rs7661844 | 4 | 87468625 | C | T |  | 0.020698 | 0.002954 | 2.46E-12 |  | 0.011329 | 0.007331 | 0.12 |
| rs1471251 | 4 | 87976359 | T | A |  | 0.027211 | 0.003028 | 2.58E-19 |  | 0.014974 | 0.007515 | 0.05 |
| rs10428504 | 4 | 89716933 | T | G |  | 0.019998 | 0.002966 | 1.57E-11 |  | -0.00966 | 0.007351 | 0.19 |
| rs72754154 | 5 | 55812380 | A | G |  | -0.04991 | 0.006803 | 2.21E-13 |  | 0.007765 | 0.016433 | 0.64 |
| rs9687846 | 5 | 55861894 | A | G |  | 0.052468 | 0.003683 | 4.99E-46 |  | -0.00027 | 0.009286 | 0.98 |
| rs37538 | 5 | 57610069 | G | C |  | 0.017685 | 0.003042 | 6.14E-09 |  | 0.0043 | 0.007531 | 0.57 |
| rs4976033 | 5 | 67714246 | G | A |  | 0.019151 | 0.003087 | 5.54E-10 |  | 0.007263 | 0.007656 | 0.34 |
| rs1533760^a^ | 5 | 78438937 | C | A |  | -0.02039 | 0.003087 | 3.97E-11 |  | 0.006087 | 0.007783 | 0.43 |
| rs1045241 | 5 | 118729286 | T | C |  | -0.02167 | 0.003353 | 1.01E-10 |  | 0.003607 | 0.008175 | 0.66 |
| rs72801474 | 5 | 132444128 | A | G |  | -0.03116 | 0.005101 | 1.01E-09 |  | -0.00074 | 0.013686 | 0.96 |
| rs1501908 | 5 | 156398169 | G | C |  | -0.02795 | 0.003065 | 7.60E-20 |  | -0.00243 | 0.00762 | 0.75 |
| rs1428445 | 5 | 157995803 | A | G |  | 0.02557 | 0.003645 | 2.30E-12 |  | -0.00982 | 0.009013 | 0.28 |
| rs187050905 | 6 | 31975259 | A | G |  | 0.110488 | 0.012036 | 4.35E-20 |  | -0.0272 | 0.057703 | 0.64 |
| rs35820711^a^ | 6 | 32566809 | G | T |  | 0.077374 | 0.007737 | 1.53E-23 |  | -0.01841 | 0.020546 | 0.37 |
| rs998584 | 6 | 43757896 | A | C |  | 0.035815 | 0.002968 | 1.61E-33 |  | 0.005007 | 0.007495 | 0.50 |
| rs4715317 | 6 | 52629010 | T | G |  | 0.018126 | 0.003092 | 4.60E-09 |  | -0.00213 | 0.007679 | 0.78 |
| rs2186037 | 6 | 127455029 | A | G |  | -0.03087 | 0.002953 | 1.41E-25 |  | -0.00597 | 0.007314 | 0.41 |
| rs9492440^a^ | 6 | 130390379 | C | T |  | -0.02438 | 0.003357 | 3.85E-13 |  | -0.01067 | 0.008434 | 0.21 |
| rs635769^a^ | 6 | 139831981 | T | C |  | 0.033059 | 0.003052 | 2.45E-27 |  | -0.00085 | 0.007591 | 0.91 |
| rs9986540 | 6 | 160242487 | T | G |  | -0.14923 | 0.012285 | 6.05E-34 |  | 0.016612 | 0.03176 | 0.60 |
| rs614754 | 6 | 160505199 | C | G |  | -0.08305 | 0.013382 | 5.44E-10 |  | -0.03499 | 0.038006 | 0.36 |
| rs12208357 | 6 | 160543148 | T | C |  | 0.049028 | 0.005816 | 3.50E-17 |  | -0.00591 | 0.014415 | 0.68 |
| rs9295128 | 6 | 160751531 | T | G |  | -0.25697 | 0.012128 | 1.58E-99 |  | 0.013833 | 0.031504 | 0.66 |
| rs10455872 | 6 | 161010118 | G | A |  | -0.14533 | 0.005492 | 4.67E-154 |  | -0.02379 | 0.015166 | 0.12 |
| rs143843429 | 6 | 161383079 | G | A |  | -0.19462 | 0.012983 | 9.07E-51 |  | -0.01603 | 0.031047 | 0.61 |
| rs61735260 | 6 | 161560461 | A | G |  | -0.10518 | 0.013345 | 3.25E-15 |  | -0.0549 | 0.033526 | 0.10 |
| rs38205 | 7 | 15913588 | A | C |  | 0.019758 | 0.003094 | 1.72E-10 |  | -0.00203 | 0.007687 | 0.79 |
| rs10242866 | 7 | 17920613 | T | C |  | 0.020592 | 0.003016 | 8.60E-12 |  | -0.00439 | 0.007491 | 0.56 |
| rs4722551^a^ | 7 | 25991826 | C | T |  | -0.03202 | 0.004045 | 2.46E-15 |  | 0.004775 | 0.010152 | 0.64 |
| rs878521 | 7 | 44255643 | A | G |  | 0.021812 | 0.003408 | 1.56E-10 |  | 0.002404 | 0.008574 | 0.78 |
| rs71551223 | 7 | 71256489 | C | A |  | -0.04947 | 0.008653 | 1.09E-08 |  | 0.013992 | 0.022397 | 0.53 |
| rs147767686 | 7 | 72389537 | T | C |  | -0.07546 | 0.009374 | 8.33E-16 |  | -0.02362 | 0.025841 | 0.36 |
| rs13234131 | 7 | 73025975 | G | A |  | -0.12028 | 0.004409 | 1.31E-163 |  | 0.018365 | 0.011093 | 0.10 |
| rs28494095 | 7 | 73097720 | C | G |  | 0.035503 | 0.005698 | 4.65E-10 |  | -0.01202 | 0.014358 | 0.40 |
| rs34534990^a^ | 7 | 150283773 | C | T |  | 0.021232 | 0.00351 | 1.45E-09 |  | 0.002407 | 0.008612 | 0.78 |
| rs2980755 | 8 | 8363683 | G | A |  | -0.02591 | 0.003011 | 7.80E-18 |  | 0.015077 | 0.008026 | 0.06 |
| rs7012814 | 8 | 9173358 | A | G |  | -0.03042 | 0.002994 | 3.06E-24 |  | 0.005763 | 0.007753 | 0.46 |
| rs7833387 | 8 | 10009533 | A | G |  | -0.02247 | 0.002984 | 5.16E-14 |  | 0.014072 | 0.00781 | 0.07 |
| rs7821812 | 8 | 10644101 | C | G |  | 0.037317 | 0.003645 | 1.38E-24 |  | -0.00486 | 0.009076 | 0.59 |
| rs35246381 | 8 | 18272535 | C | T |  | 0.024461 | 0.003553 | 5.80E-12 |  | -0.00064 | 0.008742 | 0.94 |
| rs59347135 | 8 | 19750044 | G | C |  | 0.087183 | 0.007447 | 1.20E-31 |  | -0.00309 | 0.017754 | 0.86 |
| rs75551077^a^ | 8 | 19840408 | C | G |  | -0.21466 | 0.004977 | 1.00E-200 |  | -0.02494 | 0.012318 | 0.04 |
| rs143298923 | 8 | 19919713 | A | T |  | -0.09849 | 0.014249 | 4.78E-12 |  | 0.008057 | 0.033986 | 0.81 |
| rs117174179 | 8 | 19941078 | T | G |  | 0.158444 | 0.010782 | 7.35E-49 |  | 0.040503 | 0.028302 | 0.15 |
| rs146449080 | 8 | 19969742 | G | A |  | 0.084269 | 0.012095 | 3.24E-12 |  | 0.008214 | 0.02855 | 0.77 |
| rs28446899 | 8 | 72396213 | T | C |  | 0.036597 | 0.005575 | 5.23E-11 |  | 0.016316 | 0.014522 | 0.26 |
| rs2142331^a^ | 8 | 116636719 | C | T |  | 0.019258 | 0.003024 | 1.91E-10 |  | 0.004961 | 0.007632 | 0.52 |
| rs11997051 | 8 | 126648243 | C | T |  | 0.022344 | 0.003588 | 4.74E-10 |  | 0.013515 | 0.009125 | 0.14 |
| rs1567353 | 9 | 1033773 | G | C |  | 0.019114 | 0.00323 | 3.28E-09 |  | -0.00497 | 0.008067 | 0.54 |
| rs296884 | 9 | 86582923 | T | G |  | -0.02104 | 0.003404 | 6.46E-10 |  | -0.02246 | 0.015921 | 0.16 |
| rs62565259 | 9 | 102162570 | T | C |  | -0.02241 | 0.003968 | 1.62E-08 |  | 0.004398 | 0.010291 | 0.67 |
| rs703974 | 10 | 80948593 | G | A |  | -0.0175 | 0.002995 | 5.11E-09 |  | -0.01171 | 0.007491 | 0.12 |
| rs2803619 | 10 | 113934384 | G | C |  | -0.02798 | 0.003308 | 2.72E-17 |  | -0.00708 | 0.008128 | 0.38 |
| rs12786130 | 11 | 27505954 | C | T |  | 0.020903 | 0.003507 | 2.53E-09 |  | -0.00446 | 0.008739 | 0.61 |
| rs4603265 | 11 | 46708196 | T | C |  | -0.03498 | 0.004291 | 3.59E-16 |  | -0.01801 | 0.010936 | 0.10 |
| rs10838681 | 11 | 47275064 | A | G |  | -0.02816 | 0.003327 | 2.60E-17 |  | -0.01151 | 0.008409 | 0.17 |
| rs3018561^a^ | 11 | 62392229 | C | A |  | 0.016698 | 0.003023 | 3.32E-08 |  | 0.005464 | 0.007537 | 0.47 |
| rs678614 | 11 | 64799894 | A | C |  | 0.020619 | 0.0033 | 4.15E-10 |  | 0.012111 | 0.008149 | 0.14 |
| rs10750766 | 11 | 65473798 | C | A |  | -0.02177 | 0.003265 | 2.58E-11 |  | -0.00046 | 0.008166 | 0.95 |
| rs10793130 | 11 | 75459869 | A | G |  | 0.03575 | 0.005257 | 1.05E-11 |  | -0.00319 | 0.024029 | 0.89 |
| rs117059907 | 11 | 79591327 | T | C |  | 0.053911 | 0.009611 | 2.03E-08 |  | 0.027482 | 0.026108 | 0.29 |
| rs117794084 | 11 | 116433496 | T | G |  | 0.11578 | 0.011624 | 2.30E-23 |  | -0.03286 | 0.031941 | 0.30 |
| rs964184 | 11 | 116648917 | G | C |  | 0.204539 | 0.004308 | 1.00E-200 |  | 0.001871 | 0.010616 | 0.86 |
| rs12721043 | 11 | 116692293 | A | C |  | -0.2101 | 0.01456 | 3.55E-47 |  | -0.04263 | 0.043472 | 0.33 |
| rs76604009 | 11 | 117085261 | T | C |  | -0.0614 | 0.007994 | 1.58E-14 |  | 0.02363 | 0.019054 | 0.21 |
| rs116987336 | 11 | 117175658 | A | G |  | 0.149301 | 0.009164 | 1.22E-59 |  | -0.00417 | 0.025299 | 0.87 |
| rs117619191 | 11 | 117248283 | C | T |  | 0.056634 | 0.010211 | 2.92E-08 |  | 0.008727 | 0.030171 | 0.77 |
| rs17120765 | 11 | 117316546 | A | G |  | 0.028178 | 0.005048 | 2.38E-08 |  | -0.00892 | 0.012505 | 0.48 |
| rs11045172 | 12 | 20470221 | C | A |  | -0.03263 | 0.003733 | 2.33E-18 |  | -0.00305 | 0.009757 | 0.75 |
| rs11045247 | 12 | 20592624 | A | G |  | 0.031517 | 0.005653 | 2.48E-08 |  | 0.023489 | 0.014322 | 0.10 |
| rs10444533^a^ | 12 | 107229705 | T | C |  | 0.020539 | 0.00349 | 3.98E-09 |  | 0.007435 | 0.008856 | 0.40 |
| rs11065363 | 12 | 121388498 | T | C |  | 0.025823 | 0.004067 | 2.17E-10 |  | -0.00365 | 0.01024 | 0.72 |
| rs11058058 | 12 | 122635983 | A | G |  | 0.027152 | 0.003963 | 7.31E-12 |  | 0.008692 | 0.009975 | 0.38 |
| rs580063 | 12 | 123206340 | C | T |  | -0.03014 | 0.003622 | 8.84E-17 |  | -0.01005 | 0.009085 | 0.27 |
| rs7133378 | 12 | 124409502 | A | G |  | -0.03671 | 0.003175 | 6.68E-31 |  | -0.00662 | 0.007806 | 0.40 |
| rs838886 | 12 | 125264287 | T | G |  | -0.01879 | 0.003315 | 1.44E-08 |  | 0.003282 | 0.008092 | 0.69 |
| rs7140110 | 13 | 114544024 | C | T |  | 0.020288 | 0.003237 | 3.66E-10 |  | 0.00883 | 0.008432 | 0.29 |
| rs12897637 | 14 | 64239351 | C | T |  | 0.022448 | 0.004058 | 3.17E-08 |  | -0.00785 | 0.01091 | 0.47 |
| rs221902^a^ | 14 | 71601079 | G | T |  | -0.01824 | 0.00311 | 4.51E-09 |  | 0.009791 | 0.008414 | 0.24 |
| rs61704439 | 15 | 40755812 | CAA | C |  | -0.02966 | 0.005337 | 2.76E-08 |  | -0.00883 | 0.025132 | 0.73 |
| rs7167078 | 15 | 41972392 | G | C |  | -0.02314 | 0.003191 | 4.17E-13 |  | -0.002 | 0.007894 | 0.80 |
| rs146131796 | 15 | 42450180 | T | A |  | 0.067476 | 0.011149 | 1.43E-09 |  | 0.006921 | 0.027053 | 0.80 |
| rs149492745 | 15 | 43099550 | T | C |  | 0.110417 | 0.0111 | 2.62E-23 |  | 0.030951 | 0.026269 | 0.24 |
| rs150844304 | 15 | 43726625 | C | A |  | 0.133271 | 0.00936 | 5.57E-46 |  | 0.014234 | 0.021963 | 0.52 |
| rs144022883 | 15 | 44363739 | A | G |  | 0.118926 | 0.009479 | 4.32E-36 |  | 0.028174 | 0.022392 | 0.21 |
| rs4273010 | 15 | 44947434 | C | T |  | 0.103112 | 0.00968 | 1.73E-26 |  | 0.026782 | 0.024584 | 0.28 |
| rs4776793 | 15 | 66872114 | T | C |  | 0.019216 | 0.003086 | 4.77E-10 |  | 0.011318 | 0.007684 | 0.14 |
| rs11075253 | 16 | 15148646 | A | C |  | -0.02742 | 0.003238 | 2.53E-17 |  | 0.013547 | 0.008037 | 0.09 |
| rs36229491 | 16 | 56994244 | TA | T |  | -0.07463 | 0.003162 | 5.85E-123 |  | 0.006248 | 0.014709 | 0.67 |
| rs2408345 | 16 | 62807401 | A | G |  | -0.017 | 0.002962 | 9.57E-09 |  | 0.008174 | 0.007335 | 0.27 |
| rs862320 | 16 | 69651866 | T | C |  | -0.01881 | 0.003007 | 4.00E-10 |  | -0.01109 | 0.007439 | 0.14 |
| rs12921278^a^ | 16 | 72211938 | C | T |  | 0.027229 | 0.004809 | 1.49E-08 |  | 0.005374 | 0.012146 | 0.66 |
| rs2925979 | 16 | 81534790 | T | C |  | 0.037956 | 0.003219 | 4.44E-32 |  | -0.00322 | 0.008037 | 0.69 |
| rs11658311 | 17 | 17470526 | T | C |  | 0.04048 | 0.006219 | 7.56E-11 |  | -0.01792 | 0.015408 | 0.24 |
| rs151103662^a^ | 17 | 41144158 | A | G |  | 0.071005 | 0.012168 | 5.37E-09 |  | 0.023563 | 0.032909 | 0.47 |
| rs72836561 | 17 | 41926126 | T | C |  | 0.177156 | 0.008494 | 1.64E-96 |  | 0.030179 | 0.022585 | 0.18 |
| rs78027755 | 17 | 41956178 | A | G |  | 0.050769 | 0.008372 | 1.33E-09 |  | -0.02575 | 0.021661 | 0.23 |
| rs8064331 | 17 | 42858950 | T | C |  | -0.01894 | 0.00338 | 2.12E-08 |  | -0.00359 | 0.008445 | 0.67 |
| rs7211380 | 17 | 64206768 | G | A |  | -0.03687 | 0.005909 | 4.37E-10 |  | -0.02868 | 0.014318 | 0.05 |
| rs149394327 | 17 | 64228995 | C | G |  | -0.07225 | 0.008708 | 1.07E-16 |  | -0.03391 | 0.022419 | 0.13 |
| rs62084237 | 17 | 65854807 | A | G |  | 0.024954 | 0.003953 | 2.74E-10 |  | 0.015091 | 0.018145 | 0.41 |
| rs12601079 | 17 | 76400329 | G | A |  | 0.027568 | 0.003061 | 2.13E-19 |  | 0.010153 | 0.014259 | 0.48 |
| rs8083730 | 18 | 293519 | A | G |  | -0.02972 | 0.005338 | 2.57E-08 |  | 0.006433 | 0.013275 | 0.63 |
| rs77960347 | 18 | 47109955 | G | A |  | -0.07844 | 0.012713 | 6.83E-10 |  | -0.00051 | 0.032831 | 0.99 |
| rs6507937 | 18 | 47164926 | T | C |  | 0.023511 | 0.003877 | 1.33E-09 |  | 0.004188 | 0.009797 | 0.67 |
| rs7238484 | 18 | 57735552 | T | G |  | 0.021985 | 0.003334 | 4.31E-11 |  | 0.015359 | 0.008295 | 0.06 |
| rs35514860^a^ | 19 | 4966189 | C | G |  | -0.01716 | 0.003109 | 3.41E-08 |  | 0.008319 | 0.007722 | 0.28 |
| rs66921136 | 19 | 7197880 | T | C |  | 0.022597 | 0.003277 | 5.40E-12 |  | 0.008006 | 0.015453 | 0.60 |
| rs4804833 | 19 | 7970635 | A | G |  | 0.019309 | 0.003026 | 1.76E-10 |  | 0.003058 | 0.007519 | 0.68 |
| rs116483979 | 19 | 8456738 | T | C |  | -0.05881 | 0.008689 | 1.30E-11 |  | 0.007467 | 0.019858 | 0.71 |
| rs58542926 | 19 | 19379549 | T | C |  | -0.0714 | 0.005625 | 6.55E-37 |  | 0.000712 | 0.014288 | 0.96 |
| rs12973104^a^ | 19 | 56095799 | T | C |  | 0.026414 | 0.004615 | 1.05E-08 |  | -4.29E-05 | 0.011555 | 1.00 |
| rs6073958 | 20 | 44551855 | C | T |  | 0.065303 | 0.003719 | 5.63E-69 |  | -0.0003 | 0.009138 | 0.97 |
| rs6124885 | 20 | 45534053 | G | A |  | 0.038809 | 0.006502 | 2.39E-09 |  | 0.017247 | 0.016117 | 0.28 |
| rs66806308 | 20 | 46360465 | T | C |  | -0.02077 | 0.003697 | 1.93E-08 |  | -0.00379 | 0.009463 | 0.69 |
| rs8121509 | 20 | 62712053 | C | T |  | -0.01874 | 0.002964 | 2.59E-10 |  | 0.001404 | 0.007533 | 0.85 |
| rs763121 | 22 | 38879940 | G | A |  | 0.018402 | 0.003108 | 3.21E-09 |  | -0.01481 | 0.007776 | 0.06 |

Abbreviations: Chr., chromosome; SE, standard error.

^a^ Proxy variant.

**Supplementary Table S22**. Summary of 75 genetic variants used to construct the instrumental variables for linoleic acid to total fatty acids percentage in the two-sample Mendelian randomization analyses

| **Variant** | **Chr.** | **Position (GRCh37)** | **Effect allele** | **Other allele** |  | **Linoleic acid to total fatty acids percentage**  **(current study)** | | |  | **Colorectal cancer**  **(Fernandez-Rozadilla, 2023)** | | |
| --- | --- | --- | --- | --- | --- | --- | --- | --- | --- | --- | --- | --- |
|  |  |  |  |  |  | **β** | **SE** | ***P*-value** |  | **β** | **SE** | ***P*-value** |
| rs2843128 | 1 | 2315680 | A | G |  | -0.02398 | 0.003044 | 3.40E-15 |  | 0.000387 | 0.007474 | 0.96 |
| rs11249249 | 1 | 25758134 | A | G |  | -0.01827 | 0.003093 | 3.45E-09 |  | -0.00234 | 0.007412 | 0.75 |
| rs3768321 | 1 | 40035928 | T | G |  | -0.0251 | 0.003835 | 5.94E-11 |  | 0.000614 | 0.009266 | 0.95 |
| rs11591147 | 1 | 55505647 | T | G |  | -0.08183 | 0.01173 | 3.04E-12 |  | 0.03165 | 0.029231 | 0.28 |
| rs472495 | 1 | 55521313 | G | T |  | -0.01828 | 0.003193 | 1.03E-08 |  | -0.00506 | 0.007704 | 0.51 |
| rs59770119 | 1 | 219664627 | G | A |  | 0.017137 | 0.003104 | 3.39E-08 |  | 0.000313 | 0.007462 | 0.97 |
| rs6714780 | 2 | 27189063 | A | G |  | -0.05842 | 0.007135 | 2.66E-16 |  | 0.00755 | 0.018216 | 0.68 |
| rs74873433 | 2 | 28277414 | C | T |  | -0.04525 | 0.006407 | 1.64E-12 |  | -0.01899 | 0.015378 | 0.22 |
| rs4614977 | 2 | 44087024 | G | C |  | -0.0359 | 0.006411 | 2.15E-08 |  | -0.01178 | 0.015229 | 0.44 |
| rs359254 | 2 | 60482427 | A | G |  | 0.018254 | 0.003176 | 9.06E-09 |  | -0.0037 | 0.007594 | 0.63 |
| rs10195252 | 2 | 165513091 | C | T |  | 0.026739 | 0.003103 | 6.90E-18 |  | -0.0018 | 0.00745 | 0.81 |
| rs2972137 | 2 | 227106361 | G | A |  | 0.027941 | 0.003187 | 1.82E-18 |  | -0.0171 | 0.007662 | 0.03 |
| rs4678428 | 3 | 135988412 | G | A |  | 0.027554 | 0.003534 | 6.38E-15 |  | -0.00348 | 0.008531 | 0.68 |
| rs6439657 | 3 | 136494639 | T | C |  | 0.020773 | 0.003496 | 2.83E-09 |  | -0.00045 | 0.008398 | 0.96 |
| rs13131350^a^ | 4 | 17877487 | G | A |  | -0.02524 | 0.004429 | 1.21E-08 |  | 0.004422 | 0.010749 | 0.68 |
| rs11735092 | 4 | 88226231 | C | T |  | -0.01885 | 0.003084 | 9.84E-10 |  | 0.00585 | 0.007422 | 0.43 |
| rs809955 | 4 | 140874760 | A | G |  | 0.01975 | 0.003171 | 4.72E-10 |  | -0.00519 | 0.007665 | 0.50 |
| rs10056543 | 5 | 75143624 | C | A |  | 0.01892 | 0.003289 | 8.77E-09 |  | 0.001764 | 0.007928 | 0.82 |
| rs1717565 | 5 | 78440369 | C | T |  | 0.018139 | 0.003181 | 1.19E-08 |  | 0.006488 | 0.007763 | 0.40 |
| rs111676697 | 6 | 11089529 | T | G |  | -0.02061 | 0.003659 | 1.76E-08 |  | 0.007367 | 0.009132 | 0.42 |
| rs1264701^a^ | 6 | 30066358 | T | G |  | 0.022066 | 0.003844 | 9.44E-09 |  | 0.002962 | 0.009505 | 0.76 |
| rs9266834 | 6 | 31383130 | A | T |  | -0.03355 | 0.005026 | 2.46E-11 |  | 0.0111 | 0.02262 | 0.62 |
| rs998584 | 6 | 43757896 | A | C |  | -0.02122 | 0.003063 | 4.30E-12 |  | 0.005007 | 0.007495 | 0.50 |
| rs9370162 | 6 | 52728779 | G | A |  | -0.02378 | 0.003092 | 1.47E-14 |  | 0.00904 | 0.014068 | 0.52 |
| rs71562509^a^ | 6 | 139835423 | G | T |  | -0.02536 | 0.003109 | 3.46E-16 |  | -0.01279 | 0.014079 | 0.36 |
| rs17138358 | 7 | 17920253 | C | G |  | -0.02146 | 0.003114 | 5.51E-12 |  | -0.00519 | 0.007493 | 0.49 |
| rs4719841 | 7 | 25997536 | G | A |  | -0.02567 | 0.003097 | 1.14E-16 |  | 0.012858 | 0.007547 | 0.09 |
| rs10232076 | 7 | 98948904 | T | C |  | 0.031767 | 0.005582 | 1.27E-08 |  | -0.00454 | 0.013198 | 0.73 |
| rs200015011 | 7 | 130426554 | CTCG | C |  | 0.018445 | 0.003115 | 3.21E-09 |  | -0.0057 | 0.013842 | 0.68 |
| rs17410962 | 8 | 19848080 | A | G |  | 0.069362 | 0.004727 | 1.02E-48 |  | -0.01588 | 0.011191 | 0.16 |
| rs117174179 | 8 | 19941078 | T | G |  | -0.06528 | 0.011132 | 4.52E-09 |  | 0.040503 | 0.028302 | 0.15 |
| rs28601761 | 8 | 126500031 | G | C |  | 0.06862 | 0.00316 | 1.96E-104 |  | -0.01795 | 0.007527 | 0.02 |
| rs16931853 | 9 | 14495337 | A | G |  | -0.02757 | 0.004658 | 3.25E-09 |  | -0.00131 | 0.010927 | 0.90 |
| rs4149307 | 9 | 107589744 | T | C |  | 0.023953 | 0.004219 | 1.37E-08 |  | 0.014581 | 0.010087 | 0.15 |
| rs13284054 | 9 | 107669073 | C | T |  | -0.03301 | 0.0048 | 6.16E-12 |  | 0.017518 | 0.011588 | 0.13 |
| rs12257549 | 10 | 46029631 | T | C |  | 0.019847 | 0.003607 | 3.76E-08 |  | 0.024791 | 0.01625 | 0.13 |
| rs12771773^a^ | 10 | 93685434 | G | A |  | 0.018518 | 0.003172 | 5.27E-09 |  | -0.01352 | 0.007638 | 0.08 |
| rs2146017 | 10 | 96677971 | G | T |  | 0.019199 | 0.003081 | 4.62E-10 |  | -0.00181 | 0.007467 | 0.81 |
| rs17309874 | 11 | 27667236 | A | G |  | -0.0225 | 0.003488 | 1.13E-10 |  | -0.01232 | 0.00864 | 0.15 |
| rs3136449 | 11 | 46744470 | A | G |  | 0.035293 | 0.004424 | 1.50E-15 |  | -0.01707 | 0.010927 | 0.12 |
| rs78290566 | 11 | 47249919 | CG | C |  | 0.029781 | 0.004215 | 1.61E-12 |  | -0.01179 | 0.01913 | 0.54 |
| rs115713382 | 11 | 49462519 | G | A |  | -0.02303 | 0.003974 | 6.77E-09 |  | 0.004148 | 0.01785 | 0.82 |
| rs61742921 | 11 | 61025737 | T | C |  | -0.04999 | 0.008844 | 1.59E-08 |  | -0.02909 | 0.024333 | 0.23 |
| rs35169799 | 11 | 64031241 | T | C |  | -0.03786 | 0.006241 | 1.31E-09 |  | -0.0136 | 0.015007 | 0.36 |
| rs10750766 | 11 | 65473798 | C | A |  | 0.025265 | 0.00337 | 6.51E-14 |  | -0.00046 | 0.008166 | 0.95 |
| rs10160784 | 11 | 75456055 | C | T |  | 0.035102 | 0.004202 | 6.64E-17 |  | 0.006168 | 0.009756 | 0.53 |
| rs964184 | 11 | 116648917 | G | C |  | -0.05403 | 0.00447 | 1.25E-33 |  | 0.001871 | 0.010616 | 0.86 |
| rs12721043 | 11 | 116692293 | A | C |  | 0.091634 | 0.015035 | 1.10E-09 |  | -0.04263 | 0.043472 | 0.33 |
| rs34680764 | 12 | 124513777 | G | T |  | 0.025496 | 0.004677 | 4.99E-08 |  | -0.0074 | 0.0113 | 0.51 |
| rs12590259 | 14 | 58741125 | C | A |  | -0.01757 | 0.003108 | 1.58E-08 |  | -0.01457 | 0.014086 | 0.30 |
| rs17108967 | 14 | 71542168 | C | T |  | 0.017676 | 0.003229 | 4.40E-08 |  | 0.00928 | 0.008471 | 0.27 |
| rs7156419 | 14 | 102189963 | A | G |  | 0.038355 | 0.006803 | 1.72E-08 |  | -0.00106 | 0.030845 | 0.97 |
| rs2412710 | 15 | 42683787 | A | G |  | -0.06543 | 0.011451 | 1.11E-08 |  | 0.015125 | 0.026736 | 0.57 |
| rs143875230 | 15 | 43278726 | A | G |  | -0.08445 | 0.010035 | 3.93E-17 |  | 0.015011 | 0.023842 | 0.53 |
| rs139974673 | 15 | 44027885 | C | T |  | -0.1074 | 0.009666 | 1.13E-28 |  | 0.023861 | 0.022002 | 0.28 |
| rs144972973 | 15 | 44564692 | G | A |  | -0.08774 | 0.009894 | 7.47E-19 |  | 0.031724 | 0.023755 | 0.18 |
| rs261342 | 15 | 58731153 | G | C |  | -0.02061 | 0.003726 | 3.17E-08 |  | -0.0021 | 0.008924 | 0.81 |
| rs12103006 | 16 | 24726237 | A | G |  | 0.0181 | 0.003084 | 4.40E-09 |  | -0.01026 | 0.007361 | 0.16 |
| rs11508026 | 16 | 56999328 | T | C |  | 0.030702 | 0.003091 | 3.07E-23 |  | 0.010289 | 0.007438 | 0.17 |
| rs9906673 | 17 | 17746197 | C | T |  | -0.02451 | 0.0032 | 1.88E-14 |  | 0.009257 | 0.007635 | 0.23 |
| rs72836561 | 17 | 41926126 | T | C |  | -0.05381 | 0.008775 | 8.67E-10 |  | 0.030179 | 0.022585 | 0.18 |
| rs60856912 | 17 | 65892343 | T | G |  | -0.02339 | 0.004161 | 1.89E-08 |  | 0.00944 | 0.010047 | 0.35 |
| rs77542162 | 17 | 67081278 | G | A |  | 0.058516 | 0.010243 | 1.11E-08 |  | 0.021318 | 0.030656 | 0.49 |
| rs4789182 | 17 | 73374945 | G | A |  | 0.021002 | 0.003435 | 9.71E-10 |  | -0.00498 | 0.008114 | 0.54 |
| rs4969187 | 17 | 76405837 | T | C |  | -0.01903 | 0.003206 | 2.93E-09 |  | 0.007809 | 0.007755 | 0.31 |
| rs58324296 | 19 | 4969199 | C | A |  | 0.019863 | 0.003228 | 7.63E-10 |  | 0.015764 | 0.014792 | 0.29 |
| rs12610987 | 19 | 7219697 | T | C |  | 0.022033 | 0.003145 | 2.45E-12 |  | -0.00351 | 0.007498 | 0.64 |
| rs58542926 | 19 | 19379549 | T | C |  | 0.047714 | 0.005807 | 2.10E-16 |  | 0.000712 | 0.014288 | 0.96 |
| rs62116778 | 19 | 44894070 | T | G |  | -0.04376 | 0.007876 | 2.76E-08 |  | -0.00305 | 0.021593 | 0.89 |
| rs2238691 | 19 | 46179043 | A | G |  | -0.0236 | 0.003854 | 9.13E-10 |  | -8.74E-05 | 0.00911 | 0.99 |
| rs419772 | 19 | 54794205 | C | T |  | 0.020782 | 0.003731 | 2.56E-08 |  | 0.005756 | 0.009255 | 0.53 |
| rs6073972 | 20 | 44590298 | G | C |  | -0.04923 | 0.003933 | 6.06E-36 |  | 0.007083 | 0.009468 | 0.45 |
| rs6063046 | 20 | 45596378 | G | A |  | 0.020961 | 0.003386 | 6.04E-10 |  | -0.0095 | 0.008313 | 0.25 |
| rs9637192 | 21 | 46637413 | T | C |  | 0.017029 | 0.003107 | 4.22E-08 |  | 0.002196 | 0.007402 | 0.77 |
| rs3747207 | 22 | 44324855 | A | G |  | 0.036338 | 0.003706 | 1.09E-22 |  | 0.006577 | 0.008933 | 0.46 |

Abbreviations: Chr., chromosome; SE, standard error.

^a^ Proxy variant.

**Supplementary Table S23**. Summary of 32 genetic variants used to construct the instrumental variables for saturated fatty acids to total fatty acids percentage in the two-sample Mendelian randomization analyses

| **Variant** | **Chr.** | **Position (GRCh37)** | **Effect allele** | **Other allele** |  | **Saturated fatty acids to total fatty acids percentage**  **(current study)** | | |  | **Colorectal cancer**  **(Fernandez-Rozadilla, 2023)** | | |
| --- | --- | --- | --- | --- | --- | --- | --- | --- | --- | --- | --- | --- |
|  |  |  |  |  |  | **β** | **SE** | ***P*-value** |  | **β** | **SE** | ***P*-value** |
| rs12045101 | 1 | 110267651 | T | C |  | -0.02274 | 0.003653 | 4.79E-10 |  | 0.004067 | 0.008683 | 0.64 |
| rs10798620^a^ | 1 | 178538193 | A | G |  | 0.018529 | 0.003117 | 2.79E-09 |  | -0.00728 | 0.007322 | 0.32 |
| rs13389219 | 2 | 165528876 | T | C |  | -0.02564 | 0.003178 | 7.13E-16 |  | -0.00296 | 0.007489 | 0.69 |
| rs1100396 | 3 | 132021906 | C | T |  | 0.019263 | 0.003478 | 3.04E-08 |  | 0.009204 | 0.008267 | 0.27 |
| rs4918 | 3 | 186338382 | G | C |  | 0.022192 | 0.003249 | 8.47E-12 |  | -0.00257 | 0.007739 | 0.74 |
| rs13150834 | 4 | 88222673 | G | T |  | 0.017586 | 0.003139 | 2.11E-08 |  | 0.005303 | 0.007418 | 0.47 |
| rs13102909 | 4 | 111110678 | A | G |  | -0.02121 | 0.003199 | 3.39E-11 |  | -0.00651 | 0.007581 | 0.39 |
| rs1077394 | 6 | 31610384 | C | T |  | -0.02375 | 0.003314 | 7.67E-13 |  | -0.00768 | 0.007893 | 0.33 |
| rs2269423 | 6 | 32145707 | A | C |  | -0.03429 | 0.003212 | 1.38E-26 |  | -0.01745 | 0.007704 | 0.02 |
| rs34531599 | 6 | 32663978 | T | C |  | -0.03094 | 0.00487 | 2.10E-10 |  | 0.004971 | 0.022976 | 0.83 |
| rs2908522^a^ | 6 | 139835399 | C | G |  | 0.017419 | 0.003173 | 4.02E-08 |  | -0.01392 | 0.014133 | 0.32 |
| rs9295128 | 6 | 160751531 | T | G |  | -0.10075 | 0.012773 | 3.10E-15 |  | 0.013833 | 0.031504 | 0.66 |
| rs55730499 | 6 | 161005610 | T | C |  | -0.07397 | 0.00577 | 1.32E-37 |  | -0.01769 | 0.014596 | 0.23 |
| rs143843429 | 6 | 161383079 | G | A |  | -0.0834 | 0.013662 | 1.03E-09 |  | -0.01603 | 0.031047 | 0.61 |
| rs200015011 | 7 | 130426554 | CTCG | C |  | -0.02508 | 0.003176 | 2.90E-15 |  | -0.0057 | 0.013842 | 0.68 |
| rs7018333 | 8 | 59402570 | A | G |  | 0.022126 | 0.003328 | 2.95E-11 |  | -0.00281 | 0.007819 | 0.72 |
| rs28601761 | 8 | 126500031 | G | C |  | -0.05204 | 0.003223 | 1.33E-58 |  | -0.01795 | 0.007527 | 0.02 |
| rs603424 | 10 | 102075479 | A | G |  | 0.063743 | 0.004106 | 2.51E-54 |  | 0.003798 | 0.00955 | 0.69 |
| rs4917902 | 10 | 102274998 | C | G |  | -0.03985 | 0.003745 | 1.97E-26 |  | -0.01861 | 0.008877 | 0.04 |
| rs10787429 | 10 | 113949664 | T | C |  | 0.038166 | 0.00349 | 8.04E-28 |  | -0.0059 | 0.008261 | 0.47 |
| rs3135506 | 11 | 116662407 | C | G |  | -0.03828 | 0.006372 | 1.89E-09 |  | -0.01864 | 0.015113 | 0.22 |
| rs229798 | 14 | 83757483 | G | A |  | -0.01945 | 0.003454 | 1.78E-08 |  | 0.002376 | 0.008837 | 0.79 |
| rs10468017 | 15 | 58678512 | T | C |  | 0.04999 | 0.003405 | 8.86E-49 |  | 0.00388 | 0.008194 | 0.64 |
| rs1800588 | 15 | 58723675 | T | C |  | 0.045596 | 0.003781 | 1.76E-33 |  | -0.00376 | 0.008917 | 0.67 |
| rs77381129 | 17 | 41596947 | A | G |  | 0.04206 | 0.006803 | 6.30E-10 |  | 0.019108 | 0.017431 | 0.27 |
| rs62089321 | 17 | 73272551 | C | T |  | -0.02135 | 0.003501 | 1.07E-09 |  | -0.00043 | 0.008194 | 0.96 |
| rs10659627 | 19 | 7193978 | G | GCTT |  | 0.01913 | 0.00345 | 2.94E-08 |  | 0.010407 | 0.015457 | 0.50 |
| rs8105174 | 19 | 10347032 | T | C |  | -0.02305 | 0.003982 | 7.16E-09 |  | -0.01243 | 0.009712 | 0.20 |
| rs6073958 | 20 | 44551855 | C | T |  | 0.033837 | 0.003914 | 5.45E-18 |  | -0.0003 | 0.009138 | 0.97 |
| rs5746641 | 22 | 18911733 | C | T |  | -0.06093 | 0.007717 | 2.90E-15 |  | 0.005788 | 0.019604 | 0.77 |
| rs2540656^a^ | 22 | 18974973 | T | C |  | -0.03063 | 0.004927 | 5.10E-10 |  | -0.01308 | 0.011655 | 0.26 |
| rs738409 | 22 | 44324727 | G | C |  | -0.0439 | 0.003761 | 1.79E-31 |  | 0.007853 | 0.008906 | 0.38 |

Abbreviations: Chr., chromosome; SE, standard error.

^a^ Proxy variant.

**Supplementary Table S24**. Summary of 110 genetic variants used to construct the instrumental variables for omega-6 fatty acids to total fatty acids percentage in the two-sample Mendelian randomization analyses

| **Variant** | **Chr.** | **Position (GRCh37)** | **Effect allele** | **Other allele** |  | **Omega-6 fatty acids to total fatty acid percentage**  **(current study)** | | |  | **Colorectal cancer**  **(Fernandez-Rozadilla, 2023)** | | |
| --- | --- | --- | --- | --- | --- | --- | --- | --- | --- | --- | --- | --- |
|  |  |  |  |  |  | **β** | **SE** | ***P*-value** |  | **β** | **SE** | ***P*-value** |
| rs2843128 | 1 | 2315680 | A | G |  | -0.01791 | 0.003063 | 5.00E-09 |  | 0.000386744 | 0.007474 | 0.96 |
| rs193084249 | 1 | 26987646 | G | A |  | -0.06337 | 0.010413 | 1.16E-09 |  | -0.0280069 | 0.025412 | 0.27 |
| rs10889334 | 1 | 62957199 | G | C |  | 0.045496 | 0.003218 | 2.28E-45 |  | -0.011008 | 0.007851 | 0.16 |
| rs2660302 | 1 | 98520219 | T | A |  | -0.02243 | 0.003971 | 1.63E-08 |  | -0.0139693 | 0.009427 | 0.14 |
| rs16844101 | 1 | 199011539 | T | G |  | 0.022965 | 0.004101 | 2.14E-08 |  | -0.00142256 | 0.009716 | 0.88 |
| rs2820426 | 1 | 219660535 | A | G |  | 0.021572 | 0.003159 | 8.61E-12 |  | -0.000775632 | 0.007528 | 0.92 |
| rs59484402 | 1 | 230302838 | C | CT |  | -0.02188 | 0.003216 | 1.02E-11 |  | 0.0281601 | 0.014463 | 0.05 |
| rs4564803 | 2 | 21205502 | T | G |  | 0.043794 | 0.003667 | 7.33E-33 |  | -0.00158181 | 0.008587 | 0.85 |
| rs10193900 | 2 | 25919967 | A | G |  | -0.02205 | 0.003306 | 2.55E-11 |  | 0.00422593 | 0.007863 | 0.59 |
| rs62129551 | 2 | 26940912 | T | C |  | -0.05326 | 0.006574 | 5.48E-16 |  | -0.0287288 | 0.016168 | 0.08 |
| rs114856131 | 2 | 27156721 | T | C |  | -0.07622 | 0.013677 | 2.51E-08 |  | -0.036314 | 0.034354 | 0.29 |
| rs143915183 | 2 | 27174079 | A | G |  | -0.05661 | 0.007659 | 1.46E-13 |  | -0.026752 | 0.018979 | 0.16 |
| rs6714780 | 2 | 27189063 | A | G |  | -0.07278 | 0.007176 | 3.65E-24 |  | 0.00754973 | 0.018216 | 0.68 |
| rs111750756 | 2 | 28476360 | T | C |  | -0.07042 | 0.01252 | 1.87E-08 |  | -0.0299651 | 0.030658 | 0.33 |
| rs359252 | 2 | 60481644 | T | C |  | 0.017412 | 0.003157 | 3.47E-08 |  | -0.0021047 | 0.007504 | 0.78 |
| rs13389219 | 2 | 165528876 | T | C |  | 0.034746 | 0.003136 | 1.63E-28 |  | -0.00295837 | 0.007489 | 0.69 |
| rs2564921 | 3 | 53125585 | T | C |  | 0.018524 | 0.003087 | 1.97E-09 |  | 0.00443268 | 0.007384 | 0.55 |
| rs684773 | 3 | 135956305 | A | C |  | 0.032366 | 0.003624 | 4.22E-19 |  | -0.00206046 | 0.008704 | 0.81 |
| rs7427111 | 3 | 136495041 | C | T |  | 0.025743 | 0.003516 | 2.46E-13 |  | -0.00102833 | 0.008402 | 0.90 |
| rs9844972 | 3 | 150097635 | C | G |  | -0.03491 | 0.006171 | 1.55E-08 |  | -0.00419291 | 0.014857 | 0.78 |
| rs9817452 | 3 | 156795414 | T | G |  | 0.017879 | 0.003171 | 1.72E-08 |  | 0.00625181 | 0.007576 | 0.41 |
| rs11924648^a^ | 3 | 170717996 | G | A |  | -0.02541 | 0.00463 | 4.09E-08 |  | -0.0131363 | 0.010869 | 0.23 |
| rs7700107 | 4 | 17880416 | C | A |  | -0.0274 | 0.004446 | 7.15E-10 |  | 0.00351712 | 0.010735 | 0.74 |
| rs7661844 | 4 | 87468625 | C | T |  | -0.0214 | 0.003068 | 3.04E-12 |  | 0.011329 | 0.007331 | 0.12 |
| rs1471251 | 4 | 87976359 | T | A |  | -0.02871 | 0.003145 | 7.10E-20 |  | 0.0149742 | 0.007515 | 0.05 |
| rs6825776 | 4 | 89710789 | T | A |  | -0.01807 | 0.003181 | 1.35E-08 |  | -0.000412868 | 0.007589 | 0.96 |
| rs57800857 | 4 | 140863365 | C | A |  | 0.017783 | 0.003218 | 3.29E-08 |  | -0.00279568 | 0.007732 | 0.72 |
| rs111366116 | 5 | 53295546 | T | C |  | -0.02913 | 0.004872 | 2.25E-09 |  | 0.0172852 | 0.011671 | 0.14 |
| rs72754154 | 5 | 55812380 | A | G |  | 0.04084 | 0.007066 | 7.47E-09 |  | 0.00776485 | 0.016433 | 0.64 |
| rs3936511 | 5 | 55860781 | G | A |  | -0.03914 | 0.003895 | 9.44E-24 |  | 0.00811353 | 0.009428 | 0.39 |
| rs1316776 | 5 | 78430607 | A | C |  | 0.02095 | 0.003206 | 6.42E-11 |  | 0.00373706 | 0.00783 | 0.63 |
| rs6882345 | 5 | 156397673 | G | A |  | 0.021194 | 0.003183 | 2.75E-11 |  | -0.00260676 | 0.007616 | 0.73 |
| rs2914231 | 5 | 158011435 | C | G |  | -0.02052 | 0.003664 | 2.16E-08 |  | -0.00808732 | 0.008655 | 0.35 |
| rs9266834 | 6 | 31383130 | A | T |  | -0.04143 | 0.005056 | 2.55E-16 |  | 0.0110997 | 0.02262 | 0.62 |
| rs437179 | 6 | 31929014 | A | C |  | 0.020967 | 0.003289 | 1.83E-10 |  | -0.00787637 | 0.008223 | 0.34 |
| rs28752509^a^ | 6 | 32579623 | T | C |  | -0.02482 | 0.003085 | 8.50E-16 |  | 0.0247432 | 0.014207 | 0.08 |
| rs998584 | 6 | 43757896 | A | C |  | -0.02903 | 0.003082 | 4.62E-21 |  | 0.00500661 | 0.007495 | 0.50 |
| rs9370162 | 6 | 52728779 | G | A |  | -0.02812 | 0.00311 | 1.56E-19 |  | 0.00904014 | 0.014068 | 0.52 |
| rs72959041^a^ | 6 | 127454893 | A | G |  | -0.04418 | 0.007259 | 1.16E-09 |  | 0.01005 | 0.018052 | 0.58 |
| rs12190724^a^ | 6 | 130395960 | G | C |  | 0.02024 | 0.003491 | 6.75E-09 |  | -0.0111856 | 0.008445 | 0.19 |
| rs71562509^a^ | 6 | 139835423 | G | T |  | -0.02965 | 0.003128 | 2.56E-21 |  | -0.0127885 | 0.014079 | 0.36 |
| rs9986540 | 6 | 160242487 | T | G |  | 0.107143 | 0.01276 | 4.63E-17 |  | 0.0166116 | 0.03176 | 0.60 |
| rs9295128 | 6 | 160751531 | T | G |  | 0.184802 | 0.012602 | 1.16E-48 |  | 0.0138328 | 0.031504 | 0.66 |
| rs55730499 | 6 | 161005610 | T | C |  | 0.115295 | 0.005692 | 4.03E-91 |  | -0.0176872 | 0.014596 | 0.23 |
| rs143843429 | 6 | 161383079 | G | A |  | 0.138501 | 0.013484 | 9.63E-25 |  | -0.0160319 | 0.031047 | 0.61 |
| rs61735260 | 6 | 161560461 | A | G |  | 0.085178 | 0.01386 | 7.99E-10 |  | -0.0548958 | 0.033526 | 0.10 |
| rs17138358 | 7 | 17920253 | C | G |  | -0.0195 | 0.003134 | 4.90E-10 |  | -0.00518906 | 0.007493 | 0.49 |
| rs4719841 | 7 | 25997536 | G | A |  | -0.02628 | 0.003116 | 3.36E-17 |  | 0.0128576 | 0.007547 | 0.09 |
| rs2699809 | 7 | 26402747 | T | C |  | -0.01726 | 0.003065 | 1.77E-08 |  | 0.000391186 | 0.007331 | 0.96 |
| rs147767686 | 7 | 72389537 | T | C |  | 0.070968 | 0.009736 | 3.12E-13 |  | -0.0236187 | 0.025841 | 0.36 |
| rs13234378 | 7 | 73026151 | T | A |  | 0.107436 | 0.00458 | 1.64E-121 |  | 0.018515 | 0.011096 | 0.10 |
| rs28494095 | 7 | 73097720 | C | G |  | -0.03703 | 0.005918 | 3.91E-10 |  | -0.0120157 | 0.014358 | 0.40 |
| rs11762784 | 7 | 130424646 | A | G |  | 0.026607 | 0.003132 | 1.99E-17 |  | -0.0111543 | 0.007422 | 0.13 |
| rs2980755 | 8 | 8363683 | G | A |  | 0.017562 | 0.003127 | 1.96E-08 |  | 0.0150774 | 0.008026 | 0.06 |
| rs28588745 | 8 | 10647044 | T | A |  | -0.02814 | 0.003807 | 1.45E-13 |  | -0.00317533 | 0.009121 | 0.73 |
| rs55865839 | 8 | 11625372 | C | A |  | -0.02373 | 0.003715 | 1.69E-10 |  | -0.0118759 | 0.009118 | 0.19 |
| rs4921913 | 8 | 18272377 | C | T |  | -0.0292 | 0.003692 | 2.63E-15 |  | -0.00108541 | 0.008746 | 0.90 |
| rs59347135 | 8 | 19750044 | G | C |  | -0.05636 | 0.007737 | 3.23E-13 |  | -0.00308572 | 0.017754 | 0.86 |
| rs117174179 | 8 | 19941078 | T | G |  | -0.10621 | 0.011201 | 2.51E-21 |  | 0.0405028 | 0.028302 | 0.15 |
| rs11781439 | 8 | 20573754 | A | C |  | -0.02203 | 0.003919 | 1.90E-08 |  | 0.00851794 | 0.009392 | 0.36 |
| rs1128435 | 8 | 27301725 | G | C |  | -0.0285 | 0.005203 | 4.30E-08 |  | 0.00572404 | 0.011955 | 0.63 |
| rs897161 | 8 | 126606217 | A | C |  | 0.01764 | 0.003081 | 1.03E-08 |  | -0.00950653 | 0.007381 | 0.20 |
| rs16931853 | 9 | 14495337 | A | G |  | -0.02625 | 0.004688 | 2.16E-08 |  | -0.00130837 | 0.010927 | 0.90 |
| rs10761785 | 10 | 65318766 | G | T |  | -0.02216 | 0.00308 | 6.33E-13 |  | 0.000667954 | 0.007347 | 0.93 |
| rs1892501 | 10 | 81097266 | G | A |  | -0.02155 | 0.003881 | 2.81E-08 |  | -0.0134887 | 0.009132 | 0.14 |
| rs2792022 | 10 | 93740429 | C | T |  | 0.021028 | 0.003457 | 1.18E-09 |  | -0.0162006 | 0.0083 | 0.05 |
| rs6486122^a^ | 11 | 13361524 | C | T |  | 0.018717 | 0.003312 | 1.60E-08 |  | 0.00474111 | 0.007827 | 0.54 |
| rs11030088 | 11 | 27646247 | A | G |  | -0.02225 | 0.003553 | 3.79E-10 |  | -0.012452 | 0.008739 | 0.15 |
| rs3136449 | 11 | 46744470 | A | G |  | 0.035097 | 0.004451 | 3.16E-15 |  | -0.0170658 | 0.010927 | 0.12 |
| rs10838681 | 11 | 47275064 | A | G |  | 0.024473 | 0.003455 | 1.42E-12 |  | -0.0115069 | 0.008409 | 0.17 |
| rs115713382 | 11 | 49462519 | G | A |  | -0.02195 | 0.003998 | 4.03E-08 |  | 0.00414822 | 0.01785 | 0.82 |
| rs10750766 | 11 | 65473798 | C | A |  | 0.023982 | 0.003391 | 1.53E-12 |  | -0.000461598 | 0.008166 | 0.95 |
| rs117794084 | 11 | 116433496 | T | G |  | -0.083 | 0.012074 | 6.25E-12 |  | -0.0328602 | 0.031941 | 0.30 |
| rs964184 | 11 | 116648917 | G | C |  | -0.15219 | 0.004486 | 1.00E-200 |  | 0.00187148 | 0.010616 | 0.86 |
| rs12721043 | 11 | 116692293 | A | C |  | 0.135208 | 0.015126 | 3.97E-19 |  | -0.0426276 | 0.043472 | 0.33 |
| rs116987336 | 11 | 117175658 | A | G |  | -0.11388 | 0.00952 | 5.71E-33 |  | -0.00416659 | 0.025299 | 0.87 |
| rs11045172 | 12 | 20470221 | C | A |  | 0.021257 | 0.003879 | 4.24E-08 |  | -0.00304919 | 0.009757 | 0.75 |
| rs55695203 | 12 | 21357753 | C | G |  | -0.02918 | 0.00429 | 1.05E-11 |  | -0.0131561 | 0.009903 | 0.18 |
| rs7133378 | 12 | 124409502 | A | G |  | 0.022773 | 0.003298 | 5.05E-12 |  | -0.00661537 | 0.007806 | 0.40 |
| rs1951203 | 14 | 58699353 | T | C |  | 0.018706 | 0.003104 | 1.67E-09 |  | 0.0194495 | 0.013944 | 0.16 |
| rs67981189 | 14 | 71472226 | G | A |  | 0.021183 | 0.003268 | 9.07E-11 |  | 0.0113737 | 0.008511 | 0.18 |
| rs7170463 | 15 | 41888918 | G | A |  | 0.019456 | 0.003327 | 4.98E-09 |  | -0.00119977 | 0.007995 | 0.88 |
| rs184334219 | 15 | 42721845 | A | G |  | -0.08838 | 0.011374 | 7.86E-15 |  | 0.00661616 | 0.026372 | 0.80 |
| rs143875230 | 15 | 43278726 | A | G |  | -0.11855 | 0.010093 | 7.62E-32 |  | 0.0150105 | 0.023842 | 0.53 |
| rs139974673 | 15 | 44027885 | C | T |  | -0.14483 | 0.009723 | 3.74E-50 |  | 0.0238608 | 0.022002 | 0.28 |
| rs148489550 | 15 | 44581461 | A | G |  | -0.11303 | 0.009749 | 4.53E-31 |  | 0.0252598 | 0.023234 | 0.28 |
| rs10468017 | 15 | 58678512 | T | C |  | -0.05874 | 0.00336 | 2.16E-68 |  | 0.00387984 | 0.008194 | 0.64 |
| rs1800588 | 15 | 58723675 | T | C |  | -0.07127 | 0.00373 | 2.63E-81 |  | -0.00375608 | 0.008917 | 0.67 |
| rs2871866 | 15 | 99221888 | C | T |  | 0.020505 | 0.003673 | 2.36E-08 |  | 0.00222549 | 0.008796 | 0.80 |
| rs12103006 | 16 | 24726237 | A | G |  | 0.017746 | 0.003103 | 1.08E-08 |  | -0.0102596 | 0.007361 | 0.16 |
| rs1532625 | 16 | 57005301 | T | C |  | 0.02973 | 0.003093 | 7.17E-22 |  | 0.00937266 | 0.007418 | 0.21 |
| rs34682685 | 16 | 72096227 | A | G |  | -0.02823 | 0.005057 | 2.37E-08 |  | 0.0104737 | 0.012332 | 0.40 |
| rs2925979 | 16 | 81534790 | T | C |  | -0.02194 | 0.003344 | 5.31E-11 |  | -0.00321786 | 0.008037 | 0.69 |
| rs113186018 | 17 | 41422469 | A | G |  | -0.03897 | 0.00679 | 9.56E-09 |  | 0.0252655 | 0.018839 | 0.18 |
| rs72836561 | 17 | 41926126 | T | C |  | -0.08538 | 0.008828 | 4.04E-22 |  | 0.030179 | 0.022585 | 0.18 |
| rs116878033 | 17 | 42031770 | T | C |  | -0.04529 | 0.008296 | 4.79E-08 |  | -0.0178855 | 0.020189 | 0.38 |
| rs4793155 | 17 | 42888381 | T | C |  | -0.02538 | 0.004536 | 2.22E-08 |  | -0.00544371 | 0.0111 | 0.62 |
| rs149394327 | 17 | 64228995 | C | G |  | 0.050234 | 0.009044 | 2.79E-08 |  | -0.0339109 | 0.022419 | 0.13 |
| rs60856912 | 17 | 65892343 | T | G |  | -0.02706 | 0.004187 | 1.03E-10 |  | 0.00944011 | 0.010047 | 0.35 |
| rs12601079 | 17 | 76400329 | G | A |  | -0.0201 | 0.003179 | 2.60E-10 |  | 0.0101527 | 0.014259 | 0.48 |
| rs12979771 | 19 | 4963443 | T | C |  | 0.020879 | 0.003344 | 4.26E-10 |  | 0.0246506 | 0.015019 | 0.10 |
| rs10405423 | 19 | 7211311 | C | A |  | 0.022613 | 0.003303 | 7.65E-12 |  | -0.0089742 | 0.007844 | 0.25 |
| rs58542926 | 19 | 19379549 | T | C |  | 0.080541 | 0.005841 | 3.12E-43 |  | 0.000711974 | 0.014288 | 0.96 |
| rs2238691 | 19 | 46179043 | A | G |  | -0.02207 | 0.003878 | 1.27E-08 |  | -0.000087383 | 0.00911 | 0.99 |
| rs633236 | 19 | 49274975 | T | C |  | 0.020569 | 0.003236 | 2.06E-10 |  | -0.000502739 | 0.008732 | 0.95 |
| rs6073958 | 20 | 44551855 | C | T |  | -0.06058 | 0.003862 | 2.06E-55 |  | -0.000297408 | 0.009138 | 0.97 |
| rs6063046 | 20 | 45596378 | G | A |  | 0.019072 | 0.003407 | 2.18E-08 |  | -0.00950241 | 0.008313 | 0.25 |
| rs8115058 | 20 | 62432709 | G | A |  | 0.018047 | 0.003175 | 1.32E-08 |  | 0.0144519 | 0.007676 | 0.06 |
| rs62223042^a^ | 21 | 40683740 | G | A |  | -0.01738 | 0.003177 | 4.47E-08 |  | 0.000264 | 0.007609 | 0.97 |
| rs4818766 | 21 | 46635351 | A | G |  | 0.018773 | 0.003117 | 1.71E-09 |  | 0.00177157 | 0.007391 | 0.81 |

Abbreviations: Chr., chromosome; SE, standard error.

^a^ Proxy variant.

**Supplementary Table S25**. Summary of 97 genetic variants used to construct the instrumental variables for glycoprotein acetyls (mmol/L) in the two-sample Mendelian randomization analyses

| **Variant** | **Chr.** | **Position (GRCh37)** | **Effect allele** | **Other allele** |  | **Glycoprotein acetyls (mmol/L)**  **(current study)** | | |  | **Colorectal cancer**  **(Fernandez-Rozadilla, 2023)** | | |
| --- | --- | --- | --- | --- | --- | --- | --- | --- | --- | --- | --- | --- |
|  |  |  |  |  |  | **β** | **SE** | ***P*-value** |  | **β** | **SE** | ***P*-value** |
| rs909537 | 1 | 23810063 | C | G |  | -0.03159 | 0.005206 | 1.29E-09 |  | 0.00701 | 0.012408 | 0.57 |
| rs188468174 | 1 | 25291697 | T | C |  | -0.1168 | 0.01346 | 4.09E-18 |  | -0.0703 | 0.041087 | 0.09 |
| rs114165349 | 1 | 27021913 | C | G |  | 0.093366 | 0.010505 | 6.26E-19 |  | -0.02559 | 0.025315 | 0.31 |
| rs1768808 | 1 | 46503217 | C | T |  | -0.02253 | 0.003311 | 1.02E-11 |  | 0.003337 | 0.007716 | 0.67 |
| rs2131925 | 1 | 63025942 | G | T |  | -0.03281 | 0.003284 | 1.69E-23 |  | -0.01039 | 0.007815 | 0.18 |
| rs4459081^a^ | 1 | 66164327 | G | C |  | -0.02611 | 0.003296 | 2.35E-15 |  | 0.008475 | 0.007663 | 0.27 |
| rs907662 | 1 | 117848822 | A | G |  | -0.02125 | 0.003706 | 9.88E-09 |  | 0.000133 | 0.008609 | 0.99 |
| rs9427403 | 1 | 161494322 | C | A |  | 0.024568 | 0.004105 | 2.18E-09 |  | 0.011395 | 0.009655 | 0.24 |
| rs12033493^a^ | 1 | 205062433 | T | C |  | 0.02565 | 0.003994 | 1.35E-10 |  | 0.014292 | 0.009461 | 0.13 |
| rs17008806 | 1 | 220976257 | C | A |  | -0.02318 | 0.003505 | 3.74E-11 |  | 0.009813 | 0.00819 | 0.23 |
| rs56188865 | 1 | 247606276 | C | T |  | -0.02478 | 0.003258 | 2.79E-14 |  | 0.00657 | 0.007591 | 0.39 |
| rs10184054 | 2 | 21203877 | G | C |  | -0.02389 | 0.003776 | 2.50E-10 |  | -0.00124 | 0.008648 | 0.89 |
| rs72804880 | 2 | 27173314 | A | G |  | 0.070385 | 0.007607 | 2.21E-20 |  | -0.03262 | 0.018684 | 0.08 |
| rs59537151 | 2 | 48293911 | CG | C |  | 0.019426 | 0.003462 | 2.01E-08 |  | -0.0046 | 0.015276 | 0.76 |
| rs1971819^a^ | 2 | 203705787 | G | C |  | -0.02547 | 0.004026 | 2.52E-10 |  | -0.01589 | 0.009586 | 0.10 |
| rs62260788 | 3 | 48068610 | T | C |  | 0.032278 | 0.005377 | 1.94E-09 |  | -0.0156 | 0.013643 | 0.25 |
| rs4563439 | 3 | 49383779 | T | C |  | 0.019981 | 0.00317 | 2.90E-10 |  | -0.009 | 0.007453 | 0.23 |
| rs13316065 | 3 | 49884913 | T | C |  | 0.028874 | 0.003366 | 9.66E-18 |  | -0.01074 | 0.008032 | 0.18 |
| rs79287178 | 3 | 172294500 | A | G |  | 0.082219 | 0.010055 | 2.92E-16 |  | -0.00569 | 0.022441 | 0.80 |
| rs2070632 | 3 | 186334004 | A | C |  | -0.04141 | 0.003539 | 1.28E-31 |  | -0.0061 | 0.008414 | 0.47 |
| rs13108218 | 4 | 3443931 | A | G |  | 0.038764 | 0.003275 | 2.62E-32 |  | -0.01061 | 0.007716 | 0.17 |
| rs71603401 | 4 | 18034463 | G | A |  | 0.027182 | 0.004676 | 6.12E-09 |  | 0.010807 | 0.010839 | 0.32 |
| rs7697204 | 4 | 148980174 | C | T |  | -0.02327 | 0.003586 | 8.61E-11 |  | 0.007529 | 0.008384 | 0.37 |
| rs2546191^a^ | 5 | 95232541 | A | G |  | 0.019718 | 0.003508 | 1.90E-08 |  | -0.01331 | 0.008235 | 0.11 |
| rs72801474 | 5 | 132444128 | A | G |  | -0.0388 | 0.005414 | 7.69E-13 |  | -0.00074 | 0.013686 | 0.96 |
| rs2731673 | 5 | 176839898 | T | C |  | -0.02357 | 0.003601 | 5.92E-11 |  | 0.01761 | 0.00864 | 0.04 |
| rs1800562 | 6 | 26093141 | A | G |  | -0.05155 | 0.00587 | 1.61E-18 |  | 0.013733 | 0.015058 | 0.36 |
| rs13214703 | 6 | 27941387 | C | T |  | -0.03173 | 0.00545 | 5.81E-09 |  | 0.016752 | 0.014321 | 0.24 |
| rs35901019 | 6 | 28494225 | A | G |  | -0.03132 | 0.005476 | 1.08E-08 |  | 0.003032 | 0.014183 | 0.83 |
| rs2523583 | 6 | 31327895 | G | A |  | -0.05183 | 0.003459 | 9.67E-51 |  | 0.010688 | 0.008606 | 0.21 |
| rs2763981 | 6 | 31840021 | T | A |  | -0.04807 | 0.003333 | 3.95E-47 |  | 0.011202 | 0.008201 | 0.17 |
| rs7746553 | 6 | 31895973 | G | C |  | 0.024344 | 0.00436 | 2.37E-08 |  | 0.007355 | 0.010234 | 0.47 |
| rs998584 | 6 | 43757896 | A | C |  | 0.018279 | 0.003151 | 6.63E-09 |  | 0.005007 | 0.007495 | 0.50 |
| rs12190724 | 6 | 130395960 | G | C |  | -0.0205 | 0.003568 | 9.20E-09 |  | -0.01119 | 0.008445 | 0.19 |
| rs12208357 | 6 | 160543148 | T | C |  | 0.037857 | 0.006174 | 8.72E-10 |  | -0.00591 | 0.014415 | 0.68 |
| rs9295128 | 6 | 160751531 | T | G |  | -0.10296 | 0.012885 | 1.35E-15 |  | 0.013833 | 0.031504 | 0.66 |
| rs10455872 | 6 | 161010118 | G | A |  | -0.06274 | 0.005837 | 6.12E-27 |  | -0.02379 | 0.015166 | 0.12 |
| rs4719841 | 7 | 25997536 | G | A |  | 0.020087 | 0.003185 | 2.84E-10 |  | 0.012858 | 0.007547 | 0.09 |
| rs138222746 | 7 | 36258218 | A | G |  | -0.0819 | 0.014558 | 1.85E-08 |  | -0.06596 | 0.03713 | 0.08 |
| rs139392920 | 7 | 72246350 | C | G |  | -0.08174 | 0.010493 | 6.72E-15 |  | -0.02312 | 0.027305 | 0.40 |
| rs3812316 | 7 | 73020337 | G | C |  | -0.10646 | 0.004683 | 3.01E-114 |  | 0.018773 | 0.011097 | 0.09 |
| rs10254101 | 7 | 151415536 | T | C |  | 0.019571 | 0.003463 | 1.60E-08 |  | -0.00112 | 0.00831 | 0.89 |
| rs670044 | 8 | 9892529 | A | C |  | -0.02027 | 0.00334 | 1.28E-09 |  | 0.006167 | 0.008128 | 0.45 |
| rs11784312 | 8 | 10872767 | A | G |  | 0.022489 | 0.003328 | 1.40E-11 |  | -0.00755 | 0.007927 | 0.34 |
| rs2721961 | 8 | 116657911 | G | T |  | -0.02838 | 0.003507 | 5.86E-16 |  | -0.00994 | 0.008295 | 0.23 |
| rs150611042 | 9 | 117083803 | A | C |  | 0.11953 | 0.006439 | 7.45E-77 |  | -0.03345 | 0.015605 | 0.03 |
| rs529565 | 9 | 136149500 | C | T |  | 0.027832 | 0.00337 | 1.49E-16 |  | 0.005016 | 0.007715 | 0.52 |
| rs7924036 | 10 | 65191645 | G | T |  | 0.024918 | 0.003135 | 1.89E-15 |  | 0.002866 | 0.00734 | 0.70 |
| rs17476364 | 10 | 71094504 | C | T |  | -0.02755 | 0.005047 | 4.83E-08 |  | -0.01855 | 0.012632 | 0.14 |
| rs1892501 | 10 | 81097266 | G | A |  | 0.028383 | 0.003966 | 8.29E-13 |  | -0.01349 | 0.009132 | 0.14 |
| rs1412445 | 10 | 91002804 | T | C |  | 0.021235 | 0.003318 | 1.55E-10 |  | -0.0018 | 0.007808 | 0.82 |
| rs6486122^a^ | 11 | 13361524 | C | T |  | -0.0228 | 0.003385 | 1.64E-11 |  | 0.004741 | 0.007827 | 0.54 |
| rs1519125 | 11 | 16244588 | C | G |  | 0.021778 | 0.003215 | 1.25E-11 |  | 0.002257 | 0.007512 | 0.76 |
| rs11231721 | 11 | 63957835 | A | G |  | 0.036469 | 0.006677 | 4.71E-08 |  | -0.029 | 0.015615 | 0.06 |
| rs10750766 | 11 | 65473798 | C | A |  | -0.01977 | 0.003465 | 1.16E-08 |  | -0.00046 | 0.008166 | 0.95 |
| rs964184 | 11 | 116648917 | G | C |  | 0.112675 | 0.004591 | 8.17E-133 |  | 0.001871 | 0.010616 | 0.86 |
| rs116987336 | 11 | 117175658 | A | G |  | 0.072139 | 0.009731 | 1.24E-13 |  | -0.00417 | 0.025299 | 0.87 |
| rs12575474 | 11 | 122521021 | C | T |  | 0.024633 | 0.003225 | 2.22E-14 |  | -0.00376 | 0.007604 | 0.62 |
| rs59379014 | 11 | 126228000 | T | C |  | 0.047607 | 0.006033 | 3.01E-15 |  | -0.01689 | 0.014122 | 0.23 |
| rs12300845 | 12 | 24195798 | T | G |  | 0.048481 | 0.008748 | 3.00E-08 |  | 0.017586 | 0.019481 | 0.37 |
| rs2583949 | 12 | 66194243 | T | C |  | 0.02952 | 0.005172 | 1.15E-08 |  | -0.01126 | 0.011729 | 0.34 |
| rs1890942 | 14 | 69765644 | A | G |  | -0.02058 | 0.003264 | 2.90E-10 |  | 0.003678 | 0.008334 | 0.66 |
| rs2332536 | 14 | 71634952 | G | A |  | -0.01969 | 0.003286 | 2.06E-09 |  | 0.010414 | 0.008388 | 0.21 |
| rs17580 | 14 | 94847262 | A | T |  | -0.06001 | 0.007382 | 4.34E-16 |  | -0.0204 | 0.021576 | 0.34 |
| rs149492745 | 15 | 43099550 | T | C |  | 0.075504 | 0.011785 | 1.49E-10 |  | 0.030951 | 0.026269 | 0.24 |
| rs150844304 | 15 | 43726625 | C | A |  | 0.078374 | 0.009938 | 3.13E-15 |  | 0.014234 | 0.021963 | 0.52 |
| rs138893177 | 15 | 44297617 | T | C |  | 0.0696 | 0.010083 | 5.12E-12 |  | 0.026318 | 0.022362 | 0.24 |
| rs151291132 | 15 | 44842210 | G | A |  | 0.061164 | 0.010294 | 2.82E-09 |  | 0.025695 | 0.024068 | 0.29 |
| rs17241731 | 16 | 69893821 | T | C |  | -0.06479 | 0.008893 | 3.22E-13 |  | 0.014206 | 0.02344 | 0.54 |
| rs80229424 | 16 | 70384434 | T | C |  | 0.051977 | 0.008755 | 2.91E-09 |  | 0.019532 | 0.029699 | 0.51 |
| rs17881236 | 16 | 70514382 | A | G |  | 0.05734 | 0.009295 | 6.89E-10 |  | 0.024271 | 0.024624 | 0.32 |
| rs193281563 | 16 | 70646632 | T | C |  | -0.07404 | 0.010118 | 2.53E-13 |  | -0.00159 | 0.053411 | 0.98 |
| rs113754130 | 16 | 70816361 | C | G |  | 0.085924 | 0.013944 | 7.20E-10 |  | -0.02191 | 0.041389 | 0.60 |
| rs11646091 | 16 | 70825649 | C | T |  | 0.091918 | 0.007839 | 9.66E-32 |  | -0.00423 | 0.020978 | 0.84 |
| rs139739055 | 16 | 71237119 | A | G |  | -0.1137 | 0.015673 | 4.05E-13 |  | 0.045501 | 0.040983 | 0.27 |
| rs9935618 | 16 | 71414059 | T | G |  | 0.053291 | 0.003256 | 3.64E-60 |  | 0.006852 | 0.014315 | 0.63 |
| rs1549292 | 16 | 71983664 | G | T |  | -0.04272 | 0.004902 | 2.93E-18 |  | -0.00032 | 0.010754 | 0.98 |
| rs77303550 | 16 | 72079657 | T | C |  | 0.160903 | 0.003967 | 1.00E-200 |  | 0.004135 | 0.009452 | 0.66 |
| rs7190009 | 16 | 72996813 | C | T |  | 0.049857 | 0.008525 | 4.97E-09 |  | -0.03406 | 0.019278 | 0.08 |
| rs2925979 | 16 | 81534790 | T | C |  | 0.020517 | 0.003418 | 1.93E-09 |  | -0.00322 | 0.008037 | 0.69 |
| rs35207980 | 16 | 85271686 | A | G |  | -0.02236 | 0.003891 | 9.09E-09 |  | -0.00275 | 0.009024 | 0.76 |
| rs11078597 | 17 | 1618363 | C | T |  | 0.024046 | 0.004022 | 2.26E-09 |  | -0.00906 | 0.009677 | 0.35 |
| rs2974998^a^ | 17 | 17995619 | G | A |  | 0.019014 | 0.003428 | 2.91E-08 |  | 0.004702 | 0.007889 | 0.55 |
| rs145947882 | 17 | 41809207 | C | A |  | 0.068045 | 0.010158 | 2.10E-11 |  | 0.022352 | 0.025414 | 0.38 |
| rs9303533 | 17 | 45386540 | G | A |  | 0.0205 | 0.003145 | 7.10E-11 |  | 0.002367 | 0.007328 | 0.75 |
| rs149394327 | 17 | 64228995 | C | G |  | -0.05876 | 0.009243 | 2.06E-10 |  | -0.03391 | 0.022419 | 0.13 |
| rs62086903 | 17 | 66016006 | C | T |  | 0.021884 | 0.003771 | 6.53E-09 |  | 0.003802 | 0.009271 | 0.68 |
| rs58542926 | 19 | 19379549 | T | C |  | -0.05819 | 0.005971 | 1.96E-22 |  | 0.000712 | 0.014288 | 0.96 |
| rs12609794 | 19 | 35555585 | G | A |  | -0.0234 | 0.003605 | 8.58E-11 |  | -0.00632 | 0.00843 | 0.45 |
| rs141622900 | 19 | 45426792 | A | G |  | 0.054963 | 0.007335 | 6.76E-14 |  | -0.01274 | 0.016856 | 0.45 |
| rs59774409 | 19 | 50016748 | T | C |  | 0.068987 | 0.005707 | 1.25E-33 |  | 0.008873 | 0.013449 | 0.51 |
| rs12459419 | 19 | 51728477 | T | C |  | -0.02055 | 0.003345 | 8.03E-10 |  | 0.007604 | 0.007912 | 0.34 |
| rs10405357 | 19 | 54759666 | C | T |  | 0.022655 | 0.003185 | 1.14E-12 |  | -0.00688 | 0.00759 | 0.37 |
| rs6065904 | 20 | 44534651 | A | G |  | 0.02601 | 0.003826 | 1.06E-11 |  | 0.003353 | 0.008897 | 0.71 |
| rs62222988 | 21 | 40569508 | C | T |  | 0.018551 | 0.003251 | 1.15E-08 |  | -0.00119 | 0.007584 | 0.88 |
| rs9618180 | 22 | 18479072 | C | T |  | -0.02094 | 0.00364 | 8.76E-09 |  | -0.0136 | 0.008544 | 0.11 |
| rs738409 | 22 | 44324727 | G | C |  | -0.03242 | 0.003795 | 1.30E-17 |  | 0.007853 | 0.008906 | 0.38 |

Abbreviations: Chr., chromosome; SE, standard error.

^a^ Proxy variant.

**Supplementary Table S26**. Summary of 23 genetic variants used to construct the instrumental variables for log-transformed 3-hydroxybutyrate (mmol/L) in the two-sample Mendelian randomization analyses

| **Variant** | **Chr.** | **Position (GRCh37)** | **Effect allele** | **Other allele** |  | **Log_3-hydroxybutyrate (mmol/L)**  **(current study)** | | |  | **Colorectal cancer**  **(Fernandez-Rozadilla, 2023)** | | |
| --- | --- | --- | --- | --- | --- | --- | --- | --- | --- | --- | --- | --- |
|  |  |  |  |  |  | **β** | **SE** | ***P*-value** |  | **β** | **SE** | ***P*-value** |
| rs59484402 | 1 | 230302838 | C | CT |  | 0.028938 | 0.003291 | 1.46E-18 |  | 0.02816 | 0.014463 | 0.05 |
| rs2910628 | 5 | 41659364 | T | A |  | -0.03531 | 0.003676 | 7.75E-22 |  | -0.00708 | 0.008458 | 0.40 |
| rs905095 | 5 | 42168124 | G | A |  | -0.02055 | 0.003359 | 9.52E-10 |  | 0.017451 | 0.014797 | 0.24 |
| rs72757003 | 5 | 42742377 | T | A |  | -0.04014 | 0.006962 | 8.17E-09 |  | -0.0114 | 0.016577 | 0.49 |
| rs7005904 | 8 | 8218597 | T | C |  | 0.018743 | 0.003196 | 4.49E-09 |  | -0.00273 | 0.007722 | 0.72 |
| rs7460226 | 8 | 10197718 | G | A |  | -0.01899 | 0.003236 | 4.36E-09 |  | -0.00158 | 0.007869 | 0.84 |
| rs4841465^a^ | 8 | 10819854 | C | T |  | -0.01916 | 0.003153 | 1.22E-09 |  | 0.006144 | 0.007835 | 0.43 |
| rs1736060 | 8 | 11664738 | C | T |  | 0.022101 | 0.003187 | 4.07E-12 |  | 0.000377 | 0.007738 | 0.96 |
| rs2954021 | 8 | 126482077 | A | G |  | -0.01838 | 0.003145 | 5.14E-09 |  | 0.011202 | 0.00736 | 0.13 |
| rs72691630 | 8 | 144297787 | A | G |  | -0.02278 | 0.004053 | 1.92E-08 |  | 0.005943 | 0.009549 | 0.53 |
| rs4149310 | 9 | 107589134 | T | A |  | 0.030138 | 0.004321 | 3.07E-12 |  | 0.014908 | 0.010088 | 0.14 |
| rs2297991 | 10 | 113913222 | T | C |  | -0.03548 | 0.003499 | 3.72E-24 |  | -0.01017 | 0.008049 | 0.21 |
| rs499974 | 11 | 75455021 | A | C |  | 0.030211 | 0.00433 | 3.02E-12 |  | 0.00628 | 0.009766 | 0.52 |
| rs964184^a^ | 11 | 116648917 | G | C |  | 0.037449 | 0.004673 | 1.12E-15 |  | 0.001871 | 0.010616 | 0.86 |
| rs11058058 | 12 | 122635983 | A | G |  | -0.02379 | 0.004218 | 1.70E-08 |  | 0.008692 | 0.009975 | 0.38 |
| rs73228032 | 12 | 123322415 | A | C |  | -0.02907 | 0.004501 | 1.06E-10 |  | 0.010382 | 0.011405 | 0.36 |
| rs145730801 | 14 | 94768196 | C | T |  | -0.04936 | 0.008007 | 7.07E-10 |  | -0.01868 | 0.035282 | 0.60 |
| rs10468017 | 15 | 58678512 | T | C |  | -0.02883 | 0.003448 | 6.12E-17 |  | 0.00388 | 0.008194 | 0.64 |
| rs588136 | 15 | 58730498 | C | T |  | -0.02767 | 0.003885 | 1.06E-12 |  | -0.00125 | 0.009023 | 0.89 |
| rs9939224 | 16 | 57002732 | T | G |  | -0.02621 | 0.003865 | 1.20E-11 |  | -0.00204 | 0.00909 | 0.82 |
| rs56177707 | 16 | 72877335 | A | G |  | 0.043323 | 0.006941 | 4.35E-10 |  | -0.00967 | 0.016913 | 0.57 |
| rs429358 | 19 | 45411941 | C | T |  | 0.052987 | 0.004345 | 3.36E-34 |  | 0.014097 | 0.010638 | 0.19 |
| rs11672660 | 19 | 46180184 | T | C |  | -0.02226 | 0.003972 | 2.08E-08 |  | 0.000436 | 0.009106 | 0.96 |

Abbreviations: Chr., chromosome; SE, standard error.

^a^ Proxy variant.

**Supplementary Table S27**. Summary of 51 genetic variants used to construct the instrumental variables for cholesterol relative to total lipids in small LDL percentage in the two-sample Mendelian randomization analyses

| **Variant** | **Chr.** | **Position (GRCh37)** | **Effect allele** | **Other allele** |  | **Cholesterol to total lipids in small LDL percentage**  **(current study)** | | |  | **Colorectal cancer**  **(Fernandez-Rozadilla, 2023)** | | |
| --- | --- | --- | --- | --- | --- | --- | --- | --- | --- | --- | --- | --- |
|  |  |  |  |  |  | **β** | **SE** | ***P*-value** |  | **β** | **SE** | ***P*-value** |
| rs3768321 | 1 | 40035928 | T | G |  | -0.02764 | 0.003961 | 2.97E-12 |  | 0.000614 | 0.009266 | 0.95 |
| rs11591147 | 1 | 55505647 | T | G |  | -0.15226 | 0.012111 | 3.11E-36 |  | 0.03165 | 0.029231 | 0.28 |
| rs472495 | 1 | 55521313 | G | T |  | -0.02373 | 0.003298 | 6.17E-13 |  | -0.00506 | 0.007704 | 0.51 |
| rs321236 | 1 | 96478860 | A | C |  | 0.019166 | 0.003297 | 6.13E-09 |  | -0.00402 | 0.007578 | 0.60 |
| rs629301^a^ | 1 | 109818306 | G | T |  | -0.03478 | 0.003778 | 3.41E-20 |  | -0.00557 | 0.008852 | 0.53 |
| rs4656292 | 1 | 161194641 | A | G |  | -0.02101 | 0.00327 | 1.34E-10 |  | 0.002241 | 0.007529 | 0.77 |
| rs2642438 | 1 | 220970028 | A | G |  | -0.02244 | 0.003441 | 6.91E-11 |  | 0.013449 | 0.008037 | 0.09 |
| rs1367117 | 2 | 21263900 | A | G |  | 0.03084 | 0.003323 | 1.69E-20 |  | -0.00056 | 0.007909 | 0.94 |
| rs4299376 | 2 | 44072576 | G | T |  | 0.022666 | 0.003367 | 1.69E-11 |  | 0.005144 | 0.00792 | 0.52 |
| rs1047891 | 2 | 211540507 | A | C |  | -0.02282 | 0.003383 | 1.52E-11 |  | 0.004631 | 0.008017 | 0.56 |
| rs1154988 | 3 | 135925191 | T | A |  | 0.021089 | 0.003761 | 2.05E-08 |  | -0.00272 | 0.008787 | 0.76 |
| rs6453131^a^ | 5 | 74644706 | G | T |  | 0.02948 | 0.00325 | 1.19E-19 |  | -0.00157 | 0.007533 | 0.84 |
| rs79220007 | 6 | 26098474 | C | T |  | -0.0325 | 0.005916 | 3.95E-08 |  | 0.015504 | 0.015148 | 0.31 |
| rs1130368^a^ | 6 | 32632818 | G | T |  | 0.026771 | 0.004453 | 1.83E-09 |  | 0.011607 | 0.019474 | 0.55 |
| rs4719925 | 7 | 28188995 | G | A |  | 0.033477 | 0.005644 | 3.00E-09 |  | -0.01259 | 0.012413 | 0.31 |
| rs62466318 | 7 | 73042085 | T | C |  | 0.025285 | 0.003943 | 1.44E-10 |  | 0.009634 | 0.009283 | 0.30 |
| rs2980867 | 8 | 126487691 | T | G |  | -0.0216 | 0.00343 | 3.04E-10 |  | -0.00103 | 0.008169 | 0.90 |
| rs4149310 | 9 | 107589134 | T | A |  | 0.028139 | 0.004325 | 7.72E-11 |  | 0.014908 | 0.010088 | 0.14 |
| rs13284054 | 9 | 107669073 | C | T |  | -0.02926 | 0.004961 | 3.70E-09 |  | 0.017518 | 0.011588 | 0.13 |
| rs532436 | 9 | 136149830 | A | G |  | 0.06324 | 0.004063 | 1.37E-54 |  | 0.016638 | 0.009188 | 0.07 |
| rs34297856^a^ | 9 | 139322444 | CTA | C |  | -0.01999 | 0.003483 | 9.53E-09 |  | 0.011315 | 0.016198 | 0.48 |
| rs7896518 | 10 | 65104500 | G | A |  | 0.028063 | 0.003263 | 8.07E-18 |  | -0.00484 | 0.007488 | 0.52 |
| rs2792751 | 10 | 113940329 | T | C |  | 0.019636 | 0.003527 | 2.59E-08 |  | -0.00681 | 0.008172 | 0.40 |
| rs10750766 | 11 | 65473798 | C | A |  | 0.024089 | 0.00348 | 4.47E-12 |  | -0.00046 | 0.008166 | 0.95 |
| rs1219549 | 11 | 75454622 | C | T |  | -0.02709 | 0.00433 | 3.98E-10 |  | 0.007449 | 0.009742 | 0.44 |
| rs964184 | 11 | 116648917 | G | C |  | -0.04152 | 0.004617 | 2.41E-19 |  | 0.001871 | 0.010616 | 0.86 |
| rs76970536 | 11 | 126250680 | A | G |  | 0.03799 | 0.006127 | 5.64E-10 |  | -0.00581 | 0.013993 | 0.68 |
| rs2494747 | 14 | 105258437 | G | T |  | 0.019257 | 0.003255 | 3.30E-09 |  | -0.00305 | 0.0088 | 0.73 |
| rs143875230 | 15 | 43278726 | A | G |  | -0.06868 | 0.010361 | 3.39E-11 |  | 0.015011 | 0.023842 | 0.53 |
| rs147233090 | 15 | 44028047 | T | C |  | -0.08972 | 0.010295 | 2.92E-18 |  | 0.026732 | 0.023625 | 0.26 |
| rs144972973 | 15 | 44564692 | G | A |  | -0.0673 | 0.010218 | 4.50E-11 |  | 0.031724 | 0.023755 | 0.18 |
| rs34149555^a^ | 15 | 58550000 | A | G |  | 0.021599 | 0.003172 | 9.82E-12 |  | 0.019218 | 0.013845 | 0.17 |
| rs261290 | 15 | 58678720 | T | C |  | -0.08659 | 0.003312 | 2.30E-150 |  | 0.004702 | 0.007805 | 0.55 |
| rs2070895 | 15 | 58723939 | A | G |  | -0.10152 | 0.003815 | 8.86E-156 |  | -0.00514 | 0.008869 | 0.56 |
| rs6078 | 15 | 58833993 | A | G |  | -0.05236 | 0.009507 | 3.63E-08 |  | -0.00576 | 0.02661 | 0.83 |
| rs821840 | 16 | 56993886 | G | A |  | -0.07784 | 0.003365 | 3.19E-118 |  | 0.007435 | 0.014983 | 0.62 |
| rs1566455 | 16 | 69900164 | G | T |  | 0.017798 | 0.003188 | 2.38E-08 |  | 0.010191 | 0.014125 | 0.47 |
| rs62061425 | 17 | 7073091 | G | A |  | -0.02566 | 0.003938 | 7.29E-11 |  | 0.001932 | 0.009589 | 0.84 |
| rs77542162 | 17 | 67081278 | G | A |  | 0.061958 | 0.010578 | 4.72E-09 |  | 0.021318 | 0.030656 | 0.49 |
| rs7219625 | 17 | 76390080 | T | C |  | 0.023724 | 0.003168 | 6.99E-14 |  | -0.01176 | 0.00735 | 0.11 |
| rs74489351 | 18 | 46578242 | A | C |  | 0.13611 | 0.015473 | 1.42E-18 |  | 0.000735 | 0.041391 | 0.99 |
| rs77960347 | 18 | 47109955 | G | A |  | 0.129462 | 0.01355 | 1.25E-21 |  | -0.00051 | 0.032831 | 0.99 |
| rs4939883 | 18 | 47167214 | T | C |  | -0.0369 | 0.004101 | 2.32E-19 |  | 0.004316 | 0.009702 | 0.66 |
| rs58542926 | 19 | 19379549 | T | C |  | -0.05412 | 0.005997 | 1.82E-19 |  | 0.000712 | 0.014288 | 0.96 |
| rs188247550 | 19 | 19396616 | T | C |  | -0.08226 | 0.014985 | 4.04E-08 |  | -0.05936 | 0.04061 | 0.14 |
| rs7412^a^ | 19 | 45412079 | T | C |  | -0.16955 | 0.005765 | 1.05E-189 |  | -0.02703 | 0.013873 | 0.05 |
| rs3810291 | 19 | 47569003 | G | A |  | 0.019122 | 0.003356 | 1.21E-08 |  | 0.000878 | 0.007954 | 0.91 |
| rs367070 | 19 | 54800500 | G | A |  | 0.032951 | 0.003802 | 4.46E-18 |  | 0.0039 | 0.009138 | 0.67 |
| rs364585 | 20 | 12962718 | A | G |  | -0.02085 | 0.003228 | 1.07E-10 |  | -0.00365 | 0.00756 | 0.63 |
| rs117113213 | 20 | 39165692 | A | G |  | 0.0528 | 0.008966 | 3.89E-09 |  | -0.03078 | 0.022893 | 0.18 |
| rs111602331 | 20 | 44557474 | C | T |  | 0.081144 | 0.004058 | 7.18E-89 |  | 0.004996 | 0.009447 | 0.60 |

Abbreviations: Chr., chromosome; LDL, low-density lipoprotein; SE, standard error.

^a^ Proxy variant.

**Supplementary Table S28**. Summary of 145 genetic variants used to construct the instrumental variables for log-transformed triglycerides relative to total lipids in IDL percentage in the two-sample Mendelian randomization analyses

| **Variant** | **Chr.** | **Position (GRCh37)** | **Effect allele** | **Other allele** |  | **Log_triglycerides to total lipids in IDL percentage**  **(current study)** | | |  | **Colorectal cancer**  **(Fernandez-Rozadilla, 2023)** | | |
| --- | --- | --- | --- | --- | --- | --- | --- | --- | --- | --- | --- | --- |
|  |  |  |  |  |  | **β** | **SE** | ***P*-value** |  | **β** | **SE** | ***P*-value** |
| rs193084249 | 1 | 26987646 | G | A |  | 0.075262 | 0.010382 | 4.21E-13 |  | -0.02801 | 0.025412 | 0.27 |
| rs72663503 | 1 | 39969059 | T | C |  | 0.032417 | 0.003645 | 6.01E-19 |  | 0.000798 | 0.008706 | 0.93 |
| rs11591147 | 1 | 55505647 | T | G |  | 0.190458 | 0.011761 | 6.01E-59 |  | 0.03165 | 0.029231 | 0.28 |
| rs472495 | 1 | 55521313 | G | T |  | 0.027865 | 0.003203 | 3.34E-18 |  | -0.00506 | 0.007704 | 0.51 |
| rs1690761^a^ | 1 | 62955862 | C | T |  | -0.03608 | 0.003204 | 2.06E-29 |  | -0.00965 | 0.007839 | 0.22 |
| rs7528419 | 1 | 109817192 | G | A |  | 0.044833 | 0.003671 | 2.75E-34 |  | -0.00626 | 0.008886 | 0.48 |
| rs1127317^a^ | 1 | 154556040 | G | T |  | -0.01915 | 0.003335 | 9.35E-09 |  | 0.004103 | 0.008005 | 0.61 |
| rs2820426 | 1 | 219660535 | A | G |  | -0.01786 | 0.003149 | 1.43E-08 |  | -0.00078 | 0.007528 | 0.92 |
| rs2642438 | 1 | 220970028 | A | G |  | 0.032145 | 0.003342 | 6.67E-22 |  | 0.013449 | 0.008037 | 0.09 |
| rs2281721 | 1 | 230297136 | C | T |  | 0.040171 | 0.003138 | 1.65E-37 |  | 0.004489 | 0.007537 | 0.55 |
| rs4665710 | 2 | 21221035 | A | C |  | -0.07458 | 0.003788 | 3.41E-86 |  | -0.00251 | 0.008893 | 0.78 |
| rs11689541 | 2 | 21372547 | C | T |  | 0.039794 | 0.006379 | 4.43E-10 |  | 0.005091 | 0.030212 | 0.87 |
| rs6714780 | 2 | 27189063 | A | G |  | 0.061538 | 0.007156 | 8.05E-18 |  | 0.00755 | 0.018216 | 0.68 |
| rs4245791 | 2 | 44074431 | C | T |  | -0.02688 | 0.00326 | 1.68E-16 |  | 0.0055 | 0.007922 | 0.49 |
| rs12692735 | 2 | 165504565 | T | G |  | -0.0287 | 0.003183 | 1.98E-19 |  | 0.001803 | 0.007614 | 0.81 |
| rs117350179 | 3 | 12374332 | G | C |  | 0.053716 | 0.009655 | 2.65E-08 |  | 0.009386 | 0.021782 | 0.67 |
| rs684773 | 3 | 135956305 | A | C |  | -0.04052 | 0.003612 | 3.37E-29 |  | -0.00206 | 0.008704 | 0.81 |
| rs6439657 | 3 | 136494639 | T | C |  | -0.03361 | 0.003507 | 9.43E-22 |  | -0.00045 | 0.008398 | 0.96 |
| rs9844972 | 3 | 150097635 | C | G |  | 0.044114 | 0.006152 | 7.51E-13 |  | -0.00419 | 0.014857 | 0.78 |
| rs9817452 | 3 | 156795414 | T | G |  | -0.02001 | 0.003162 | 2.48E-10 |  | 0.006252 | 0.007576 | 0.41 |
| rs3215234 | 3 | 170724091 | GT | G |  | 0.034902 | 0.004627 | 4.61E-14 |  | 0.027694 | 0.02107 | 0.19 |
| rs13316580 | 3 | 172262053 | C | T |  | 0.024973 | 0.004557 | 4.25E-08 |  | -0.00765 | 0.010901 | 0.48 |
| rs73243877 | 4 | 26047616 | G | A |  | 0.025079 | 0.004086 | 8.37E-10 |  | 0.016323 | 0.009811 | 0.10 |
| rs7676961^a^ | 4 | 39684217 | T | C |  | 0.018794 | 0.003054 | 7.59E-10 |  | -0.01072 | 0.007324 | 0.14 |
| rs7675258 | 4 | 77413179 | G | A |  | -0.01836 | 0.003066 | 2.12E-09 |  | 0.00662 | 0.007341 | 0.37 |
| rs7661844 | 4 | 87468625 | C | T |  | 0.019959 | 0.003059 | 6.83E-11 |  | 0.011329 | 0.007331 | 0.12 |
| rs13107325 | 4 | 103188709 | T | C |  | 0.057108 | 0.005805 | 7.77E-23 |  | 0.017069 | 0.014028 | 0.22 |
| rs61335963 | 5 | 55809035 | C | G |  | -0.03875 | 0.006227 | 4.90E-10 |  | 0.008351 | 0.014681 | 0.57 |
| rs3936511^a^ | 5 | 55860781 | G | A |  | 0.039275 | 0.003883 | 4.87E-24 |  | 0.008114 | 0.009428 | 0.39 |
| rs37538 | 5 | 57610069 | G | C |  | 0.017536 | 0.003151 | 2.62E-08 |  | 0.0043 | 0.007531 | 0.57 |
| rs5744552 | 5 | 74816944 | G | C |  | -0.03969 | 0.003143 | 1.48E-36 |  | -0.00834 | 0.007531 | 0.27 |
| rs146313134 | 5 | 78433740 | A | C |  | -0.02403 | 0.003186 | 4.66E-14 |  | -0.01595 | 0.016286 | 0.33 |
| rs10044647^a^ | 5 | 122668602 | G | A |  | 0.017112 | 0.003122 | 4.25E-08 |  | 0.005667 | 0.007372 | 0.44 |
| rs272838 | 5 | 131638817 | T | C |  | 0.03047 | 0.004183 | 3.23E-13 |  | -0.01314 | 0.00975 | 0.18 |
| rs72801474^a^ | 5 | 132444128 | A | G |  | -0.0308 | 0.005282 | 5.49E-09 |  | -0.00074 | 0.013686 | 0.96 |
| rs1363232 | 5 | 156383422 | A | G |  | -0.01762 | 0.003232 | 5.00E-08 |  | -0.00462 | 0.007776 | 0.55 |
| rs2963468 | 5 | 158003020 | G | A |  | 0.020384 | 0.003657 | 2.48E-08 |  | -0.00942 | 0.008683 | 0.28 |
| rs7773004 | 6 | 26267755 | G | A |  | -0.02602 | 0.003056 | 1.70E-17 |  | -0.00118 | 0.007358 | 0.87 |
| rs35840219^a^ | 6 | 31249267 | C | G |  | 0.057171 | 0.005745 | 2.50E-23 |  | -0.01618 | 0.015087 | 0.28 |
| rs622871 | 6 | 31878495 | A | G |  | -0.02732 | 0.003335 | 2.58E-16 |  | -0.00847 | 0.008416 | 0.31 |
| rs28752510 | 6 | 32579626 | A | G |  | 0.030872 | 0.003077 | 1.09E-23 |  | 0.024753 | 0.014207 | 0.08 |
| rs998584 | 6 | 43757896 | A | C |  | 0.026973 | 0.003073 | 1.69E-18 |  | 0.005007 | 0.007495 | 0.50 |
| rs9370162 | 6 | 52728779 | G | A |  | 0.019852 | 0.003101 | 1.54E-10 |  | 0.00904 | 0.014068 | 0.52 |
| rs72959041^a^ | 6 | 127454893 | A | G |  | 0.056728 | 0.007236 | 4.52E-15 |  | 0.01005 | 0.018052 | 0.58 |
| rs2908522^a^ | 6 | 139835399 | C | G |  | 0.028575 | 0.003122 | 5.53E-20 |  | -0.01392 | 0.014133 | 0.32 |
| rs191555775^a^ | 6 | 161005389 | T | A |  | 0.056528 | 0.004971 | 5.87E-30 |  | 0.014382 | 0.023492 | 0.54 |
| rs836548^a^ | 7 | 6439448 | T | A |  | -0.01944 | 0.003439 | 1.58E-08 |  | 0.011064 | 0.008283 | 0.18 |
| rs38205 | 7 | 15913588 | A | C |  | 0.019437 | 0.003204 | 1.31E-09 |  | -0.00203 | 0.007687 | 0.79 |
| rs6461354 | 7 | 17914600 | T | C |  | 0.022952 | 0.003122 | 1.98E-13 |  | -0.00448 | 0.007489 | 0.55 |
| rs4722551 | 7 | 25991826 | C | T |  | -0.03659 | 0.004188 | 2.41E-18 |  | 0.004775 | 0.010152 | 0.64 |
| rs112577710 | 7 | 44200715 | TTAATTA | T |  | 0.025244 | 0.004267 | 3.31E-09 |  | 0.029936 | 0.019197 | 0.12 |
| rs147767686 | 7 | 72389537 | T | C |  | -0.08216 | 0.009706 | 2.59E-17 |  | -0.02362 | 0.025841 | 0.36 |
| rs13234378 | 7 | 73026151 | T | A |  | -0.12159 | 0.004564 | 4.64E-156 |  | 0.018515 | 0.011096 | 0.10 |
| rs3177697 | 7 | 100804542 | T | G |  | 0.021338 | 0.003579 | 2.50E-09 |  | -0.00669 | 0.008604 | 0.44 |
| rs34748838^a^ | 7 | 130459242 | T | C |  | -0.02535 | 0.003065 | 1.37E-16 |  | -0.01038 | 0.007418 | 0.16 |
| rs2980755 | 8 | 8363683 | G | A |  | -0.02179 | 0.003118 | 2.78E-12 |  | 0.015077 | 0.008026 | 0.06 |
| rs7012814 | 8 | 9173358 | A | G |  | -0.03732 | 0.0031 | 2.32E-33 |  | 0.005763 | 0.007753 | 0.46 |
| rs36102779 | 8 | 9749609 | G | A |  | -0.02279 | 0.003332 | 8.02E-12 |  | 0.008533 | 0.008234 | 0.30 |
| rs7821812 | 8 | 10644101 | C | G |  | 0.030939 | 0.003775 | 2.49E-16 |  | -0.00486 | 0.009076 | 0.59 |
| rs13280055 | 8 | 11522353 | A | G |  | 0.035388 | 0.004687 | 4.34E-14 |  | 0.006909 | 0.011295 | 0.54 |
| rs35246381 | 8 | 18272535 | C | T |  | 0.023899 | 0.003679 | 8.25E-11 |  | -0.00064 | 0.008742 | 0.94 |
| rs59347135 | 8 | 19750044 | G | C |  | 0.072884 | 0.007711 | 3.36E-21 |  | -0.00309 | 0.017754 | 0.86 |
| rs143298923 | 8 | 19919713 | A | T |  | -0.08125 | 0.014754 | 3.66E-08 |  | 0.008057 | 0.033986 | 0.81 |
| rs117174179 | 8 | 19941078 | T | G |  | 0.144913 | 0.011162 | 1.60E-38 |  | 0.040503 | 0.028302 | 0.15 |
| rs17092008 | 8 | 19961274 | T | C |  | 0.035732 | 0.006331 | 1.66E-08 |  | 0.016762 | 0.015912 | 0.29 |
| rs117452590 | 8 | 41688190 | A | G |  | -0.07213 | 0.012912 | 2.32E-08 |  | 0.060325 | 0.035106 | 0.09 |
| rs28446899 | 8 | 72396213 | T | C |  | 0.033399 | 0.005773 | 7.23E-09 |  | 0.016316 | 0.014522 | 0.26 |
| rs1574542 | 8 | 117122598 | A | G |  | -0.01727 | 0.003152 | 4.29E-08 |  | -5.16E-05 | 0.007553 | 0.99 |
| rs2980888 | 8 | 126507308 | T | C |  | 0.06442 | 0.003325 | 1.43E-83 |  | 0.000591 | 0.008175 | 0.94 |
| rs72724627 | 8 | 126635162 | G | A |  | 0.028122 | 0.004198 | 2.11E-11 |  | 0.013093 | 0.010379 | 0.21 |
| rs550057^a^ | 9 | 136146597 | T | C |  | -0.02617 | 0.003515 | 9.71E-14 |  | 0.014071 | 0.00827 | 0.09 |
| rs10761771^a^ | 10 | 65230164 | C | T |  | -0.04273 | 0.003062 | 3.12E-44 |  | 0.000802 | 0.00735 | 0.91 |
| rs2803619 | 10 | 113934384 | G | C |  | -0.03297 | 0.003425 | 6.23E-22 |  | -0.00708 | 0.008128 | 0.38 |
| rs7074440 | 10 | 114785424 | A | G |  | 0.020782 | 0.003337 | 4.71E-10 |  | 0.008623 | 0.008038 | 0.28 |
| rs11030107^a^ | 11 | 27694835 | G | A |  | 0.021269 | 0.00348 | 9.91E-10 |  | -0.01473 | 0.008536 | 0.08 |
| rs4603265 | 11 | 46708196 | T | C |  | -0.03781 | 0.004442 | 1.71E-17 |  | -0.01801 | 0.010936 | 0.10 |
| rs78290566 | 11 | 47249919 | CG | C |  | -0.03328 | 0.004229 | 3.56E-15 |  | -0.01179 | 0.01913 | 0.54 |
| rs113253936 | 11 | 48160609 | T | C |  | -0.02701 | 0.004888 | 3.29E-08 |  | 0.007063 | 0.01199 | 0.56 |
| rs11229003 | 11 | 57108291 | A | G |  | 0.033982 | 0.004807 | 1.55E-12 |  | -0.01058 | 0.011715 | 0.37 |
| rs11605489 | 11 | 62472357 | G | A |  | 0.018946 | 0.003244 | 5.20E-09 |  | -0.00737 | 0.007722 | 0.34 |
| rs35169799 | 11 | 64031241 | T | C |  | 0.048206 | 0.006261 | 1.37E-14 |  | -0.0136 | 0.015007 | 0.36 |
| rs10750766 | 11 | 65473798 | C | A |  | -0.03149 | 0.00338 | 1.19E-20 |  | -0.00046 | 0.008166 | 0.95 |
| rs673335 | 11 | 75450576 | C | T |  | 0.028893 | 0.004195 | 5.72E-12 |  | 0.004154 | 0.009714 | 0.67 |
| rs117794084 | 11 | 116433496 | T | G |  | 0.117088 | 0.012036 | 2.31E-22 |  | -0.03286 | 0.031941 | 0.30 |
| rs964184^a^ | 11 | 116648917 | G | C |  | 0.188735 | 0.004465 | 1.00E-200 |  | 0.001871 | 0.010616 | 0.86 |
| rs12721043 | 11 | 116692293 | A | C |  | -0.14089 | 0.01508 | 9.49E-21 |  | -0.04263 | 0.043472 | 0.33 |
| rs76604009 | 11 | 117085261 | T | C |  | -0.04707 | 0.008278 | 1.30E-08 |  | 0.02363 | 0.019054 | 0.21 |
| rs116987336 | 11 | 117175658 | A | G |  | 0.14553 | 0.009489 | 4.64E-53 |  | -0.00417 | 0.025299 | 0.87 |
| rs11045172 | 12 | 20470221 | C | A |  | -0.02444 | 0.003867 | 2.61E-10 |  | -0.00305 | 0.009757 | 0.75 |
| rs58310495 | 12 | 21357711 | T | C |  | 0.033099 | 0.004277 | 1.01E-14 |  | -0.01365 | 0.009889 | 0.17 |
| rs3922628 | 12 | 123209295 | A | T |  | -0.02478 | 0.003783 | 5.78E-11 |  | -0.01093 | 0.009087 | 0.23 |
| rs7133378 | 12 | 124409502 | A | G |  | -0.02248 | 0.003288 | 8.16E-12 |  | -0.00662 | 0.007806 | 0.40 |
| rs7160525^a^ | 14 | 64232220 | A | G |  | 0.030878 | 0.004184 | 1.59E-13 |  | -0.00791 | 0.010877 | 0.47 |
| rs61704439 | 15 | 40755812 | CAA | C |  | -0.03476 | 0.005527 | 3.20E-10 |  | -0.00883 | 0.025132 | 0.73 |
| rs7170463 | 15 | 41888918 | G | A |  | -0.02422 | 0.003317 | 2.90E-13 |  | -0.0012 | 0.007995 | 0.88 |
| rs184334219 | 15 | 42721845 | A | G |  | 0.118272 | 0.011339 | 1.82E-25 |  | 0.006616 | 0.026372 | 0.80 |
| rs143875230 | 15 | 43278726 | A | G |  | 0.148649 | 0.010061 | 2.26E-49 |  | 0.015011 | 0.023842 | 0.53 |
| rs139974673 | 15 | 44027885 | C | T |  | 0.173819 | 0.009691 | 6.96E-72 |  | 0.023861 | 0.022002 | 0.28 |
| rs148489550 | 15 | 44581461 | A | G |  | 0.135194 | 0.009718 | 5.67E-44 |  | 0.02526 | 0.023234 | 0.28 |
| rs11854318 | 15 | 58571982 | A | G |  | -0.0501 | 0.003459 | 1.66E-47 |  | -0.00725 | 0.008267 | 0.38 |
| rs4775041^a^ | 15 | 58674695 | C | G |  | 0.137479 | 0.003314 | 1.00E-200 |  | 0.003864 | 0.008079 | 0.63 |
| rs261334^a^ | 15 | 58726744 | G | C |  | 0.162572 | 0.003723 | 1.00E-200 |  | -0.00118 | 0.009025 | 0.90 |
| rs6078 | 15 | 58833993 | A | G |  | 0.076189 | 0.009233 | 1.57E-16 |  | -0.00576 | 0.02661 | 0.83 |
| rs7183404^a^ | 15 | 59157634 | T | C |  | -0.03799 | 0.006182 | 7.98E-10 |  | -0.01824 | 0.015102 | 0.23 |
| rs4776793 | 15 | 66872114 | T | C |  | 0.019307 | 0.003195 | 1.52E-09 |  | 0.011318 | 0.007684 | 0.14 |
| rs7175132 | 15 | 101891508 | G | A |  | -0.01735 | 0.003153 | 3.74E-08 |  | -0.01207 | 0.007588 | 0.11 |
| rs12918213 | 16 | 1249494 | G | A |  | 0.017334 | 0.003088 | 1.99E-08 |  | -0.0159 | 0.017008 | 0.35 |
| rs11644601 | 16 | 15172118 | C | T |  | -0.02599 | 0.003357 | 9.89E-15 |  | 0.016853 | 0.008081 | 0.04 |
| rs12720918 | 16 | 56994212 | C | T |  | 0.01968 | 0.003395 | 6.76E-09 |  | -0.00655 | 0.008239 | 0.43 |
| rs12720926 | 16 | 56998918 | G | A |  | -0.02486 | 0.003101 | 1.09E-15 |  | 0.010568 | 0.007439 | 0.16 |
| rs113563886 | 16 | 67947158 | C | T |  | -0.03006 | 0.004777 | 3.16E-10 |  | 0.000208 | 0.021425 | 0.99 |
| rs244418 | 16 | 69622762 | A | G |  | -0.02482 | 0.00311 | 1.49E-15 |  | -0.01101 | 0.00744 | 0.14 |
| rs2925979 | 16 | 81534790 | T | C |  | 0.033081 | 0.003333 | 3.30E-23 |  | -0.00322 | 0.008037 | 0.69 |
| rs34460487 | 17 | 4685228 | A | G |  | 0.018729 | 0.003231 | 6.75E-09 |  | 0.002064 | 0.008177 | 0.80 |
| rs72836561 | 17 | 41926126 | T | C |  | 0.121194 | 0.0088 | 3.90E-43 |  | 0.030179 | 0.022585 | 0.18 |
| rs75134409 | 17 | 42911697 | C | A |  | -0.01846 | 0.003183 | 6.65E-09 |  | 0.006666 | 0.014322 | 0.64 |
| rs77542162 | 17 | 67081278 | G | A |  | -0.09384 | 0.010274 | 6.68E-20 |  | 0.021318 | 0.030656 | 0.49 |
| rs12601079 | 17 | 76400329 | G | A |  | 0.025685 | 0.003169 | 5.33E-16 |  | 0.010153 | 0.014259 | 0.48 |
| rs12958584 | 18 | 19632380 | T | C |  | 0.029224 | 0.004607 | 2.26E-10 |  | -0.00262 | 0.010874 | 0.81 |
| rs74489351 | 18 | 46578242 | A | C |  | -0.09121 | 0.015029 | 1.29E-09 |  | 0.000735 | 0.041391 | 0.99 |
| rs77960347 | 18 | 47109955 | G | A |  | -0.0839 | 0.013163 | 1.85E-10 |  | -0.00051 | 0.032831 | 0.99 |
| rs34232444 | 19 | 4965404 | C | T |  | -0.01945 | 0.003223 | 1.59E-09 |  | 0.017474 | 0.014664 | 0.23 |
| rs4804833 | 19 | 7970635 | A | G |  | 0.017301 | 0.003133 | 3.36E-08 |  | 0.003058 | 0.007519 | 0.68 |
| rs116483979 | 19 | 8456738 | T | C |  | -0.0508 | 0.008996 | 1.64E-08 |  | 0.007467 | 0.019858 | 0.71 |
| rs10412048 | 19 | 11193949 | G | A |  | 0.090636 | 0.004695 | 6.05E-83 |  | -0.0251 | 0.011465 | 0.03 |
| rs2738447 | 19 | 11227480 | A | C |  | 0.020781 | 0.003116 | 2.57E-11 |  | -0.01461 | 0.007488 | 0.05 |
| rs8112221 | 19 | 16094321 | A | T |  | -0.0187 | 0.003381 | 3.23E-08 |  | 0.009825 | 0.008124 | 0.23 |
| rs62115559 | 19 | 44649737 | G | A |  | 0.119329 | 0.010942 | 1.10E-27 |  | 0.00994 | 0.028963 | 0.73 |
| rs185920692 | 19 | 44718619 | T | C |  | 0.090043 | 0.013163 | 7.90E-12 |  | -0.05727 | 0.033454 | 0.09 |
| rs62117160 | 19 | 45232161 | A | G |  | 0.324978 | 0.0073 | 1.00E-200 |  | -0.04766 | 0.020813 | 0.02 |
| rs112450640 | 19 | 45296364 | A | G |  | 0.424904 | 0.011072 | 1.00E-200 |  | -0.0303 | 0.02775 | 0.27 |
| rs41290102 | 19 | 45371188 | T | C |  | -0.07188 | 0.013168 | 4.80E-08 |  | 0.020074 | 0.028781 | 0.49 |
| rs79701229 | 19 | 45384931 | A | G |  | -0.08645 | 0.013516 | 1.59E-10 |  | -0.0331 | 0.036719 | 0.37 |
| rs12691088 | 19 | 45418486 | A | G |  | -0.10476 | 0.01283 | 3.23E-16 |  | 0.014706 | 0.027694 | 0.60 |
| rs140480140 | 19 | 45421650 | A | G |  | -0.06906 | 0.009278 | 9.82E-14 |  | 0.029247 | 0.045892 | 0.52 |
| rs62117257 | 19 | 45550013 | T | C |  | -0.03439 | 0.005696 | 1.57E-09 |  | 0.006444 | 0.015271 | 0.67 |
| rs17875609 | 19 | 45820144 | T | C |  | 0.096115 | 0.010671 | 2.13E-19 |  | -0.01822 | 0.028489 | 0.52 |
| rs10408163 | 19 | 47597102 | T | C |  | -0.02275 | 0.003378 | 1.63E-11 |  | 0.0071 | 0.008147 | 0.38 |
| rs416867 | 19 | 54796630 | A | G |  | -0.02472 | 0.003677 | 1.80E-11 |  | 0.005348 | 0.009139 | 0.56 |
| rs11879347^a^ | 19 | 56093556 | T | A |  | 0.030127 | 0.004777 | 2.85E-10 |  | 6.27E-05 | 0.011529 | 1.00 |
| rs686548 | 20 | 12973521 | A | T |  | 0.029298 | 0.003135 | 9.22E-21 |  | -0.00457 | 0.007572 | 0.55 |
| rs2618566 | 20 | 17844684 | G | T |  | -0.01764 | 0.003226 | 4.51E-08 |  | -0.00993 | 0.00795 | 0.21 |
| rs7679 | 20 | 44576502 | C | T |  | 0.0676 | 0.003943 | 7.74E-66 |  | 0.006449 | 0.009461 | 0.50 |
| rs8126001 | 20 | 62711459 | T | C |  | -0.01869 | 0.003071 | 1.17E-09 |  | 0.006222 | 0.007539 | 0.41 |
| rs738409 | 22 | 44324727 | G | C |  | 0.022772 | 0.003702 | 7.70E-10 |  | 0.007853 | 0.008906 | 0.38 |

Abbreviations: Chr., chromosome; IDL, intermediate-density lipoprotein; SE, standard error.

^a^ Proxy variant.


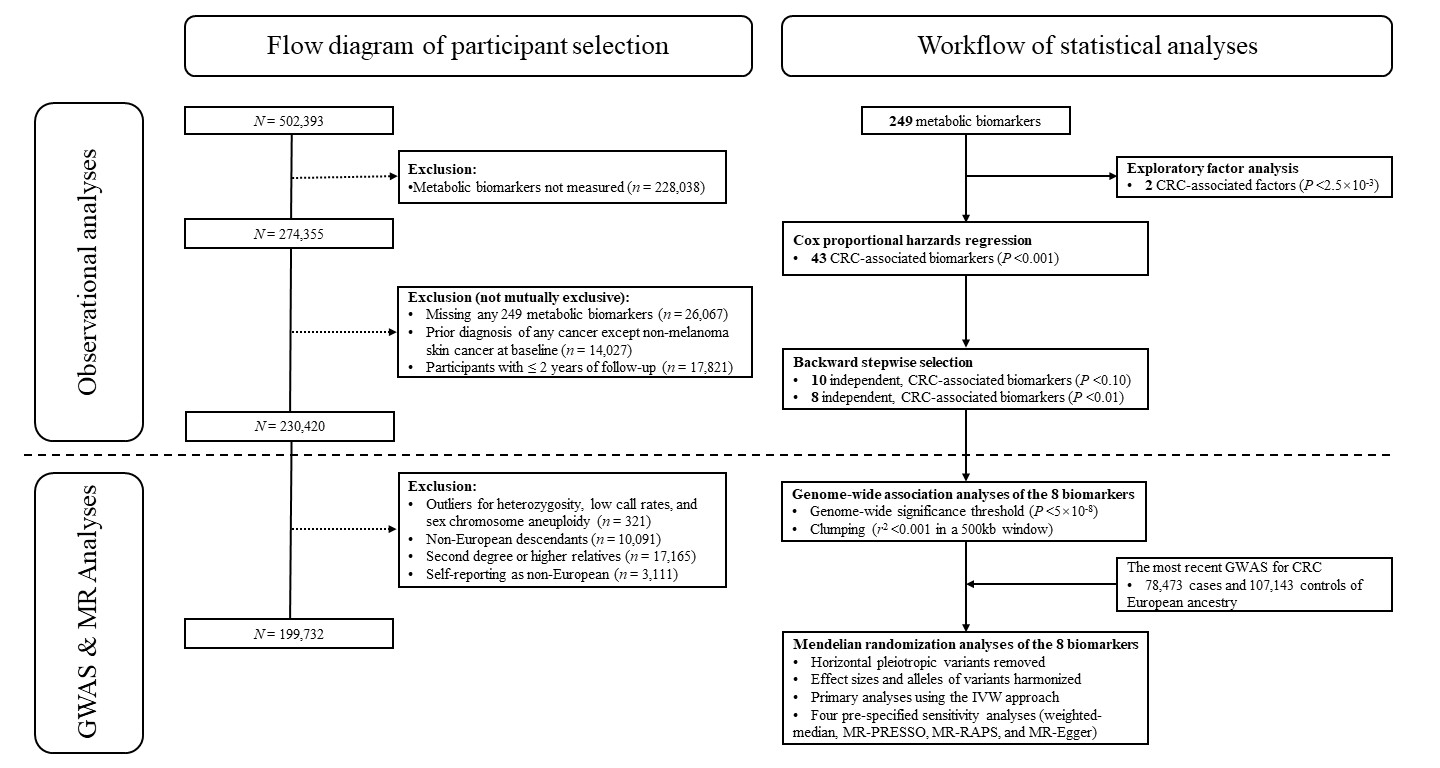


**Supplementary Figure S1**. Overview of study participants selection and workflow of statistical analyses for the current study, UK Biobank. The numbers of participants at each exclusion stage and the exclusion criteria were shown on the left. The numbers of metabolic biomarkers included in statistical analyses were highlighted on the right. CRC, colorectal cancer; GWAS, genome-wide association study; IVW, inverse-variance weighted; MR, Mendelian randomization; MR-PRESSO, MR-pleiotropy residual sum and outlier; MR-RAPS, MR-robust adjusted profiles score.


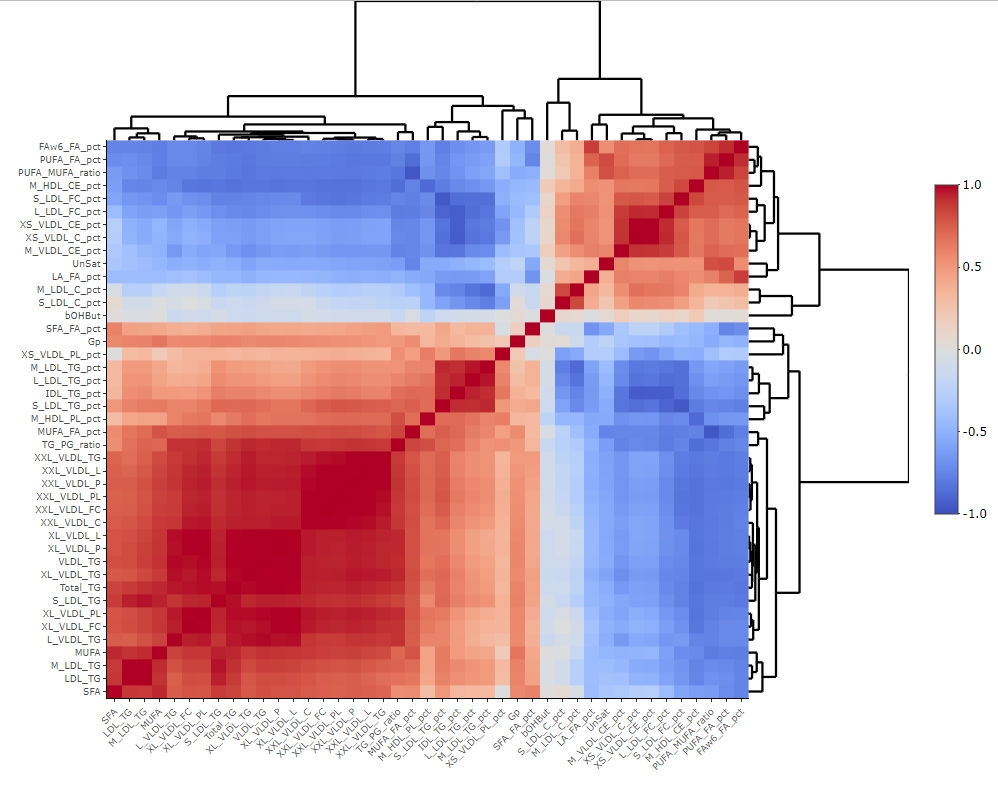


**Supplementary Figure S2. Correlation matrix for the 43 CRC-associated metabolic biomarkers in the UK Biobank cohort.** Full names of labeled biomarkers are in the Supplementary Table S1.


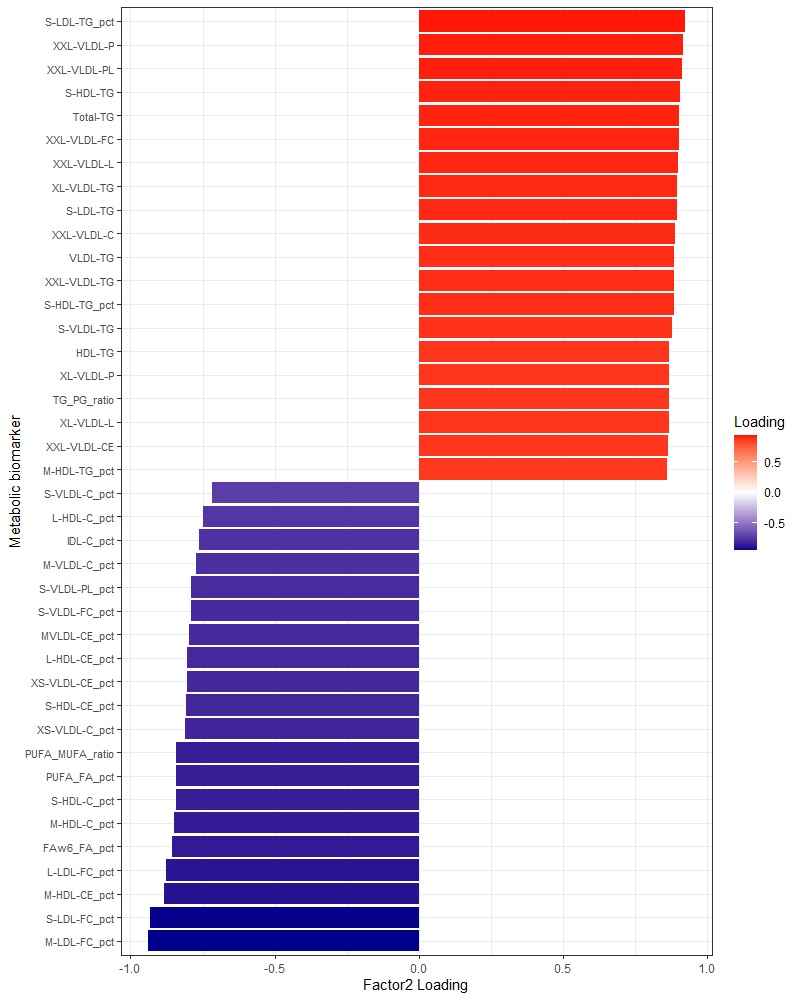


**Supplementary Figure S3. Loadings of the top 40 contributing (20 positive and 20 negative) biomarkers to factor 2.**


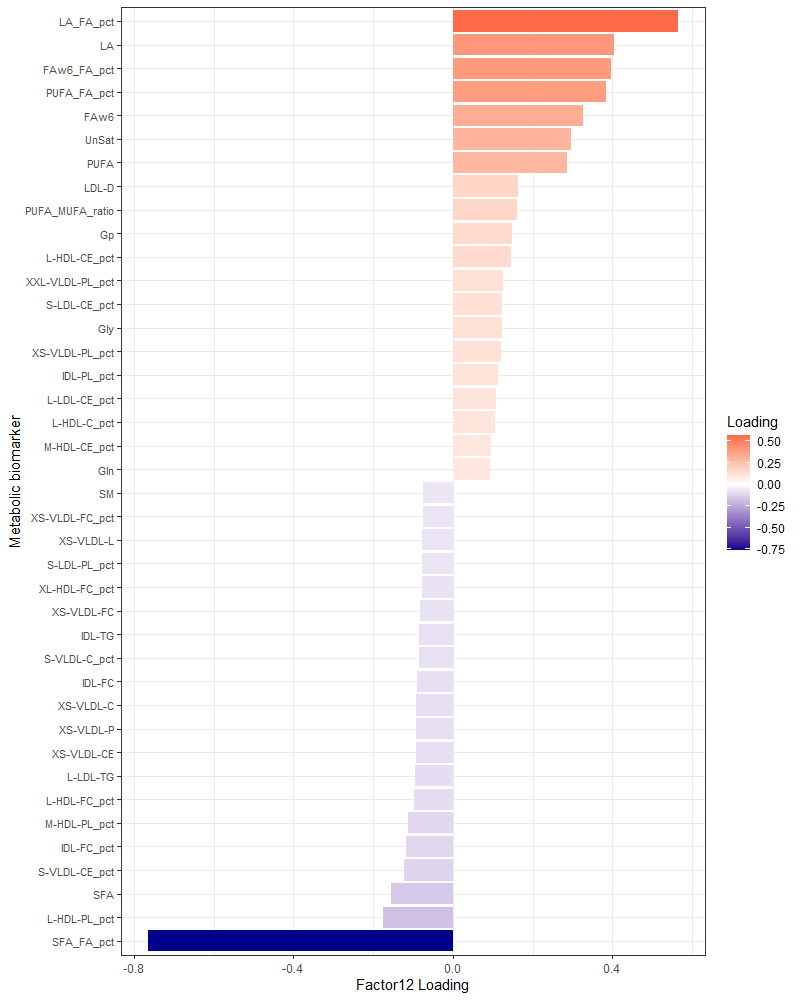


**Supplementary Figure S4. Loadings of the top 40 contributing (20 positive and 20 negative) biomarkers to factor 12.**


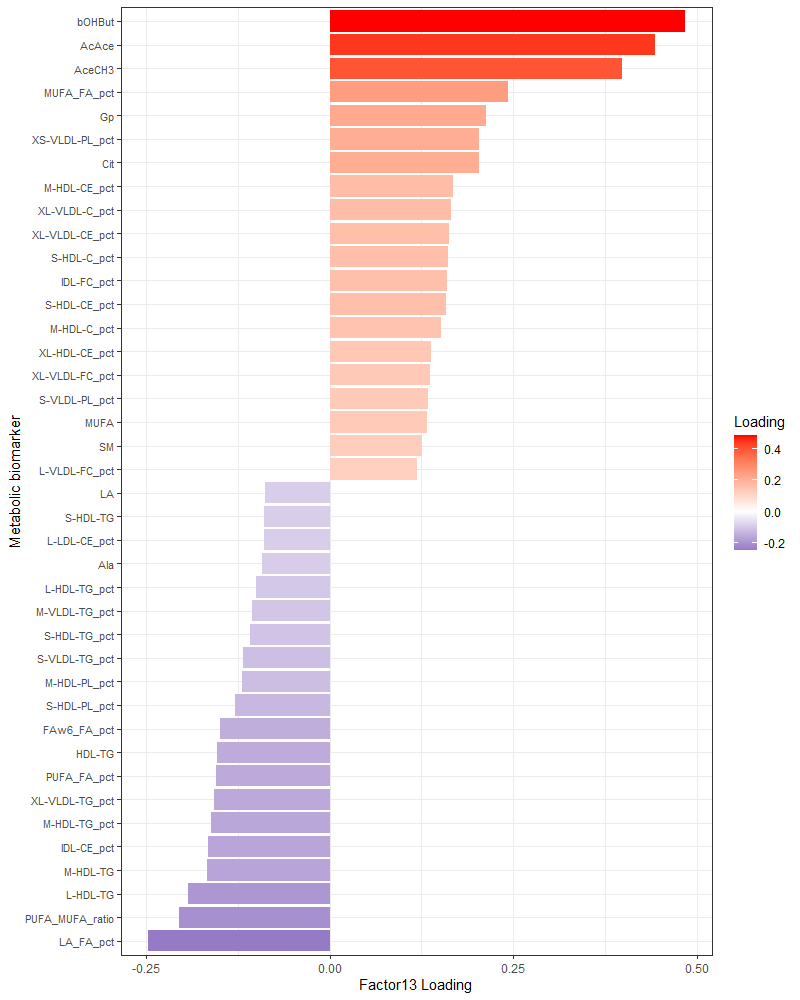


**Supplementary Figure S5. Loadings of the top 40 contributing (20 positive and 20 negative) biomarkers to factor 13.**
